# NSAIDs Modulate Clonal Evolution in Barrett's Esophagus

**DOI:** 10.1371/journal.pgen.1003553

**Published:** 2013-06-13

**Authors:** Rumen L. Kostadinov, Mary K. Kuhner, Xiaohong Li, Carissa A. Sanchez, Patricia C. Galipeau, Thomas G. Paulson, Cassandra L. Sather, Amitabh Srivastava, Robert D. Odze, Patricia L. Blount, Thomas L. Vaughan, Brian J. Reid, Carlo C. Maley

**Affiliations:** 1 Genomics and Computational Biology Graduate Program, University of Pennsylvania, Philadelphia, Pennsylvania, United States of America; 2 The Wistar Institute, Philadelphia, Pennsylvania, United States of America; 3 Department of Biostatistics, Johns Hopkins Bloomberg School of Public Health, Baltimore, Maryland, United States of America; 4 Department of Genome Sciences, University of Washington, Seattle, Washington, United States of America; 5 Division of Human Biology, Fred Hutchinson Cancer Research Center, Seattle, Washington, United States of America; 6 Division of Public Health Sciences, Fred Hutchinson Cancer Research Center, Seattle, Washington, United States of America; 7 Genomics Resource, DNA Array Laboratory, Fred Hutchinson Cancer Research Center, Seattle, Washington, United States of America; 8 Department of Pathology, Brigham and Women's Hospital, Harvard Medical School, Boston, Massachusetts, United States of America; 9 Department of Medicine, University of Washington, Seattle, Washington, United States of America; 10 Center for Evolution and Cancer, Helen Diller Family Comprehensive Cancer Center, Department of Surgery, University of California San Francisco, San Francisco, California, United States of America; HudsonAlpha Institute for Biotechnology, United States of America

## Abstract

Cancer is considered an outcome of decades-long clonal evolution fueled by acquisition of somatic genomic abnormalities (SGAs). Non-steroidal anti-inflammatory drugs (NSAIDs) have been shown to reduce cancer risk, including risk of progression from Barrett's esophagus (BE) to esophageal adenocarcinoma (EA). However, the cancer chemopreventive mechanisms of NSAIDs are not fully understood. We hypothesized that NSAIDs modulate clonal evolution by reducing SGA acquisition rate. We evaluated thirteen individuals with BE. Eleven had not used NSAIDs for 6.2±3.5 (mean±standard deviation) years and then began using NSAIDs for 5.6±2.7 years, whereas two had used NSAIDs for 3.3±1.4 years and then discontinued use for 7.9±0.7 years. 161 BE biopsies, collected at 5–8 time points over 6.4–19 years, were analyzed using 1Million-SNP arrays to detect SGAs. Even in the earliest biopsies there were many SGAs (284±246 in 10/13 and 1442±560 in 3/13 individuals) and in most individuals the number of SGAs changed little over time, with both increases and decreases in SGAs detected. The estimated SGA rate was 7.8 per genome per year (95% support interval [SI], 7.1–8.6) off-NSAIDs and 0.6 (95% SI 0.3–1.5) on-NSAIDs. Twelve individuals did not progress to EA. In ten we detected 279±86 SGAs affecting 53±30 Mb of the genome per biopsy per time point and in two we detected 1,463±375 SGAs affecting 180±100 Mb. In one individual who progressed to EA we detected a clone having 2,291±78 SGAs affecting 588±18 Mb of the genome at three time points in the last three of 11.4 years of follow-up. NSAIDs were associated with reduced rate of acquisition of SGAs in eleven of thirteen individuals. Barrett's cells maintained relative equilibrium level of SGAs over time with occasional punctuations by expansion of clones having massive amount of SGAs.

## Author Summary

Cancer is a disease that develops over decades as result of accumulation of abnormalities in the genomes of otherwise normal cells. Cells in tumors compete for space and resources. Those cells able to survive the Darwinian struggle for existence within tissues progressively evolve uncontrolled growth and in some cases this results in cancer. Aspirin and other non-steroidal anti-inflammatory drugs (NSAIDs) reduce death rate from multiple types of cancer by about 20%. However, the mechanisms by which NSAIDs act to prevent cancer are not fully understood. By examining thirteen individuals with Barrett's esophagus over time, we showed that the rate of accumulation of genomic abnormalities decreased when most individuals started taking NSAIDs. We also observed that, surprisingly, the number of abnormalities in the Barrett's tissues did not increase much over decades. However, in one individual who progressed to esophageal cancer, we observed massive genomic abnormalities affecting 19% of the genome. These findings suggest that NSAIDs may prevent cancer by reducing the accumulation of genomic abnormalities over time and that detection of stable versus unstable genomes may be used in the clinic to help manage treatment options in Barrett's esophagus.

## Introduction

Clonal evolution is a theory that explains the phenomenon of the progressive morphological and genetic change of somatic cell populations from normal homeostatic cell division and death within tissues to abnormal neoplastic growth and cancerous spatial expansion within and across tissues [1]–[3]. Clonal evolution is the Darwinian evolution by natural selection of asexually (mitotically) dividing somatic cells. Somatic genomic abnormalities (SGA), such as copy number alterations and loss of heterozygosity (LOH), can be used as polymorphic DNA markers for identifying evolving clones. Strictly defined, a clone is a genetically identical subpopulation of cells within the cell population of a tissue, that descends from a most recent common ancestor (MRCA) cell and therefore all of the clone's cells inherit the SGAs that were originally present in the MRCA cell. However, a commonly used, relaxed definition of a clone is descent with modification from a MRCA cell, which allows for accumulation of additional SGA heterogeneity among the cells of the clone. A clone ideally represents the shared cell lineage history of a subpopulation of cells. The acquisition of SGA variability (SGA polymorphism) over the course of cell division allows for classification of cell subpopulations into clones. In the remainder of this study, we use clone in its relaxed definition and we estimate phylogenetic trees from acquired SGA variability to qualitatively describe relatedness among evolving clones. In other words, we call a clone a set of biopsies that share a large number of SGA features by descent, or from a phylogenetic tree point of view, a set of tips (taxa) of the phylogenetic tree, which are more related than others (i.e. cluster together as a clade), but which are not necessarily identical (identical tips will have interconnecting branches with zero lengths). The generation of new clones is stochastic and the change in clones' frequencies in the population is determined by clones' relative fitness as well as stochastic effects (genetic drift). New adaptive and new neutral clones can arise stochastically over time [4] with every newly acquired SGA that does or does not affect fitness, respectively. Though adaptive mutations are thought to drive clonal expansions, it is an open question if adaptive clones tend to expand to fill much or all of the BE segment, or if they tend to remain relatively localized [5]. In order to prevent progression to cancer, mechanisms that modulate clonal evolution by either preventing or managing SGA acquisition and/or the spread of SGA-containing clones need to be elucidated.

Barrett's esophagus (BE) is a condition of the distal esophagus in which the normal stratified squamous epithelium is replaced by columnar epithelium with intestinal metaplasia [6]. BE is thought to develop as a complication of chronic gastroesophageal reflux disease (GERD) and individuals with BE are at increased risk of progression to esophageal adenocarcinoma (EA): 1–7 persons with BE progress to EA per 1000 person-years [7], [8]. Strategies for early detection and prevention of esophageal adenocarcinoma have focused on all aspects of the GERD-BE-EA sequence: acid suppression medications, anti-reflux surgery, esophagectomy, ablation of BE, endoscopic biopsy surveillance of BE, and chemoprevention using aspirin or other non-steroidal anti-inflammatory drugs (NSAIDs) [6], [9]. BE is a condition in which clonal evolution can be studied *in vivo*, since a standard of care is periodic endoscopic surveillance, allowing studies of clonal evolutionary dynamics over time.

Genomic instability is a common feature of solid cancers [1], [10]–[13]. In a recent study, Beroukhim et al. evaluated 3131 cancer specimens from 26 histologic types and 1480 normal tissue specimens and found that copy number gains and losses affected 17% and 16% of the genome in a typical cancer specimen and only 0.35% and 0.1% of the genome in a typical normal tissue specimen [14]. Despite the recent massive accumulation of data on genomic alterations in cancers from the Cancer Genome Atlas and the International Cancer Genome Consortium initiatives, as well as phylogenetic reconstruction of lineages within tumors [15]–[18], theoretical modeling of the generative process (clonal evolution) producing the observed SGA patterns and underlying neoplastic progression has remained limited [2], [3], [15], [19]. BE is associated with genomic instability and acquired SGA [20]–[23] allowing analysis of the acquisition of SGA over time. This provides data for estimating SGA acquisition rate that is a key parameter of clonal evolution.

NSAID use significantly reduces the incidence and mortality rates of many types of cancer, including esophageal adenocarcinoma [24]–[29]. Rothwell et al. showed that the hazard ratio for cancer incidence of NSAID users vs. NSAID non-users was 0.66 (95% CI 0.50–0.87); however a robust NSAID cancer preventive effect manifests significantly only after ≥5 years of regular use [24]. The majority of epidemiological studies in BE suggest that NSAID use in individuals with BE reduces risk of developing EA [25]–[28]. Specifically, Vaughan et al. evaluated 350 individuals followed up for a median of 5.4 years (range 0.2–8.9) and showed that the 5-year cumulative incidence of EA was 14.3% (95% CI 9.3–21.6) for NSAID never users compared to 6.6% (3.1–13.6) for current NSAID users and that the hazard ratio for EA incidence of NSAID users vs. NSAID non-users was 0.20 (95% CI 0.10–0.41) [27]. Galipeau et al. showed that NSAID use reduced the 10-year cumulative incidence of esophageal adenocarcinoma from 79% to 30% in individuals with BE who had one or more somatic genomic abnormalities detected at baseline endoscopy, which included DNA content tetraploidy and/or aneuploidy, assayed by DNA content flow cytometry, or genetic abnormalities, such as loss of heterozygosity (LOH) on chromosomes 9p and 17p, assayed by PCR of small tandem repeat (STR) loci [29]. NSAID use for chemoprevention is attractive due to the widespread use and low toxicity and side effects of that class of drugs; however the molecular mechanisms underlying the NSAID cancer preventive effect are not fully understood. In this study, our aim was to evaluate the effect of NSAIDs on the accumulation of somatic genomic abnormalities by evaluating the entire genome (1 Million SNP loci) for SGA. We hypothesized that NSAID use modulates clonal evolution by reducing the prevalence of SGA by either reducing the incidence of SGA over time (SGA rate: number of SGAs acquired per genome per year) or interfering with the expansion of lineages bearing newly acquired SGAs over time.

**Figure 1:**
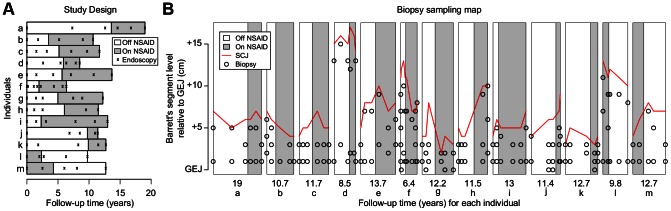
Study design and biopsy sampling. Throughout this figure, white indicates time off NSAIDs and gray indicates time on NSAIDs. (Panel A) Thirteen individuals with BE, showing times of endoscopies as black x's, and indicating time on and off NSAIDs. (Panel B) The temporal and spatial location of all biopsies in the study. Red lines show the extent of Barrett's segment from the gastroesophageal junction (GEJ) to the squamocolumnar junction (SCJ). The Y-axis is measured in cm from the GEJ. The X-axis is scaled in years of follow-up, with the total amount of follow-up for each individual indicated below the data for that individual. Small black circles indicate the locations of the biopsies that were assayed in this study.

To test this hypothesis, we used a prospective observational crossover study design: a longitudinal study in which the sequence of NSAID use was recorded for each individual during the follow-up period (Figure 1A). We selected thirteen individuals with BE from our cohort, who had endoscopic follow-up of mean 11.8±3 years (range: 6.4–19) and who began or discontinued NSAID use exactly once during follow-up. All thirteen individuals had to have at least two consecutive time points (≥6 biopsies) off NSAIDs and at least two consecutive time points (additional ≥6 biopsies) on NSAIDs (time and locations of all the biopsies are shown in Figure 1B). To estimate SGA prevalence in biopsies on and off NSAIDs we used summary statistics of observed patterns of SGA; to estimate SGA rates on and off NSAIDs we used an evolutionary analysis of observed SGA patterns to take into account SGA phylogenetic identity by descent. Drummond et al. showed that mutation rates can be estimated from longitudinal samples in virus populations using coalescent and phylogenetic methods within a Bayesian Markov Chain Monte Carlo framework for sampling model parameter space (BEAST package, Bayesian Evolutionary Analysis Sampling Trees) [30], [31]. This takes into account the fact that samples at later time points are neither direct descendants of samples from earlier time points, nor independent samples, but rather share common ancestors in a phylogeny that represents their evolutionary relationships. BEAST simultaneously estimates the mutation rates, population sizes, and the forest of most likely phylogenetic trees within an individual's Barrett's segment. We adapted BEAST to separately estimate SGA acquisition rates on and off NSAIDs. Thus, the crossover study design provided 13 independent tests of the hypothesis of NSAID-associated reduction in SGA acquisition rate since every individual had both on and off NSAID periods and SGA acquisition during those periods.

## Results

**Figure 2:**
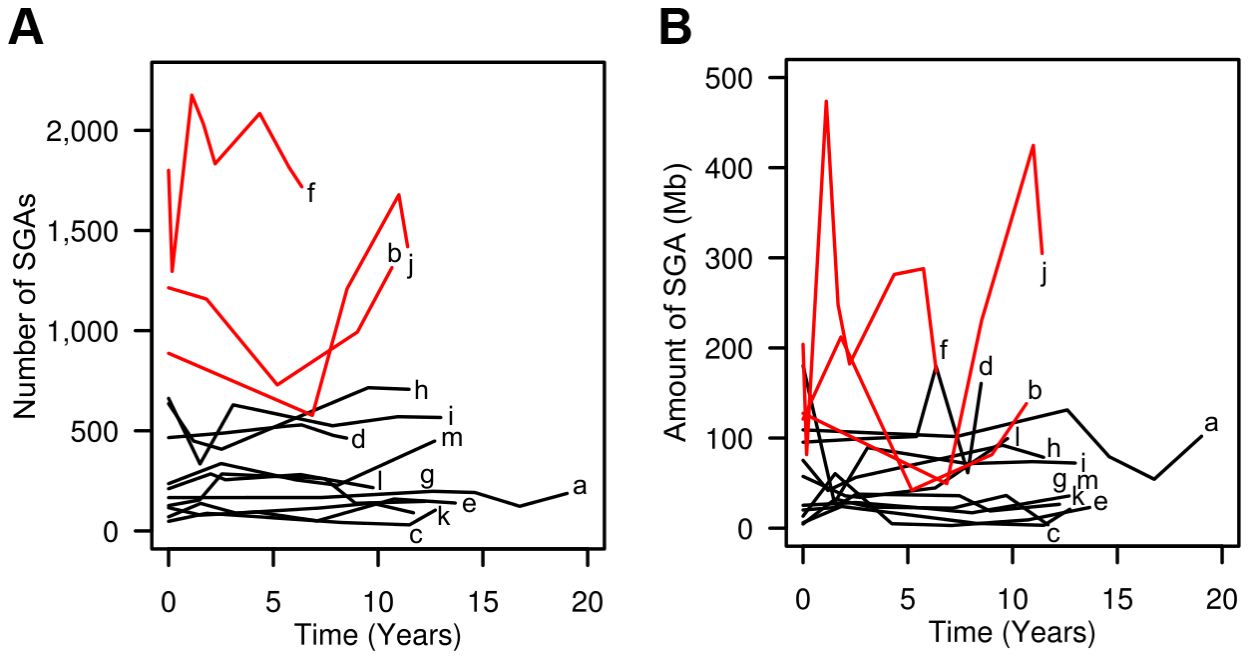
SGA remains approximately constant over time in most individuals. Black lines indicate 10 individuals with apparent evolutionary stasis and red lines indicate 3 individuals with apparent increase in SGA over time. (Panel A) Mean number of SGA lesions. Solid lines connect the means at each time point for all individuals (a–m), where the symbols a–m are plotted at the end of the lines. (Panel B) Mean amount of genome affected by SGA.

We evaluated the dynamics of detected SGAs over time. The mean number of SGAs and the proportion of the genome they affected did not obviously increase over time, for as many as 19 years (e.g., Figure 2, individual a). Individuals b, f, and j, shown in red in Figure 2A and 2B, showed much greater variation in detected SGA per biopsy, per time point, compared to the rest of the individuals, shown in black. Progression to EA was not part of our study inclusion criteria, and individual j was the only individual who progressed. Individual f did not progress to EA, but rather opted for esophagectomy for high-grade dysplasia after 6.4 years of follow-up and subsequently died of a different cancer 11.9 years later. In individuals b, f, and j, the mean (± standard deviation) number of SGAs per biopsy per time point was 1,082±177, 1,844±573, and 1,154±746, and the amount of genome affected by SGAs was 119±79 Mb, 242±121 Mb, and 227±222 Mb, respectively. In the rest of the individuals, the mean number of SGAs per genome per time point was 279±86 and the amount of genome affected was 53±30 Mb. Assuming a human genome length of 3,164 Mb (Human genome GCRh37.p5 assembly), individuals b, f, and j had 3.8±2.5%, 7.6±3.8%, and 7.2±7% altered somatic genome per time point, compared to 1.7±0.9% altered somatic genome in the rest of the individuals. The number of events and total sizes for each type of lesion, as well as the detected presence of within-biopsy heterogeneity, in each biopsy are shown in Table S1 and Figures S1, S2. In 10 out of 13 individuals (everyone except b, f, and j) the number of SGAs remained relatively constant over time. Different biopsies from these individuals displayed different SGA lesions, leading to upward and downward fluctuations in mean number or genome amount of SGA. In some cases a biopsy from an earlier time point had more genomic lesions than a biopsy at a later time point, suggesting that we sampled a persistent but more ancestral clone at the later time point. For example, the first biopsy in individuals i and l had the highest number of SGAs compared to biopsies at later time points (Figure S1 and Table S1). Overall, the dynamics of SGAs in BE segments appear more consistent with equilibrium over time rather than with accumulation of SGAs affecting ever greater portions of the genome over time.

**Figure 3:**
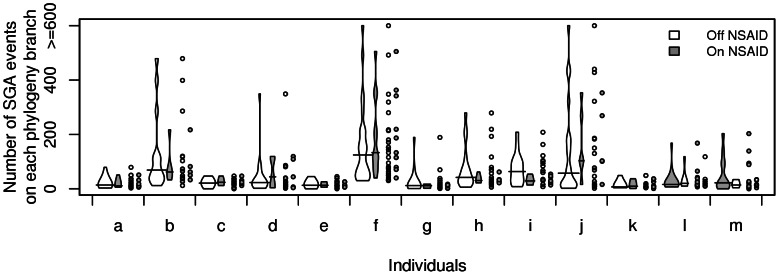
PAUP analysis of SGA events distribution on lineages of within-individual phylogenies suggests that the greatest number of SGA events occurred on outlier minority of lineages that evolved during off-NSAID periods. Two violin plots show the distribution of lineage lengths (estimated number of SGA events) that evolved during off-NSAID (white) and on-NSAID (grey) periods within an individual phylogeny (solid line across the violin denotes the median). The volumes of a pair of violin plots are scaled relative to the number of data points the pair contains. Scatter plots show the raw data underlying the violin plots and illustrate the outliers. The majority of individual phylogenies (a, b, d, e, f, g, h, i, j, k) show an off-NSAID lineage containing the maximum number of SGA events.

**Figure 4:**
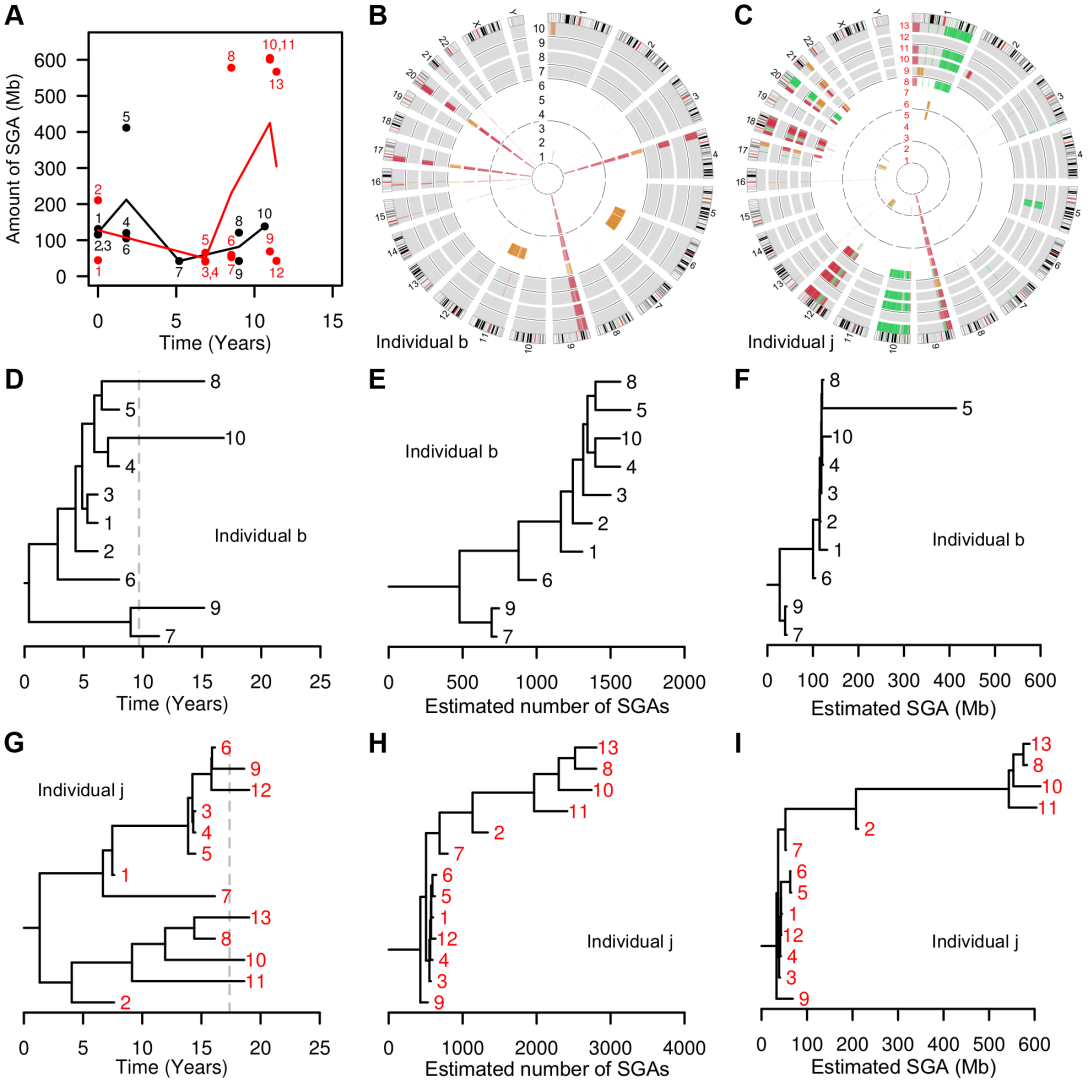
Clonal evolution in individuals b and j. (Panel A) Solid lines connect the mean amount of SGA detected across biopsies at each time point; dots correspond to individual biopsies. In individual b (black line), we observed evolutionary stasis with mean SGA remaining at 119±79 Mb over more than a decade of follow-up. In individual j (red line), a massive burst of SGA was detected in year 8.5; three years later individual j progressed to esophageal adenocarcinoma. Individual b started NSAIDs after year 5, while individual j started NSAIDs only after year 10. (Panels B and C) Circos plots showing genome-wide views of SGA over time. Each ring, labeled with a biopsy number, represents whole-genome SGA data from a different biopsy, with earlier samples toward the center. Thin black line rings separate endoscopies (time points), white background shows time periods off-NSAIDs and gray background shows time periods on-NSAIDs. Within the rings, black segments designate homozygous deletion, red single copy loss, orange copy-neutral LOH, and green copy gain. (Panel B) Circos plot of individual b. Note the appearance of “new” whole chromosome LOH at chromosome 6 and 11 in biopsy 5, taken during the off-NSAIDs period, and the detection of a minimally mutated clone in biopsies 9 and 7, taken during the on-NSAIDs period. (Panel C) Circos plot of individual j. A massive burst of SGAs was detected first in biopsy 8, in year 8.5, before the individual began regular NSAID use. Biopsy 2 (second inner ring), taken at the baseline endoscopy 8.5 years prior to the burst, shared a subset of the SGAs seen in the massively altered clone (chromosomes 10, 12, 17 and 18), and thus is likely an early example of its lineage. (Panels D and G) Consensus phylogenetic trees estimated by BEAST reveal long-term co-existence of clones. Branch lengths are scaled according to time, the tips of the phylogeny are biopsies aligned on the x-axis according to their sampling time, and all internal nodes are estimated coalescence times assuming a logistic population growth. Dashed gray line represents the start of NSAID use. (Panels E, F, H, I) Maximum parsimony trees estimated by PAUP reveal the ancestral relationships among biopsies based on shared SGA characters. Differences between the topology of the trees estimated by PAUP and BEAST are typically due to poorly supported short branches and do not affect the analyses of SGA acquisition rates. Branch lengths are scaled according to estimated number of SGAs (Panels E, H) or the amount of genome affected by SGA (Panels F, I). Note that these trees appear very different from those estimated by BEAST as the BEAST branch lengths are scaled by inferred time depth, and the rate of SGA accumulation appears highly variable with time.

All biopsies sampled from an individual are related due to common ancestry arising from, or prior to, the time of origination of the Barrett's segment through the process of cell and crypt division. Therefore, we expect biopsies to share some SGAs and we accounted for the statistical non-independence of observed SGA across biopsies, due to common ancestry, by estimating maximum parsimony phylogenetic trees for each individual using SGAs as characters and biopsies as taxa. We estimated phylogenetic trees using PAUP and found that for the majority of individuals the maximum number of SGA events per lineage occurred in branches when the individual was off NSAIDs as opposed to in branches during periods when they were taking NSAIDs (Figure 3). For example, PAUP analysis showed massive SGA on a single off-NSAID lineage in individual j, which resulted in subsequent SGA-heavy descendant on-NSAID lineages (phylogeny of individual j, Figure 4H,I).

**Figure 5:**
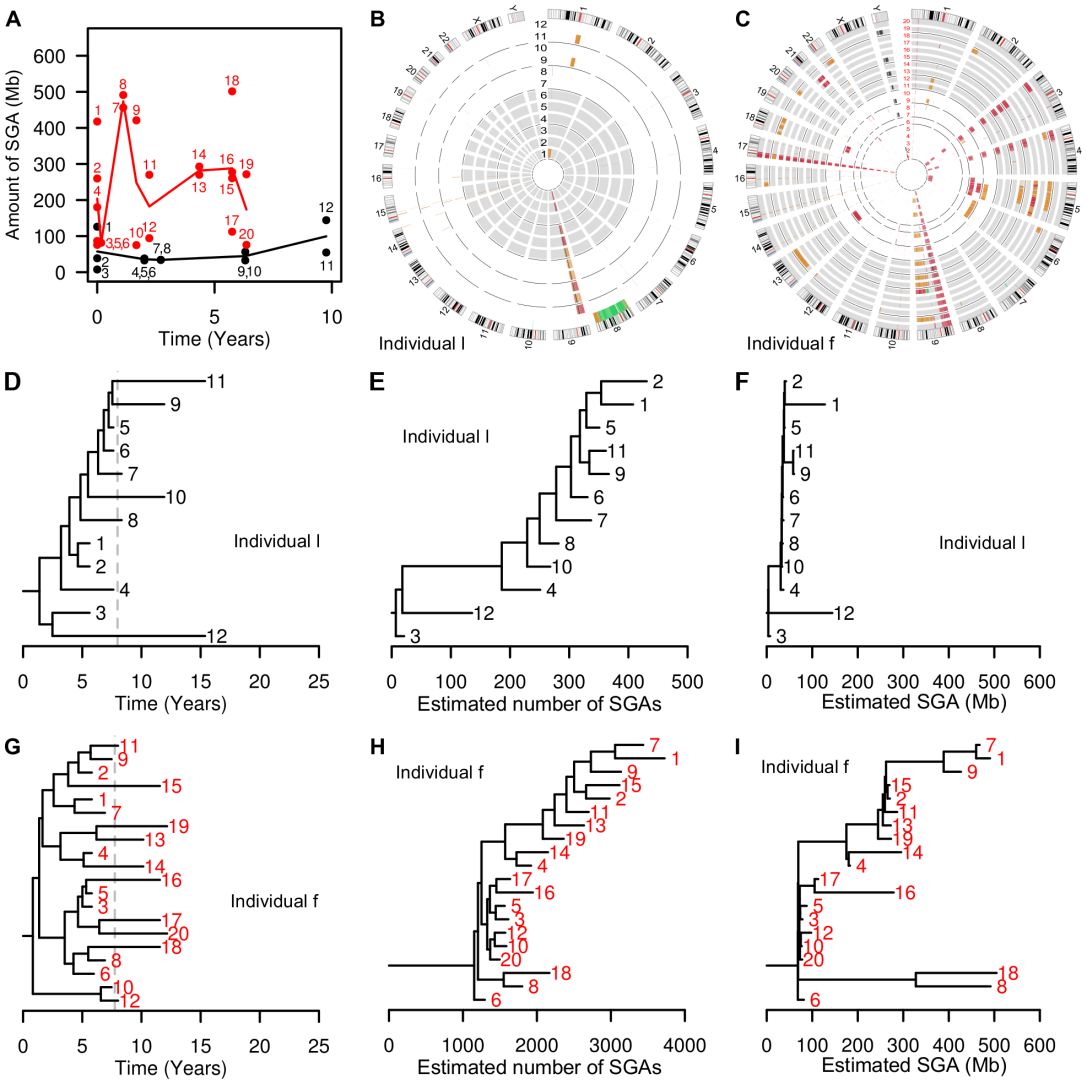
Clonal evolution in individuals l and f. (Panel A) Amount of SGA in biopsies (dots) and mean SGA over all biopsies at that time point (lines) for individual l (black) and individual f (red). (Panels B and C) Circos plots showing genome-wide view of SGA over time (see Figure 4 legend for details). (Panel B) Individual l. During the off-NSAID period we detected a whole-chromosome gain of chromosome 8 in biopsy 12 (green band) and copy-neutral LOH events on chromosome 1 in biopsies 9 and 11 (orange bands). (Panel C) We detected 1,844±573 of SGAs in individual f, who opted for esophagectomy for high-grade dysplasia after 6.4 years of follow-up and died of another cancer 11.9 years later. (Panels D, E, F) BEAST consensus tree, parsimony tree of SGA number, and parsimony tree of SGA quantity for individual l. (Panels G, H, I) BEAST consensus tree, parsimony tree of SGA number, and parsimony tree of SGA quantity for individual f. In both individuals, BEAST trees reveal long-term co-existence of multiple clones. and maximum parsimony trees reveal an underlying progressive evolution of SGA events irrespective of time. Phylogenetic trees generated as indicated in the legend to Figure 4.

While the natural history of clonal evolution is different in each individual, some common patterns can be discerned. The majority of SGAs are present in the first time point, with little accumulation of SGAs afterwards (Figure S3). In the earliest biopsies, taken at baseline endoscopy, there were many SGAs (1442±560 in individuals b, f, and j and 284±246 in the rest). This can also be seen in the radial spokes apparent in the Circos plots (Figures 4B, 4C, 5B, 5C, and S4), and also in the lesions that were detected in all biopsies of an individual (some of which are the most common lesions in BE [21], [23], on chromosomes 9p (CDKN2A) and 3p (FHIT) (Figure S5).

Multiple clones appear to co-exist over the entire period of follow-up. This can be seen in the strong spatial divergence between biopsies at the same time point in individuals a, f. g and I (Figure S6). However, within a given level (±1 cm), there was no significant increase in genetic divergence with time. While it is often assumed that the evolutionary history of a cancer involves multiple selective sweeps by new, selectively advantageous genotypes, we found only one such case of a clone that grew to stably dominate the Barrett's segment (individual h, Figures S8 and S9) over at total of 153 patient-years. Due to sampling limitations, we cannot be sure that clone drove all other clones extinct (went to fixation). Genetic divergence, based on number of SGA events, only significantly increased during follow-up in individuals b and j (Figure S6) (individuals d and f also showed increasing divergence based on amount of SGA; Figure S7) but decreased in the one individual (h) with a large clonal expansion (Figure S6). The absence of selective sweeps can also be seen in the consensus trees generated by BEAST (Figures 4D, 4G, 5D, 5G, and S8). Even in the one individual who progressed to EA, individual j, the clone with massive SGAs remained spatially localized (Figure 4). This clone is defined as the set of biopsies 8, 10, 11, and 13, which had a combined 2,291±78 SGAs affecting 588±18 Mb or 19% of the genome, and which were sampled at levels 41, 39, 41, and 40 cm in a segment that spanned levels 35–44 cm from SCJ to GEJ (Figure 4 C, G–I). Interestingly, a precursor of that clone had been detected nine years prior to its emergence (biopsy 2 in Figures 4 G–I).

We show clonal evolution in individuals b, j, and f in higher detail in Figures 4 and 5 since these individuals had a higher than average number of SGA events and amount of genome affected by SGA (Figure 2). We show clonal evolution in individual l (Figure 5) in higher detail to show clonal evolution during an on-off NSAID use pattern. In addition, the SGA amount in individual l is close to the mean SGA amount in all individuals, except b, f, and j, while SGA amount in individual f is higher than the mean (Figure 2) and using Circos plots side-by-side contrasts qualitatively SGA amount and SGA chromosomal location between individuals l and f (Figure 5 B,C).

In summary, the majority of individuals showed no dramatic accumulation of new SGAs consistent with long-term evolutionary stasis during follow-up (Circos plots in Figures 4, 5, S1, S2), and the one progressor to EA, individual j, showed that evolutionary stasis can be punctuated by the expansion of a clone with massive amount of SGAs (Figure 4 C,G–I).

The maximum parsimony phylogenetic analysis revealed the shared common ancestry of biopsies within an individual based on SGA homology. Inferred PAUP phylogenetic trees, which had branches scaled by the estimated number of shared SGA events in Figures 4E,H, 5E,H, and Figure S9, showed significantly imbalanced tree shapes (Table S2) for all individuals, except individual f and j. When we rescaled the branch lengths of the same phylogenetic trees by the amount of genome affected in Figures 4F, I, 5F, I, and Figure S10, the trees showed that within an individual the majority of biopsies are closely related and only few biopsies or lineages diverge dramatically from the majority cluster, which is indicative of SGA bursts.

**Figure 6:**
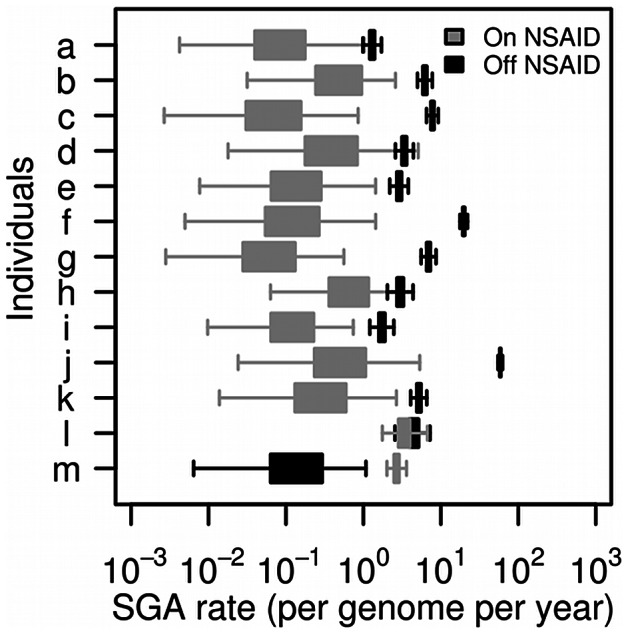
BEAST analysis suggests that NSAID use reduces the SGA rate (number of SGA events per genome per year). For all individuals (a–m), the mean off-NSAID SGA rate was 7.8 (95% support interval [SI]: 7.1–8.6) and the mean on-NSIAD SGA rate was 0.6 (95% SI: 0.3–1.5). For individuals a–k, the mean off-NSAID SGA rate was 8.8 (95% SI: 8.1–9.5,), whereas the mean on-NSAID SGA rate was 0.2 (95% SI: 0.03–1.0). For the two individuals l and m that started surveillance on NSAIDs and then went off NSAIDs, there are mixed results. The mean on-NSAID SGA rate for individual l was 3.1 (95% SI: 2.2–4.7) and the mean off-NSAID SGA rate was 4.4 (95% SI: 3.1–5.9). However, for individual m the mean on-NSAID SGA rate was 2.5 (95% SI: 2.1–3.0) and the mean off-NSAID SGA rate was 0.1 (95% SI: 0.01–0.6). Note that confidence intervals are tighter for the earlier time period for each individual as more inferred ancestry events fell within that time period.

Phylogenetic methods of analysis are required to account for the complex dependency structures in samples that are related by common ancestry. Bayesian methods allow for more detailed models of evolution than parsimony methods. We tested our hypothesis that NSAID use reduces SGA acquisition rate in BE by estimating SGA acquisition rate during off-NSAID and on-NSAID periods using a custom modified version of BEAST [31]. This reconstructs the set of most likely phylogenies that relate the samples within the Barrett's segment and simultaneously estimates the SGA rates along the branches of those phylogenies during the on and off NSAIDs periods. This method is based on the coalescent in which lineages may disappear either because they are not sampled or because they go extinct. We added a new evolutionary model of SGA into BEAST in order to estimate SGA rate using Bayesian MCMC sampling (see Methods and Text S1: Equations S3–4). We excluded SGAs detected in the first time point and only measured SGAs that were detected during follow-up, in order to reduce the influence of clonal evolution that occurred prior to surveillance. For the two individuals who were already on NSAIDs when we started surveying them, and later went off NSAIDs, individual l showed a lower SGA rate on NSAIDs than off NSAIDs, but the 95% support intervals for the two rates overlap (Figure 6). In contrast, individual m showed a higher SGA rate on NSAIDs than off NSAIDs. In individuals a–k, the SGA rate on NSAIDs was approximately an order of magnitude lower than the SGA rate off NSAIDs, with non-overlapping 95% support intervals (Figure 6), which is consistent with the hypothesis that NSAID use reduces SGA acquisition rate (on average 7.8 SGAs per genome per year off-NSAID vs. on average 0.6 SGAs per genome per year on-NSAID in individuals a–k).

## Discussion

While it is clear that NSAIDs prevent many forms of cancer [24], particularly esophageal adenocarcinoma [25]–[27], the mechanism of that preventive effect is unknown. Because neoplastic progression is a process of somatic evolution, NSAIDs must affect somatic evolution in order to prevent cancer. We hypothesized that NSAIDs slow the rate of somatic evolution by lowering the mutation rate, specifically, the rate of acquisition of copy number alterations and loss of heterozygosity (SGAs). By adapting a tool from evolutionary biology (BEAST) we were able to estimate the SGA rates *in vivo* both on and off NSAIDs within the same individuals.

Our data shows that overall NSAID use is associated with an approximately 10-fold reduction in the rate of acquisition of SGAs and expansion of those lineages to detectable levels, from 7.8 SGAs per genome per year (95% support interval [SI], 7.1–8.6) to 0.6 SGAs per genome per year (Figure 6). However, this was only clear in 11 of our 13 individuals (with non-overlapping 95% support intervals in 8 of those 11). In the two individuals who stopped NSAID use during follow-up, the data does not show a significant reduction in SGA rate by NSAIDs. This may be due to the fact that these individuals were originally off NSAIDs for some unknown period of time prior to surveillance, and the lesions acquired during that time are being lumped into the on-NSAIDs SGA rate estimation. It is likely that NSAID use will not affect all individuals in the same way, and that their effects may be modulated by other factors that vary across individuals. A future larger cohort study will be required to determine if this effect generalizes to most individuals with BE.

The BEAST phylogenetic analysis assumes two constant SGA rates, one on-NSAIDs and one off-NSAIDs. To relax this assumption, we added a maximum parsimony analysis (PAUP) of the biopsy SGA data that allows for differences in SGA load on estimated lineages. In 10/13 individuals we observed the maximum SGA load occurring on an off-NSAID lineage (Figure 3). In individual j, we observed a single lineage arise during the period off NSAIDs that carried a massive SGA load, and spawned descendant lineages also with heavy SGA loads (individual j in Figure 3 and Figure 4H,I). This suggests that NSAIDs may prevent the occurrence of massive numbers of SGA on single lineages (branches of the phylogeny) or limit the clonal expansion of such lineages.

This intensive longitudinal study with 5–8 time points per individual, with 10–20 biopsies per individual, over 6.4–19 years of follow-up, revealed a number of additional surprises. There was long-term evolutionary stasis in most individuals (Figure 2), only one large clonal expansion (in individual h; Figures S8, S9), and the sudden appearance of a massively altered clone in individual j (though a precursor of that clone was detected in biopsy 2, nine years earlier; Figure 4).

The term evolutionary stasis has been used in evolutionary biology to describe a lack of phenotypic change in a species over a given timeframe [32]. Models of this stasis are based on an evolving population diffusing across a fitness plateau due to evolutionarily neutral mutations, until they find an adaptive genotype [33]–[35]. This definition of stasis predated the advent of technologies that allowed characterization of genomic alterations, large and small, that can occur in a lineage that does not lead to measurable phenotypic changes. We are using the term stasis in this study to describe genomes that maintain in equilibrium a relatively constant level of genomic alterations over time and space, in contrast to those individuals in which large scale genomic alterations develop. Our results suggest that while clones may come and go, along with the SGAs they carry, the overall population of Barrett's cells maintains an equilibrium level of SGA events, that only grows slowly if at all (Figures 2, 6). The only exception was the one individual (j) who progressed to cancer, in whom we detected large-scale SGAs just before they started using NSAIDs. The observation of long-term evolutionary stasis is consistent with the observation that BE rarely progresses to EA [7], [8] and the hypothesis that BE can function as a benign and perhaps protective evolutionary adaptation of epithelial tissue to duodenal gastroesophageal reflux [36]. Our selection criteria, both for individuals that have used NSAIDs, and for at least 4 time points over at least 4.5 years of follow-up may have also led to selection bias for individuals with evolutionary stasis in their Barrett's epithelium.

Apparent evolutionary stasis at the level of analyses of biopsies may miss ongoing accumulation of SGAs within single crypts. If those clones never grow larger than a few crypts, they would not be detected by our assays. Further work will be necessary to determine if the stasis seen at the biopsy level is a result of the lack of accumulation of SGAs in crypts or the lack of clonal expansions of those SGAs to detectable sizes. In addition, SNP arrays do not reveal point mutations, small indels, and some structural rearrangements that would be revealed by genomic sequencing. Additional analyses will be required to test if there is significant accumulation of these other genomic alterations during progression.

The dominant model of neoplastic progression, with sequential selective sweeps, is not supported by our data. There are enough novel lesions in each biopsy that if a clonal expansion was driven by a point mutation, epigenetic change, or other structural alteration not assayed by a SNP array, we would still be able to detect the expansion in the hitchhiker LOH and copy number alterations. In only one case (individual h) did we observe a clone taking over the entire Barrett's segment during follow-up, and in that individual it only happened once. Rather, we observed that after the initial expansion of the Barrett's epithelium, there is long-term coexistence of Barrett's cell lineages (Figures S8, S9, S10). In fact, there is little genetic divergence within the same level of the BE segment over time, though biopsies at different levels tend to be genetically divergent (Figures S6, S7).

We used a relatively simple model of the likelihood of SGA events in BEAST. Future work should improve on this with better models of genomic lesions as well as the inclusion of natural selection in the inferred dynamics. In addition, assaying single cells [37], [38], or single crypts, would avoid potential confusion generated by mixed clonal populations within a sample.

In summary, NSAID use in BE is associated with approximately an order of magnitude reduction in the rate of acquisition of SGA in 11 of the 13 individuals, suggesting that the pathway whereby NSAIDs exert their protective effect involves the reduction in number of SGAs or the inhibition of spread of SGA-containing clones. Our results also suggest that most genetic lesions occur prior to baseline detection in the clinic, but that during clinical management the Barrett's cells remain in equilibrium at the genome level. Measurement of mutation rates (i.e. SGA rates) *in vivo* might be used in the clinic to reduce overdiagnosis and unwarranted treatment and detection of high mutation rates or massive bursts of SGA might be used to better identify patients needing more aggressive surveillance and therapy.

## Methods

### Human subjects

Individuals were selected from the Seattle Barrett's Esophagus Study, a research cohort founded in 1983. Surveillance endoscopies were performed and biopsies were taken using a standardized four quadrant sampling protocol [39]. At endoscopy, anatomical landmarks including the gastroesophageal junction (GEJ) and squamocolumnar junction (SCJ) were noted, which define the lower (distal) and upper (proximal) boundaries, respectively, of the Barrett's segment. During an endoscopy, biopsies were taken every one or two cm along the length of the Barrett's segment. At each level, four biopsies were taken approximately at 0°, 90°, 180°, and 270° around the circumference of the esophagus for histologic evaluation. Endoscopic biopsies for molecular studies were collected in Minimal Essential Media (MEM) with 10% DMSO (Sigma #D-5879), 5% heat inactivated fetal calf serum, 5 mM Hepes buffer on ice and frozen at −70°C. In 1995 the research protocol added an epidemiologic interview in which individuals were questioned about NSAID use, as previously described [27]. In addition, the protocol added blood collection at the time of endoscopy for use as a control, since blood DNA represents putatively unaltered germline genotype.

### Study design

Individuals were selected in the cohort who had at least a 3 cm-long BE segment at baseline. Individuals were further selected based on NSAID use status changing exactly once during prospective follow-up and based on having at least two endoscopic procedures while using NSAIDs and at least two while not using NSAIDs. At least five years of follow-up was also required in order to observe evolution over time. Thirteen individuals met these inclusion criteria (Figure 1A). The history of NSAID use at each endoscopy was evaluated with a questionnaire that was also used in a US collaborative case-control study of esophageal adenocarcinoma [40]. As part of the questionnaire, individuals are shown cards (i.e., typed lists of drugs with trade names and generic names) to facilitate recall. Individuals were also asked about indications for taking NSAIDs, and reasons for stopping in those who were no longer regular users. The criterion for regular NSAID use at an endoscopy was taking an NSAID at least once per week for the last 6 months. Regular NSAID use over multiple endoscopies defines a time interval on-NSAIDs and absence of NSAID use over multiple endoscopies defines a time interval off-NSAIDs. We approximated the transition point between NSAID use and non-use by taking the middle time point equidistant between the two endoscopies when the NSAID use changed (Figure 1A, white-gray boundary). Eleven individuals (a–k) were not on NSAIDs at the start of surveillance and then went on NSAIDs (had an “off–on NSAIDs” pattern during surveillance), and two individuals (l, m) had the opposite, starting surveillance on NSAIDs and then stopping their use (an “on–off NSAIDs” pattern). The median follow-up surveillance time per individual was 11.6 years (range 6.3–19). A total of 74 endoscopies and 161 biopsies were selected (Figure 1B) as well as one blood sample for each of the thirteen individuals to serve as normal constitutive genotype control.

### Sample preparation

The 161 frozen biopsies were thawed and rinsed in Hanks buffered salt solution without divalent cations (HBSS, Gibco/BRL). Biopsies were incubated 60 minutes at room temperature in 30 mM EDTA in HBSS preheated to 37°C. Barrett's epithelium was isolated by gently peeling it away from the stroma with microdissection needles under a dissecting microscope [41]. The 13 frozen blood samples were processed the same way as the biopsies, except for the epithelial isolation step. DNA was extracted using Puregene DNA Isolation Kit as recommended by the manufacturer (Gentra Systems, Inc. Minneapolis, MN). Samples were quantitated using the Picogreen method (Quant-iT dsDNA Assay, Invitrogen). A total of 200 ng of DNA at 50 ng/ul concentration was analyzed using Illumina Omni-Quad 1M SNP arrays according to manufacturer's protocol. All samples were evaluated at the Fred Hutchinson Cancer Research Center Genomics Core Laboratory.

### GenomeStudio processing

All raw intensity files were loaded in Illumina's GenomeStudio v3, normalized and clustered using the SNP manifest and cluster files for build37 of the human genome. In all our analyses we used the total signal intensity R for each SNP, which is the sum of the normalized X (“A” allele, Cy5 red) and Y (“B” allele, Cy3 green) intensities. We also used the B allele frequency (BAF), which is a modified version of the allelic intensity ratio theta (θ = 2/p*arctan(Y/X)), to reduce SNP-to-SNP variation in theta using the canonical clusters.

### GLAD segmentation

Each individual's BE DNA samples were paired to the individual's control sample (DNA from blood from the same individual), which always appeared normal, i.e. lacking any chromosomal alterations (none of the control samples had any split in BAF over the entire genome, Figure S11). For each individual, we first excluded the 0.2% of SNPs with the lowest R values in the control sample, to remove SNP probes that either perform poorly or fall within germline copy number variant (CNV) regions. We corrected for dye bias (higher fluorescence of the B allele, Cy3 green) by re-centering the BAF of heterozygous and homozygous SNPs of all samples from observing that the median BAF of heterozygous SNPs was ∼0.53, instead of 0.5. Then, for each individual, we identified the set of heterozygous SNPs; i.e., SNPs having a BAF in control sample between 0.33 and 0.66. Finally, we separated the data into three signal profiles: log_2_ (R of BE sample/R of control sample) for heterozygous SNPs only, log_2_ (R of BE sample/R of reference) for homozygous SNPs only, and reflected and scaled BAF of BE sample, (mBAF = abs (BAF of BE sample – 0.5)*2) for heterozygous (informative for LOH) SNPs only. We performed separate wavelet-based segmentation on these three signal profiles using the HaarSeg algorithm [42] from the GLAD [43] package (using parameters haarStart = 3, haarEnd = 9, fdrQ = 0.0001, onlySmoothing = T).

### SGA detection

For each individual, we combined all break points of the segmented three signal profiles from each biopsy to create the set of all the observed breakpoints. We defined the events as the segments between each pair of consecutive breakpoints in this set. Thus, events had to have the same exact breakpoints in order to be considered the same event. For every new segment, we used thresholds to call allelic imbalance based on the smoothed mBAF profile, and to call single or double copy gain or loss, based on the homozygous and heterozygous log_2_R profiles. Thus every new segment meeting the thresholds received one of eight molecular state calls: AB (normal), AA (copy neutral LOH), A (single copy loss), 0 (double copy loss), AAB (single copy gain), AAA (LOH plus subsequent single copy gain), AAAA (LOH plus subsequent double copy gain), AABB (double copy gain). In summary, Table S3 shows all calling thresholds used and Dataset S1 shows raw data segmentation and SGA calls for all 161 biopsies of individuals a–m.

The GLAD segmentation detects break points of SGA for each sample individually. For each individual, we ran a segment merging procedure that merged two adjacent, neighboring segments if they had the same molecular state call across all samples of that individual. Thus, the number of segments per individual can vary. IMPUTE2 [44] and a reference dataset of 566 CEU haplotypes, part of the 1000 Genomes Project [45], was used to phase each individual's blood control sample. Having haplotype assignments for the A and B alleles of every SNP, we developed an algorithm to assign a haplotype state for every segment of allelic imbalance. This results in conversion of AA, A, AAB, AAA, AAAA calls to BB, B, BBA, BBB, BBBB calls for segments having lost or gained the opposite allele. For simplicity of all subsequent analyses, all segments having AB molecular states were assigned an “absence of SGA” call, and all segments having other molecular states were assigned a “presence of SGA” call. The final results are individual-specific phylogenetic matrices having samples as taxa, chromosomal segments as characters, and binary molecular states (SGA absence/presence, or 0/1) as character states.

### Mixtures of clones and technical detection reproducibility

We found evidence of a mixture of clones in 24 of the 161 biopsies (15%, Table S1), based on differential split in BAF on at least two locations within the same chromosome or on at least two locations in two distinct chromosomes. We have done technical replicates comparing two independent blood samples for individuals m and f (Table S5). All four blood DNA samples were assayed with the Illumina OmniQuad platform on different dates and our results show high concordance between blood samples within individuals (Pearson r>0.99 for B allele frequency and r>0.94 for intensity; Figure S11). We detected only 6 and 18 SGA events in blood samples of individuals f and m, respectively, when using the same SGA detection pipeline and SGA events calling thresholds (Table S6). Overall, the SGA calls are based on many SNPs and are robust to technical noise in single SNPs.

### Phylogenetic analyses

To measure mutation rate change associated with NSAID use, we used a two epoch model in BEAST [31], where the transition time between the first and second sampling periods is the time of change in NSAID use. We ran BEAST for 10 million Bayesian MCMC iterations that sample the space of genealogies and population parameters to obtain posterior distributions for model parameters that best fit the data. We used uniform prior distributions for SGA rate with lower and upper bounds of 10^−5^ and 10^4^ SGAs per biopsy genome per year, respectively, for the duration of any of the on-NSAID and off-NSAID periods and estimated SGA rate separately for the first and second sampling periods, where each SGA rate adheres to the molecular clock hypothesis (SGA occur at constant rate for all evolving lineages) for the period duration. We added a 0/1 mutation model in BEAST for the SGA absence/presence character states (Text S1: Equations S3–4) and this model assumed that SGAs do not revert to the normal type, i.e., 1→0 transition is impossible. An SGA character was defined by the breakpoints, which had to occur at the exact same SNPs between samples to be considered the same character. We also modified BEAST's likelihood calculation algorithm to consider a last universal common ancestor (LUCA) that has an unaltered genomic state (zeros for all sites), and that connects to the most recent common ancestor (MRCA), at the root of the tree, creating an extra LUCA-MRCA branch. Thus, the final likelihood of the tree is the product of the likelihood of the tree at the root, calculated with Felsenstein's pruning algorithm [46], multiplied by the probability of the LUCA-MRCA branch length. We used crypt density results (Table S4) and a logistic growth model (Text S1) to estimate the age of the root of the tree and to constrain the internal node coalescence times in the two epoch (off- on- NSAID) BEAST analysis.

Maximum parsimony trees were estimated using Wagner parsimony with delayed transformation (DELTRAN) on the individual-specific phylogenetic matrix with 0/1 SGA states using the PAUP program [47]. For PAUP analyses, we also used a character transition matrix that assumes infinite cost for 1→0 transitions, i.e. SGAs do not revert to normal type. For individuals with off-on NSAID regimens (a–k) all lineages (i.e. phylogeny branches) having *any* off-NSAID descendant lineages (i.e. leading to off-NSAID biopsies) were considered to have evolved during off-NSAID usage period. Similarly, for individuals l and m, all lineages having *any* on-NSAID descendant lineages were considered to have evolved during on-NSAID usage period. In all individuals (a–m), lineages having descendant lineages leading to all on-NSAID or all off-NSAID biopsies were considered to have evolved during on-NSAID and off-NSAID usage periods, respectively.

### Non-phylogenetic analyses of SGA data

We calculated genetic divergence between biopsies separated by time and space (Text S2, Figures S6, S7). We also performed additional analyses of SGA lesions new appearances and regressions during on- and off- NSAID periods that do not use phylogenies (Text S3, Figures S12, S13 and Text S4, Figure S14). The spatial distribution of the number of SGA events detected in biopsies is summarized with a violin plot for each individual (Figure S15).

All code used for the above analyses is licensed under the GNU Lesser GPL (http://www.gnu.org/licenses/lgpl.html) and made publicly available at https://github.com/rkostadi/BEClonalEvolutionNSAID.

### Ethics statement

The Seattle Barrett's Esophagus Study has been approved by the University of Washington Human Subjects Review Committee since 1983 with reciprocity from the Fred Hutchinson Cancer Research Center Institutional Review Board since 1994. . Informed consent had been collected from the research participants. Seventy seven percent of the participants are male and 23% are female. The ethnic distribution of the study cohort is 85% White, 0.4% Black, 1.3% Hispanic, 0.9% Asian, and 0.9% Native American and 11% unknown. This gender and ethnic distribution reflects the known demographics of Barrett's esophagus and esophageal adenocarcinoma in the United States, which is predominantly a disease of white, middle aged and elderly men.

## Supporting Information

Dataset S1 **Raw data segmentation and SGA calls for all 161 biopsies of individuals a–m.** Every page shows an individual biopsy and has 5 panels (top to bottom): first panel, raw Log_2_R ratio between biopsy and leukocyte control, where gray SNPs are homozygous SNPs and black SNPs are heterozygous SNPs; second panel, GLAD segmentation of homozygous SNPs (blue line) and heterozygous SNPs (red line); third panel, raw mBAF (reflected and scaled B Allele Frequency of the BE sample) where homozygous SNPs are shown in gray, and heterozygous SNPs are shown in black; fourth panel, GLAD segmentation of the mBAF data of heterozygous SNPs, which are informative for allelic imbalance; fifth panel, final SGA calls for chromosomal regions: GN (copy gain, green), CNLOH (copy neutral LOH, orange), SD (single deletion, or single copy number loss, red), HD (homozygous deletion, or double copy number loss, black). Please note, this file is 23.1 Mb.
(PDF)

Figure S1 **Number of SGA events in every biopsy over time for each individual show that the genomes of biopsies maintain equilibrium in the number of SGA events, with few exceptions (note biopsies with high variation in the number of SGA events in individuals b, f, and j).**
(TIF)

Figure S2 **The total amount of SGA (in Mb) in every biopsy over time for each individual show that the genomes of biopsies maintain equilibrium in the total amount of SGA, with few exceptions (note biopsies with high total SGA in individuals b, d, f, and j).**
(TIF)

Figure S3 **Linear chromosome plot of detected somatic genomic abnormalities (SGAs) in biopsies from the baseline endoscopy (top panel), the first sampling period (off-NSAIDs for a–k and on-NSAIDs for l, m; middle panel), and the second sampling period (on-NSAIDs for a–k and off-NSAIDs for l, m; bottom panel).** This plot shows the genomic location and size of newly detected SGAs during off-NSAID (red) and on-NSAID (green) periods that are summarized in Figure 3A. Black bars represent SGAs observed in a prior sampling period or at baseline; top panel – black represents SGAs detected in any biopsy from the baseline endoscopy; middle panel – black represents SGAs detected in any biopsy from baseline endoscopy that is also detected in at least one biopsy in the first sampling period; bottom panel – black represents SGAs detected in any biopsy from baseline or first sampling period, or both, that is also detected in at least one biopsy in the second sampling period. In the middle panel, red and green bars represent newly acquired SGAs that are detected in at least one biopsy in the first sampling period, but not detected at baseline. In the bottom panel, red and green bars represent newly acquired SGAs that are detected in at least one biopsy in the second sampling period, but not detected in any biopsy from baseline or first sampling period.
(TIF)

Figure S4 **Circos plots of individuals a, c, d, e, g, h, i, k, and m.** Each ring represents whole-genome SGA data from a different biopsy. Thin black line rings separate endoscopies (time points), white background shows time periods off-NSAIDs and gray background shows time periods on-NSAIDs. Within the rings, black segments designate homozygous deletion, red single copy loss, orange copy-neutral LOH, and green shows copy gain.
(TIF)

Figure S5 **Linear chromosome plot of SGAs that are common across all biopsies within an individual.** These are lesions that were present by the time of the first endoscopy and so may have been established with the hypothesized initial expansion of Barrett's epithelium in competition with squamous epithelium.
(TIF)

Figure S6 **Genetic divergence, estimated as average pairwise SGA-based Hamming distance, between biopsies over time and space (y-axis).** For each individual (individuals a–m, rows), column 1 shows genetic divergences among biopsies within time points over individual follow-up time (the x-axis represents follow-up time); column 2 shows genetic divergences only among biopsies that are within ±1 cm of each other regardless of the time point of sampling (x-axis represents temporal distance in years); and column 3 shows genetic divergences only among biopsies that are within the same time point (x-axis represents spatial distance between pairs of biopsies in cm).
(PDF)

Figure S7 **Genetic divergence, estimated as average pairwise proportion of differentially altered genome, between biopsies over time and space (y-axis).** The three columns of plots are identical to those in Figure S6; only the y-axis has changed.
(PDF)

Figure S8 **Estimated trees by BEAST for individuals a–m.** Branch lengths are scaled according to time, the tips of the phylogeny are biopsies aligned on the x-axis according to their sampling time, and all internal nodes are estimated coalescence times assuming a logistic population growth model (see Methods). Dashed gray line represents the time point of change in NSAID use. All these trees show long-term co-existence of clones and only one case of a clonal expansion taking over the Barrett's segment (individual h).
(TIF)

Figure S9 **Estimated trees by PAUP trees where branch lengths represent estimated number of SGA events for individuals a, c, d, e, g, h, i, k and m.** The topologies of these trees suggest progressive accumulation of SGAs.
(TIF)

Figure S10 **Estimated trees by PAUP trees where branch lengths represent the total amount of SGA (Mb) of the estimated 0→1 SGA events from Figure S9 for individuals a, c, d, e, g, h, i, k and m.**
(TIF)

Figure S11 **Log_2_ R ratio between replicate blood samples from the same individual (m and f, panels A and B, respectively) showed no detectable large-scale alterations and high Pearson correlation of SNP probe total intensity R (Pearson r = 0.9481 and r = 0.9407, for individual m and f, respectively).** B allele frequency showed high correlation within replicate blood samples from the same individual (Pearson r = 0.995 and r = 0.9962, for individuals m and f, panels D and E, respectively). Log_2_ R ratio between two blood samples from two different individuals (m and f) showed no detectable large-scale alterations and decreased Pearson correlation between SNP probes total intensity R (Pearson r = 0.8739, panel C). B allele frequency between blood samples from different individuals showed that SNP probes can fall into all nine possible genotype categories reflecting the difference between germline genotype of individuals (Pearson r = 0.6755, panel F). Supplementary Table S5 describes blood sample details.
(TIF)

Figure S12 **An individual-specific SGA matrix (panel A) is used as example to show the conceptual difference between using summary statistics and using phylogenies to compute the rate of acquisition of new SGA.** The individual-specific SGA phylogenetic matrix shows the SGA molecular states absence/presence (0/1) for 4 biopsies (numbered 1–4, rows) and for 4 SGA characters/events (lettered a–d, columns). According to an independent lineages model, each biopsy samples a clone that is evolving independently. Here de novo SGA are counted simply by counting SGA events that are never observed in previous time periods (panel B). According to a clonal evolution model, acquisition of SGA events is assumed to occur strictly on a phylogenetic tree that maximizes the number of shared SGA events (for example, events a and b are observed in biopsies 1 and 2, therefore they most likely occurred on a branch prior to their most recent common ancestor; similarly event d most likely occurred once on the branch leading to the most recent common ancestor of biopsies 3 and 4, and minimizes the number of homoplasies (for example, event c is observed in biopsies 2 and 3, therefore it appears twice on branches of the phylogeny most likely due to convergent evolution) (panel C). There is uncertainty in estimating the dates of most recent common ancestors (internal nodes in the phylogeny) therefore we used BEAST with a minimal set of assumptions to estimate the rates of acquisition of SGA events during off-NSAID and on-NSAID time periods. We show how the two models may differ when estimating the SGA rate during two time periods (panel D). We have used both models when estimating SGA rate off- and on-NSAID (Figures S13 and 6), and found consistent results that NSAIDs reduce acquisition of SGAs.
(TIF)

Figure S13 **NSAIDs influence the number of SGA events entering and leaving detection.** (Panel A) Number of new SGA events detected during on- and off-NSAIDs intervals. (Panel B) Number of previously observed SGA events not observed at the final biopsy (Panels A, B). We binned newly appearing or regressed, but previously sampled, SGA according to lesion size (0 bp–100 Mb, x-axis), but detected no apparent effect of NSAID use on selection for or against clones with lesions of a specific size category; rather, NSAID use affected clones with all size categories of SGAs equally. (Wilcoxon rank-sum test, * = one-sided p-value <0.05, box-whisker plots show the medians and interquartile ranges, with the default whisker ranges).
(TIF)

Figure S14 **Dropout SGA events analysis.** For each individual, the probability of a lesion dropping out of detection 1-P was estimated (y-axis). In 7 out of 13 individuals the probability of a lesion dropping out of detection was higher on-NSAIDs.
(TIF)

Figure S15 **Spatial distribution of SGA load within Barrett's segments.** No consistent and striking differences were found between SGA load in the upper level of the Barrett's segment (SCJ, squamocolumnar junction) compared to the lower level (GEJ, gastroesophageal junction). Note that previous analyses of the Seattle Barrett's Esophagus cohort have not found any preferential level of occurrence of adenocarcinoma in relation to SCJ and GEJ boundaries of Barrett's segments.
(TIF)

Table S1 **Number of SGA events and total amount of SGA detected for each of 161 biopsies.** For each biopsy (rows), the following information is provided (columns): individual ID, sex of individual, individual's rounded age at the endoscopy time point of the biopsy, biopsy ID, biopsy age since baseline endoscopy, biopsy level recorded from incisors, gastroesophageal junction (GEJ) level recorded from incisors, squamocolumnar junction (SCJ) level recorded from incisors, years off-NSAID per individual, years on-NSAIDs per individual, NSAID use recorded at biopsy (N-non-user, C-current user, F-former user, NA-not available), date of the SNP array assay per biopsy, total base pair of SGA detected by category per biopsy (CNLOH-copy neutral loss of heterozygosity, SD-single deletion, HD-homozygous (double) deletion, GN-copy number gain, ALL-all categories of SGA combined), total number of SGA events detected by category per biopsy, and evidence that the biopsy is a mixture of > = 2 clones manually scored, all based on the SGA segmentation results shown in Dataset S1.
(XLSX)

Table S2 **Tree shape imbalance statistics for individuals a–k estimated for PAUP and BEAST trees calculated from generating 500 random trees having the same number of taxa under Yule and PDA null models** [**48**] **using the R package “apTreeshape”** [**49**] **.** Significant p-values reject the null hypothesis that the observed tree shape is as balanced as the 500 random tree shapes, where the test statistics are the Colless and Sackin formulas [48] for calculating tree shape imbalance.
(XLSX)

Table S3 **Calling thresholds (lower and upper bounds given in parentheses) used for identifying segments with single copy loss, double copy loss, copy gain, and copy neutral LOH.**
(XLSX)

Table S4 **Crypt density results.** Age is the age of the individual at the first endoscopy. L is the BE segment length measured as the distance in centimeters from the GEJ to the OS anatomical landmarks; A is an estimate of the total area of the BE segment by assuming a circumference of 7.5 cm of the esophagus (estimated from [50]); X is the number of levels that were biopsied and had slides from which various numbers of biopsies were evaluated for counting the number of crypts and the number of branching crypts per slide; K is an estimate of the maximum crypt count in BE segments extrapolated from the maximum number of observed crypts in every scored level; N_t_ is an estimate of the total crypt count in BE segments at baseline endoscopy; I_b_ is the fraction of crypts that appear to be branching in a sample of crypts; T_r_ is the estimated crypt doubling time in days (see Text S1: Equation S1); T_init_ is the estimated time from initiation of the BE segment to baseline endoscopy in years, assuming a logistic growth starting with 1 crypt that grows to a population of N_t_ crypts at baseline, and assuming a carrying capacity of K crypts for the BE segment (see Text S1: Equation S2); BEAST T_init_ was bounded to 1 year earlier than the T_init_ to allow some flexibility in the estimate of the exact initiation date during the BEAST MCMC runs. (*) We did not have crypt count information for individual g, so we estimated T_init_ and BEAST T_init_ by taking the average from individuals c, h, i, and m since they had the same segment length (5 cm) as individual g.
(XLSX)

Table S5 **Technical replicate blood samples information.**
(XLSX)

Table S6 **Results for detected SGA in blood-blood sample (technical replicate) comparisons.**
(XLS)

Text S1 **Additional methods for modeling somatic evolution in the crypt structured Barrett's segment that supplement BEAST phylogenetic analysis.** This includes equations for estimating crypt doubling time and the initiation phase duration, as well as the substitution matrix (transition rate matrix) and continuous time solutions for the probability of transitions for the evolution of SGAs.
(DOC)

Text S2 **Additional methods for calculating genetic divergence between biopsies separated by space and time.**
(DOC)

Text S3 **Alternative non-phylogeny-based methods for analysis of SGA occurrences and regressions during on- and off- NSAID periods.**
(DOC)

Text S4 **Alternative method and analysis of SGA events dropping out of detection (or regressing) during on- and off- NSAID periods.**
(DOC)
