# Supplementary material for: NSAIDs Modulate Clonal Evolution in Barrett's Esophagus
Source: PLoS Genet. 2013 Jun 13;9(6):e1003553. doi: 10.1371/journal.pgen.1003553 (PMC3681672; doi:10.1371/journal.pgen.1003553)
Supplement: Dataset S1 — Raw data segmentation and SGA calls for all 161 biopsies of individuals a–m. Every page shows an individual biopsy and has 5 panels (top to bottom): first panel, raw Log2R ratio between biopsy and leukocyte control, where gray SNPs are homozygous SNPs and black SNPs are heterozygous SNPs; second panel, GLAD segmentation of homozygous SNPs (blue line) and heterozygous SNPs (red line); third panel, raw mBAF (reflected and scaled B Allele Frequency of the BE sample) where homozygous SNPs are shown in gray, and heterozygous SNPs are shown in black; fourth panel, GLAD segmentation of the mBAF data of heterozygous SNPs, which are informative for allelic imbalance; fifth panel, final SGA calls for chromosomal regions: GN (copy gain, green), CNLOH (copy neutral LOH, orange), SD (single deletion, or single copy number loss, red), HD (homozygous deletion, or double copy number loss, black). Please note, this file is 23.1 Mb. (PDF) [file pgen.1003553.s001.pdf]

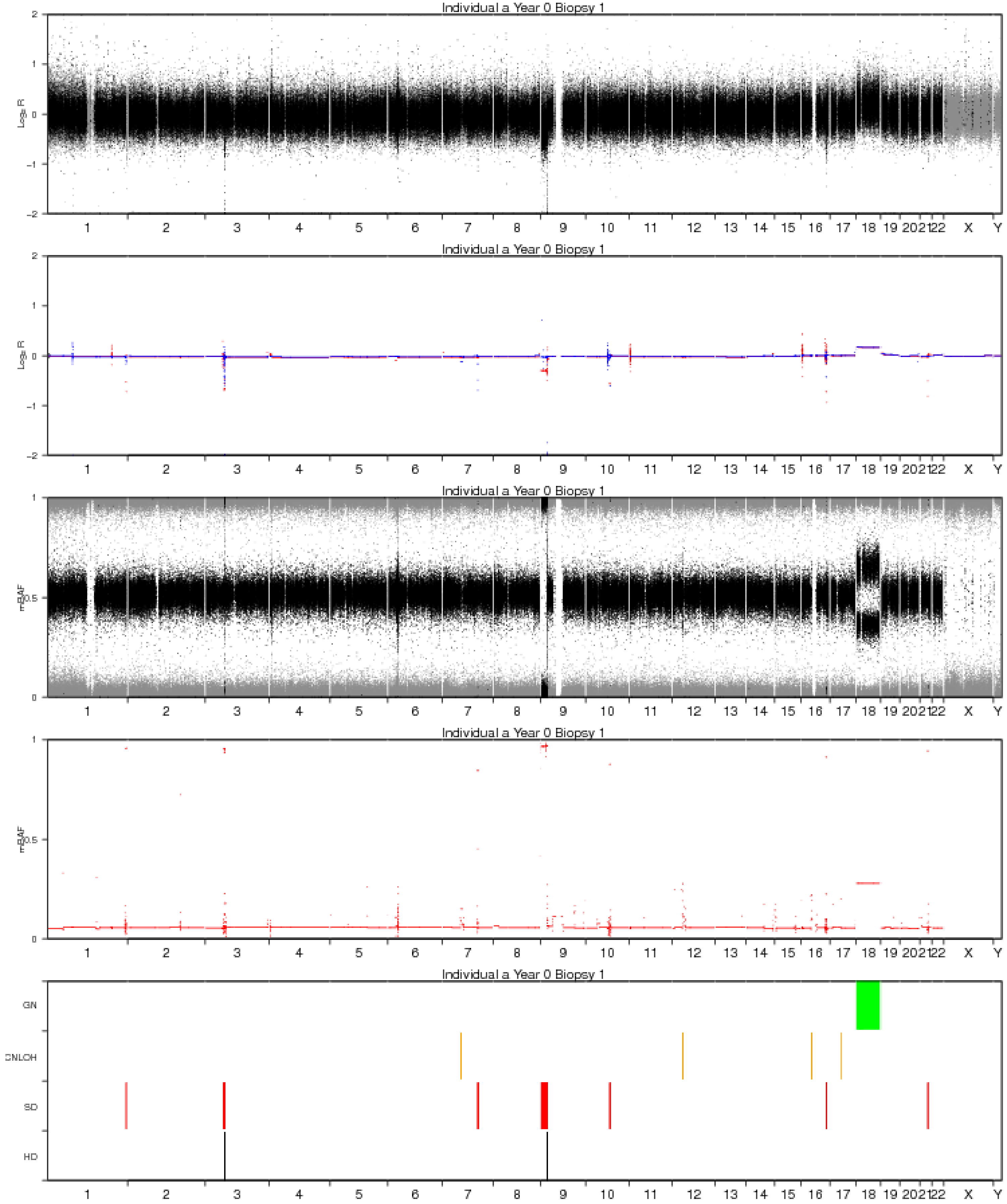
Individual a Year 0 Biopsy 1
Log R
mBAF
GN
CNLOH
SD
HD
1 2 3 4 5 6 7 8 9 10 11 12 13 14 15 16 17 18 19 20 21 22 X Y

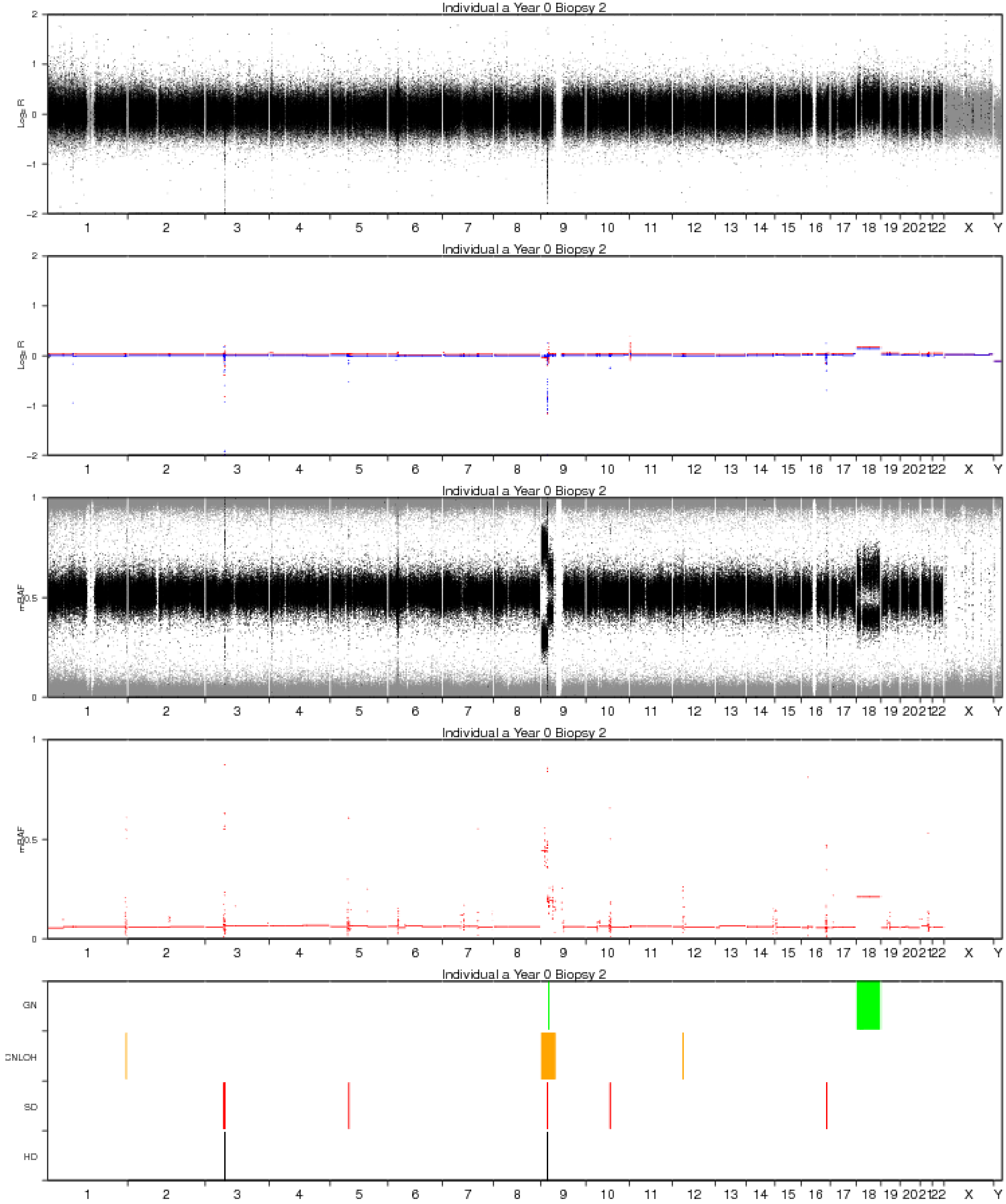
Individual a Year 0 Biopsy 2
Log R
mBAF
GN
CNLOH
SD
HD
1 2 3 4 5 6 7 8 9 10 11 12 13 14 15 16 17 18 19 20 21 22 X Y

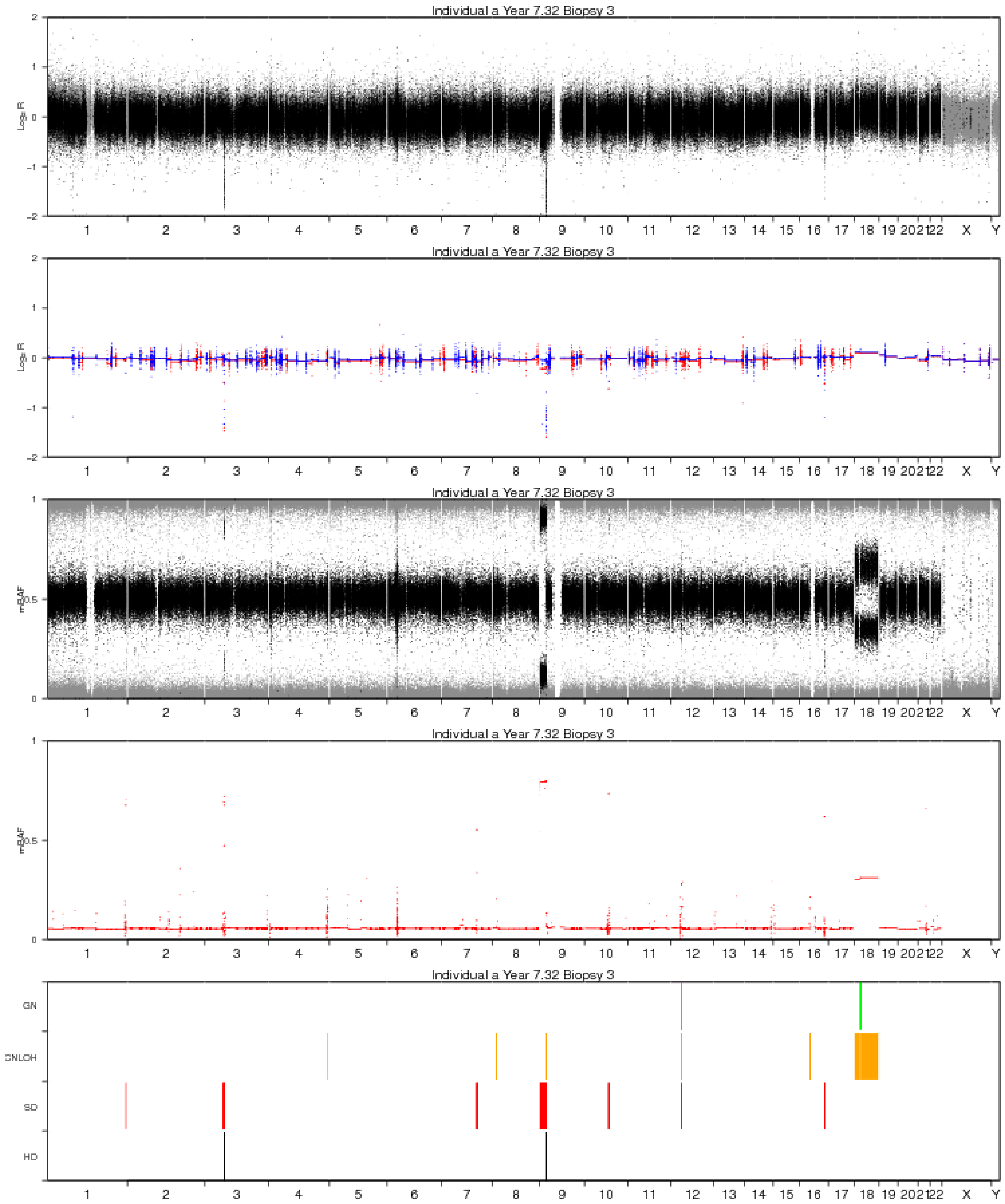

Individual a Year 7.32 Biopsy 3
Log2 R
mBAF
GN
CNLOH
SD
HD
1 2 3 4 5 6 7 8 9 10 11 12 13 14 15 16 17 18 19 20 21 22 X Y

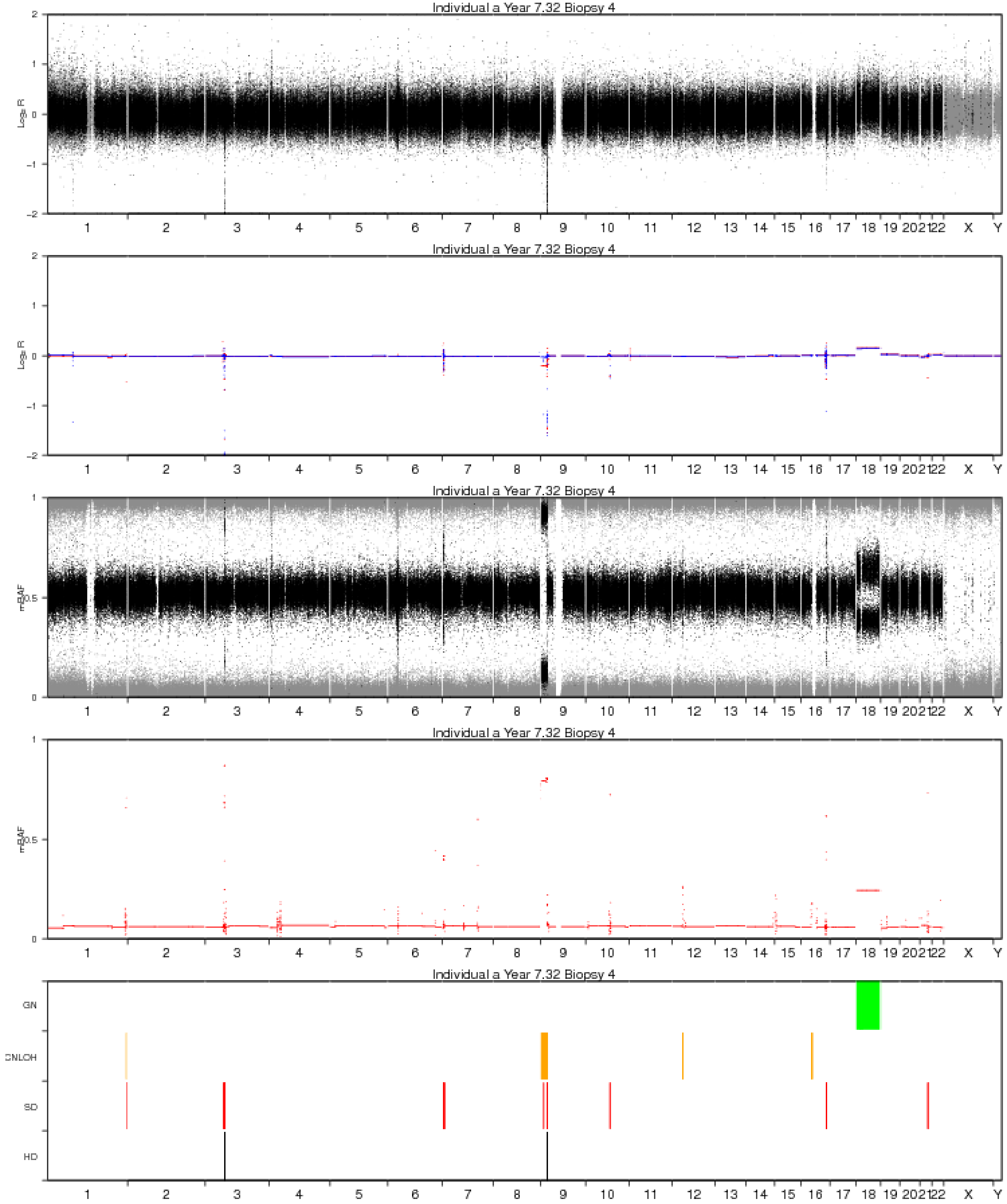
Individual a Year 7.32 Biopsy 4
Log R
2
1
0
−1
−2
1 2 3 4 5 6 7 8 9 10 11 12 13 14 15 16 17 18 19 20 21 22 X Y
Individual a Year 7.32 Biopsy 4
Log R
Individual a Year 7.32 Biopsy 4
mBAF
1
0.5
0
Individual a Year 7.32 Biopsy 4
mBAF
Individual a Year 7.32 Biopsy 4
GN
CNLOH
SD
HD

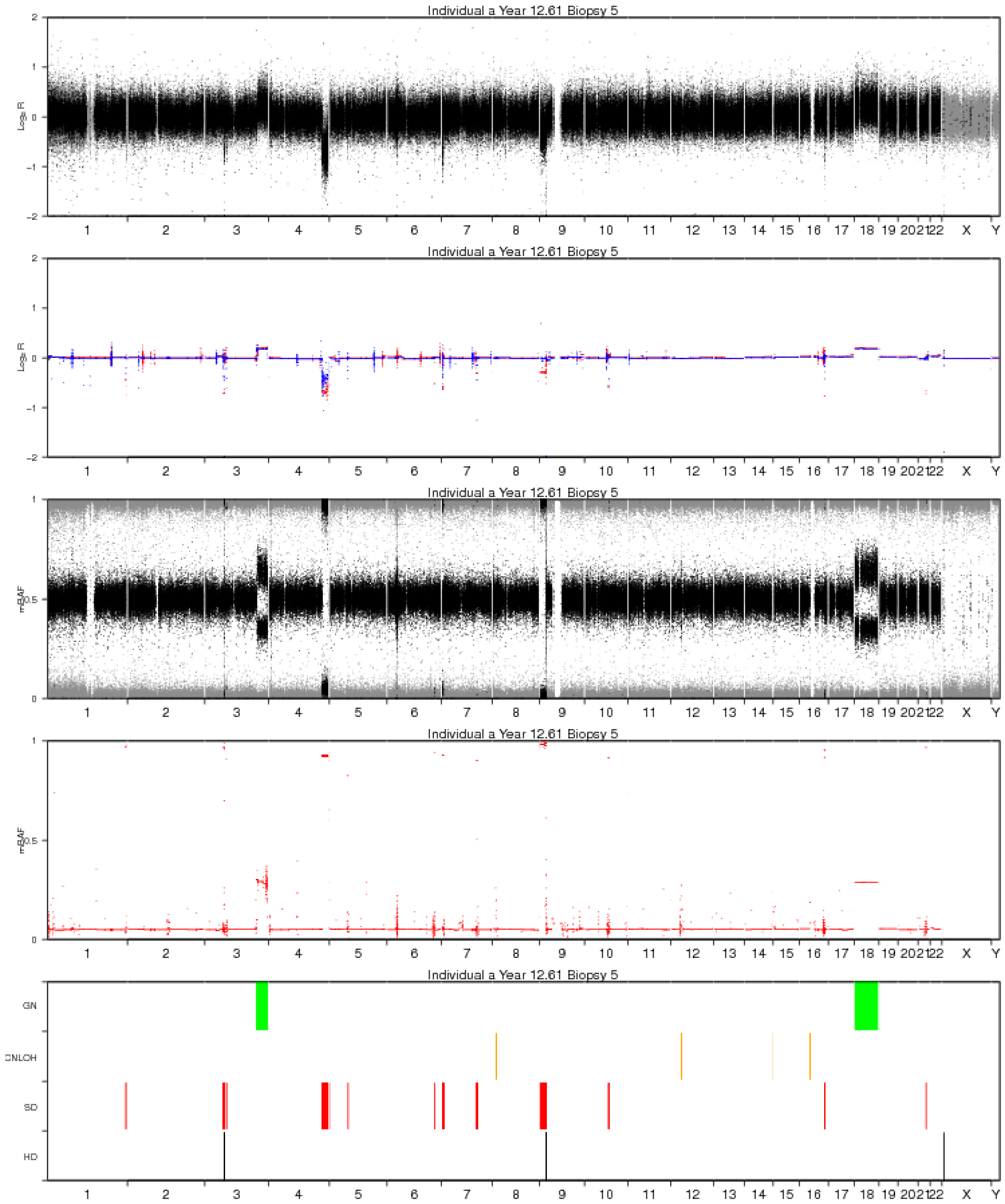
Individual a Year 12.61 Biopsy 5
Log R
2
1
0
−1
−2
1 2 3 4 5 6 7 8 9 10 11 12 13 14 15 16 17 18 19 20 21 22 X Y
Individual a Year 12.61 Biopsy 5
Log R
2
1
0
−1
−2
1 2 3 4 5 6 7 8 9 10 11 12 13 14 15 16 17 18 19 20 21 22 X Y
Individual a Year 12.61 Biopsy 5
mBAF
1
0.5
0
1 2 3 4 5 6 7 8 9 10 11 12 13 14 15 16 17 18 19 20 21 22 X Y
Individual a Year 12.61 Biopsy 5
mBAF
1
0.5
0
1 2 3 4 5 6 7 8 9 10 11 12 13 14 15 16 17 18 19 20 21 22 X Y
Individual a Year 12.61 Biopsy 5
GN
CNLOH
SD
HD
1 2 3 4 5 6 7 8 9 10 11 12 13 14 15 16 17 18 19 20 21 22 X Y

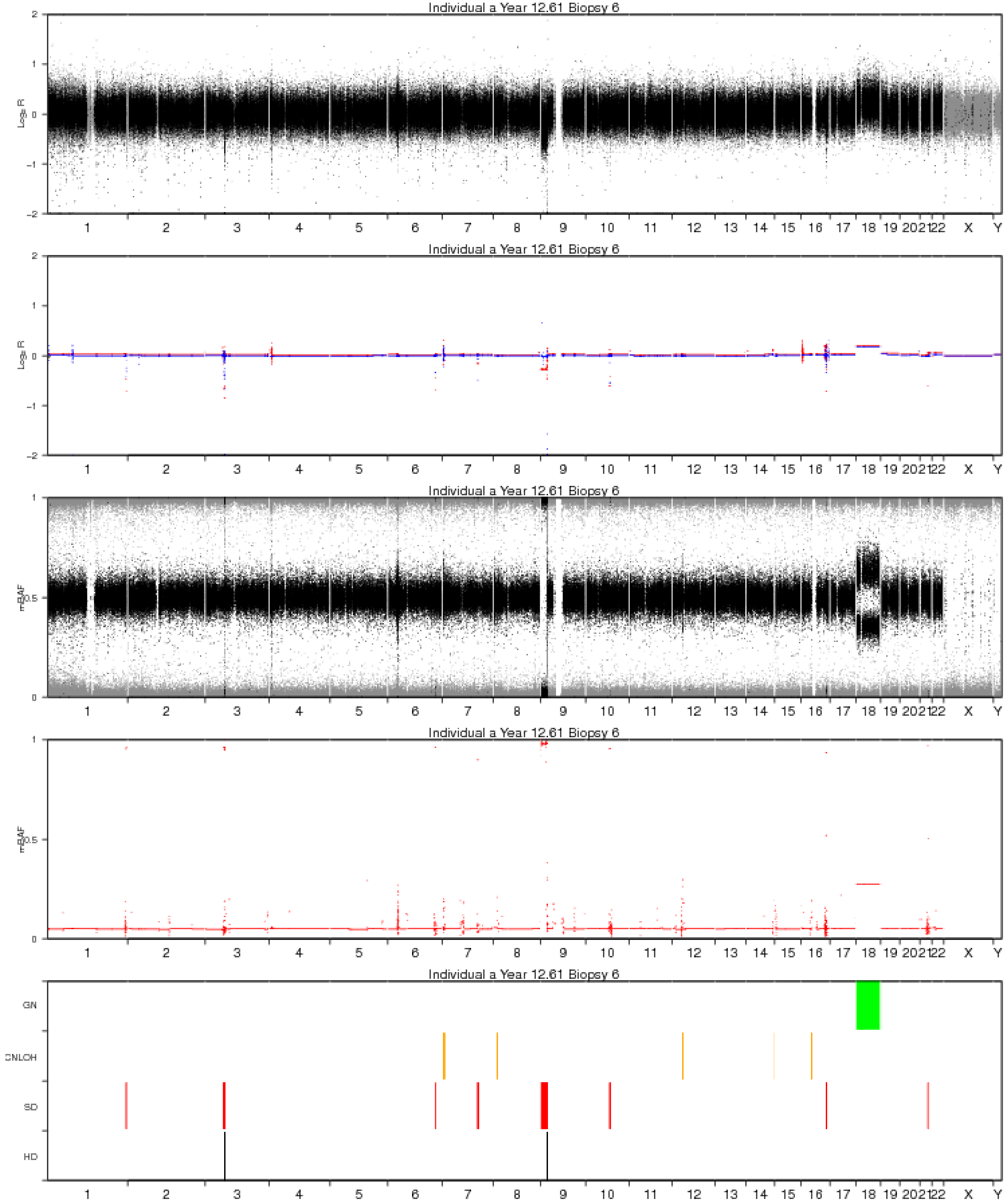
Individual a Year 12.61 Biopsy 6
Log R
mBAF
GN
CNLOH
SD
HD
1 2 3 4 5 6 7 8 9 10 11 12 13 14 15 16 17 18 19 20 21 22 X Y
2
1
0
−1
−2
0.5

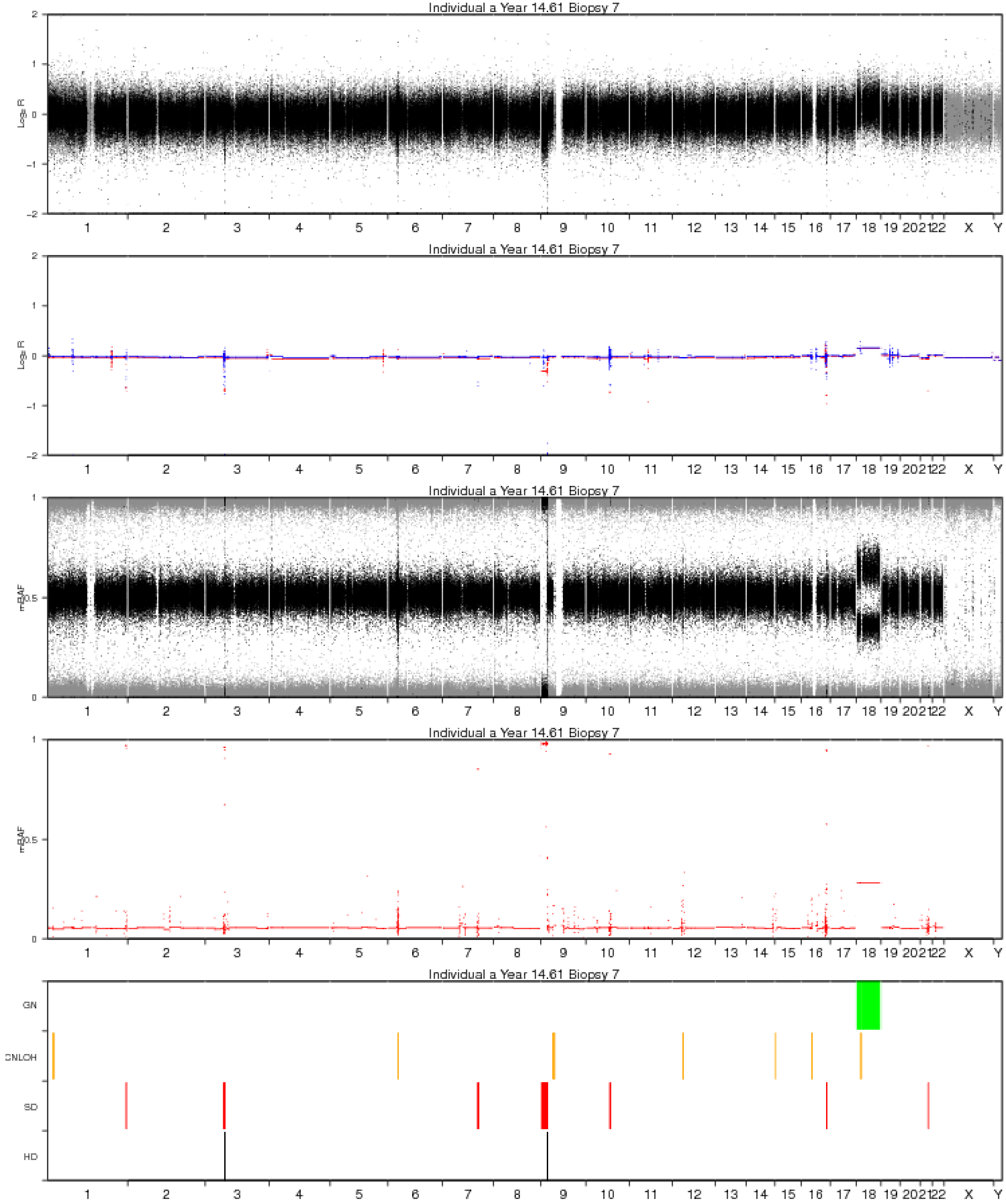

Individual a Year 14.61 Biopsy 7
Log R
mBAF
GN
CNLOH
SD
HD
1 2 3 4 5 6 7 8 9 10 11 12 13 14 15 16 17 18 19 20 21 22 X Y

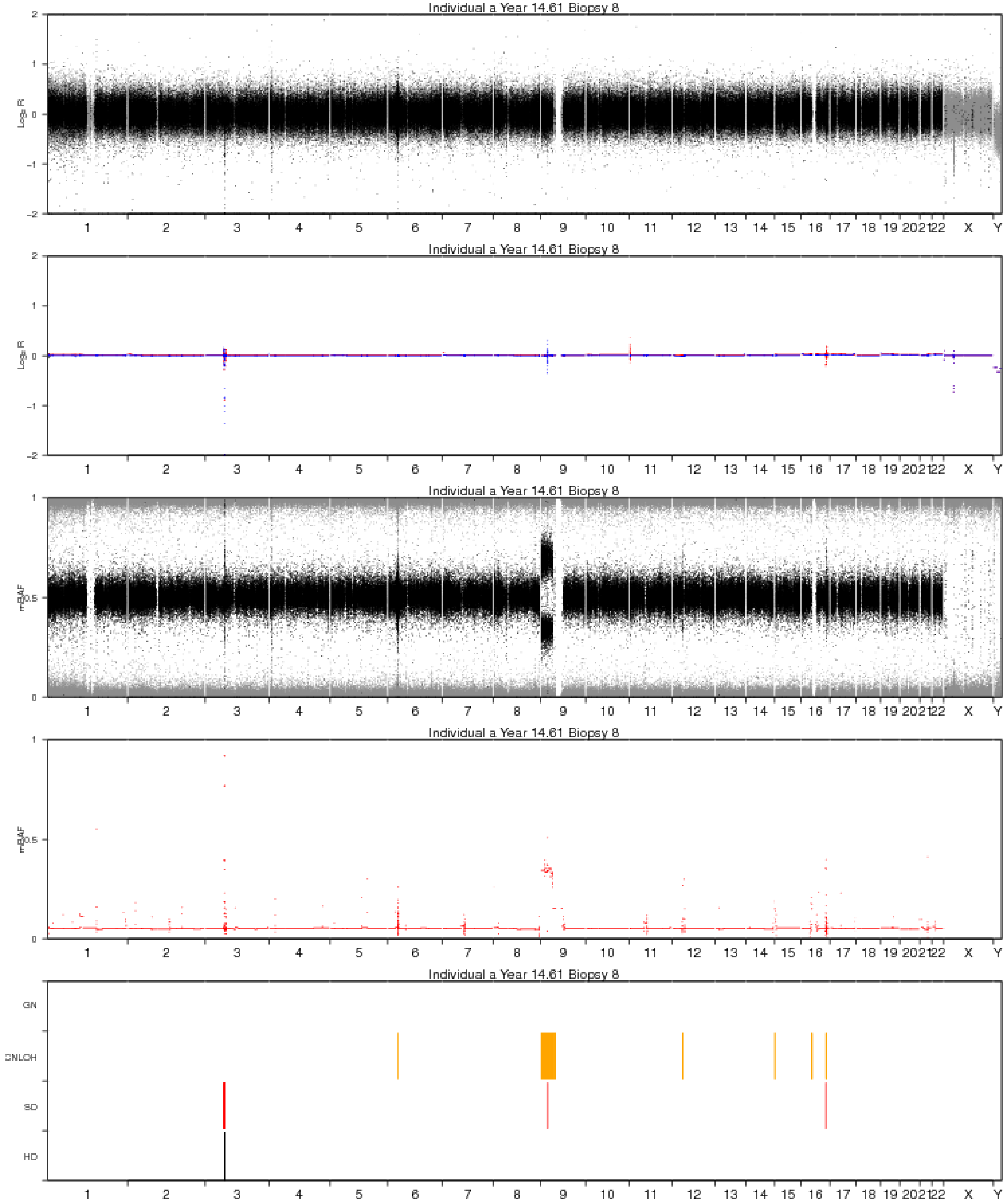
Individual a Year 14.61 Biopsy 8
Log R
mBAF
GN
CNLOH
SD
HD
1 2 3 4 5 6 7 8 9 10 11 12 13 14 15 16 17 18 19 20 21 22 X Y

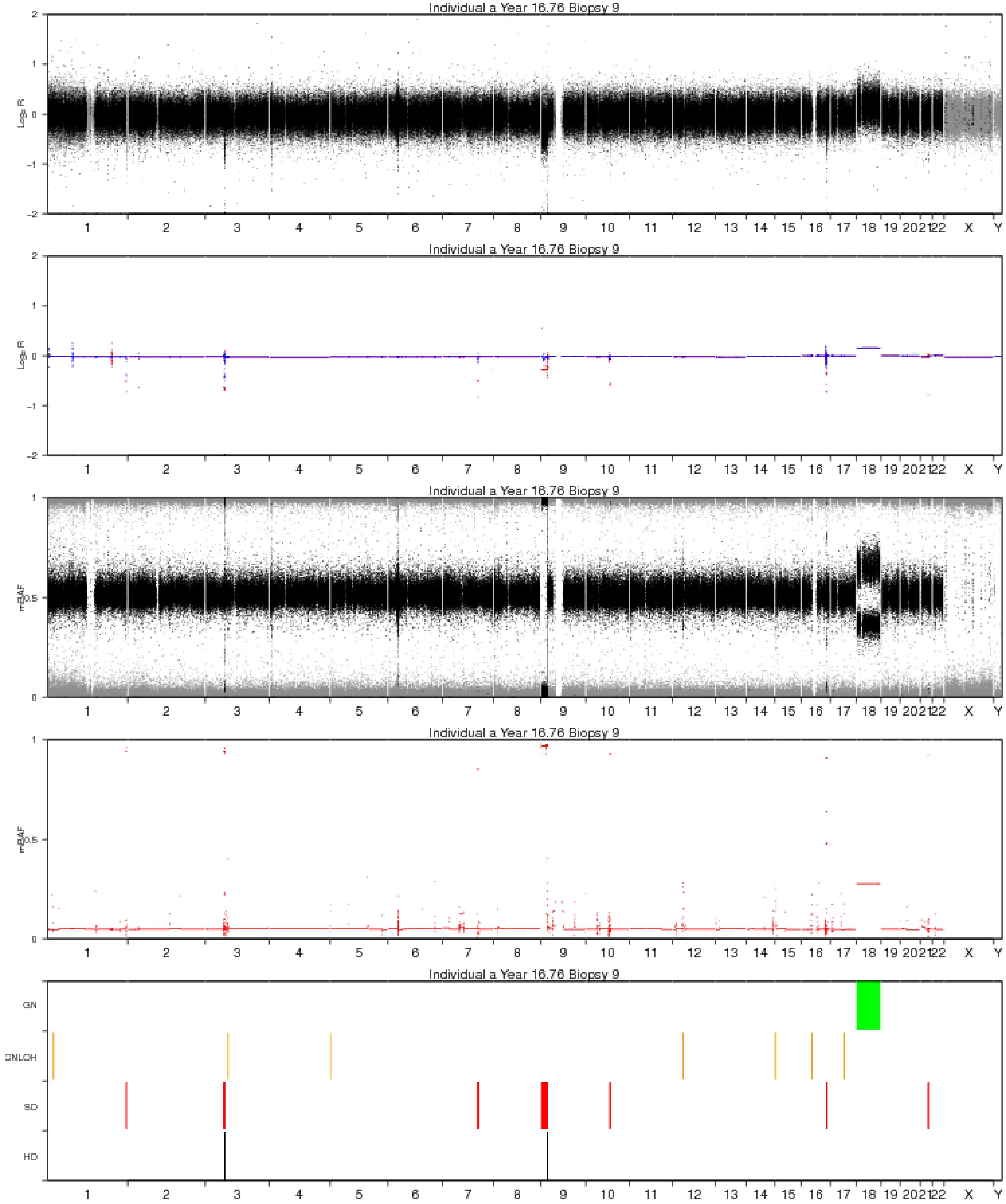
Individual a Year 16.76 Biopsy 9
Log R
mBAF
GN
CNLOH
SD
HD
1 2 3 4 5 6 7 8 9 10 11 12 13 14 15 16 17 18 19 20 21 22 X Y

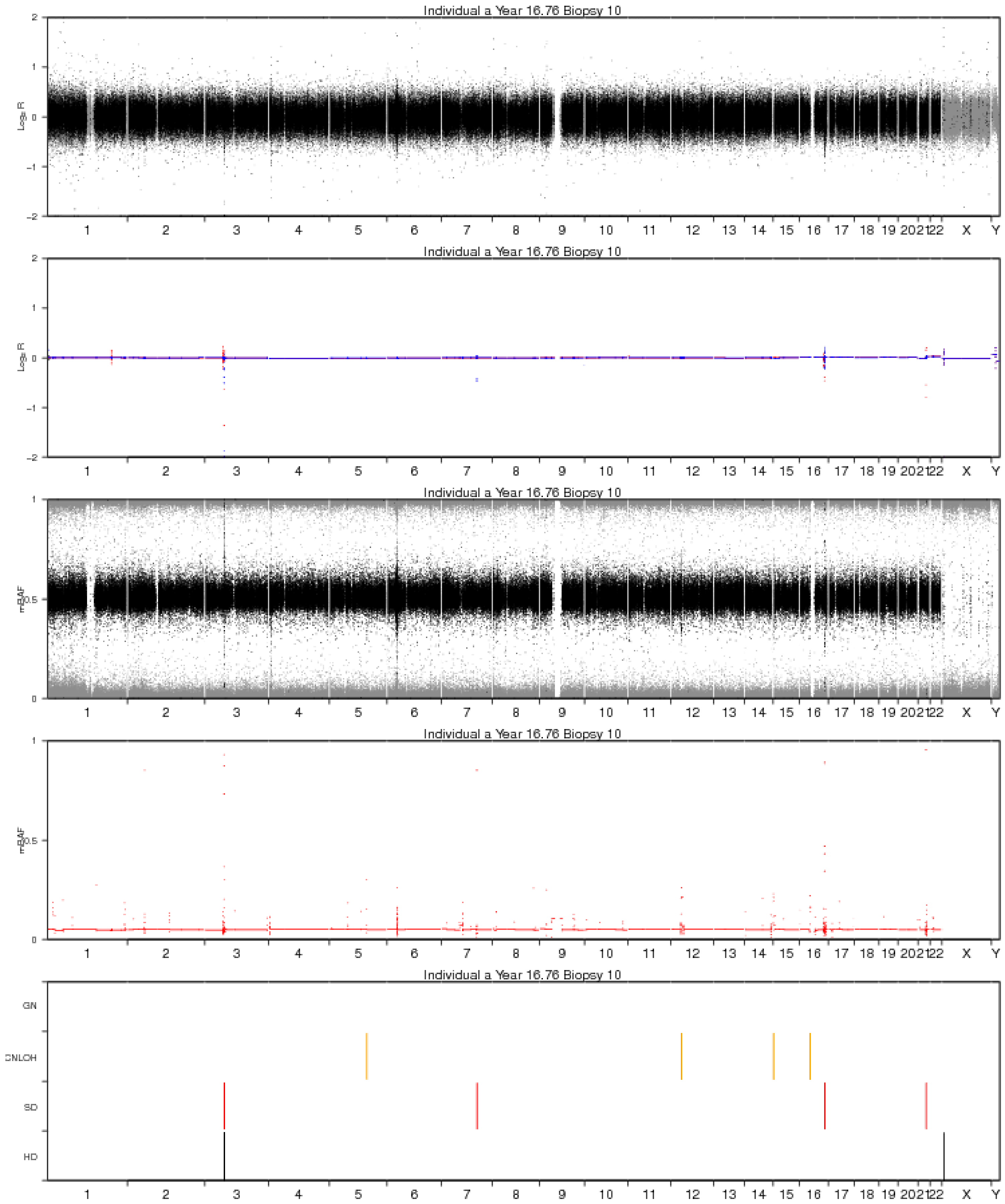
Individual a Year 16.76 Biopsy 10
Log R
mBAF
GN
CNLOH
SD
HD
1 2 3 4 5 6 7 8 9 10 11 12 13 14 15 16 17 18 19 20 21 22 X Y

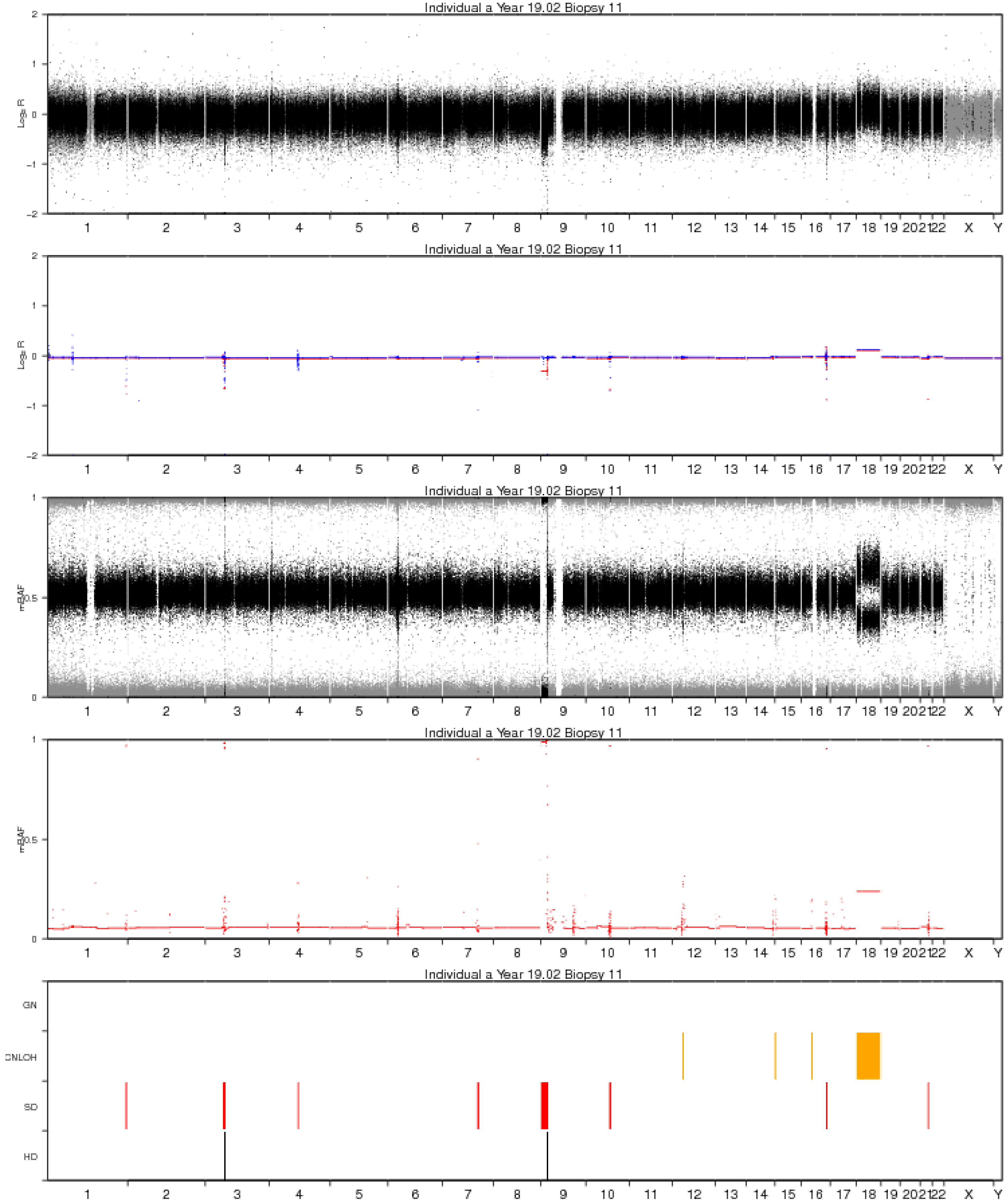
Individual a Year 19.02 Biopsy 11
Log R
mBAF
GN
CNLOH
SD
HD
1 2 3 4 5 6 7 8 9 10 11 12 13 14 15 16 17 18 19 20 21 22 X Y

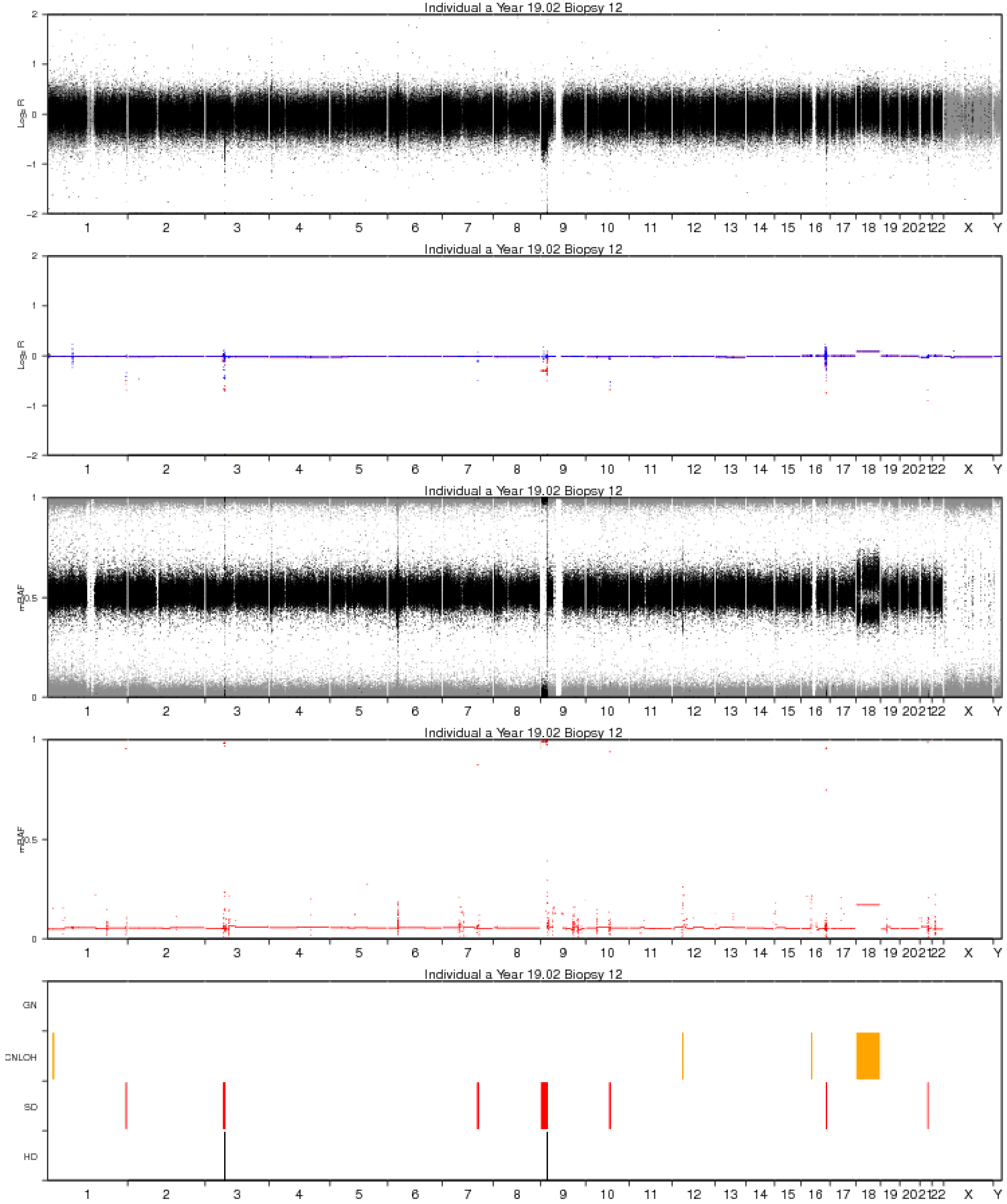
Individual a Year 19.02 Biopsy 12
Log R
mBAF
GN
CNLOH
SD
HD
1
2
3
4
5
6
7
8
9
10
11
12
13
14
15
16
17
18
19
20
21
22
X
Y

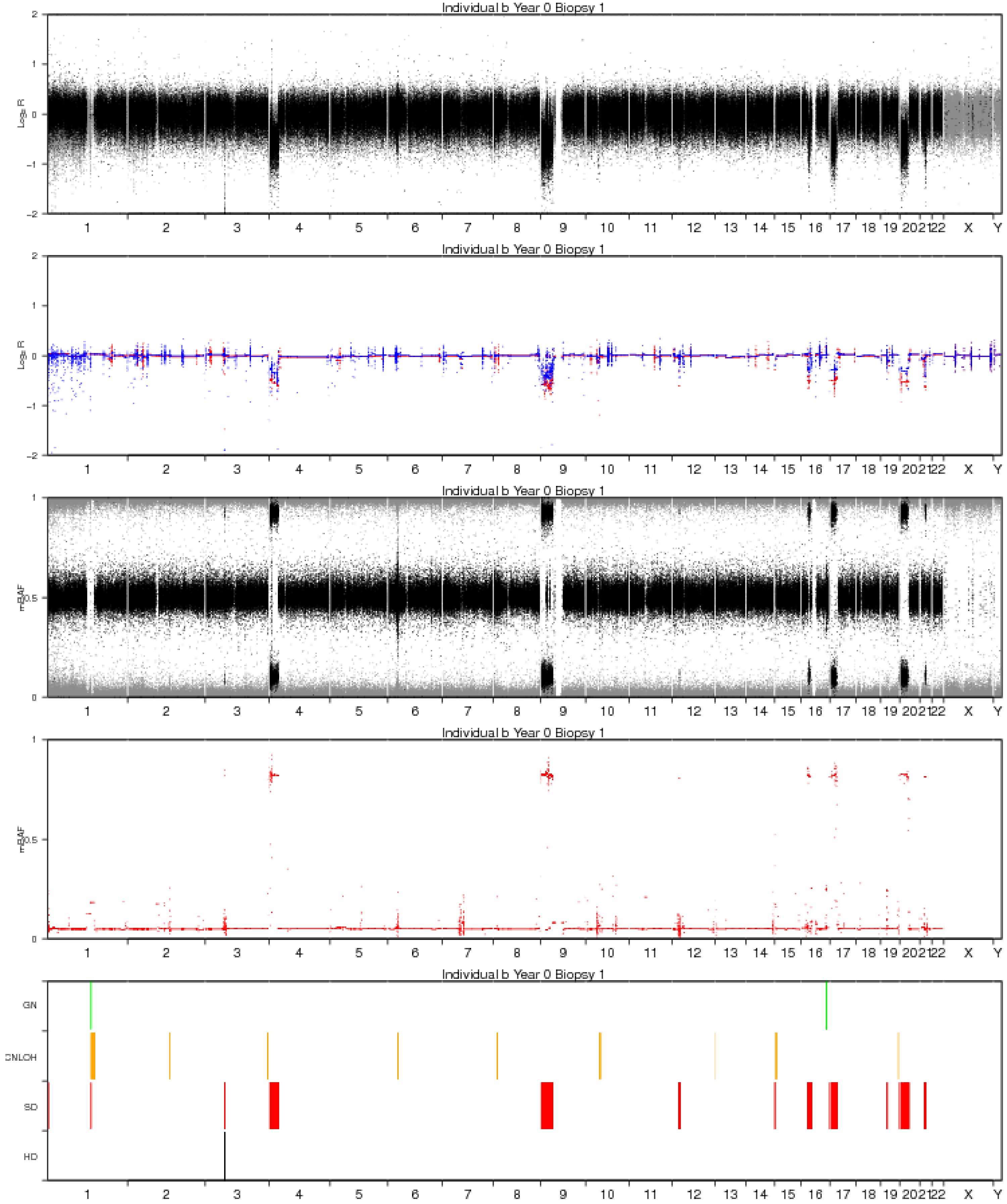
Individual b Year 0 Biopsy 1
Log2 R
mBAF
GN
CNLOH
SD
HD
1 2 3 4 5 6 7 8 9 10 11 12 13 14 15 16 17 18 19 20 21 22 X Y

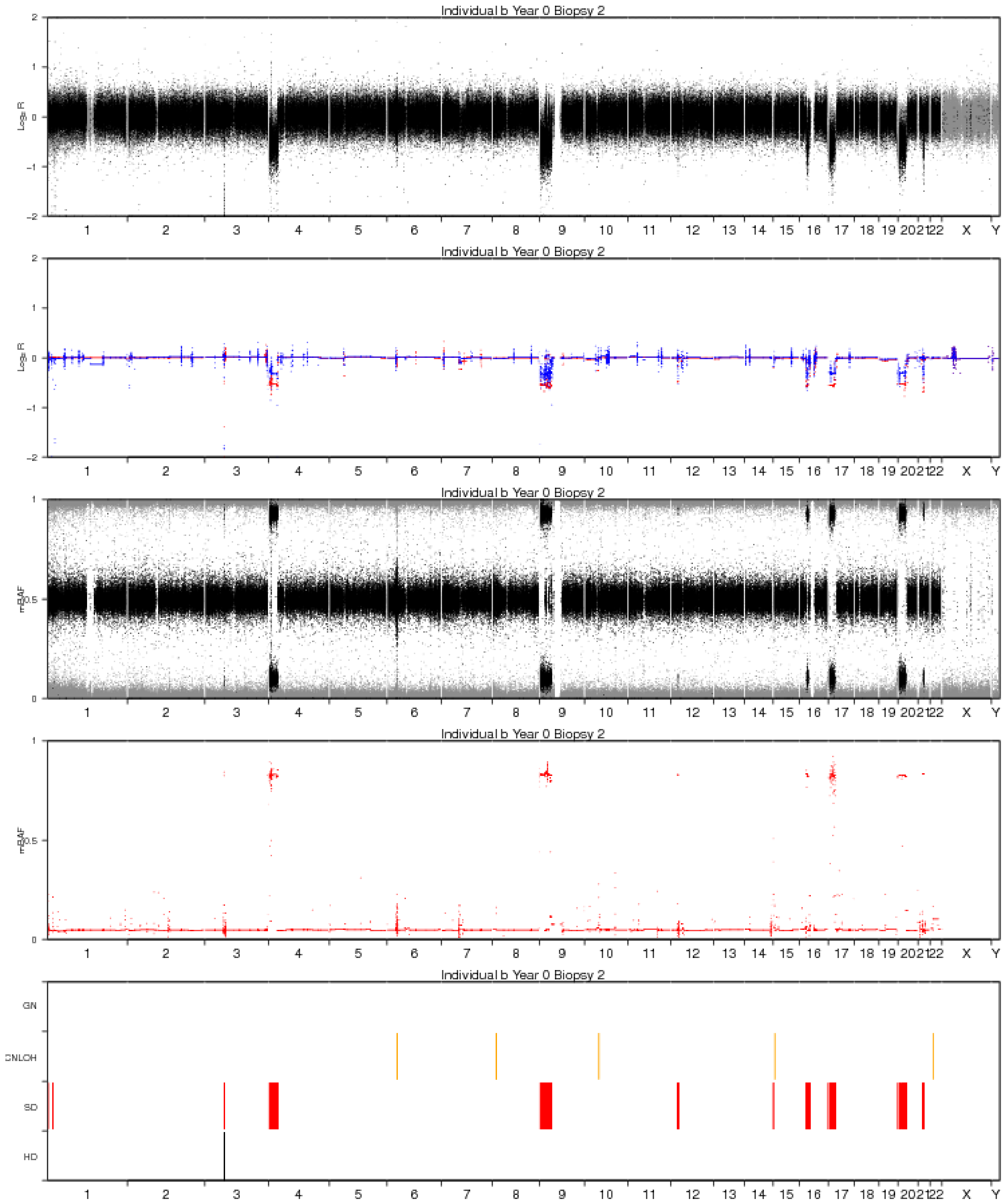
Individual b Year 0 Biopsy 2
Log2 R
2
1
0
−1
−2
1 2 3 4 5 6 7 8 9 10 11 12 13 14 15 16 17 18 19 20 21 22 X Y
Individual b Year 0 Biopsy 2
Log2 R
2
1
0
−1
−2
1 2 3 4 5 6 7 8 9 10 11 12 13 14 15 16 17 18 19 20 21 22 X Y
Individual b Year 0 Biopsy 2
mBAF
1
0.5
0
1 2 3 4 5 6 7 8 9 10 11 12 13 14 15 16 17 18 19 20 21 22 X Y
Individual b Year 0 Biopsy 2
mBAF
1
0.5
0
1 2 3 4 5 6 7 8 9 10 11 12 13 14 15 16 17 18 19 20 21 22 X Y
Individual b Year 0 Biopsy 2
GN
CNLOH
SD
HD
1 2 3 4 5 6 7 8 9 10 11 12 13 14 15 16 17 18 19 20 21 22 X Y

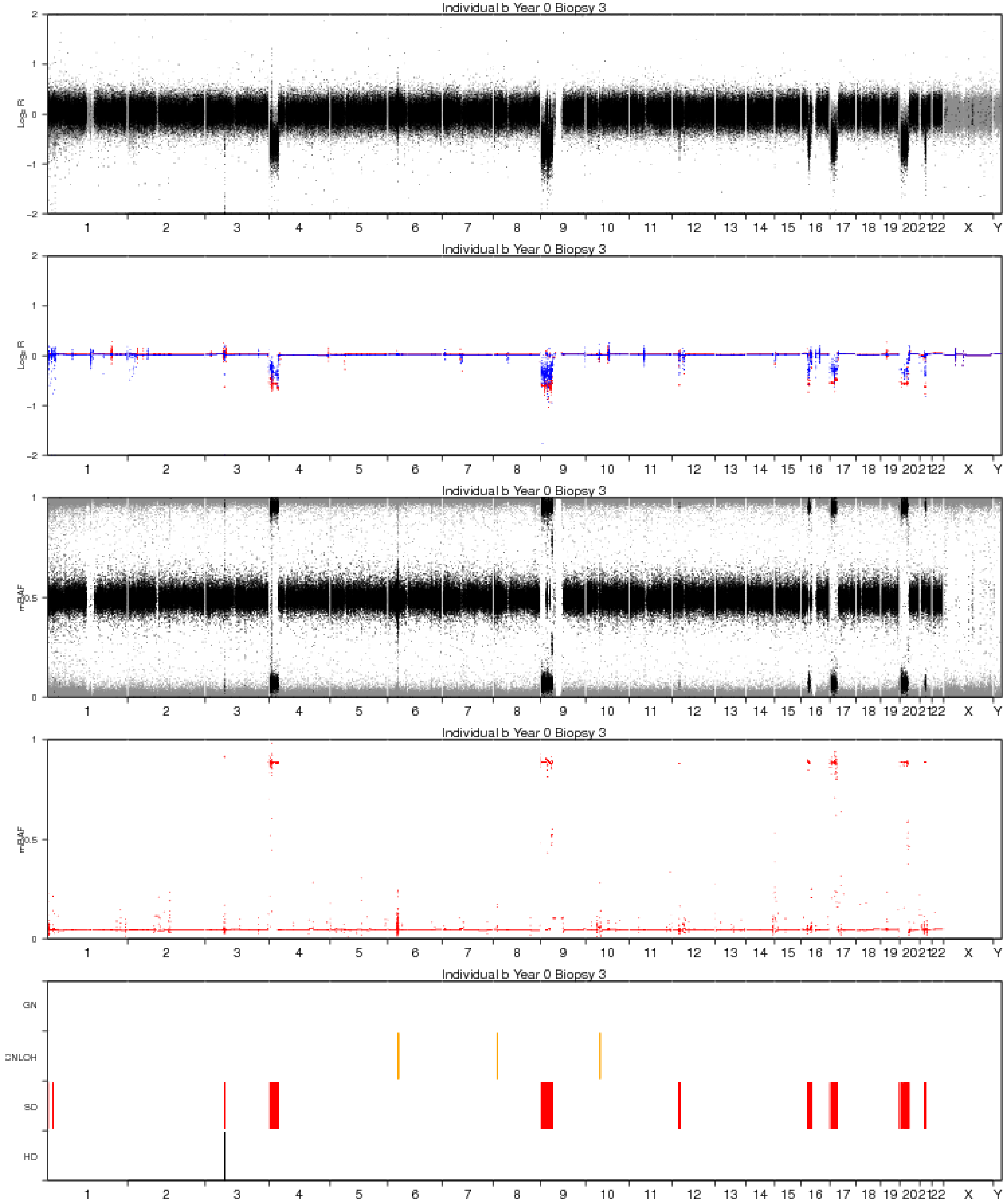
Individual b Year 0 Biopsy 3
Log R
2
1
0
−1
−2
1 2 3 4 5 6 7 8 9 10 11 12 13 14 15 16 17 18 19 20 21 22 X Y
Individual b Year 0 Biopsy 3
Log R
Individual b Year 0 Biopsy 3
mBAF
1
0.5
0
Individual b Year 0 Biopsy 3
mBAF
Individual b Year 0 Biopsy 3
GN
CNLOH
SD
HD

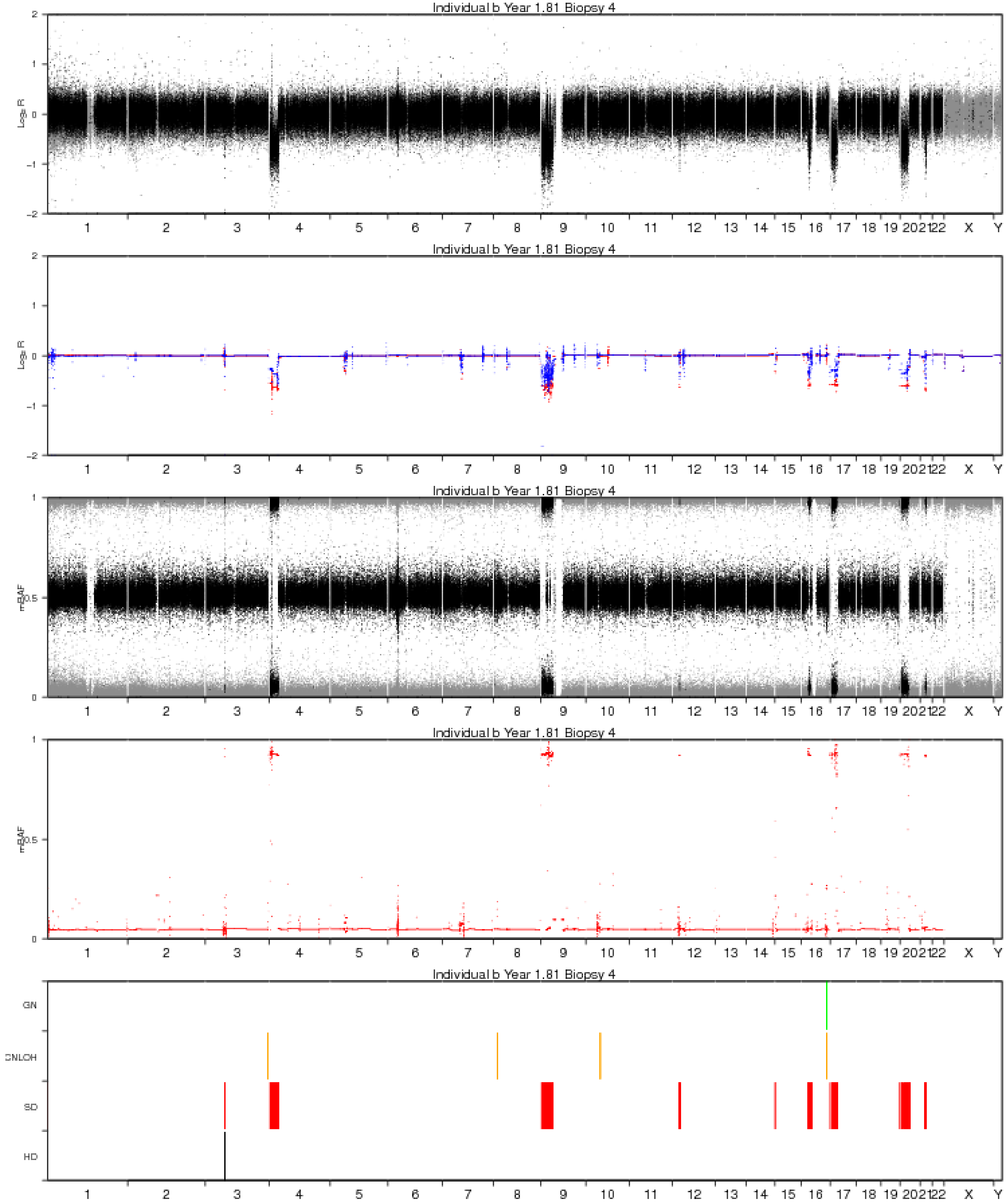

Individual b Year 1.81 Biopsy 4
Log R
mBAF
GN
CNLOH
SD
HD
1 2 3 4 5 6 7 8 9 10 11 12 13 14 15 16 17 18 19 20 21 22 X Y

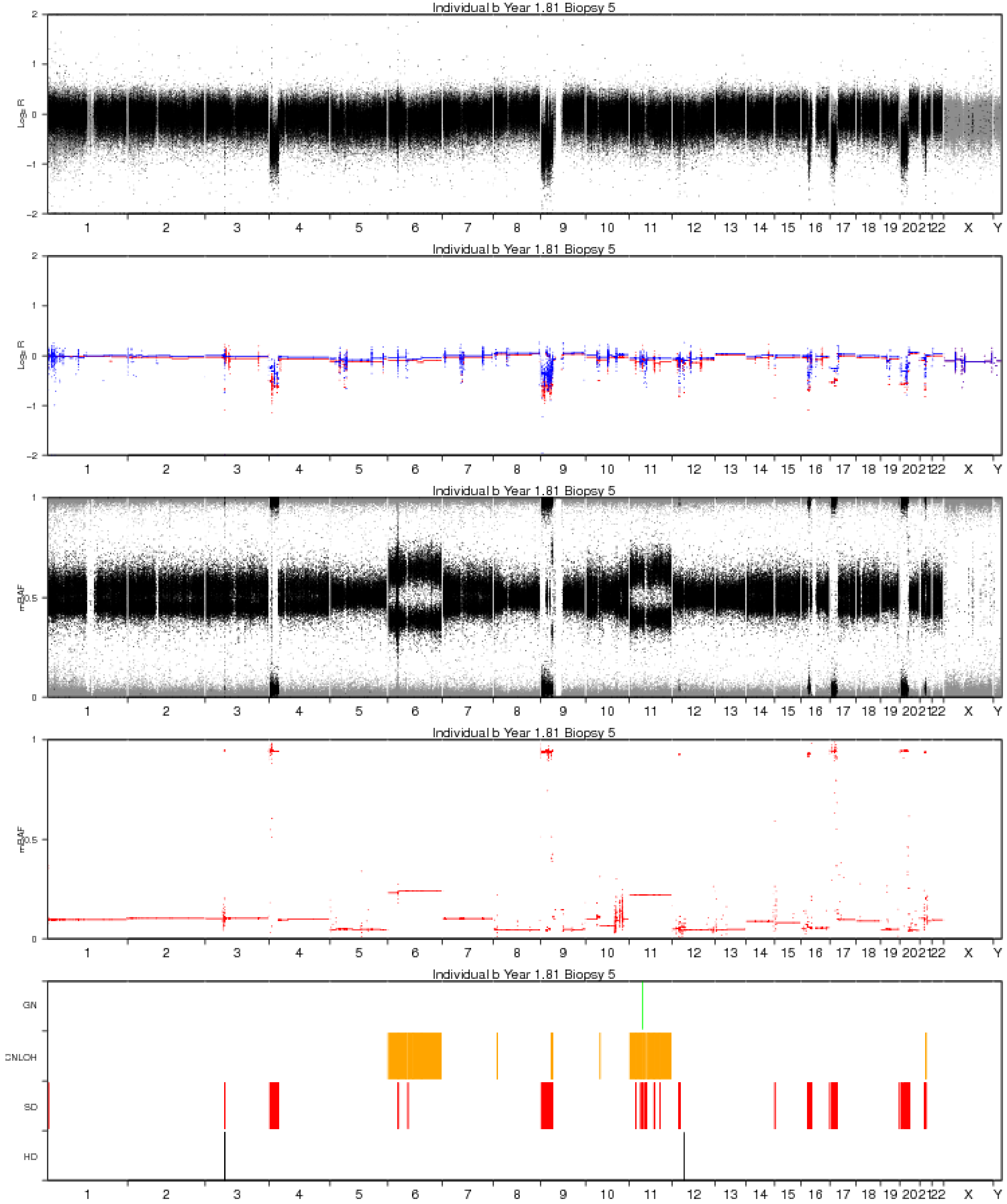
Individual b Year 1.81 Biopsy 5
Log₂ R
mBAF
GN
CNLOH
SD
HD
1 2 3 4 5 6 7 8 9 10 11 12 13 14 15 16 17 18 19 20 21 22 X Y

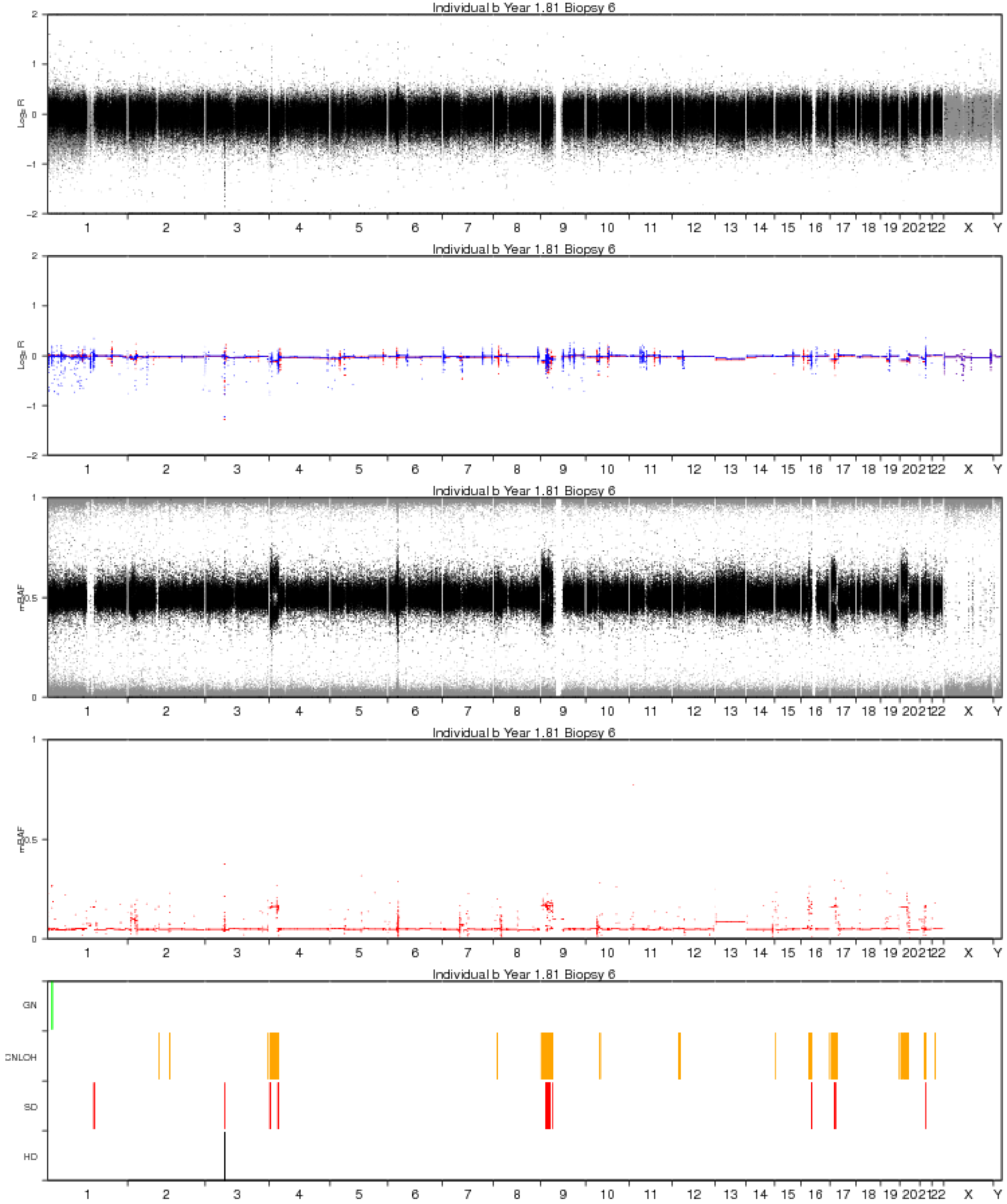
Individual b Year 1.81 Biopsy 6
Log2 R
2
1
0
−1
−2
1 2 3 4 5 6 7 8 9 10 11 12 13 14 15 16 17 18 19 20 21 22 X Y
Individual b Year 1.81 Biopsy 6
Log2 R
2
1
0
−1
−2
1 2 3 4 5 6 7 8 9 10 11 12 13 14 15 16 17 18 19 20 21 22 X Y
Individual b Year 1.81 Biopsy 6
mBAF
1
0.5
0
1 2 3 4 5 6 7 8 9 10 11 12 13 14 15 16 17 18 19 20 21 22 X Y
Individual b Year 1.81 Biopsy 6
mBAF
1
0.5
0
1 2 3 4 5 6 7 8 9 10 11 12 13 14 15 16 17 18 19 20 21 22 X Y
Individual b Year 1.81 Biopsy 6
GN
CNLOH
SD
HD
1 2 3 4 5 6 7 8 9 10 11 12 13 14 15 16 17 18 19 20 21 22 X Y

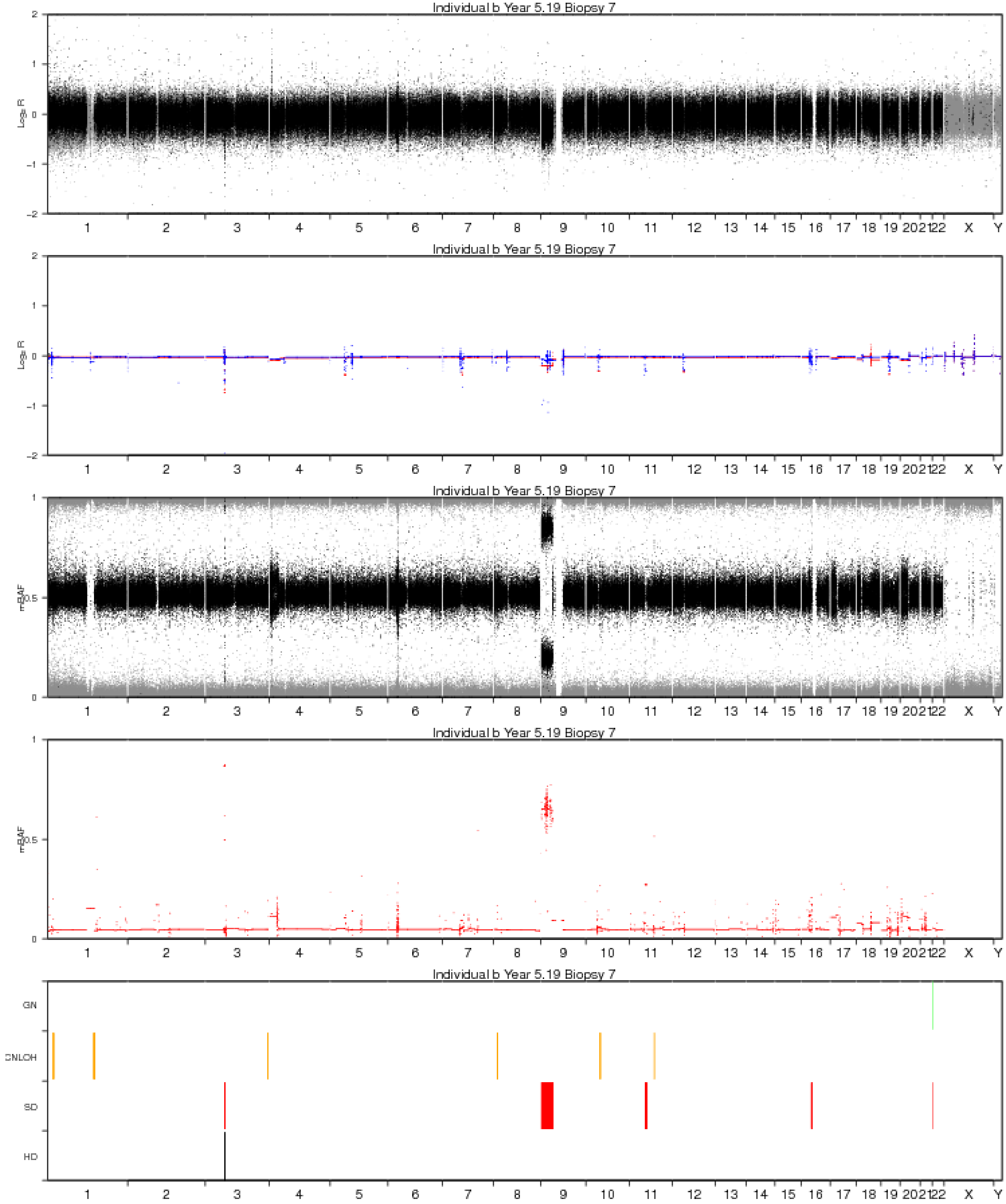

Individual b Year 5.19 Biopsy 7
Log2 R
mBAF
GN
CNLOH
SD
HD
1 2 3 4 5 6 7 8 9 10 11 12 13 14 15 16 17 18 19 20 21 22 X Y

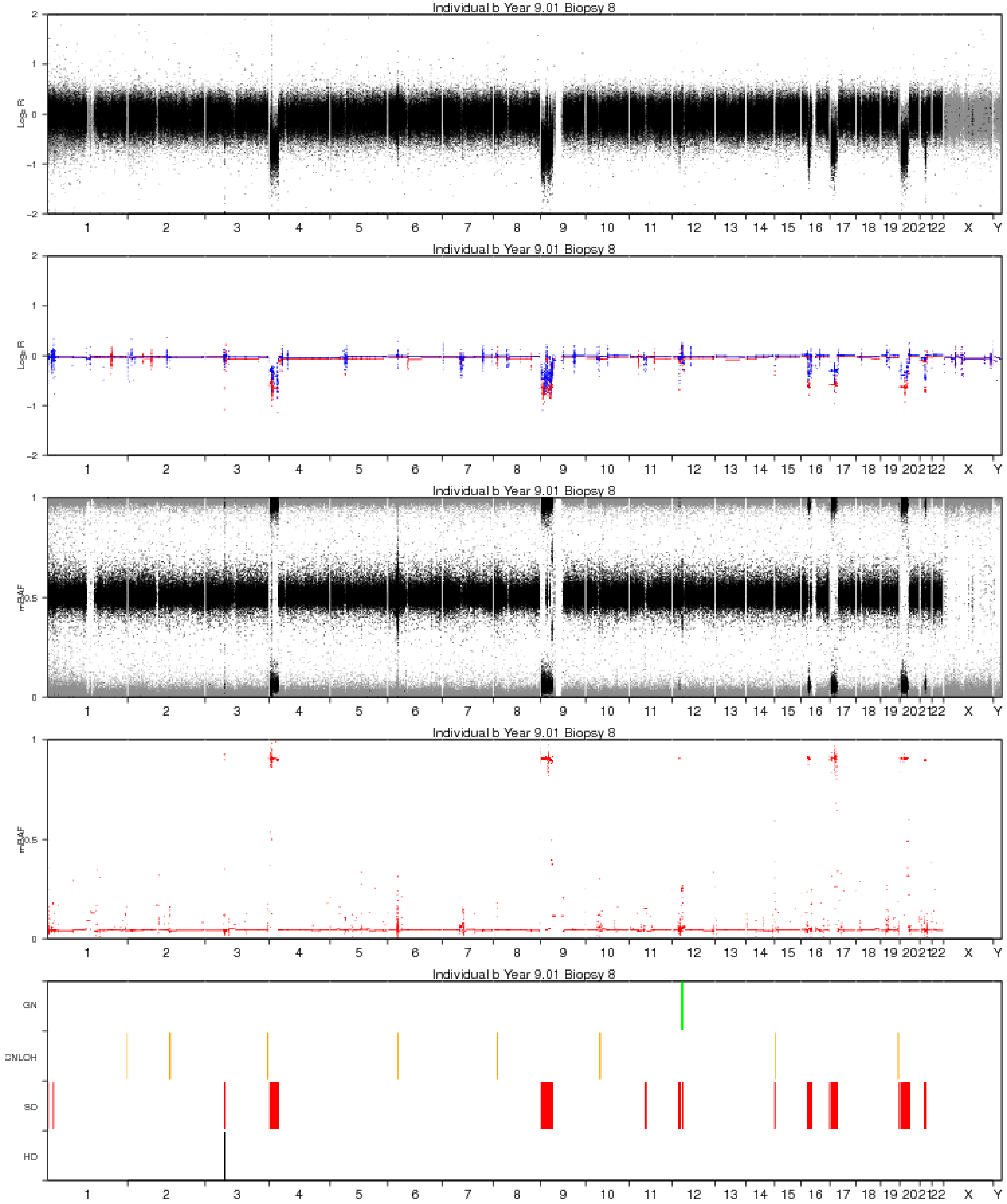
Individual b Year 9.01 Biopsy 8
Log R
mBAF
GN
CNLOH
SD
HD
1 2 3 4 5 6 7 8 9 10 11 12 13 14 15 16 17 18 19 20 21 22 X Y

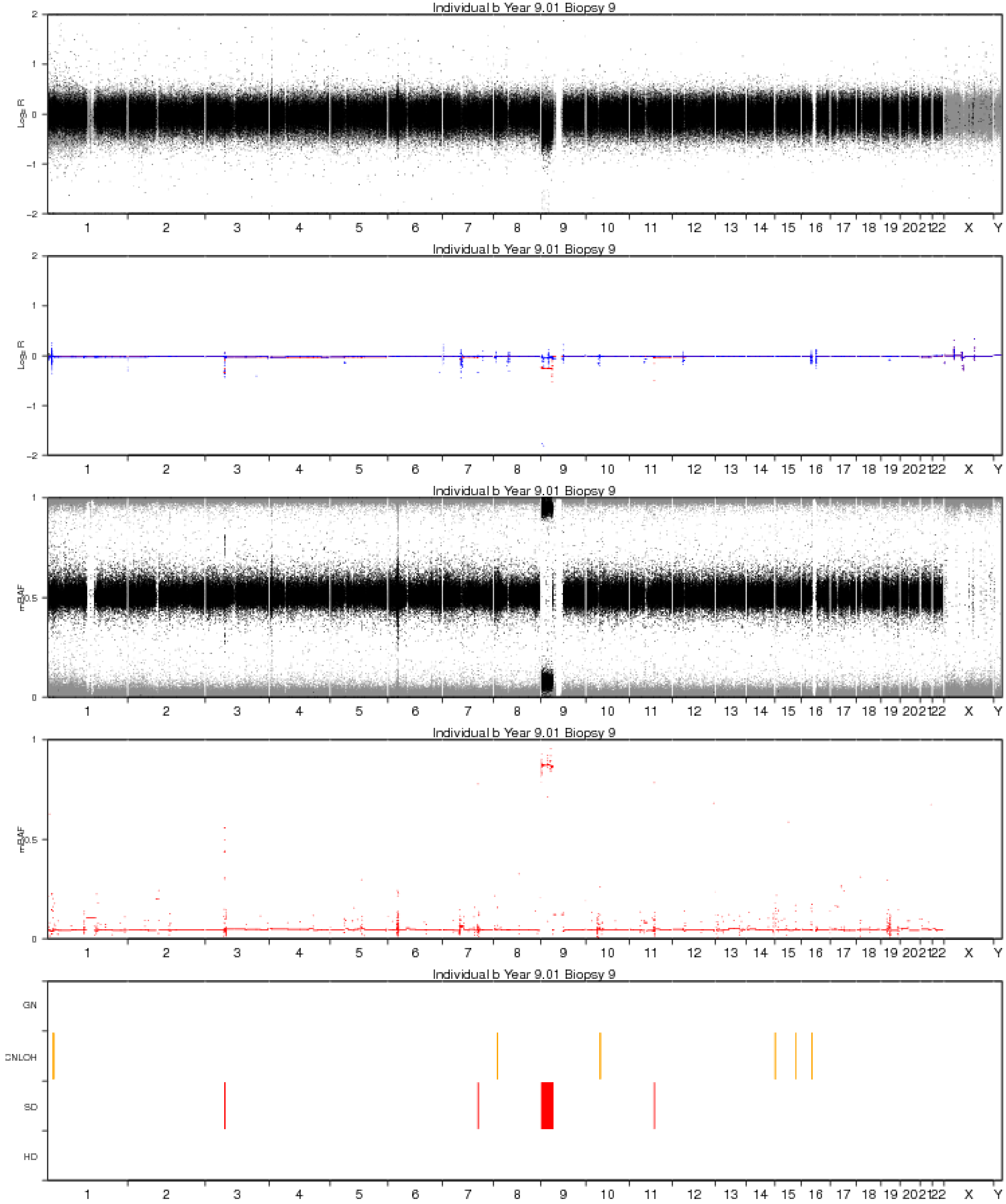
Individual b Year 9.01 Biopsy 9
Log R
mBAF
GN
CNLOH
SD
HD
1 2 3 4 5 6 7 8 9 10 11 12 13 14 15 16 17 18 19 20 21 22 X Y

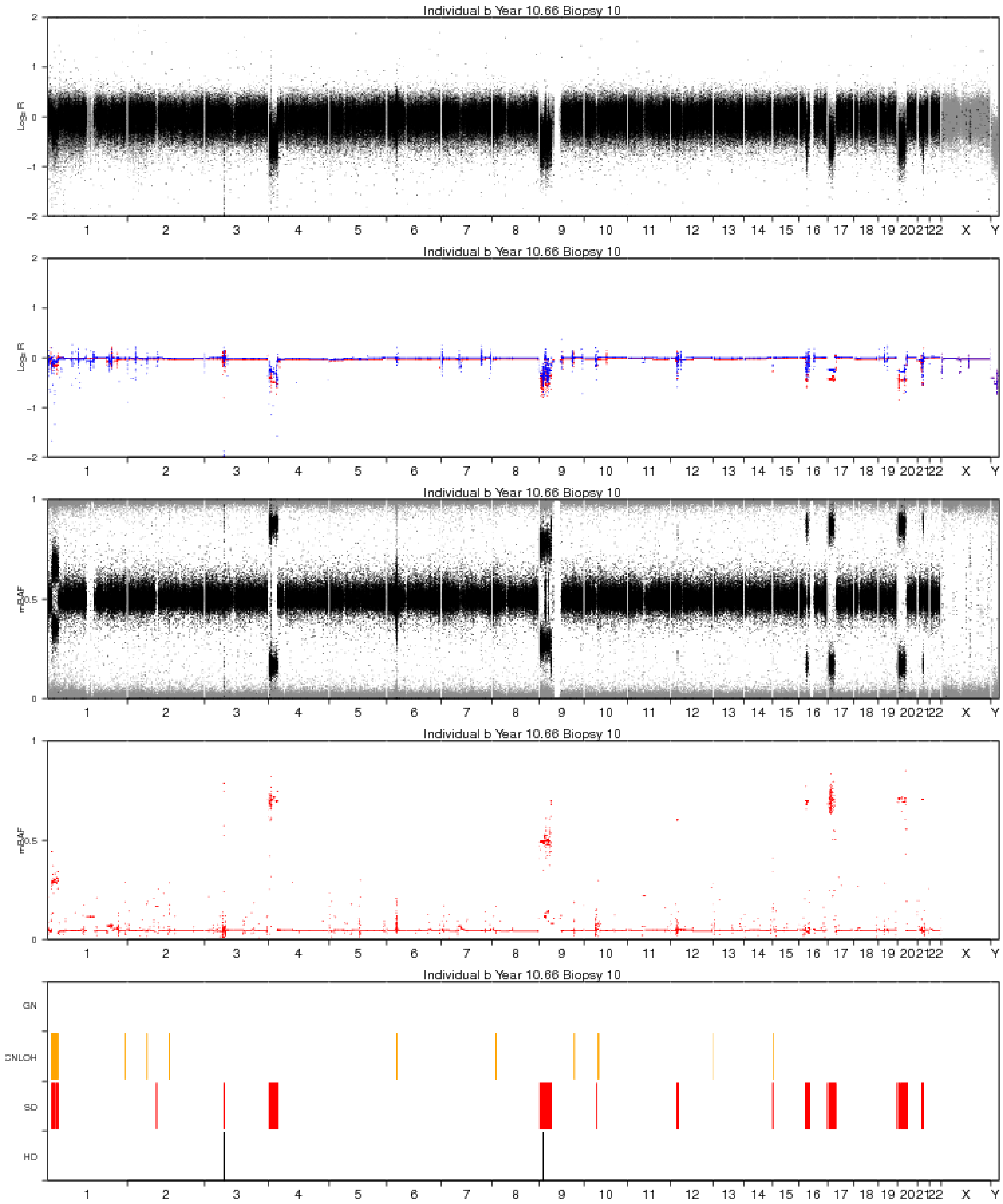

Individual b Year 10.66 Biopsy 10
Log2 R
mBAF
GN
CNLOH
SD
HD
1 2 3 4 5 6 7 8 9 10 11 12 13 14 15 16 17 18 19 20 21 22 X Y

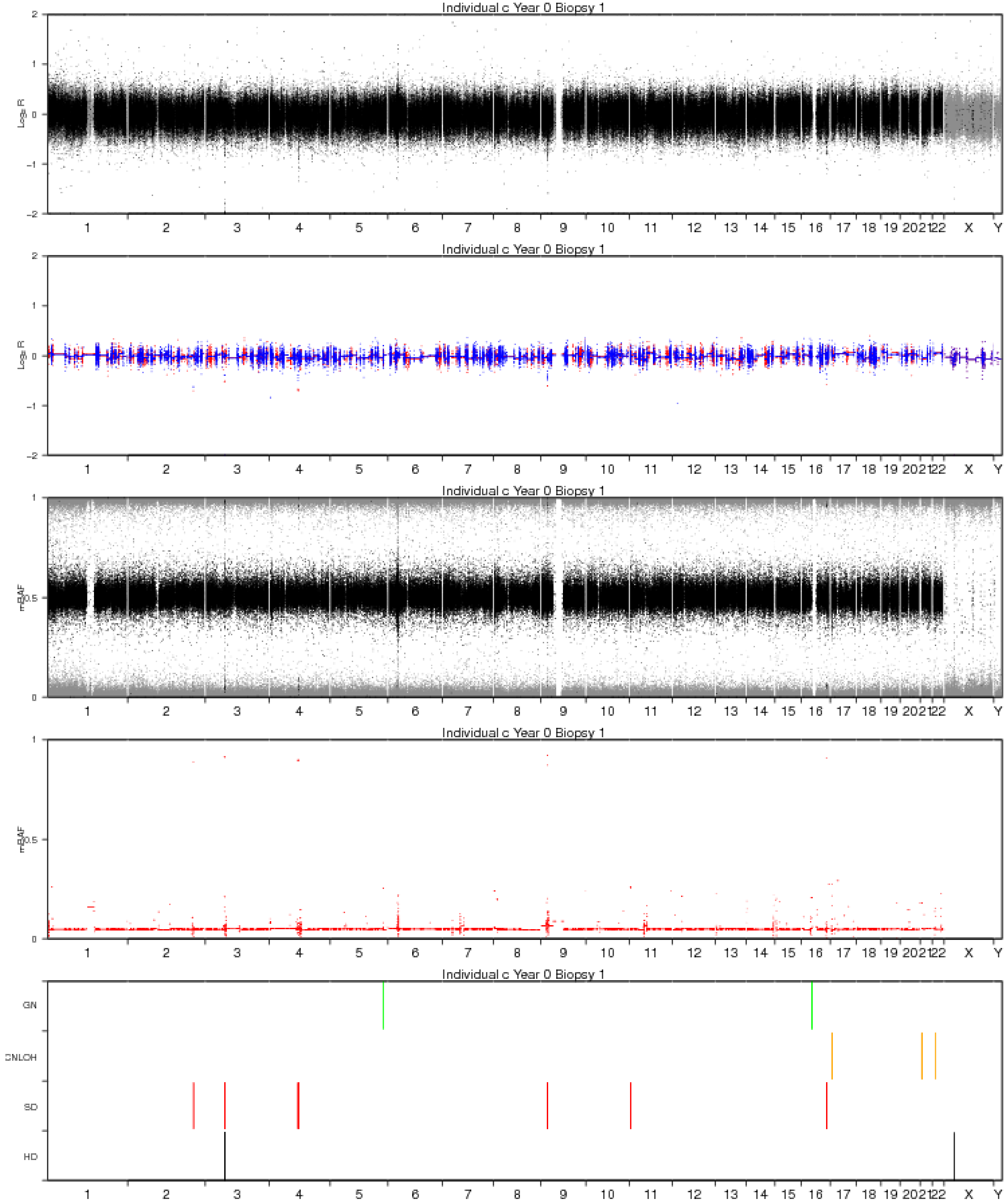
Individual c Year 0 Biopsy 1
Log R
mBAF
GN
CNLOH
SD
HD
1 2 3 4 5 6 7 8 9 10 11 12 13 14 15 16 17 18 19 20 21 22 X Y

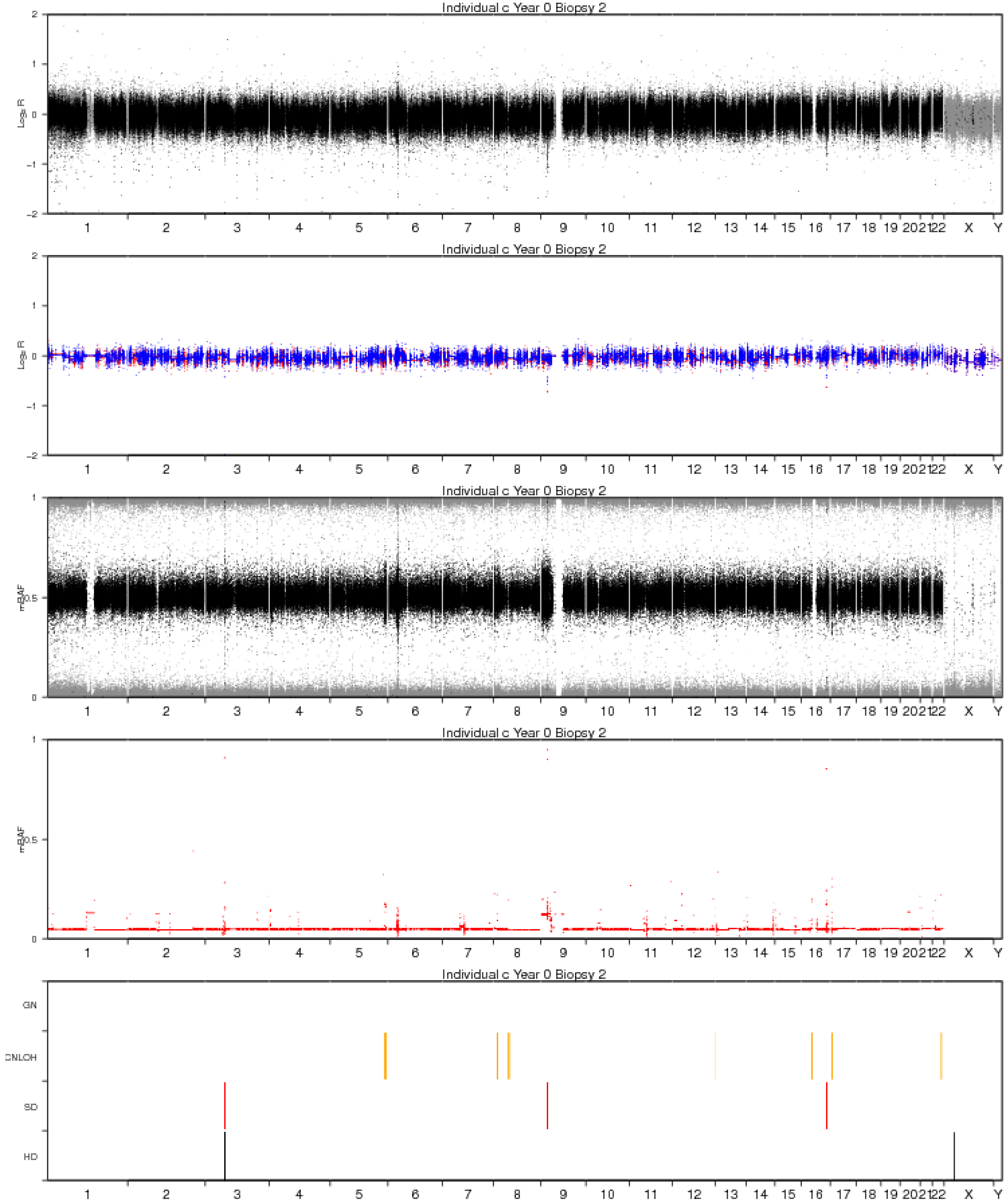

Individual c Year 0 Biopsy 2
Log R
mBAF
GN
CNLOH
SD
HD
1
2
3
4
5
6
7
8
9
10
11
12
13
14
15
16
17
18
19
20
21
22
X
Y

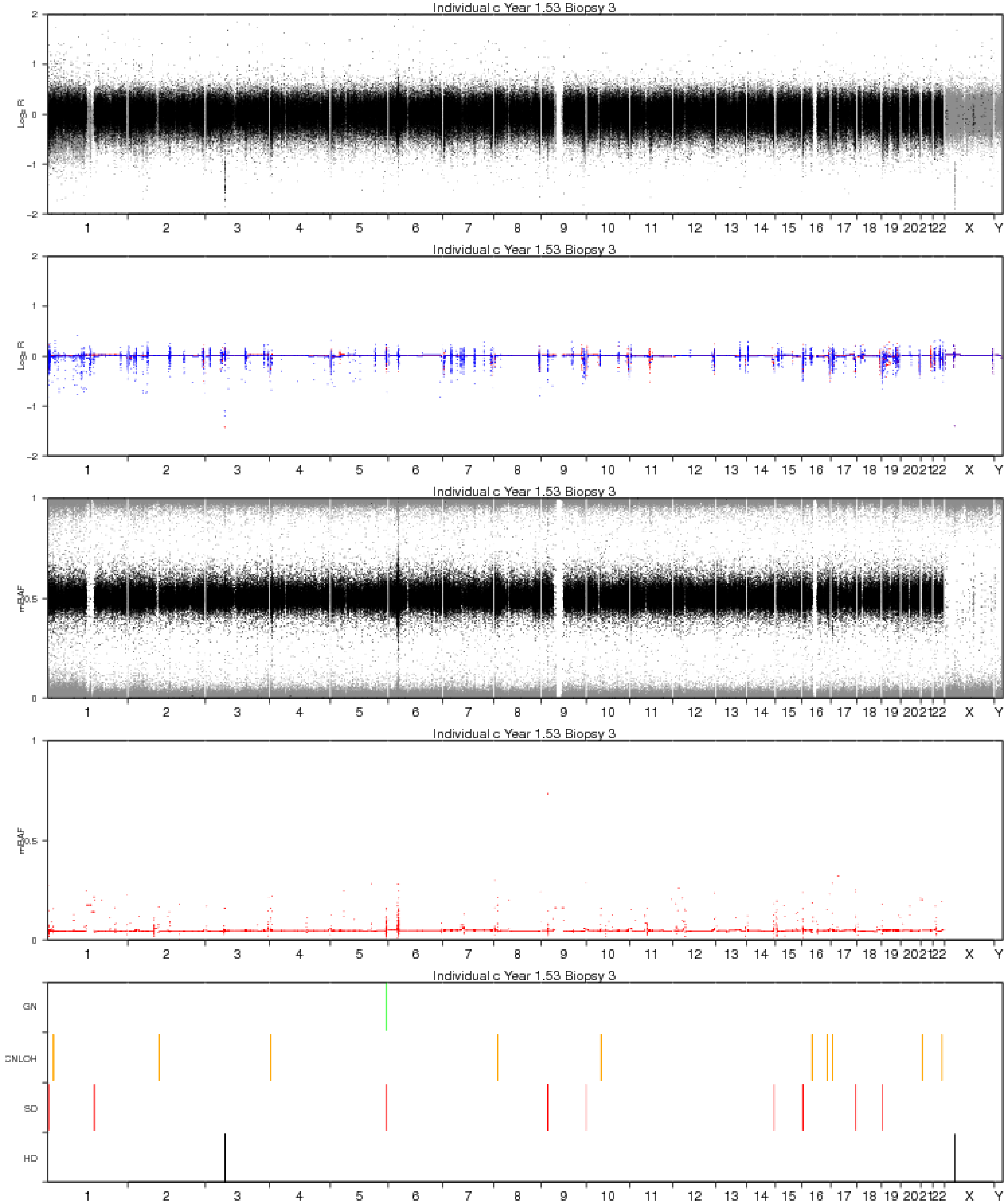
Individual c Year 1.53 Biopsy 3
Log R
mBAF
GN
CNLOH
SD
HD
1 2 3 4 5 6 7 8 9 10 11 12 13 14 15 16 17 18 19 20 21 22 X Y

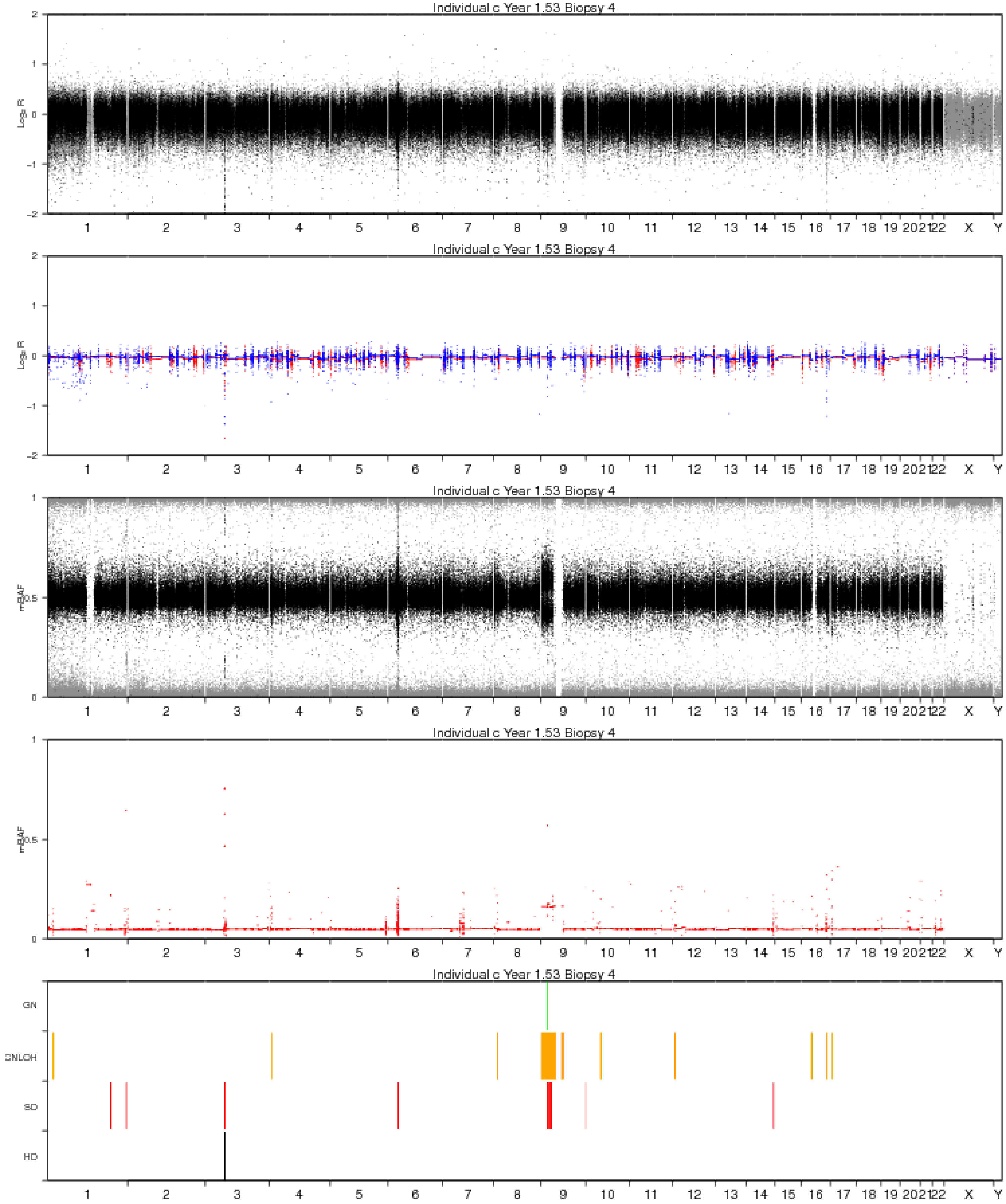
Individual c Year 1.53 Biopsy 4
Log R
mBAF
GN
CNLOH
SD
HD
1 2 3 4 5 6 7 8 9 10 11 12 13 14 15 16 17 18 19 20 21 22 X Y

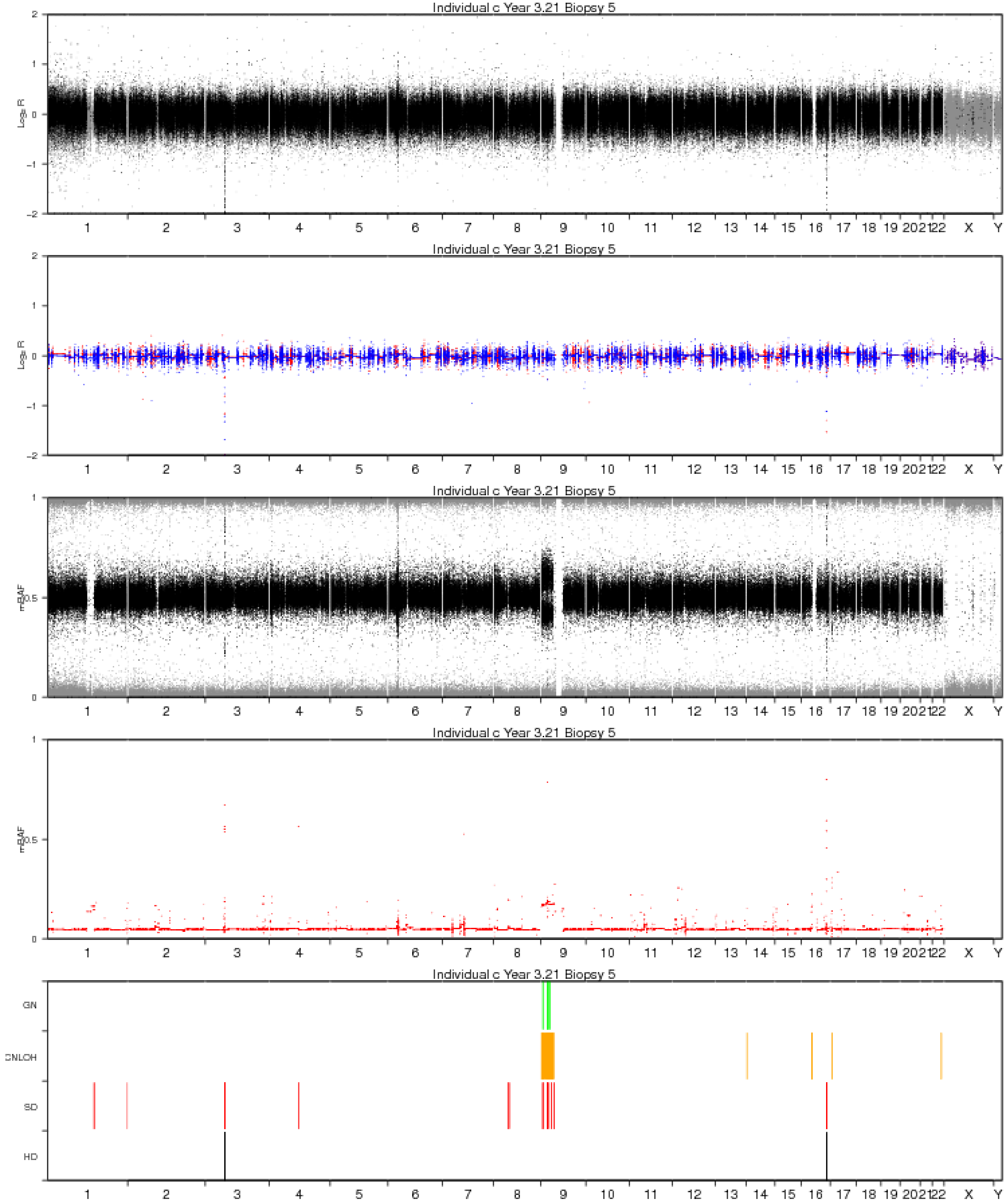

Individual c Year 3.21 Biopsy 5
Log R
mBAF
GN
CNLOH
SD
HD
1 2 3 4 5 6 7 8 9 10 11 12 13 14 15 16 17 18 19 20 21 22 X Y

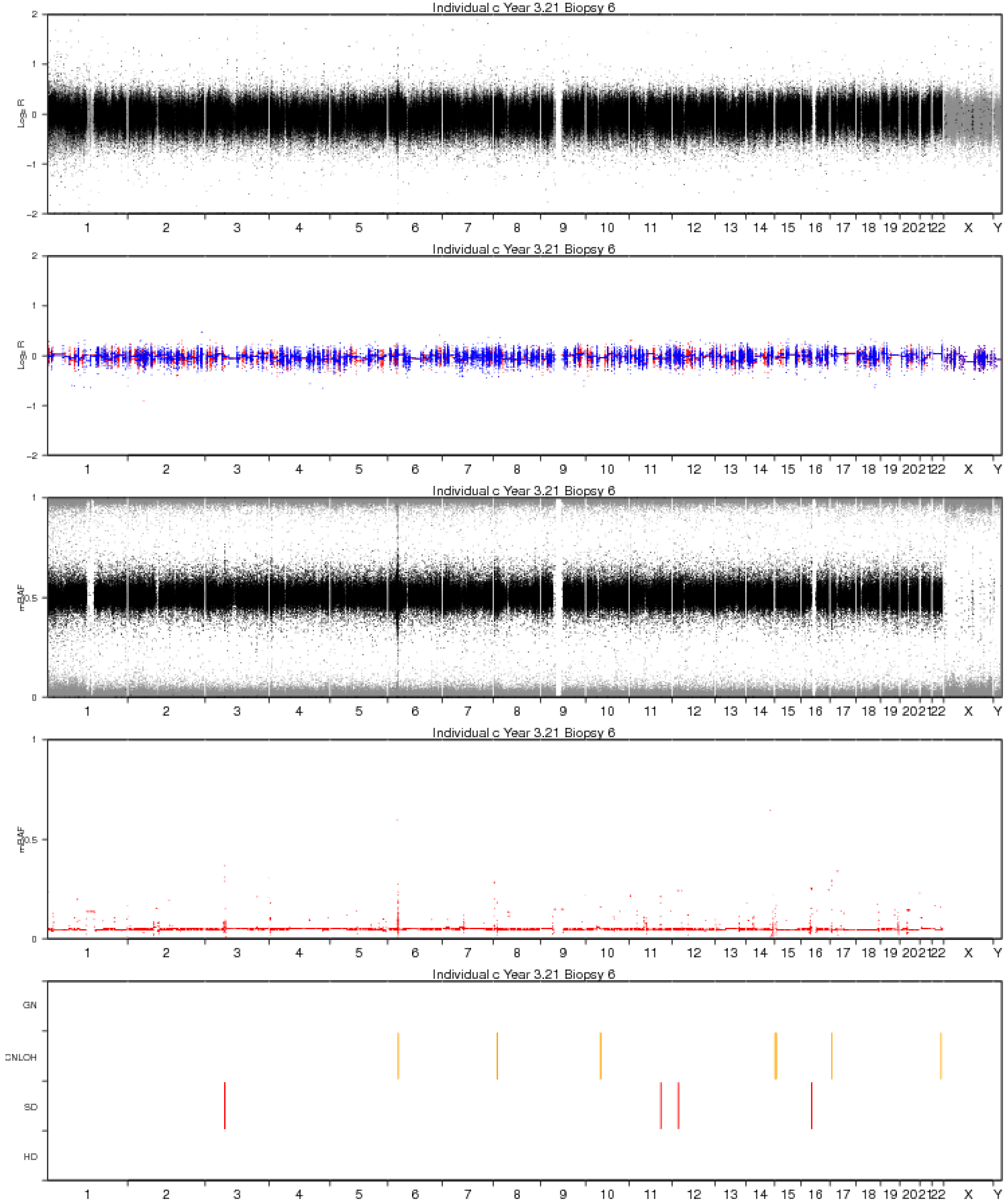
Individual c Year 3.21 Biopsy 6
Log2 R
mBAF
GN
CNLOH
SD
HD
1 2 3 4 5 6 7 8 9 10 11 12 13 14 15 16 17 18 19 20 21 22 X Y

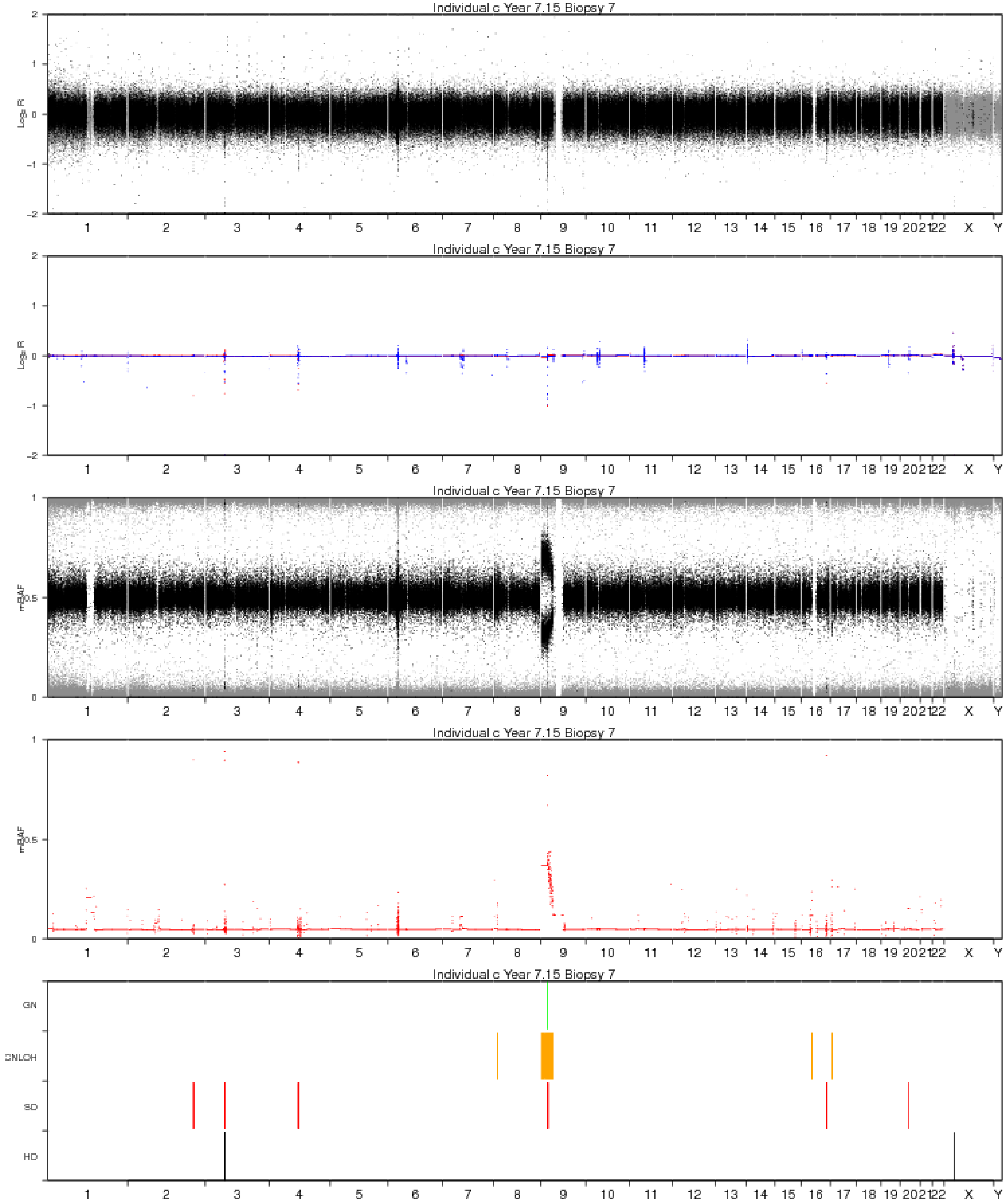
Individual c Year 7.15 Biopsy 7
Log R
mBAF
GN
CNLOH
SD
HD
1 2 3 4 5 6 7 8 9 10 11 12 13 14 15 16 17 18 19 20 21 22 X Y

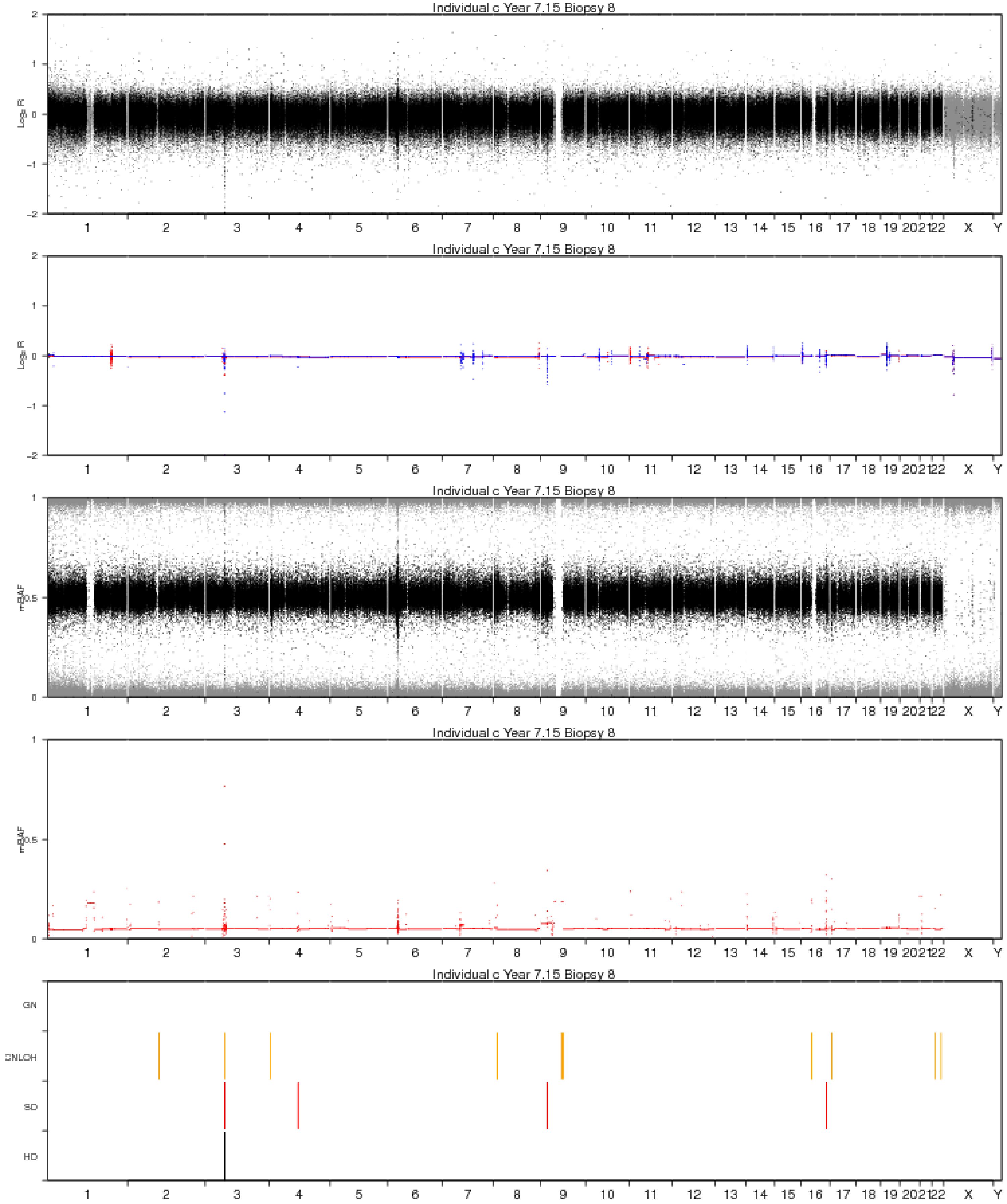
Individual c Year 7.15 Biopsy 8
Log2 R
mBAF
GN
CNLOH
SD
HD
1 2 3 4 5 6 7 8 9 10 11 12 13 14 15 16 17 18 19 20 21 22 X Y

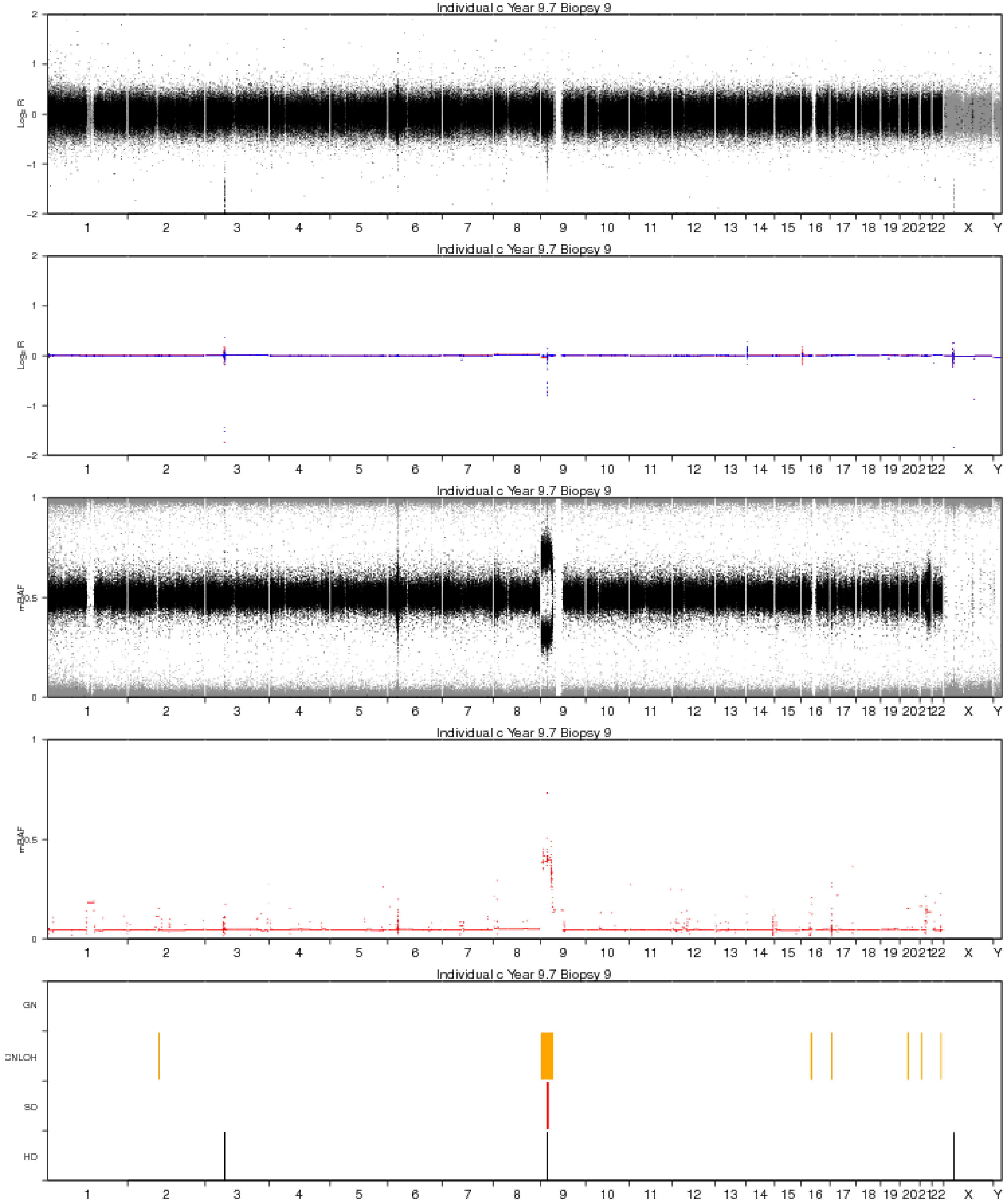
Individual c Year 9.7 Biopsy 9
Log R
mBAF
GN
CNLOH
SD
HD
1 2 3 4 5 6 7 8 9 10 11 12 13 14 15 16 17 18 19 20 21 22 X Y

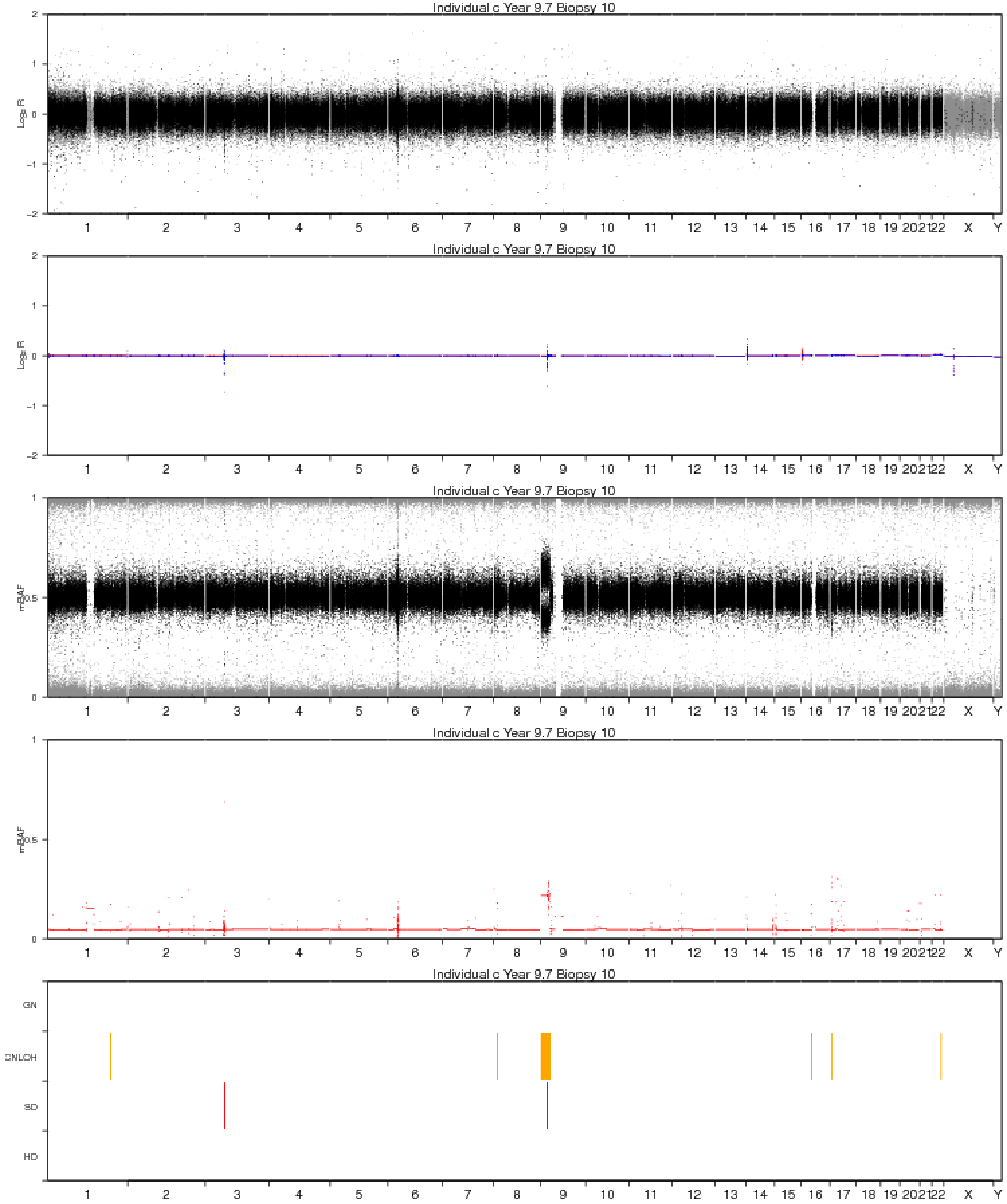
Individual c Year 9.7 Biopsy 10
Log R
mBAF
GN
CNLOH
SD
HD
1 2 3 4 5 6 7 8 9 10 11 12 13 14 15 16 17 18 19 20 21 22 X Y

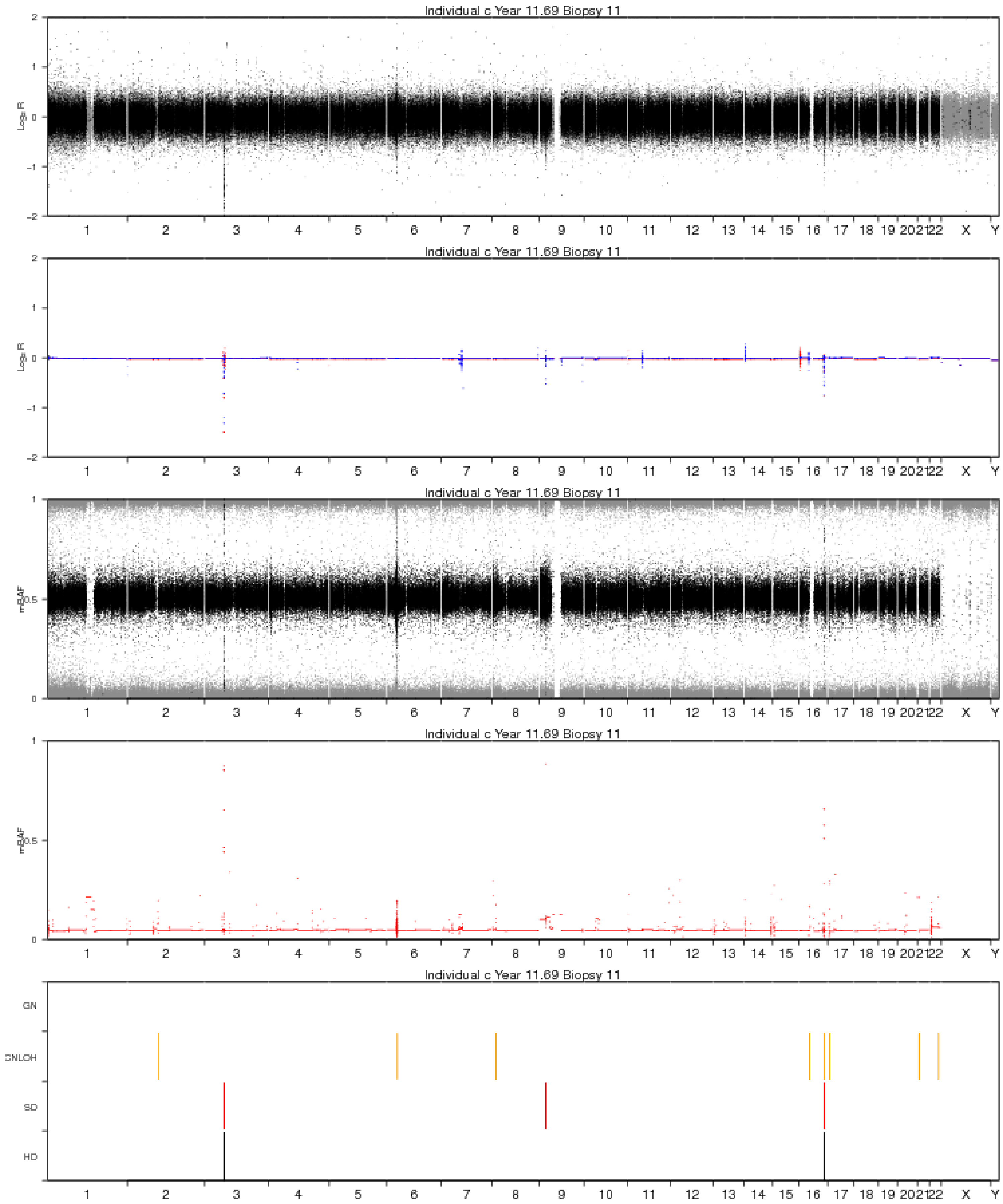
Individual c Year 11.69 Biopsy 11
Log R
mBAF
GN
CNLOH
SD
HD
1 2 3 4 5 6 7 8 9 10 11 12 13 14 15 16 17 18 19 20 21 22 X Y

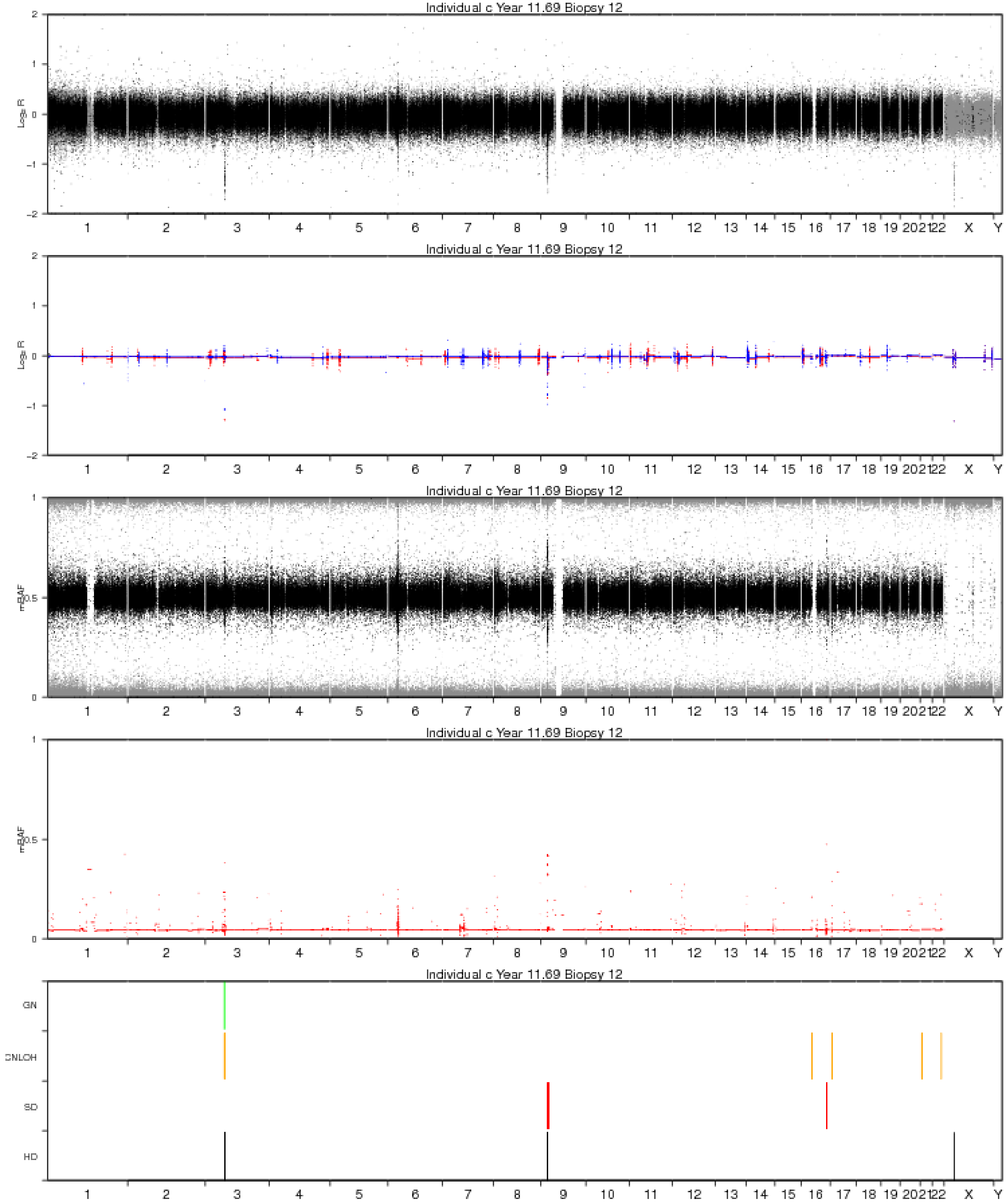
Individual c Year 11.69 Biopsy 12
Log R
mBAF
GN
CNLOH
SD
HD
1 2 3 4 5 6 7 8 9 10 11 12 13 14 15 16 17 18 19 20 21 22 X Y

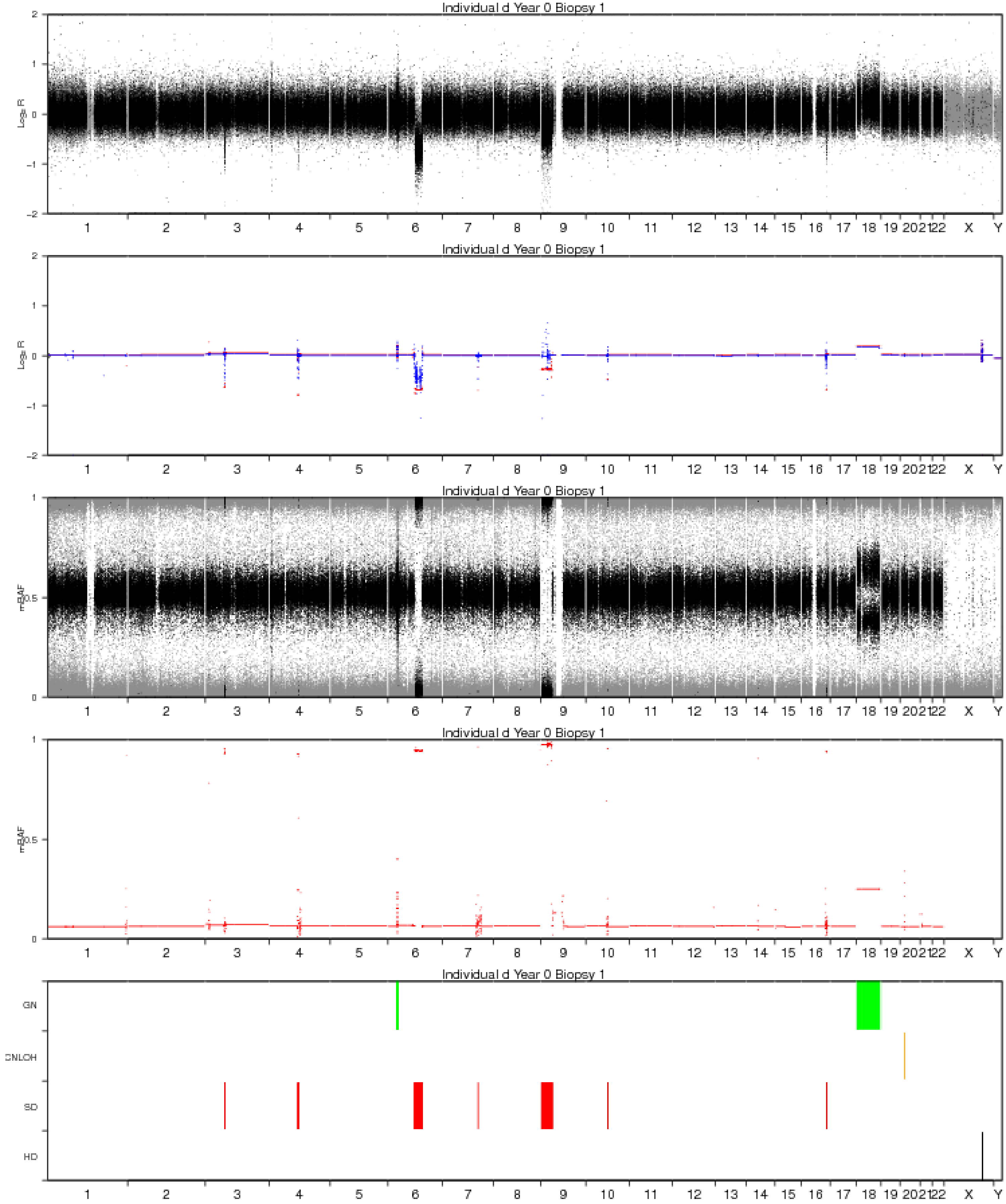

Individual d Year 0 Biopsy 1
Log R
mBAF
GN
CNLOH
SD
HD
1 2 3 4 5 6 7 8 9 10 11 12 13 14 15 16 17 18 19 20 21 22 X Y

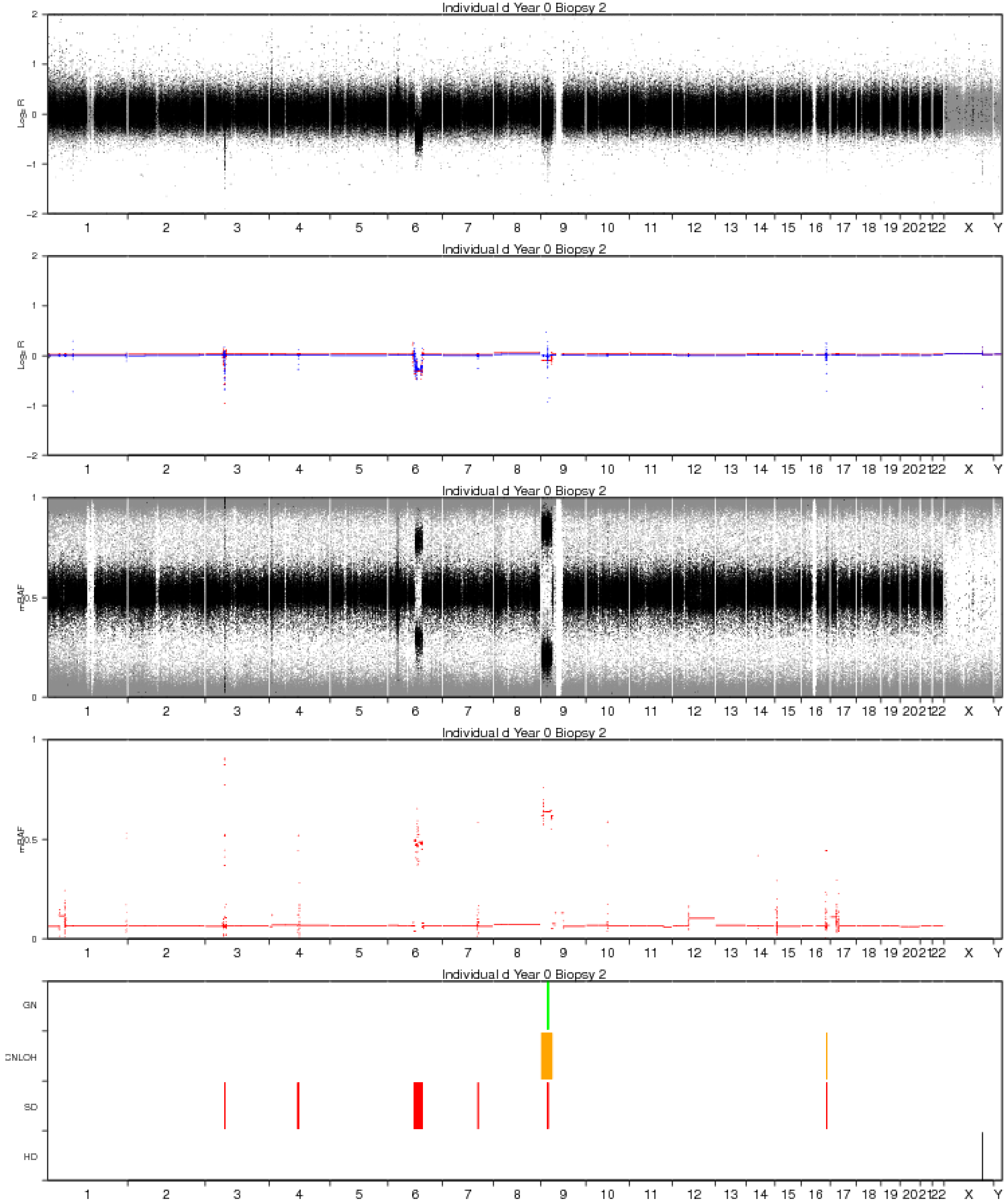
Individual d Year 0 Biopsy 2
Log R
mBAF
GN
CNLOH
SD
HD
1 2 3 4 5 6 7 8 9 10 11 12 13 14 15 16 17 18 19 20 21 22 X Y

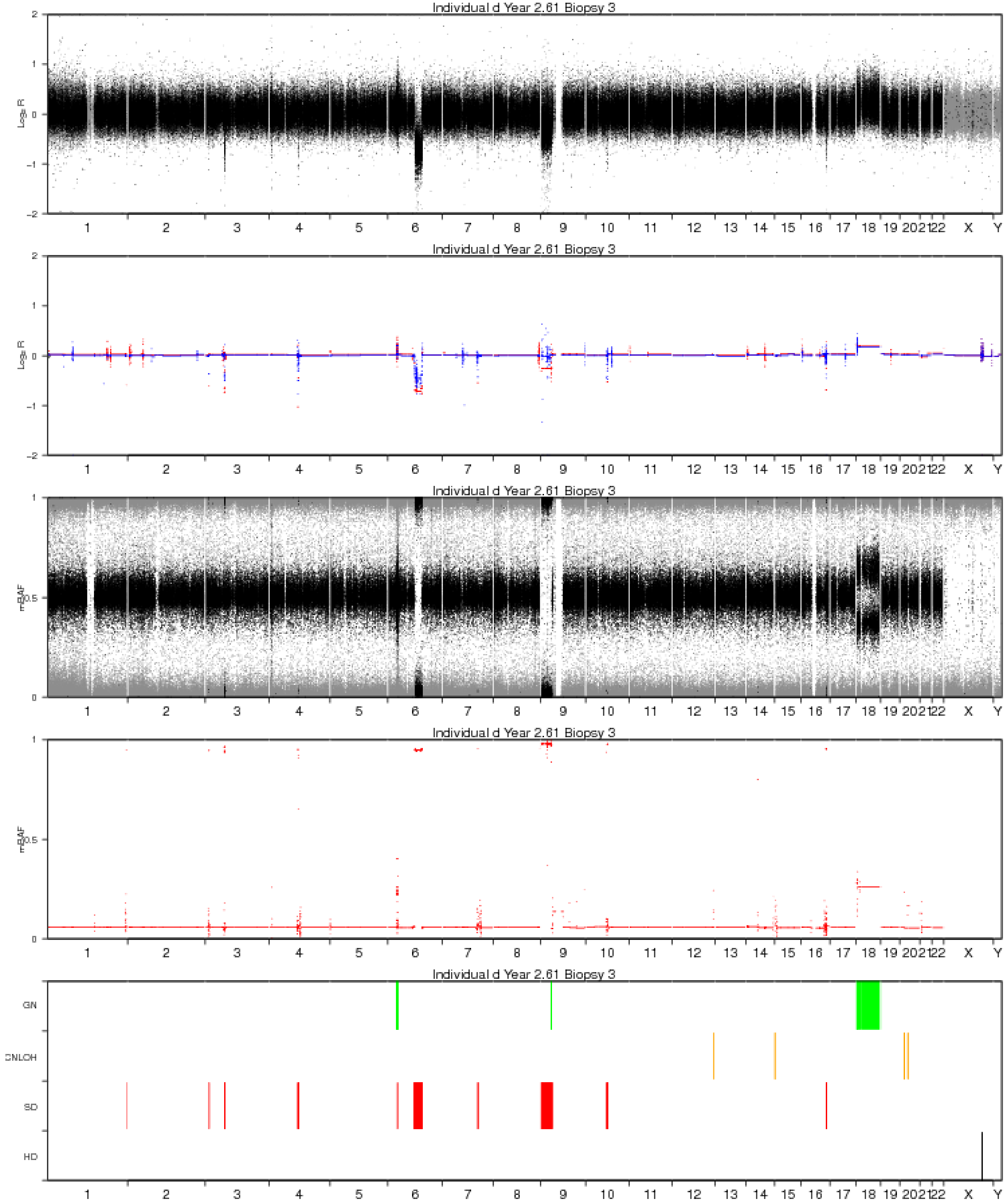
Individual d Year 2.61 Biopsy 3
Log R
mBAF
GN
CNLOH
SD
HD
1 2 3 4 5 6 7 8 9 10 11 12 13 14 15 16 17 18 19 20 21 22 X Y

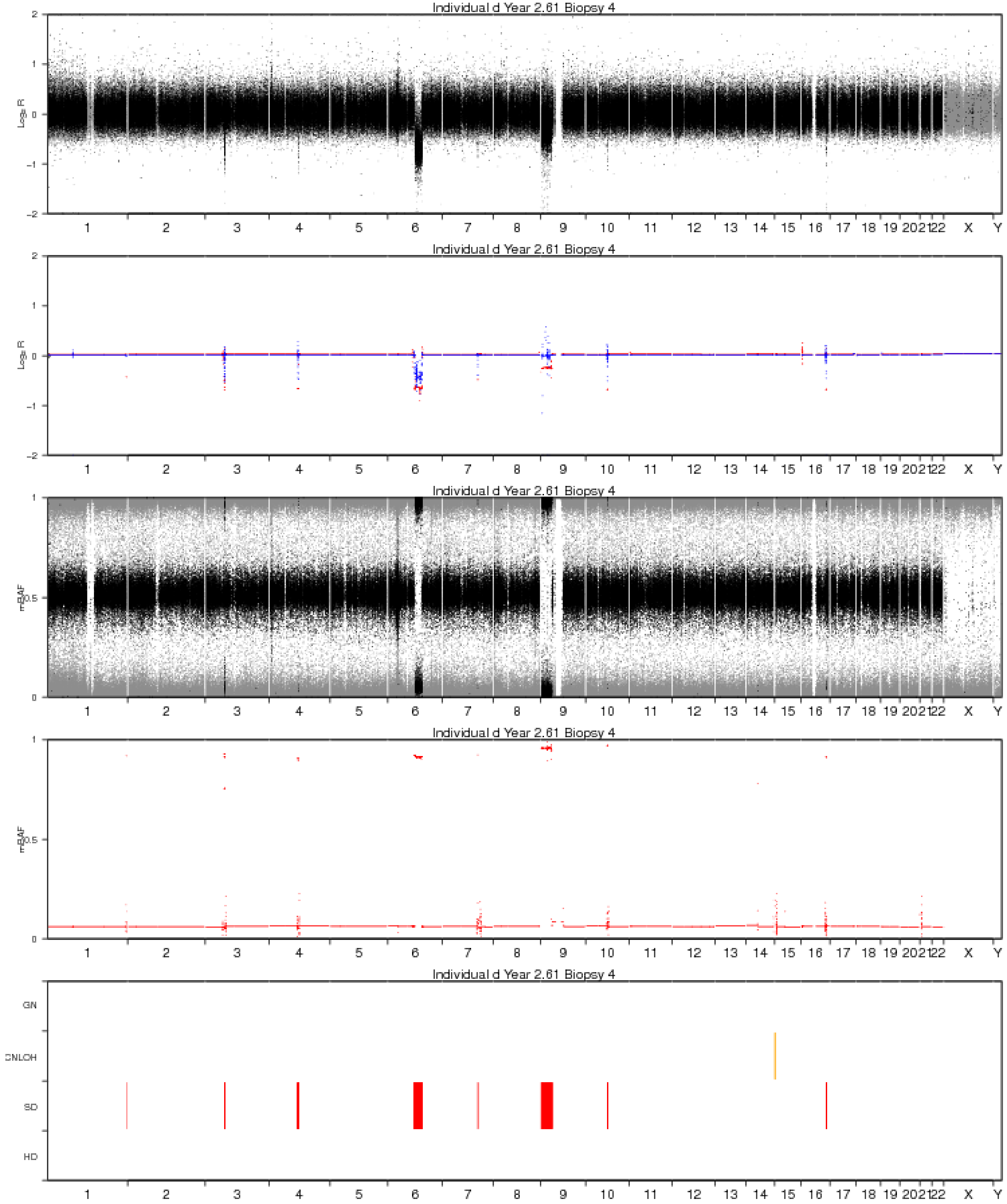
Individual d Year 2.61 Biopsy 4
Log R
mBAF
GN
CNLOH
SD
HD
1 2 3 4 5 6 7 8 9 10 11 12 13 14 15 16 17 18 19 20 21 22 X Y

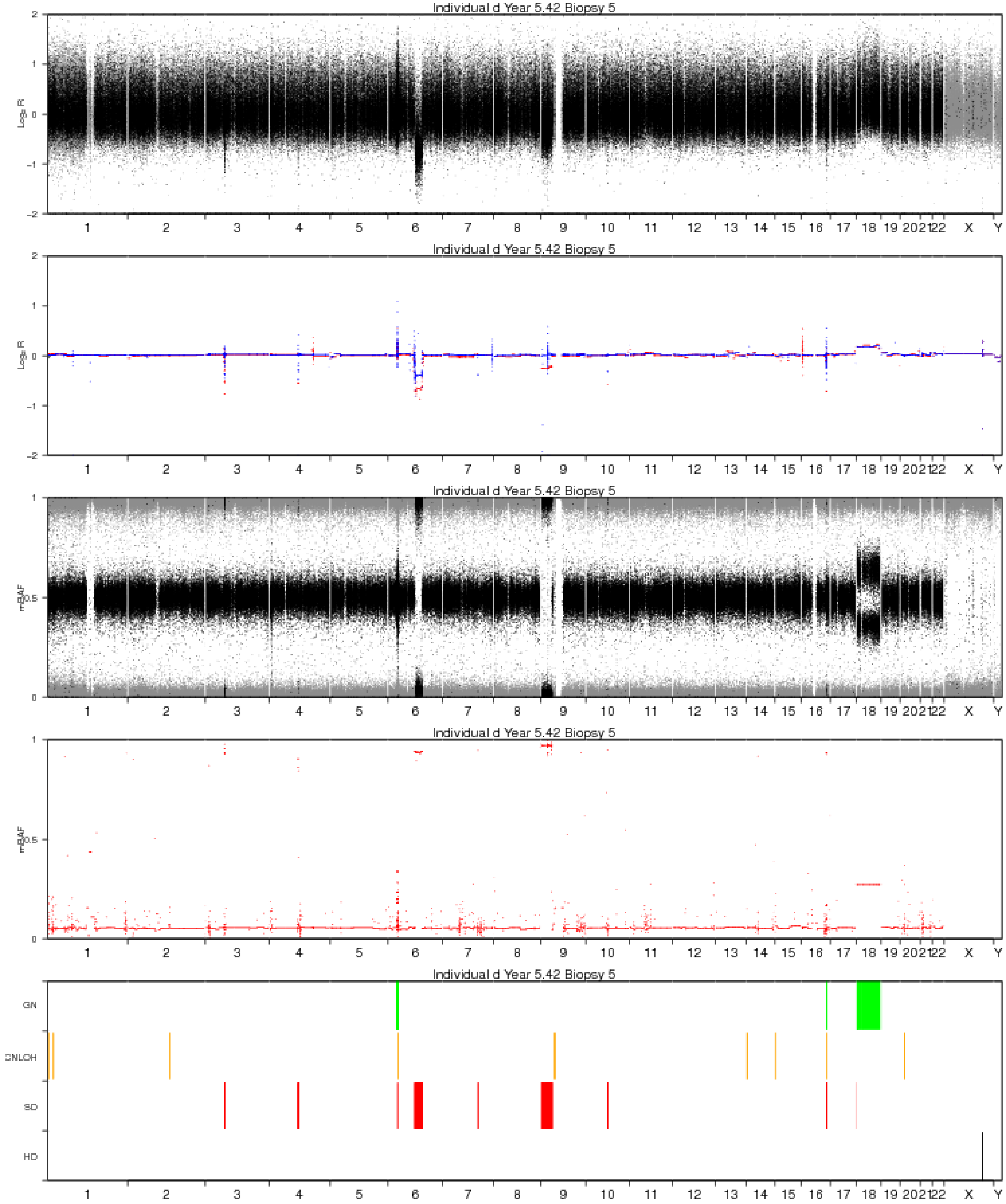
Individual d Year 5.42 Biopsy 5
Log R
mBAF
GN
CNLOH
SD
HD
1 2 3 4 5 6 7 8 9 10 11 12 13 14 15 16 17 18 19 20 21 22 X Y

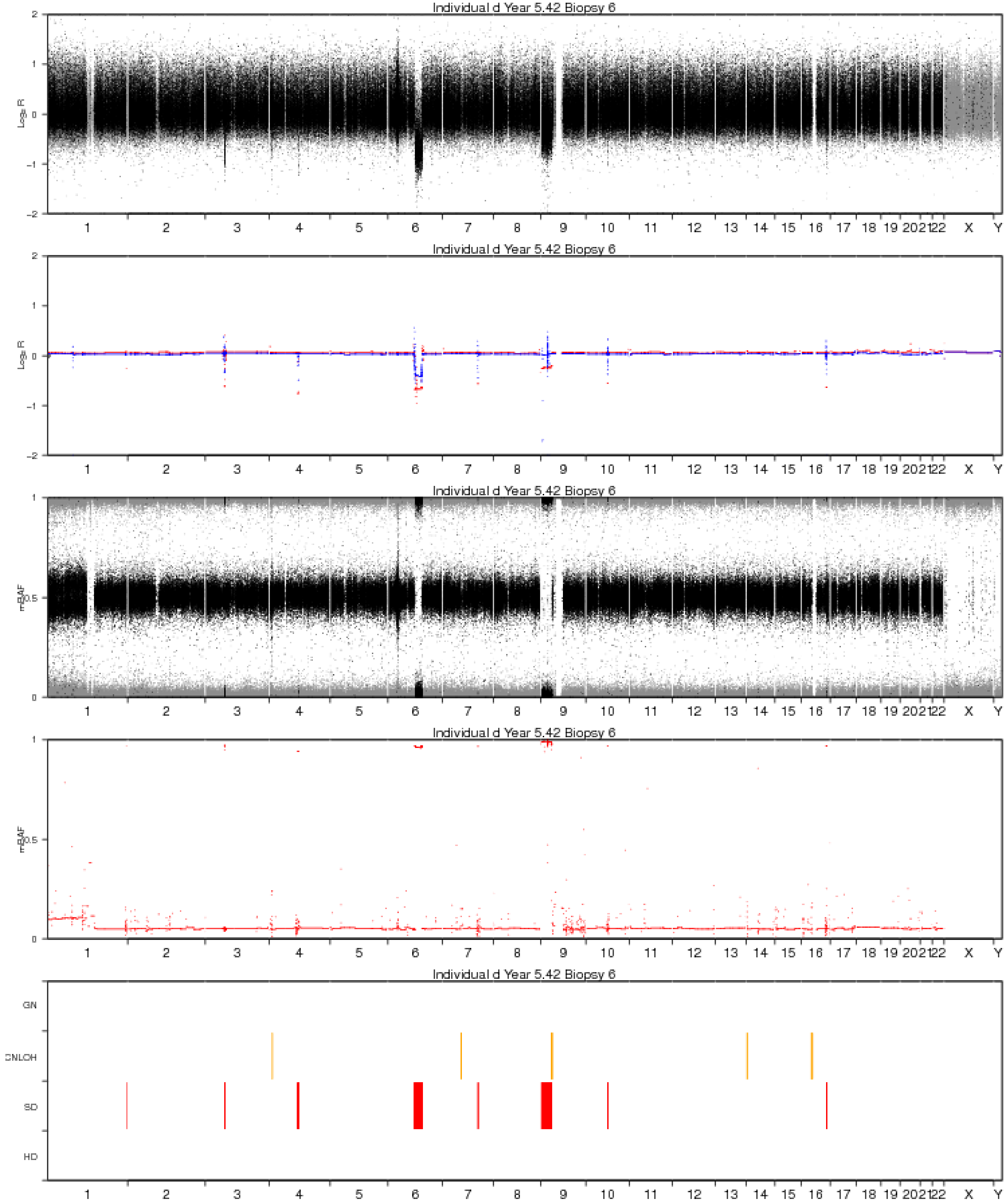
Individual d Year 5.42 Biopsy 6
Log R
mBAF
GN
CNLOH
SD
HD
1 2 3 4 5 6 7 8 9 10 11 12 13 14 15 16 17 18 19 20 21 22 X Y

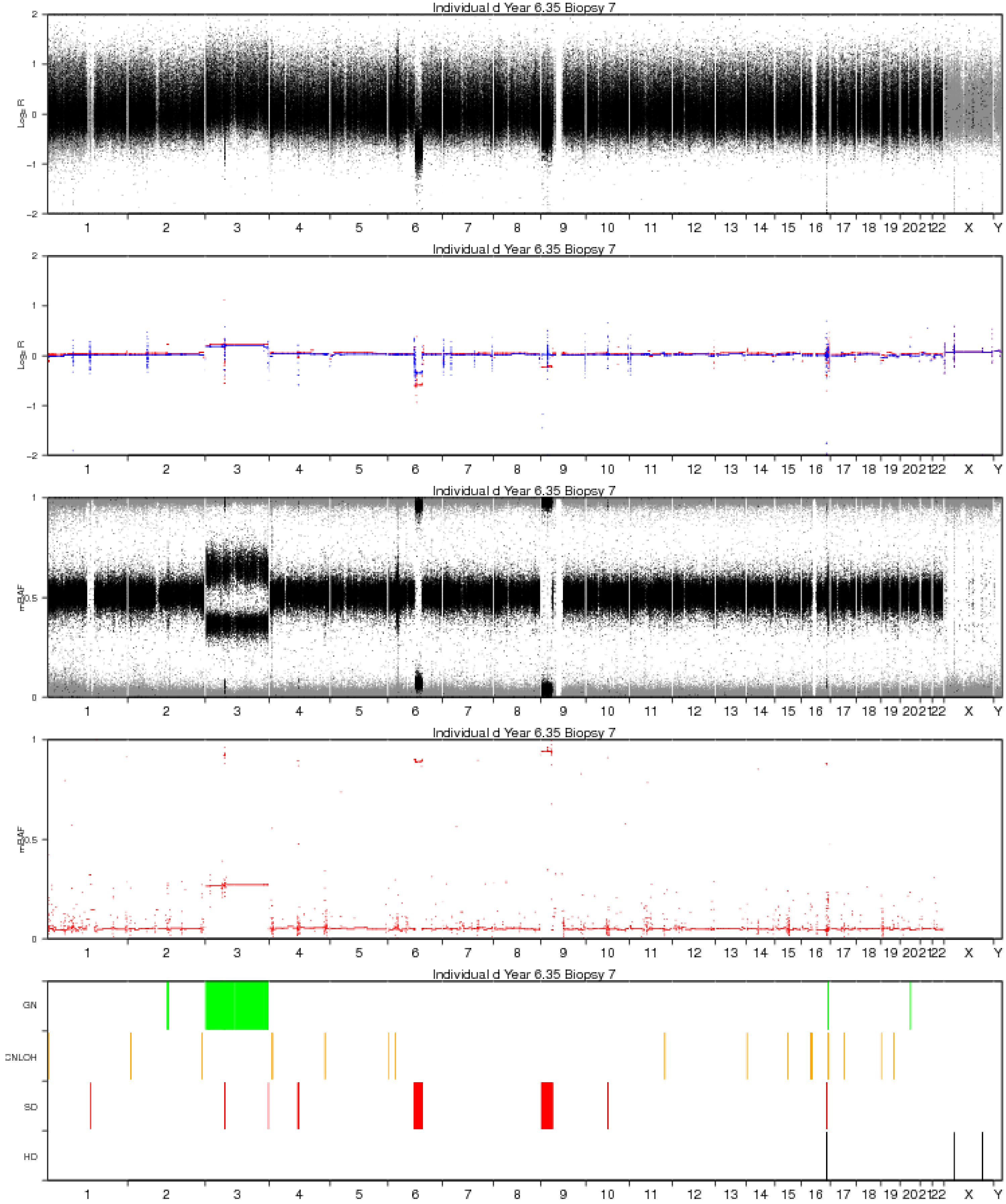
Individual d Year 6.35 Biopsy 7
Log2 R
mBAF
GN
CNLOH
SD
HD
1 2 3 4 5 6 7 8 9 10 11 12 13 14 15 16 17 18 19 20 21 22 X Y

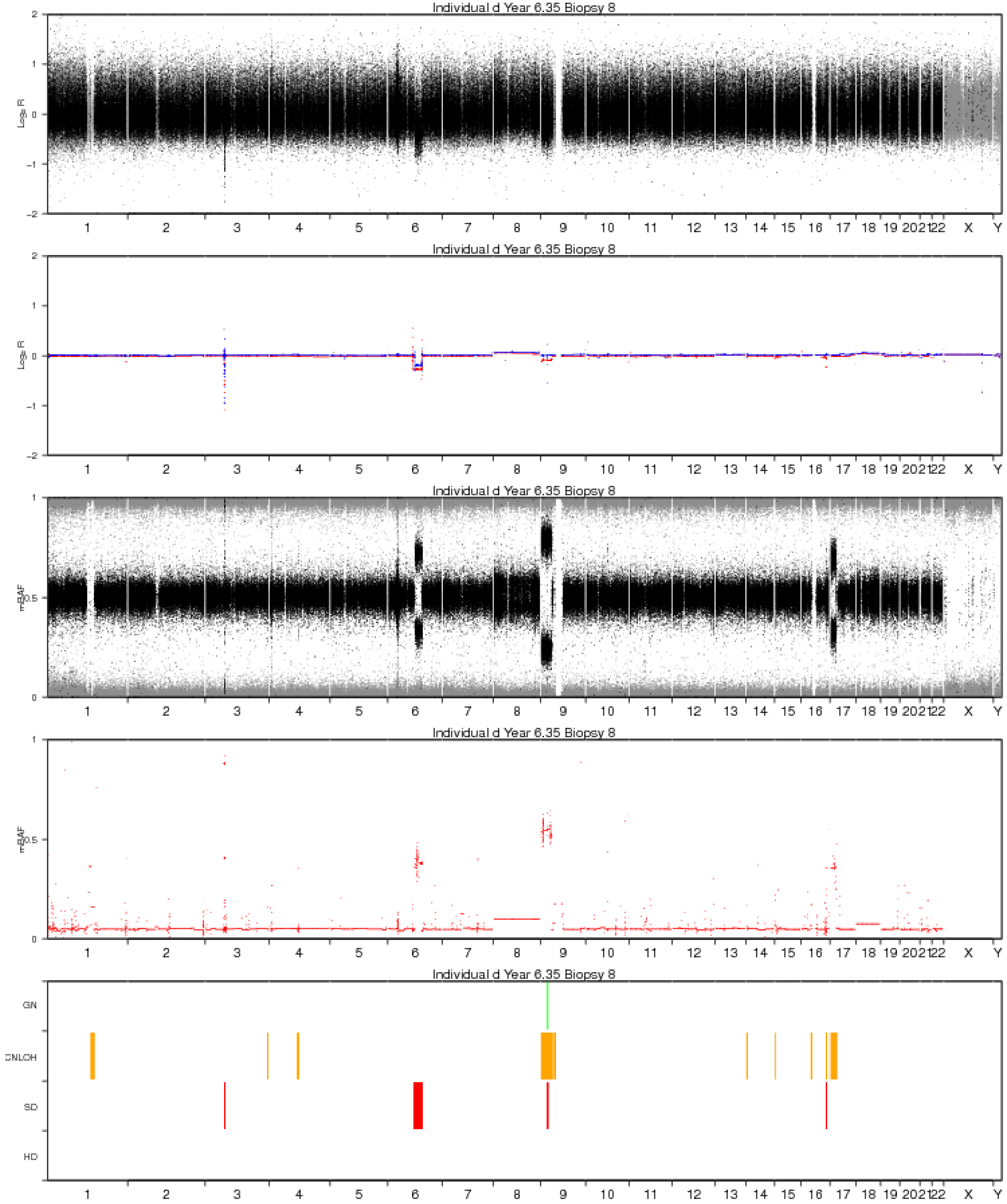
Individual d Year 6.35 Biopsy 8
Log R
mBAF
GN
CNLOH
SD
HD
1 2 3 4 5 6 7 8 9 10 11 12 13 14 15 16 17 18 19 20 21 22 X Y

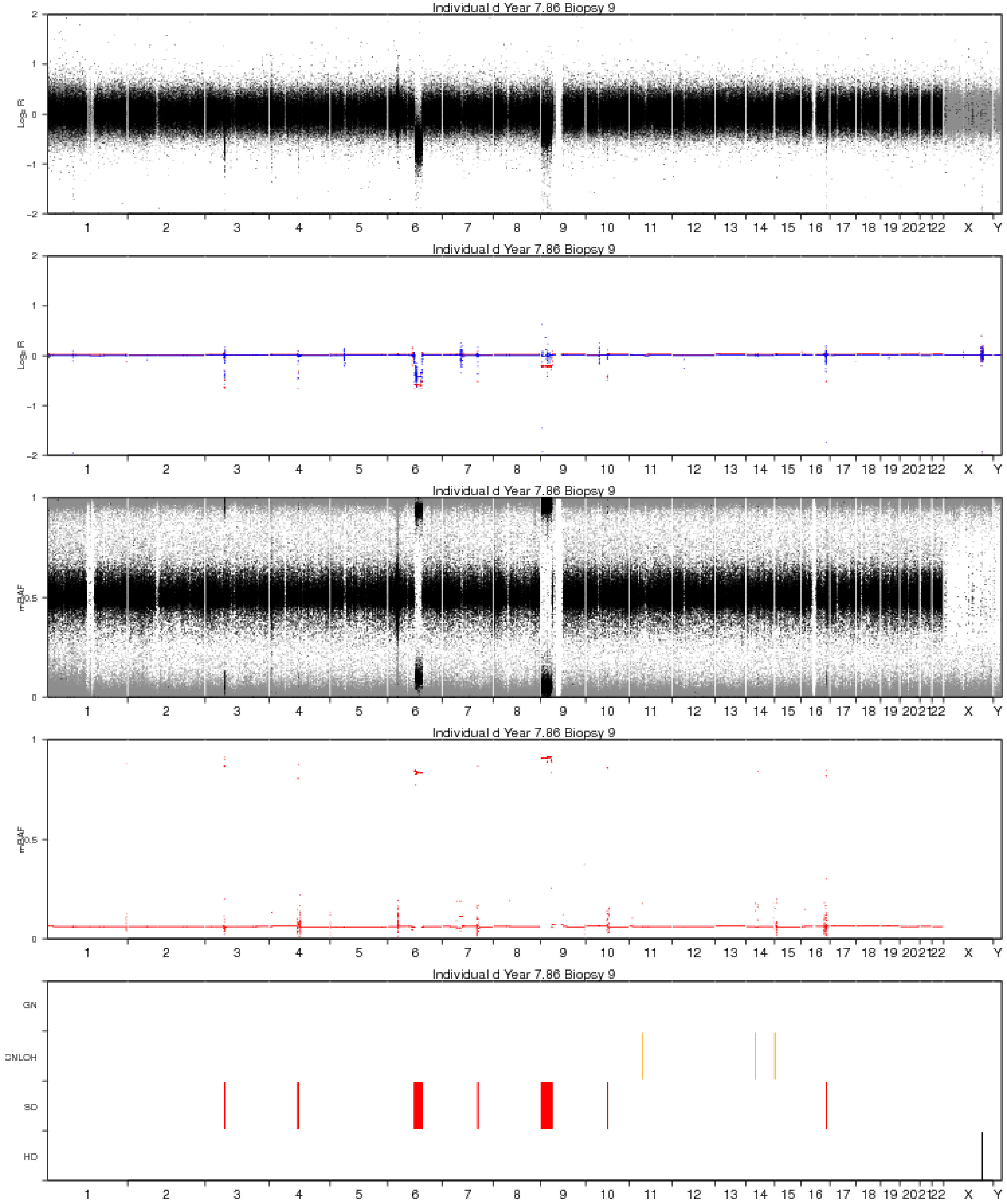
Individual d Year 7.86 Biopsy 9
Log R
mBAF
GN
CNLOH
SD
HD
1 2 3 4 5 6 7 8 9 10 11 12 13 14 15 16 17 18 19 20 21 22 X Y

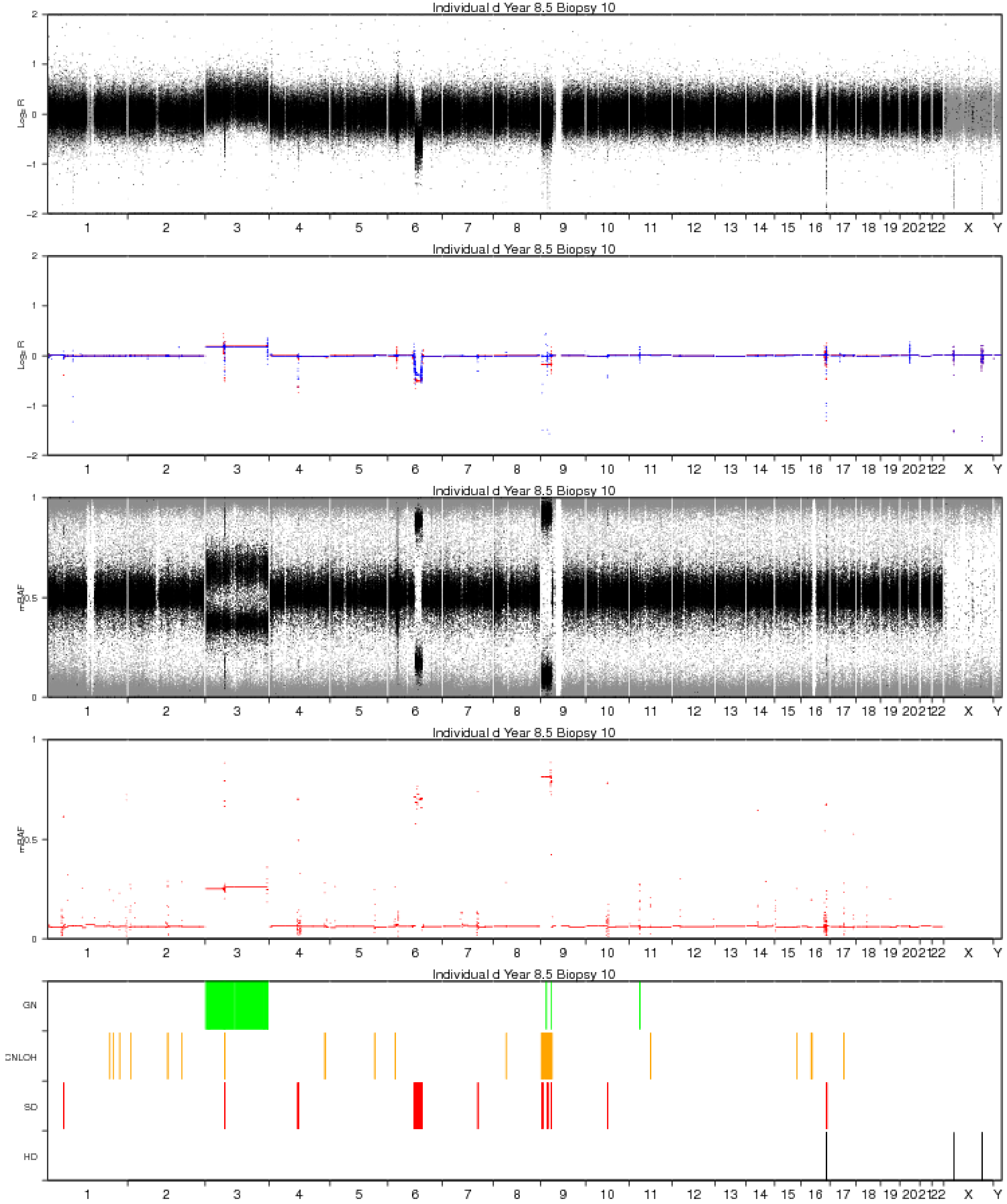
Individual d Year 8.5 Biopsy 10
Log R
mBAF
GN
CNLOH
SD
HD
1 2 3 4 5 6 7 8 9 10 11 12 13 14 15 16 17 18 19 20 21 22 X Y

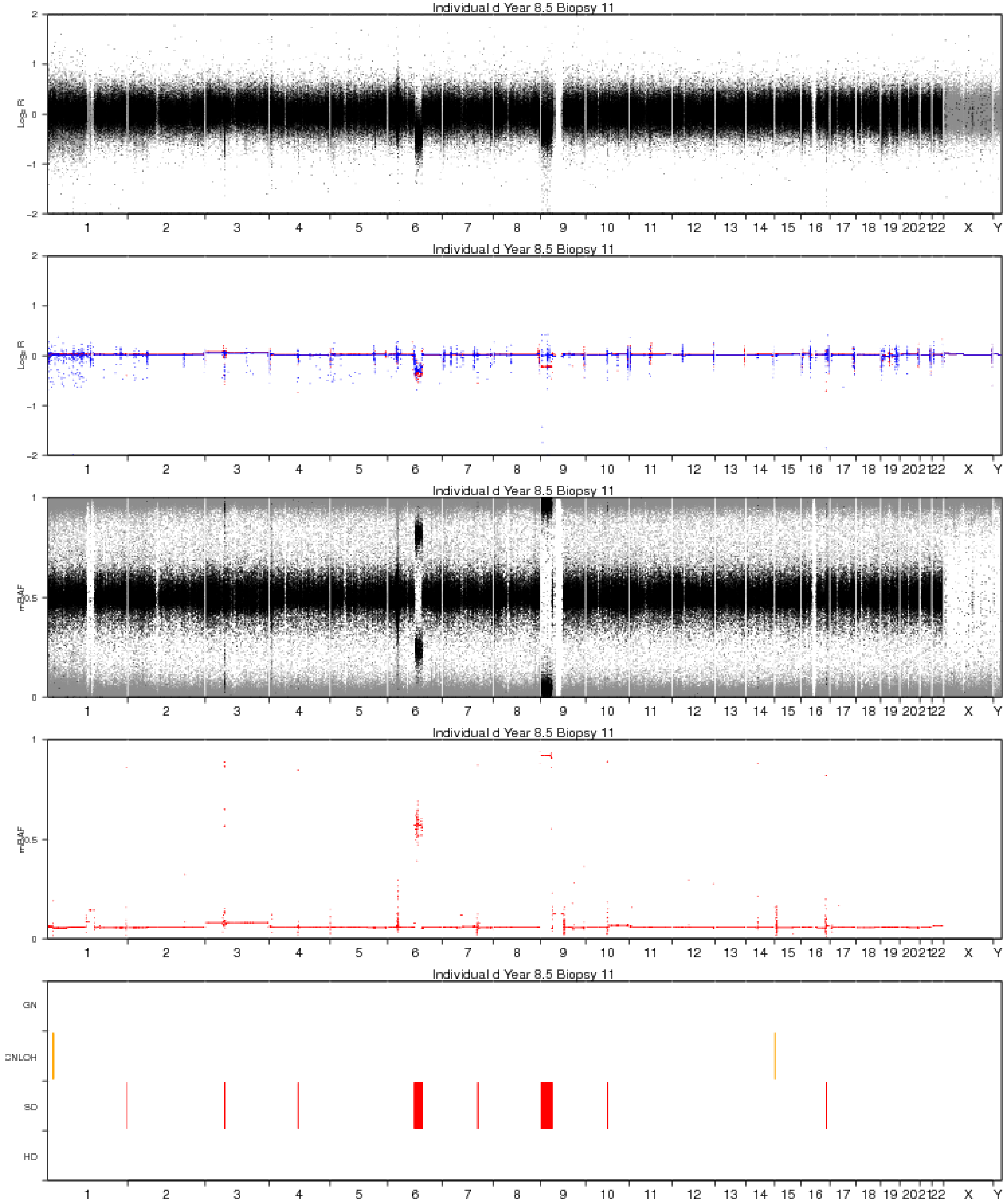
Individual d Year 8.5 Biopsy 11
Log R
2
1
0
−1
−2
1 2 3 4 5 6 7 8 9 10 11 12 13 14 15 16 17 18 19 20 21 22 X Y
Individual d Year 8.5 Biopsy 11
Log R
Individual d Year 8.5 Biopsy 11
mBAF
1
0.5
0
Individual d Year 8.5 Biopsy 11
mBAF
Individual d Year 8.5 Biopsy 11
GN
CNLOH
SD
HD

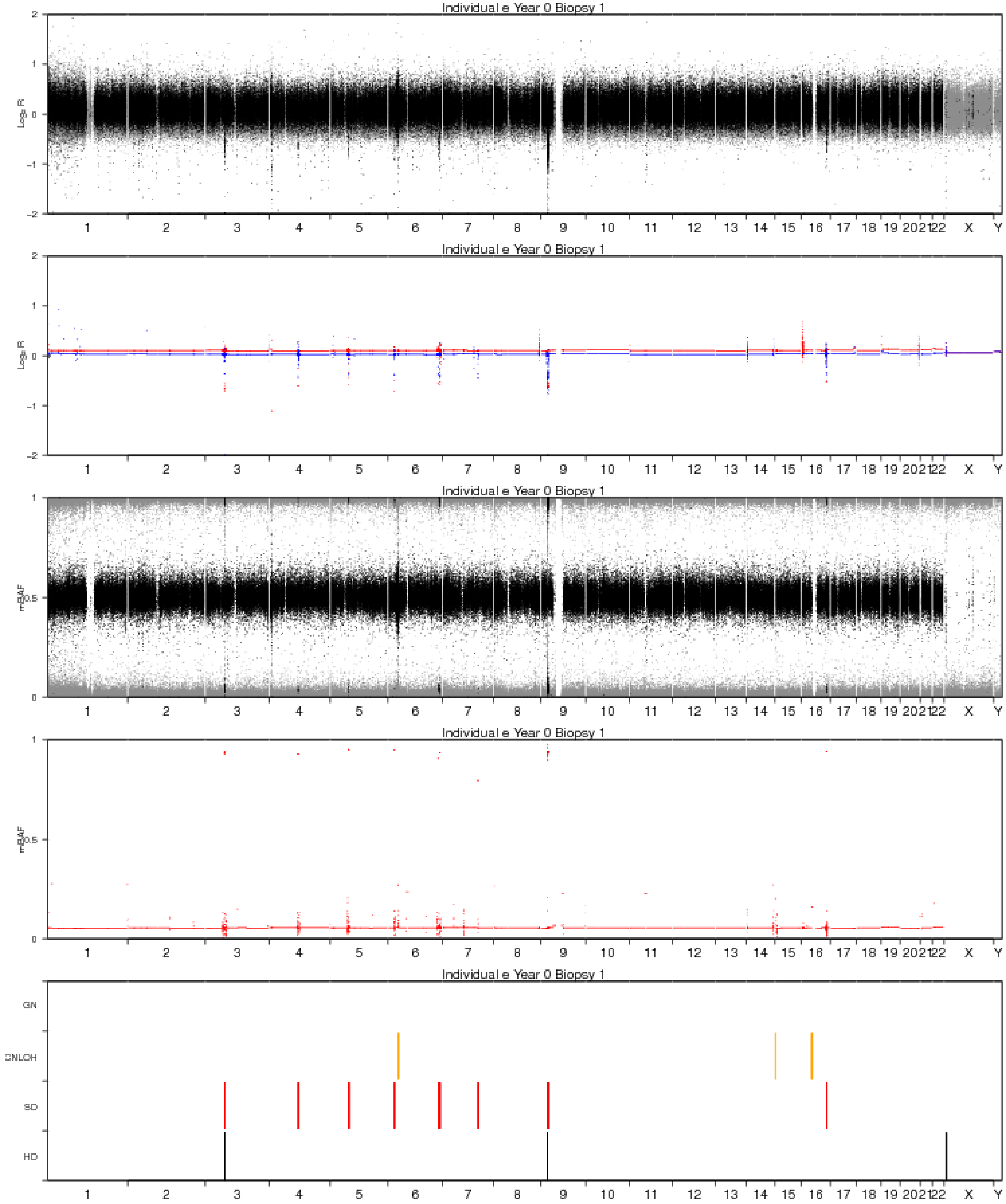
Individual e Year 0 Biopsy 1
Log R
mBAF
GN
CNLOH
SD
HD
1
2
3
4
5
6
7
8
9
10
11
12
13
14
15
16
17
18
19
20
21
22
X
Y

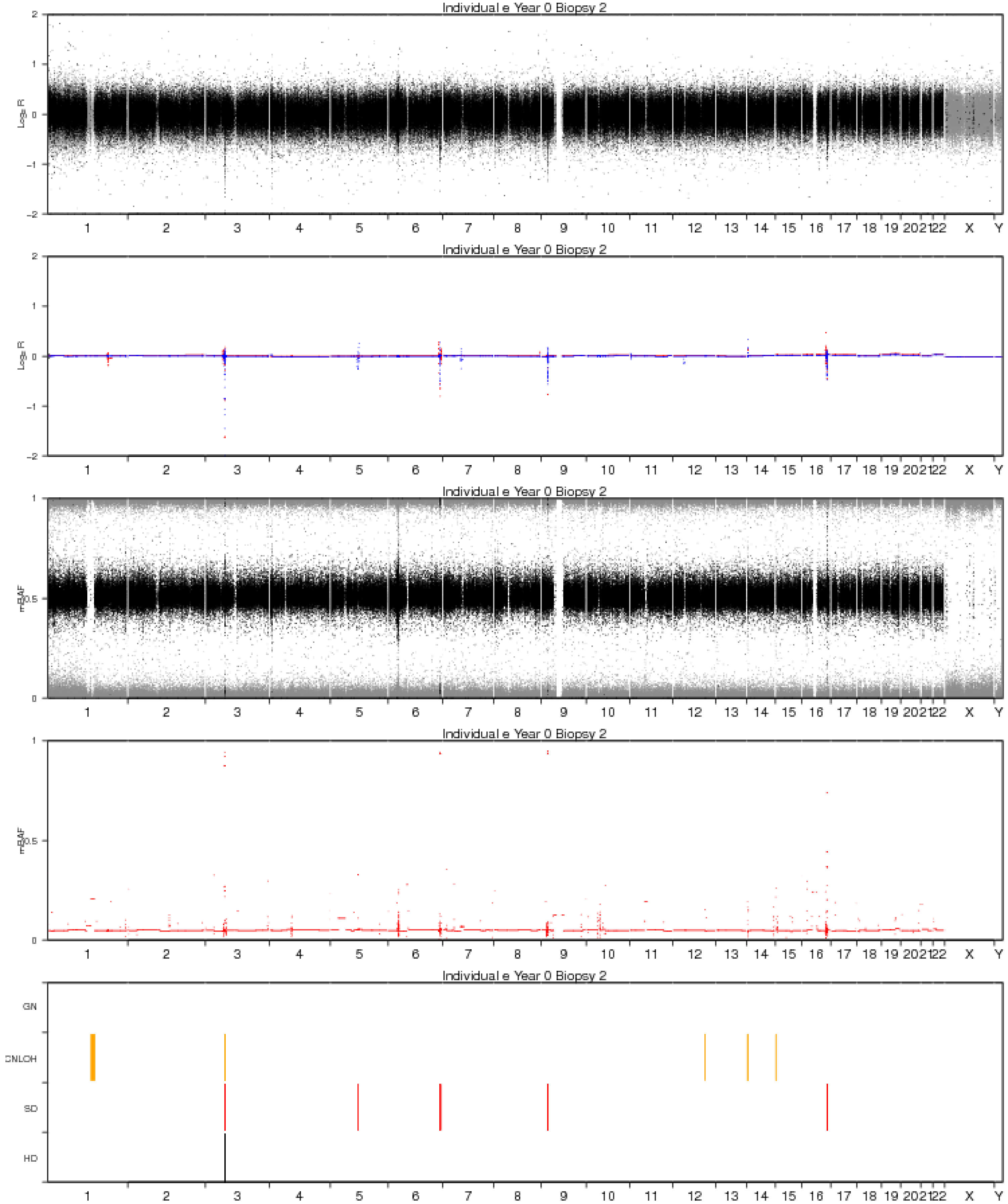
Individual e Year 0 Biopsy 2
Log R
mBAF
GN
CNLOH
SD
HD
1 2 3 4 5 6 7 8 9 10 11 12 13 14 15 16 17 18 19 20 21 22 X Y

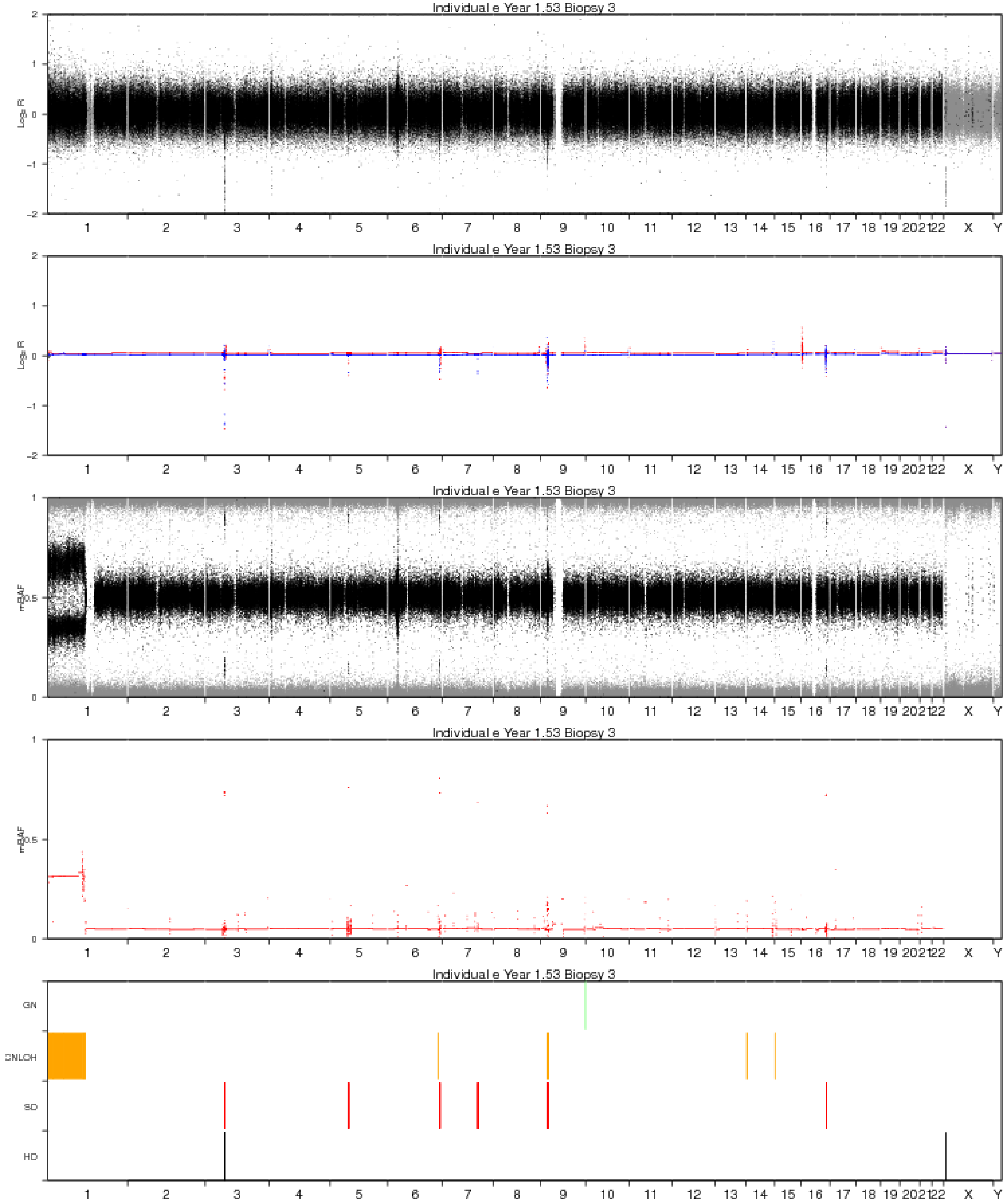
Individual e Year 1.53 Biopsy 3
Log R
2
1
0
−1
−2
1 2 3 4 5 6 7 8 9 10 11 12 13 14 15 16 17 18 19 20 21 22 X Y
Individual e Year 1.53 Biopsy 3
Log R
Individual e Year 1.53 Biopsy 3
mBAF
1
0.5
0
Individual e Year 1.53 Biopsy 3
mBAF
Individual e Year 1.53 Biopsy 3
GN
CNLOH
SD
HD

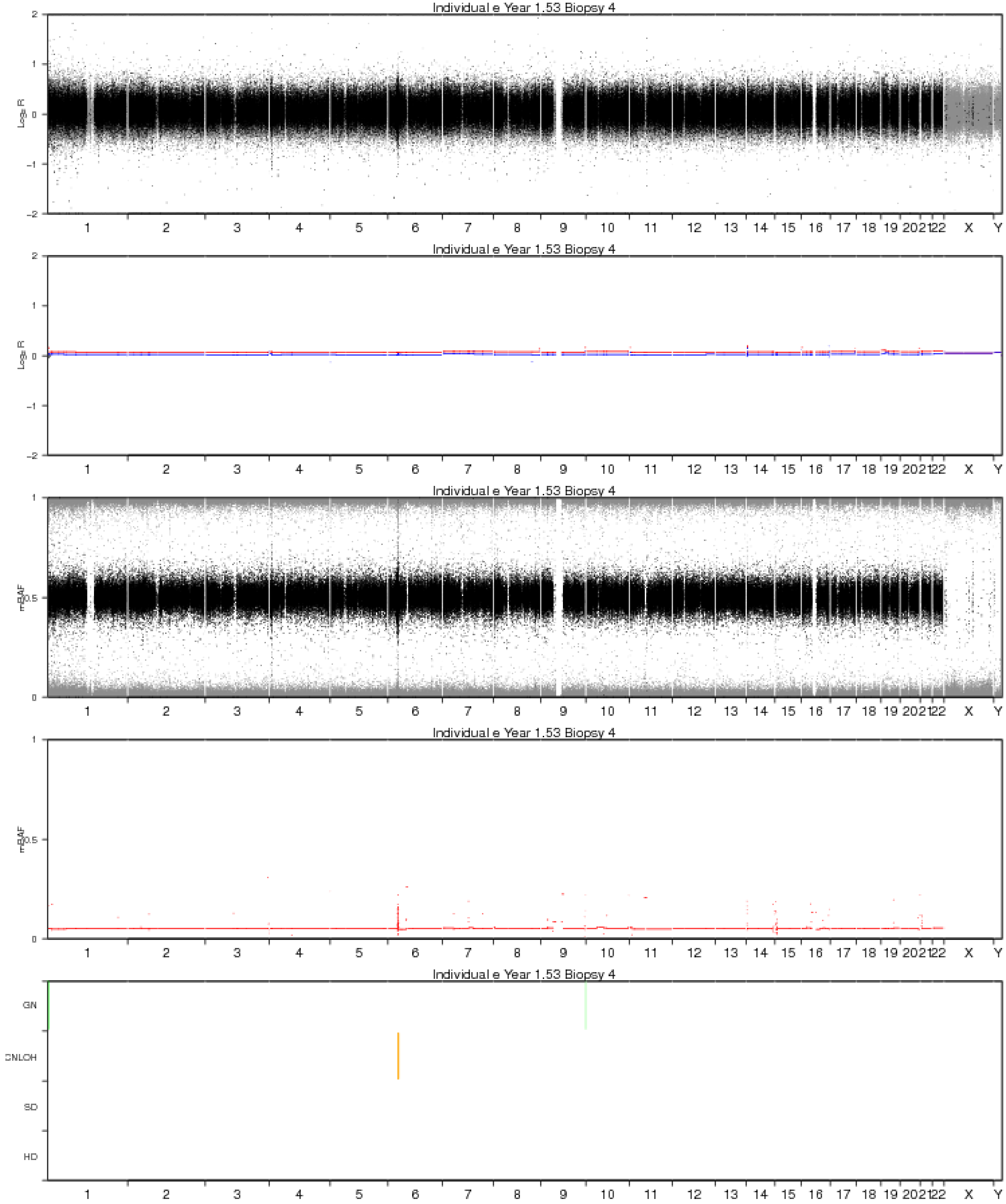

Individual e Year 1.53 Biopsy 4
Log R
mBAF
GN
CNLOH
SD
HD
1
2
3
4
5
6
7
8
9
10
11
12
13
14
15
16
17
18
19
20
21
22
X
Y

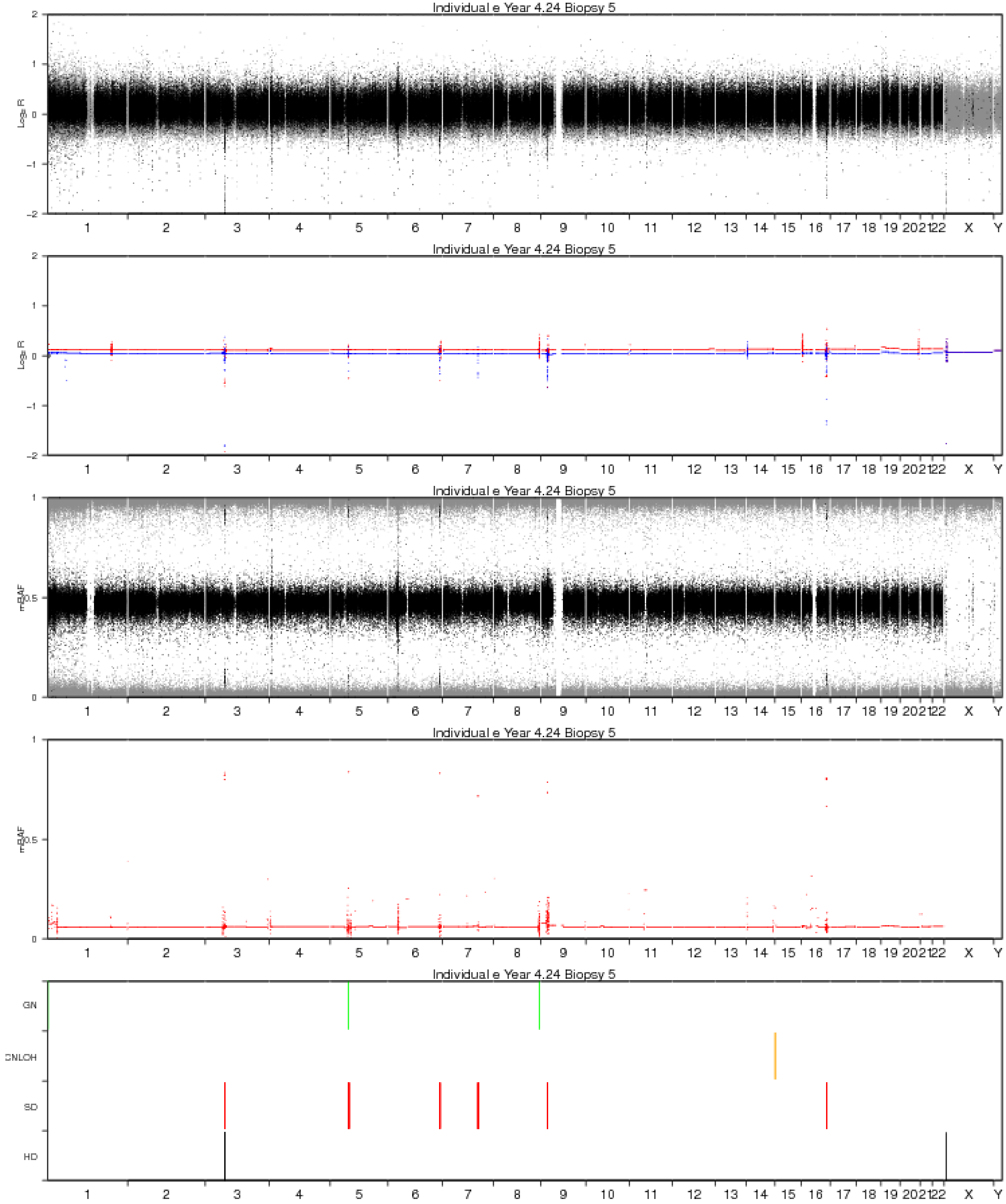

Individual e Year 4.24 Biopsy 5
Log R
mBAF
GN
CNLOH
SD
HD
1 2 3 4 5 6 7 8 9 10 11 12 13 14 15 16 17 18 19 20 21 22 X Y

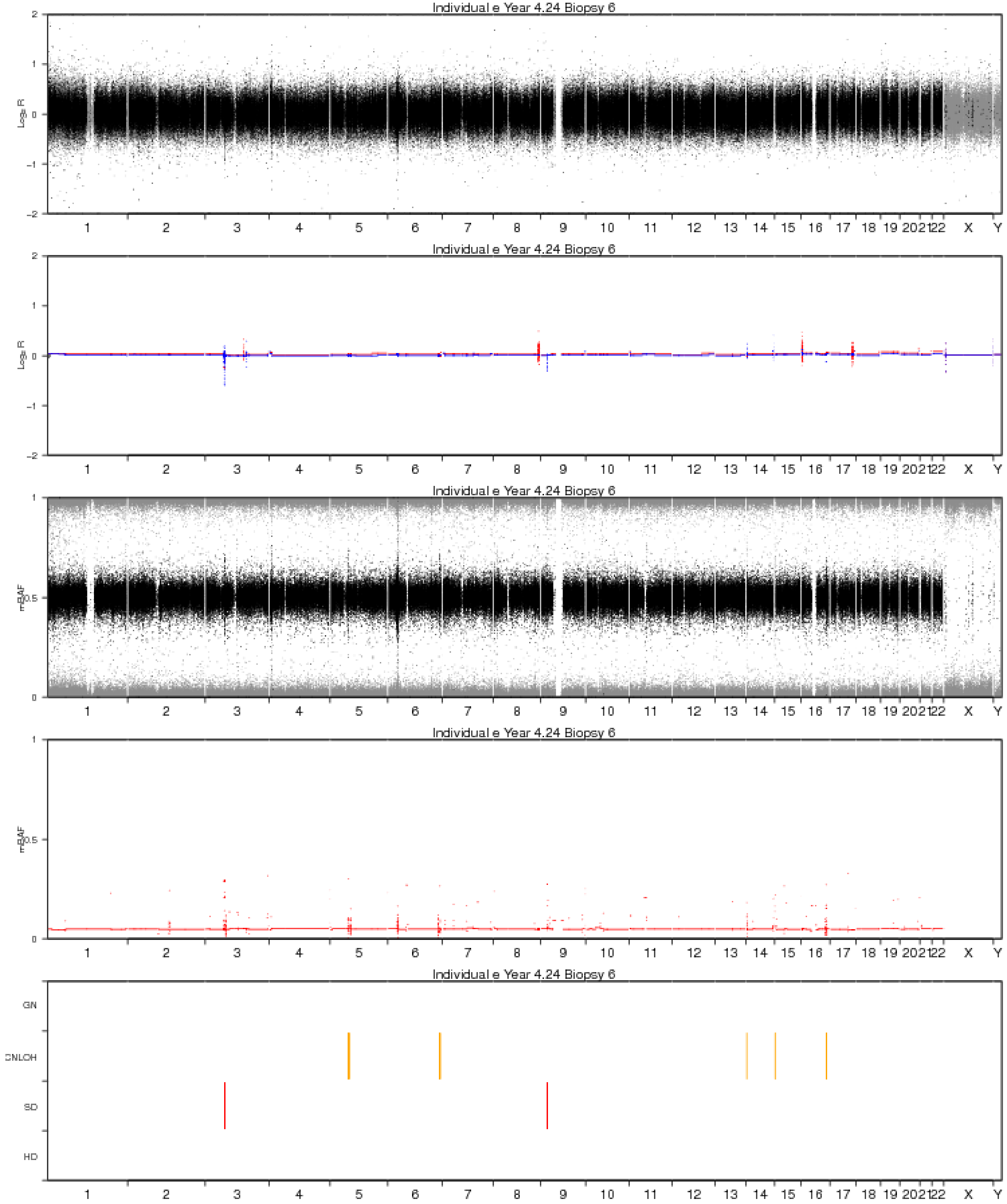
Individual e Year 4.24 Biopsy 6
Log R
mBAF
GN
CNLOH
SD
HD
1
2
3
4
5
6
7
8
9
10
11
12
13
14
15
16
17
18
19
20
21
22
X
Y

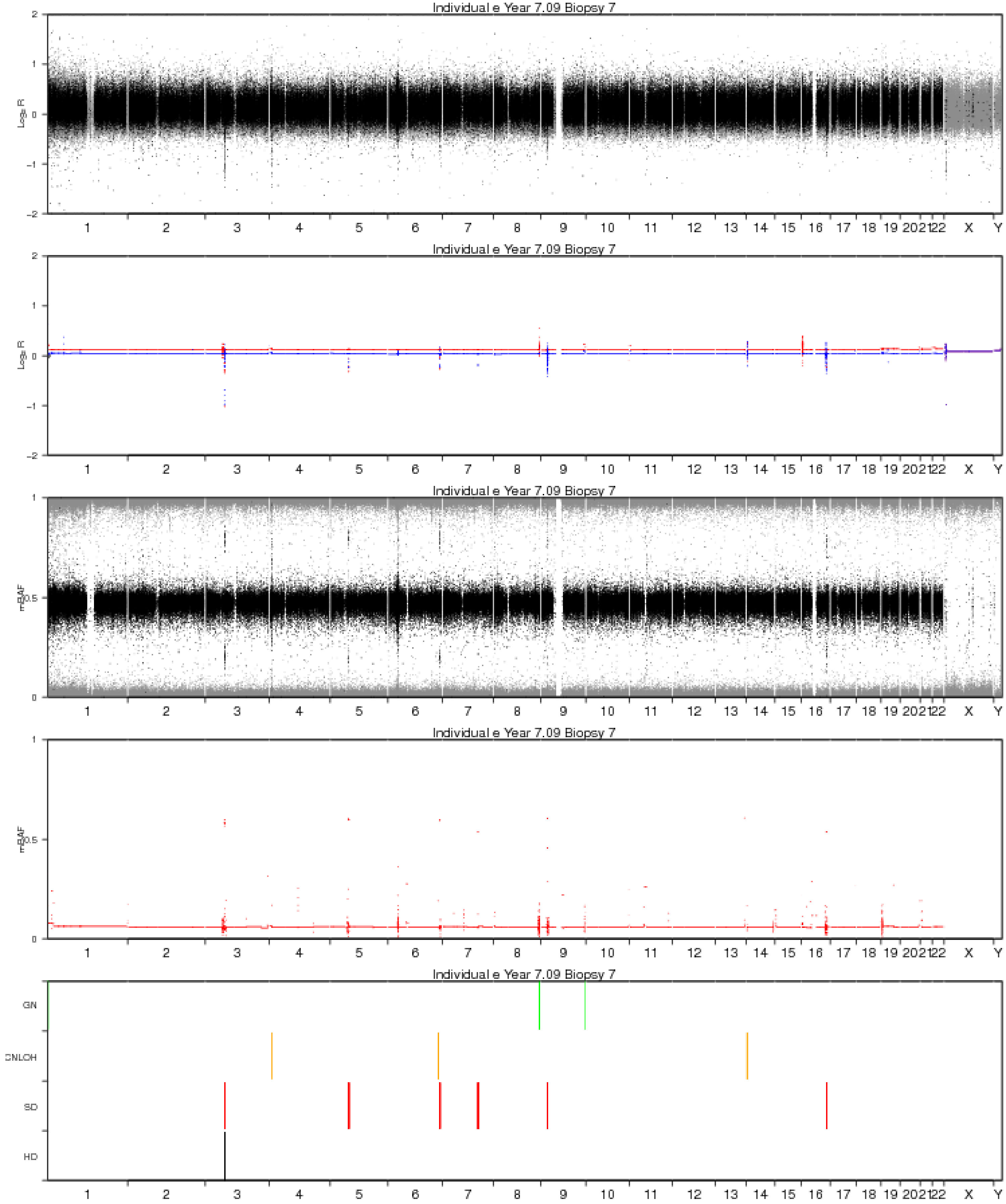
Individual e Year 7.09 Biopsy 7
Log R
mBAF
GN
CNLOH
SD
HD
1 2 3 4 5 6 7 8 9 10 11 12 13 14 15 16 17 18 19 20 21 22 X Y

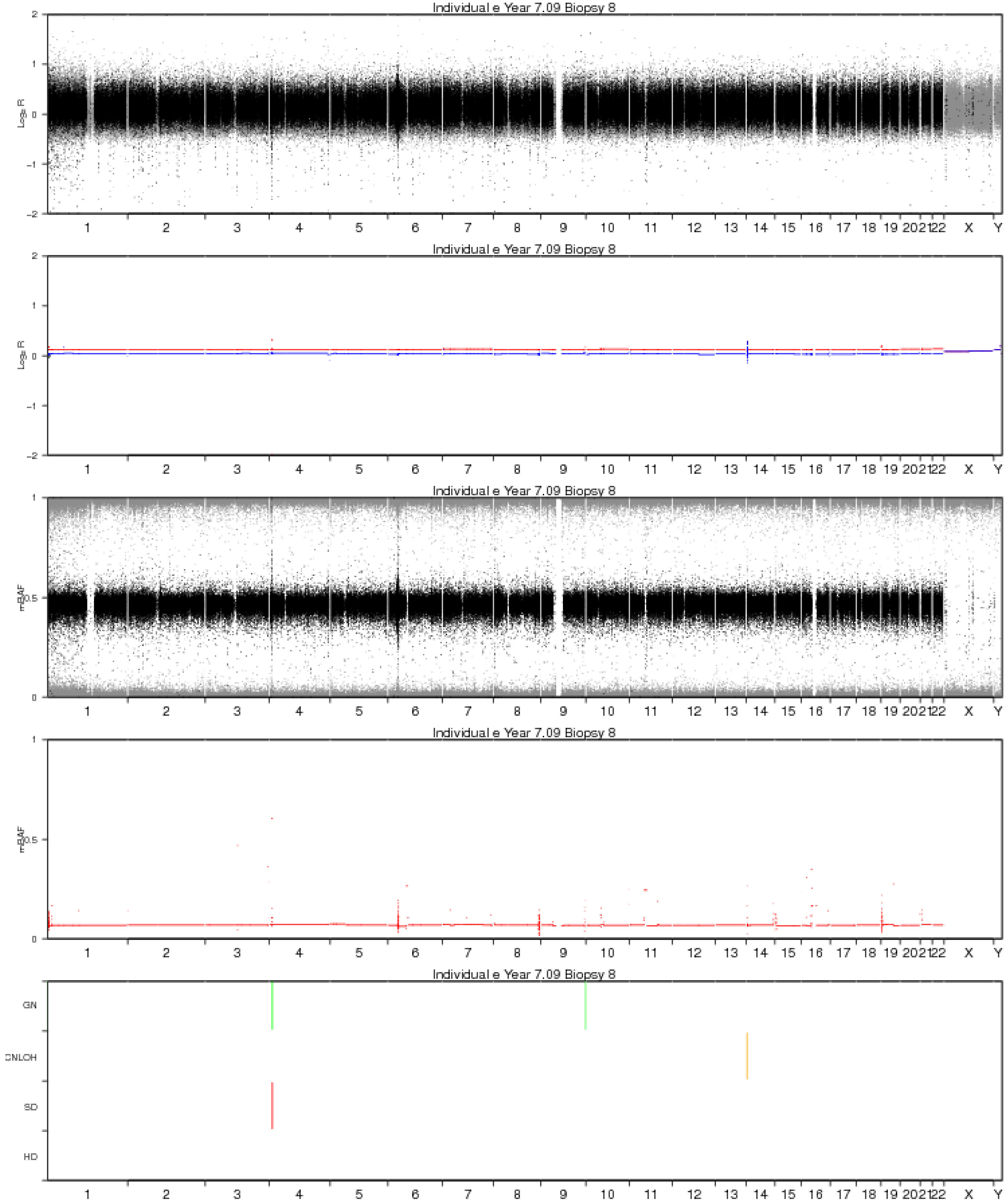
Individual e Year 7.09 Biopsy 8
Log2 R
mBAF
GN
CNLOH
SD
HD
1 2 3 4 5 6 7 8 9 10 11 12 13 14 15 16 17 18 19 20 21 22 X Y

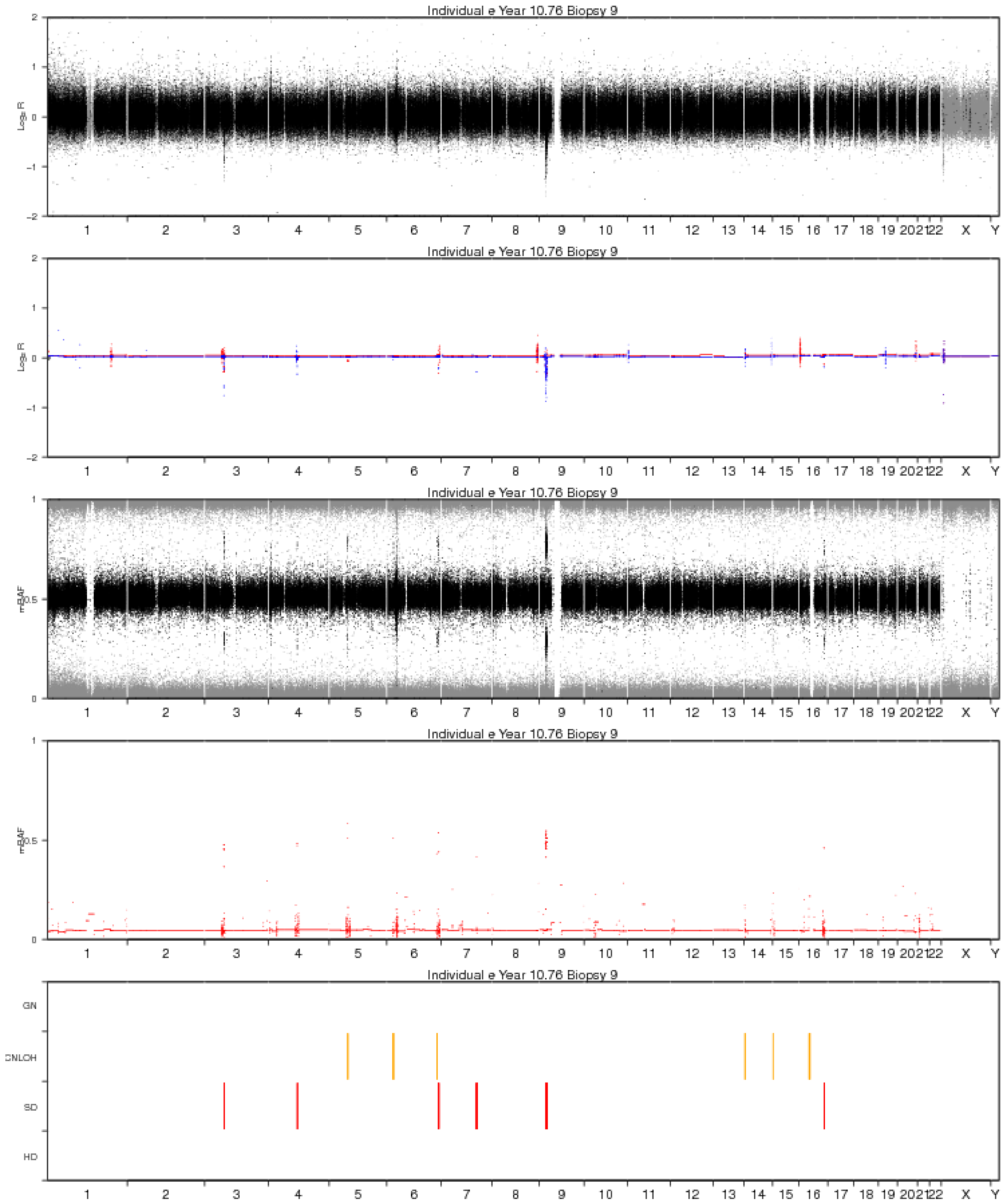
Individual e Year 10.76 Biopsy 9
Log R
mBAF
GN
CNLOH
SD
HD
1
2
3
4
5
6
7
8
9
10
11
12
13
14
15
16
17
18
19
20
21
22
X
Y

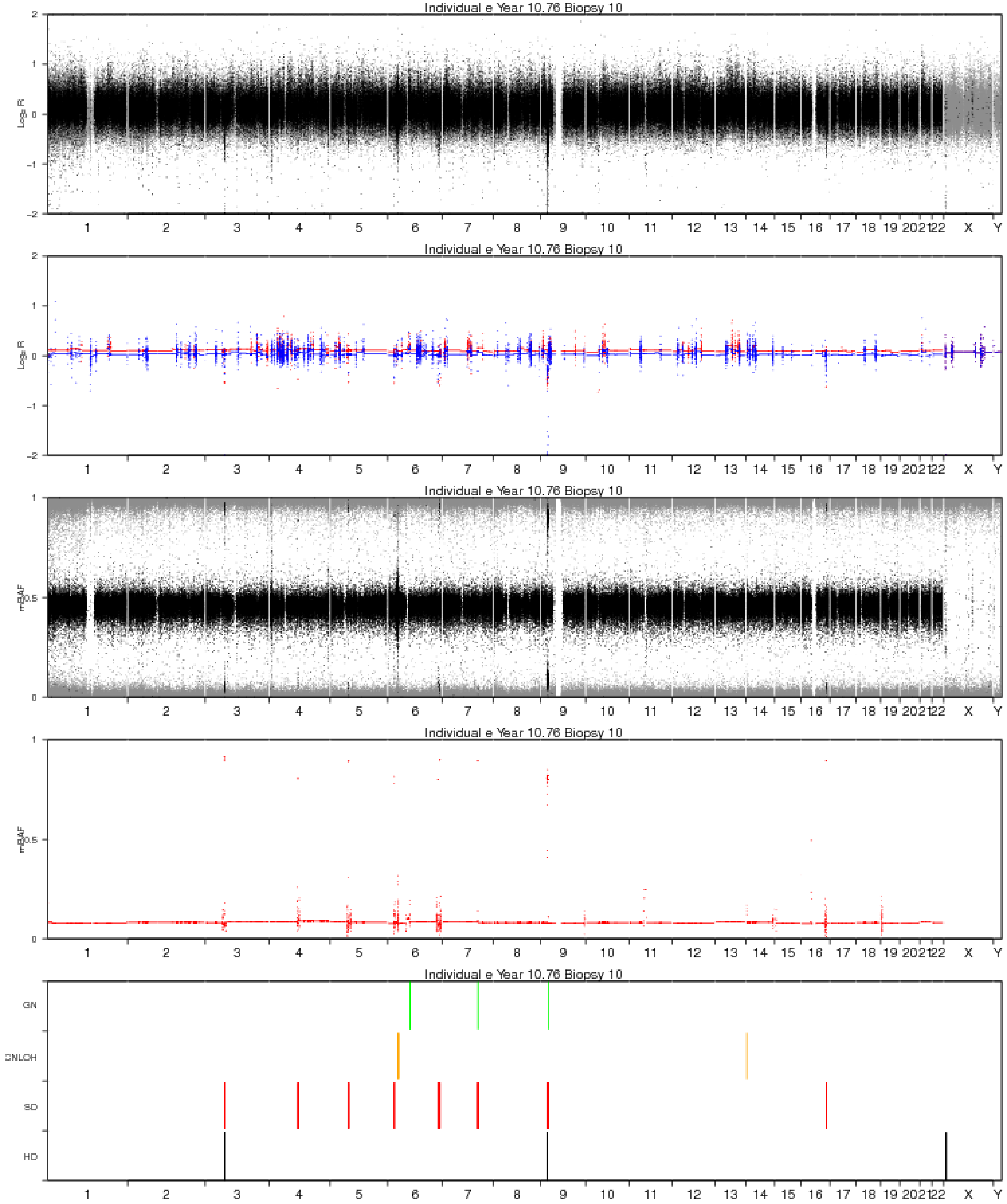
Individual e Year 10.76 Biopsy 10
Log R
mBAF
GN
CNLOH
SD
HD
1 2 3 4 5 6 7 8 9 10 11 12 13 14 15 16 17 18 19 20 21 22 X Y

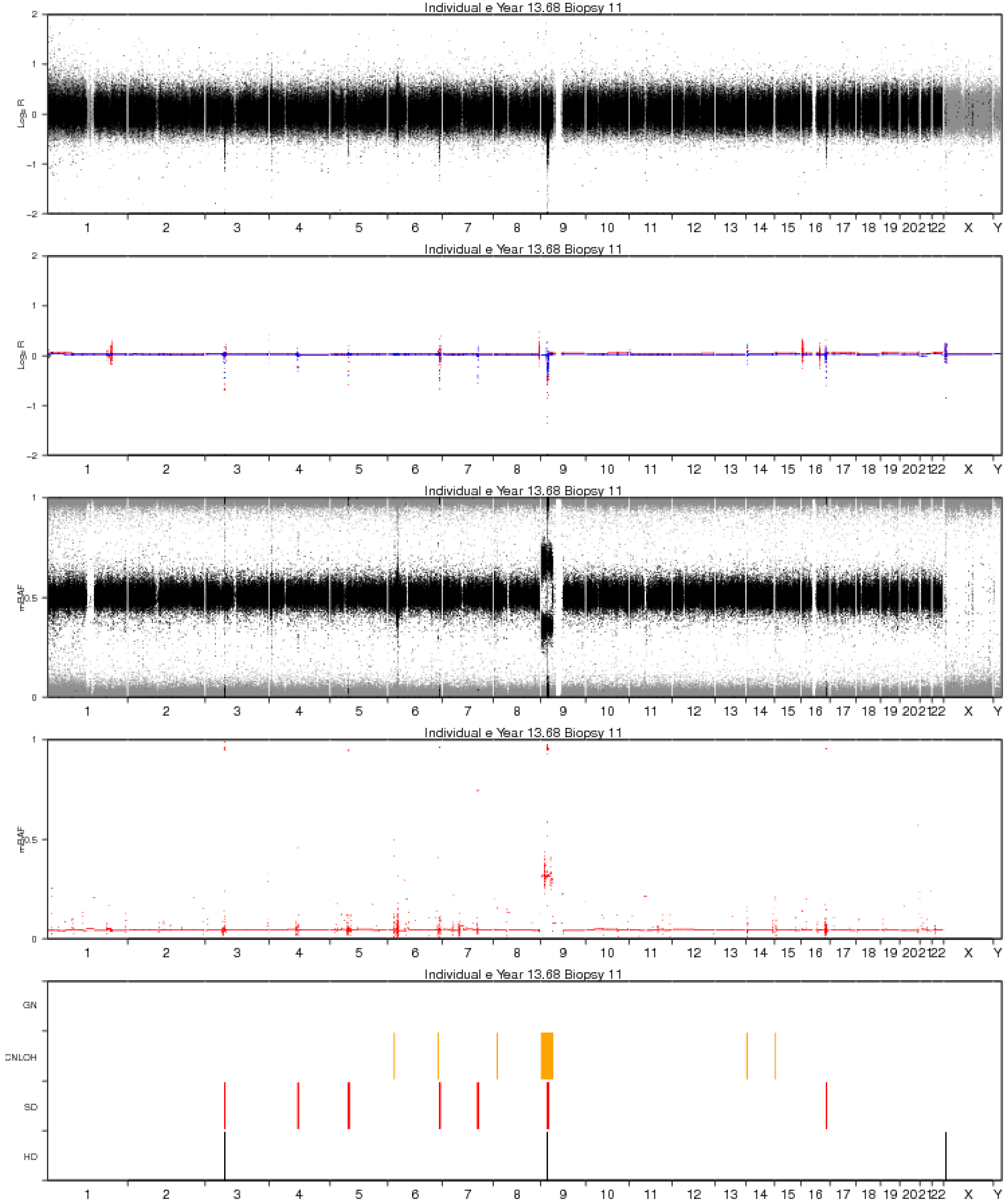

Individual e Year 13.68 Biopsy 11
Log R
mBAF
GN
CNLOH
SD
HD
1 2 3 4 5 6 7 8 9 10 11 12 13 14 15 16 17 18 19 20 21 22 X Y

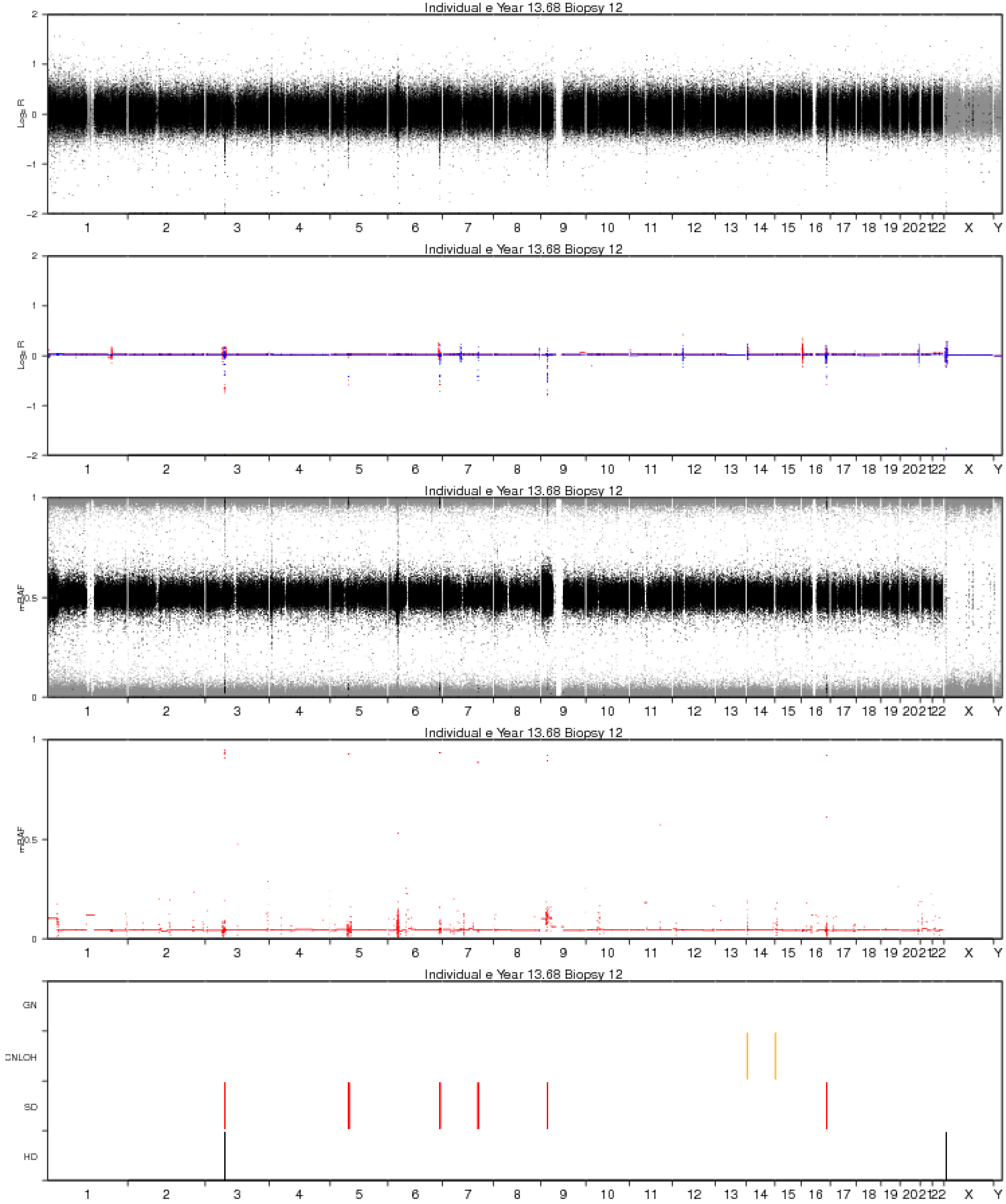

Individual e Year 13.68 Biopsy 12
Log2 R
mBAF
GN
CNLOH
SD
HD
1 2 3 4 5 6 7 8 9 10 11 12 13 14 15 16 17 18 19 20 21 22 X Y

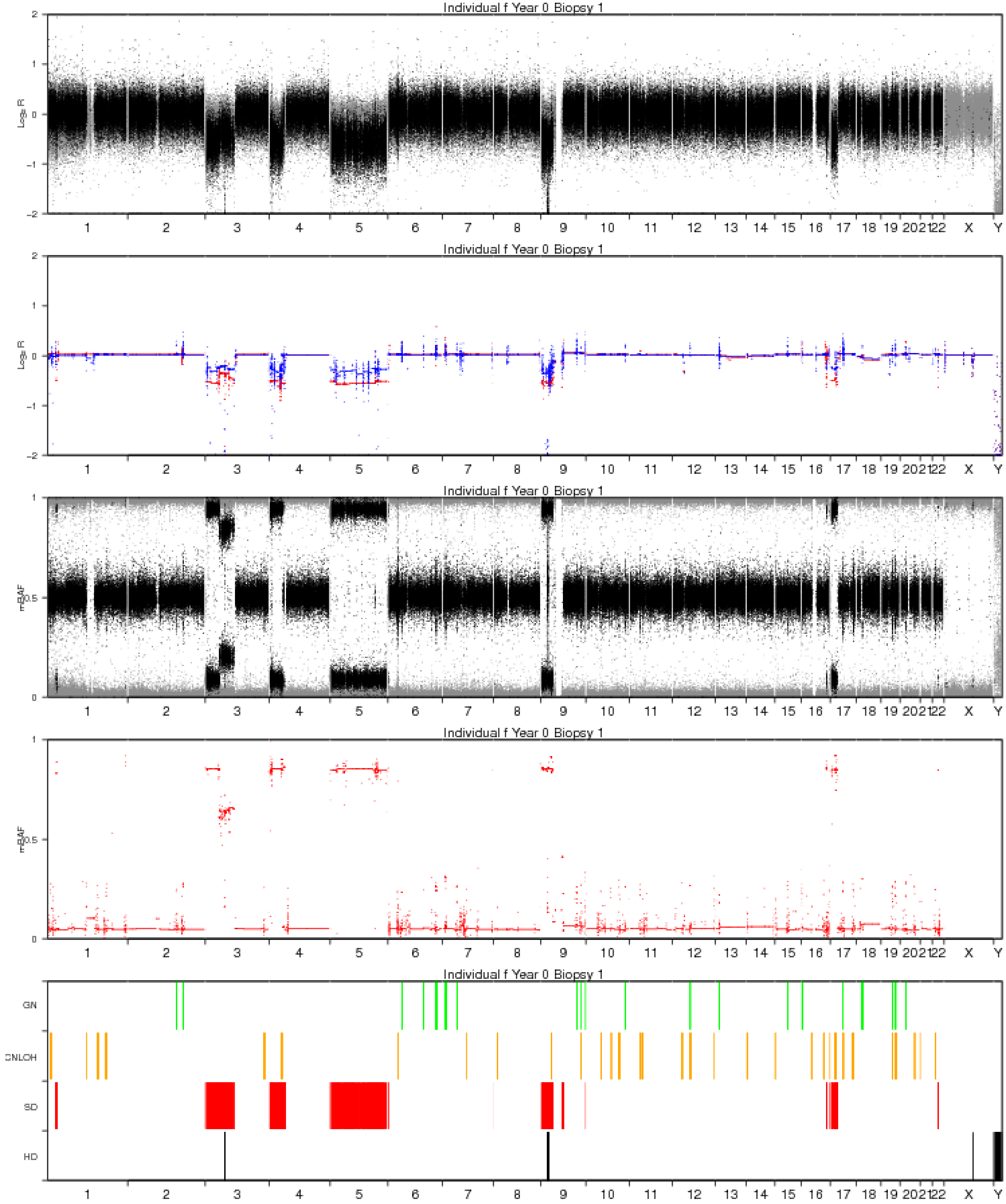

Individual f Year 0 Biopsy 1
Log R
mBAF
GN
CNLOH
SD
HD
1 2 3 4 5 6 7 8 9 10 11 12 13 14 15 16 17 18 19 20 21 22 X Y

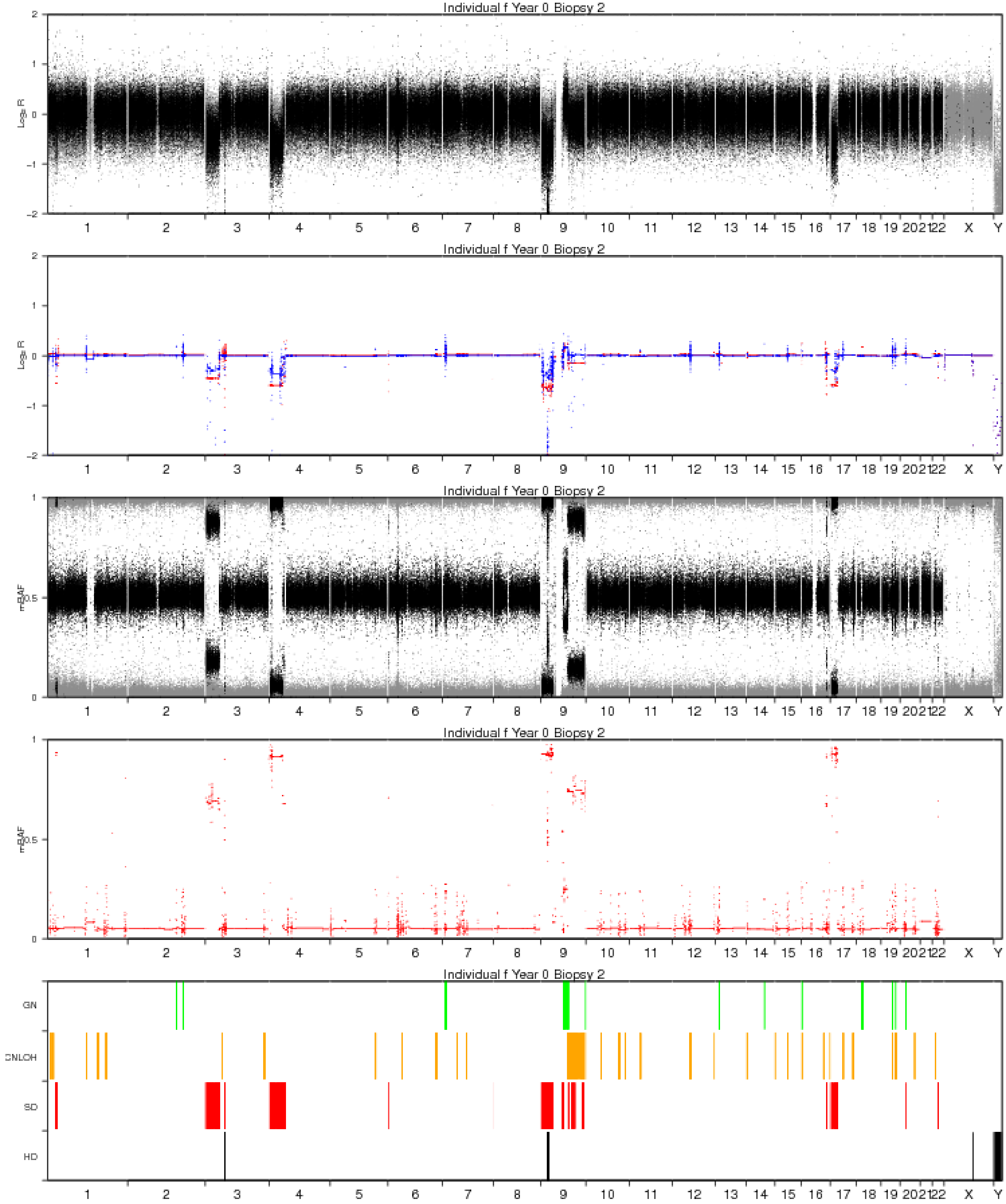
Individual f Year 0 Biopsy 2
Log R
mBAF
GN
CNLOH
SD
HD
1 2 3 4 5 6 7 8 9 10 11 12 13 14 15 16 17 18 19 20 21 22 X Y

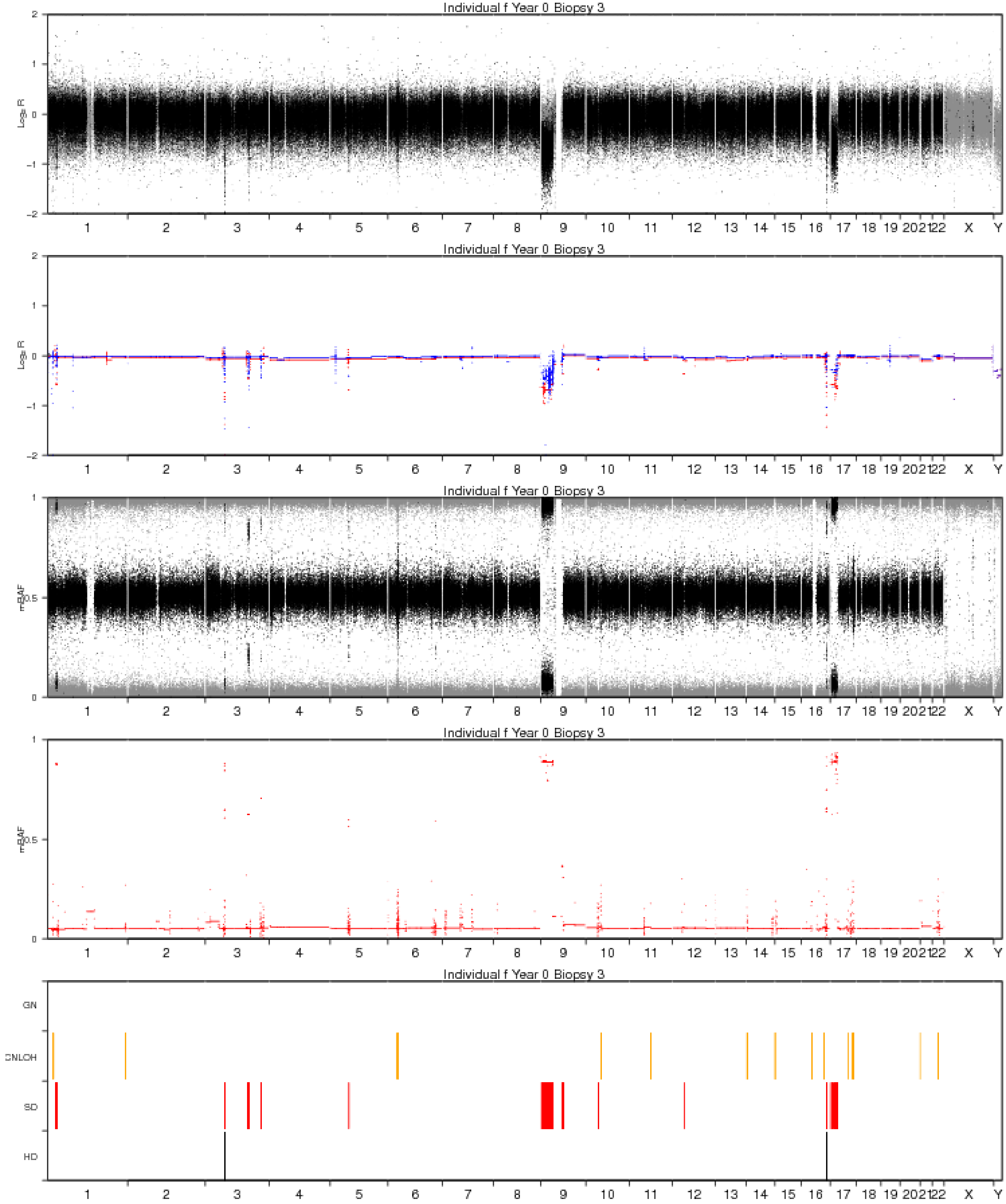
Individual f Year 0 Biopsy 3
Log R
mBAF
GN
CNLOH
SD
HD
1 2 3 4 5 6 7 8 9 10 11 12 13 14 15 16 17 18 19 20 21 22 X Y

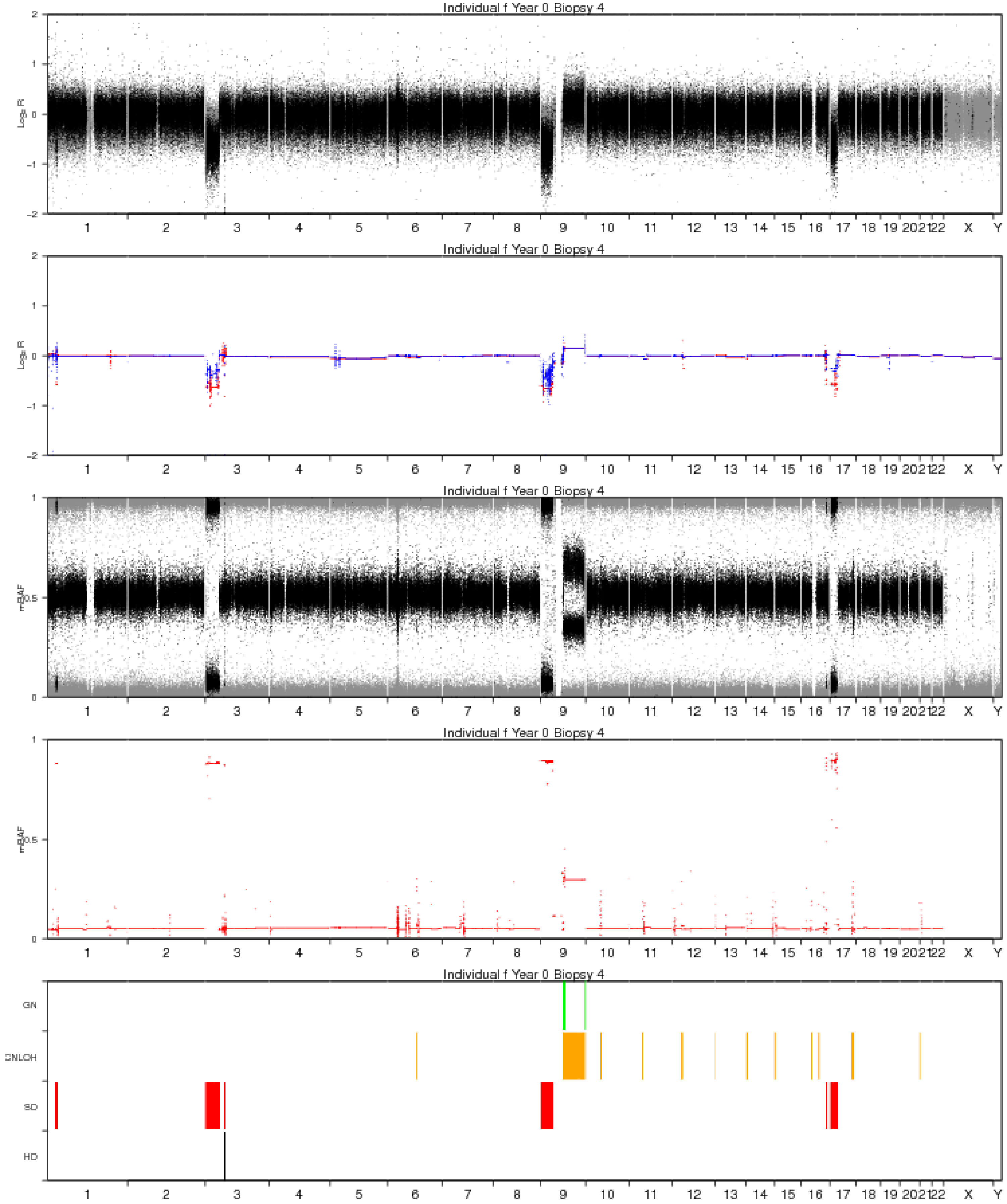
Individual f Year 0 Biopsy 4
Log2 R
mBAF
GN
CNLOH
SD
HD
1 2 3 4 5 6 7 8 9 10 11 12 13 14 15 16 17 18 19 20 21 22 X Y

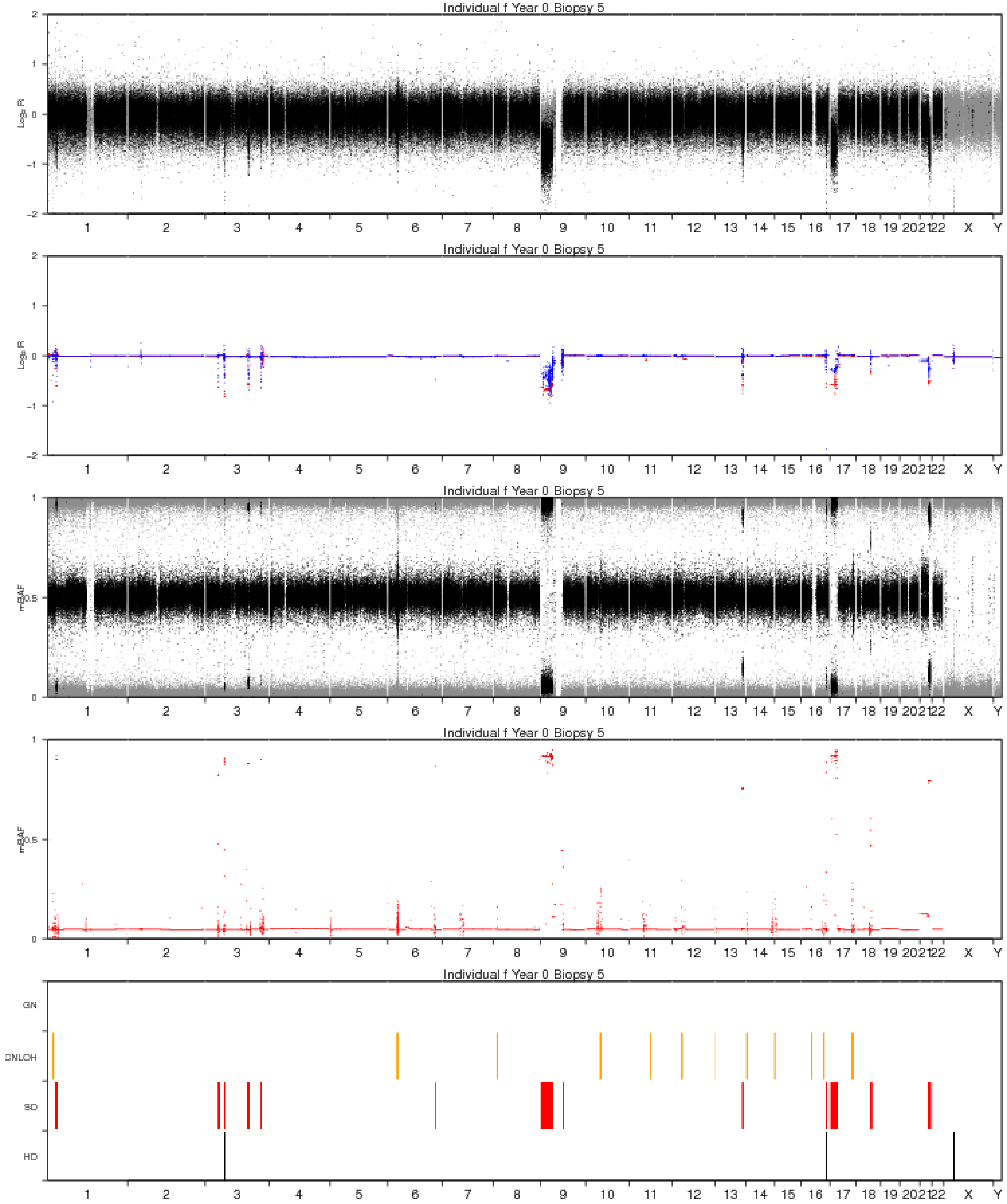
Individual f Year 0 Biopsy 5
Log R
mBAF
GN
CNLOH
SD
HD
1 2 3 4 5 6 7 8 9 10 11 12 13 14 15 16 17 18 19 20 21 22 X Y

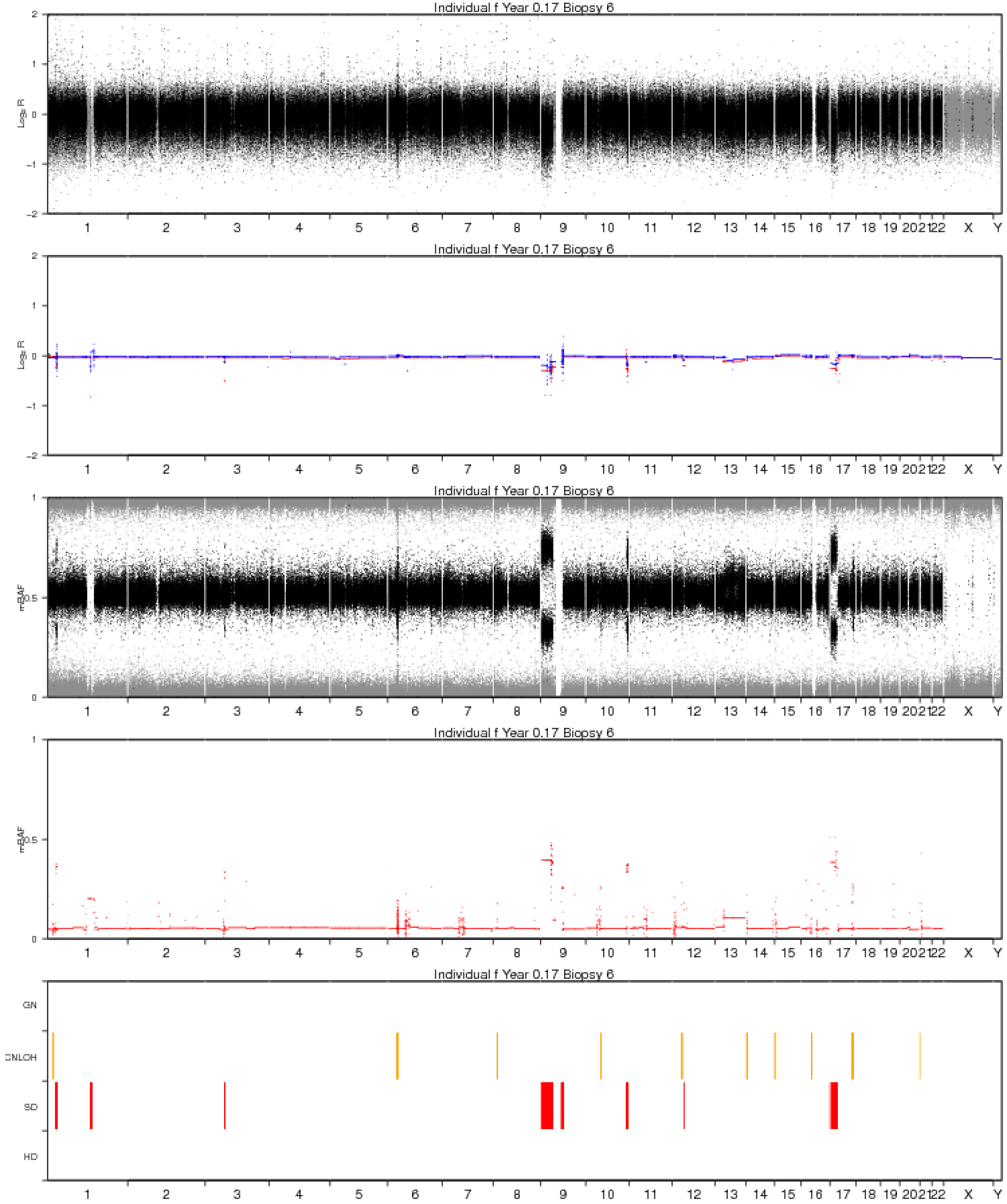
Individual f Year 0.17 Biopsy 6
Log R
mBAF
2
1
0
−1
−2
0.5
1 2 3 4 5 6 7 8 9 10 11 12 13 14 15 16 17 18 19 20 21 22 X Y
GN
CNLOH
SD
HD

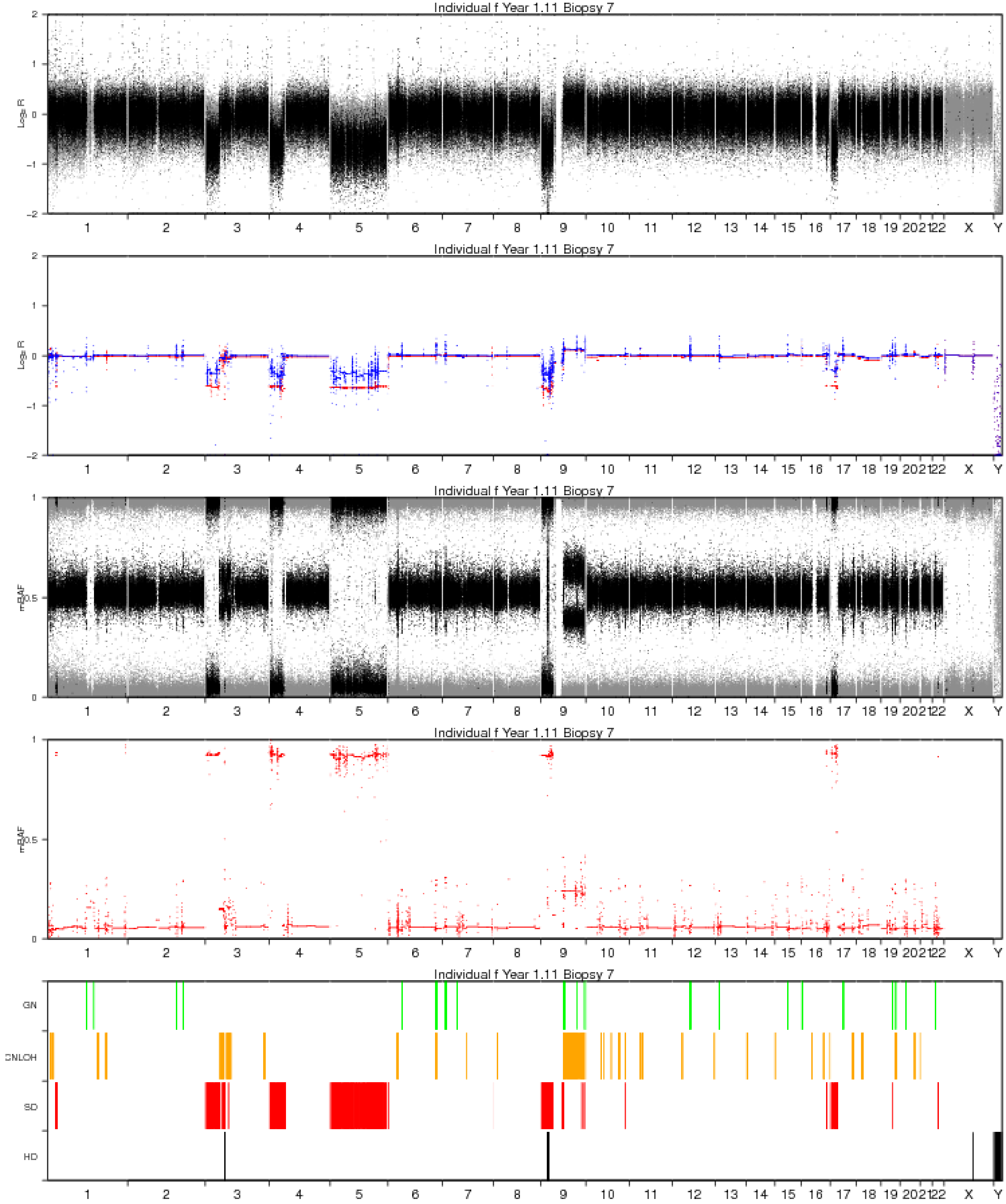
Individual f Year 1.11 Biopsy 7
Log R
mBAF
GN
CNLOH
SD
HD
1 2 3 4 5 6 7 8 9 10 11 12 13 14 15 16 17 18 19 20 21 22 X Y

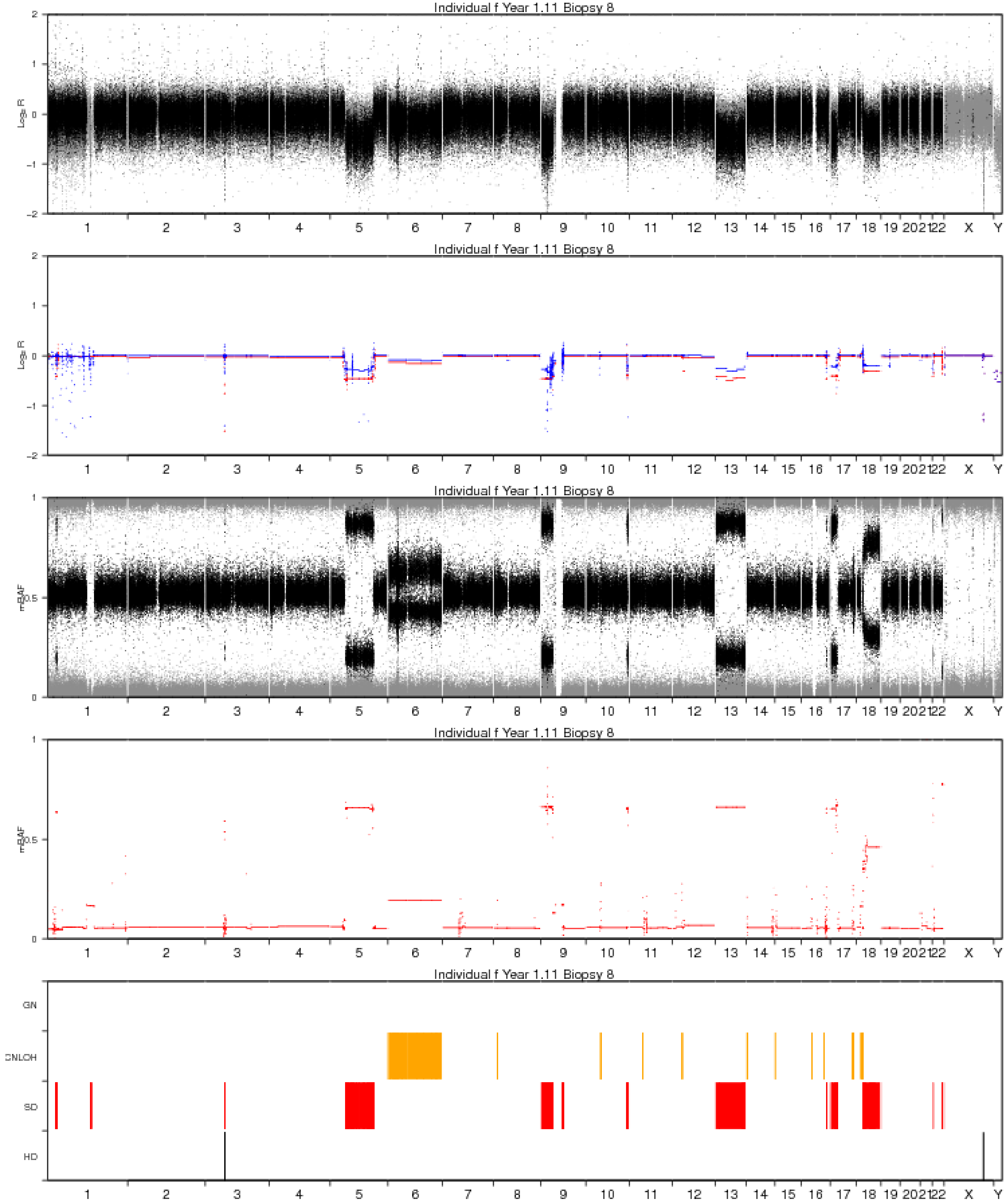

Individual f Year 1.11 Biopsy 8
Log R
mBAF
GN
CNLOH
SD
HD
1 2 3 4 5 6 7 8 9 10 11 12 13 14 15 16 17 18 19 20 21 22 X Y

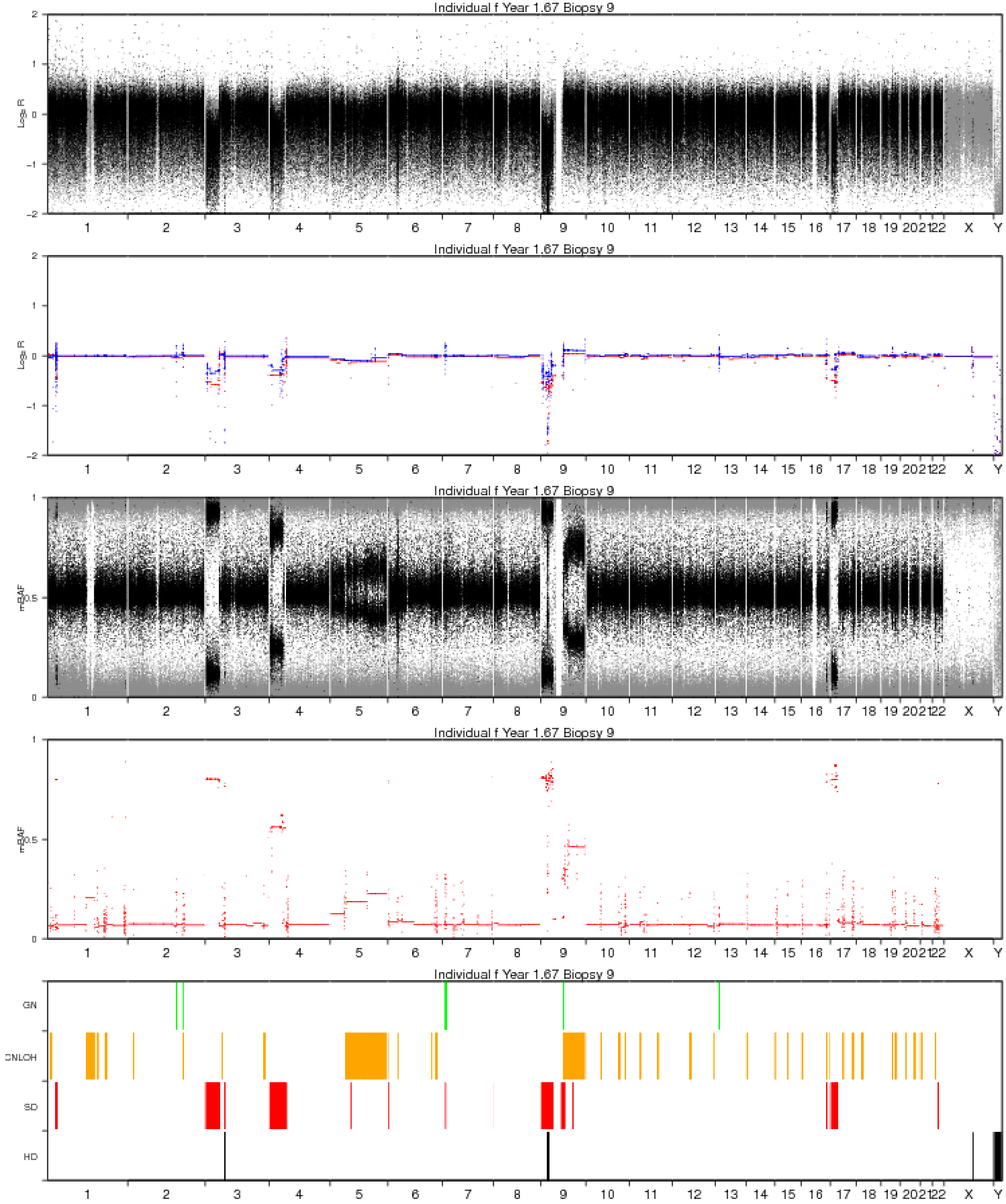
Individual f Year 1.67 Biopsy 9
Log R
mBAF
GN
CNLOH
SD
HD
1 2 3 4 5 6 7 8 9 10 11 12 13 14 15 16 17 18 19 20 21 22 X Y

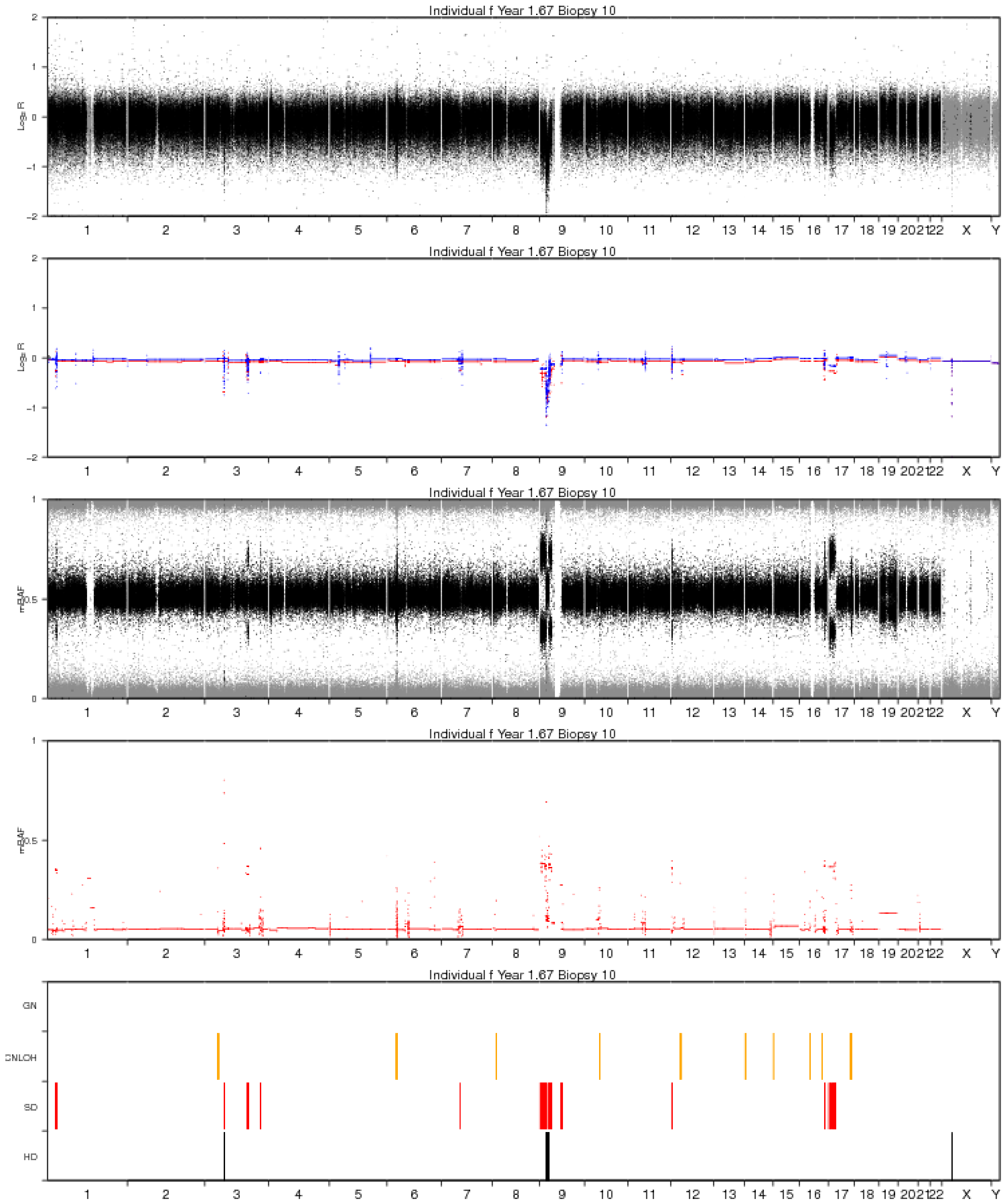
Individual f Year 1.67 Biopsy 10
Log R
2
1
0
−1
−2
1 2 3 4 5 6 7 8 9 10 11 12 13 14 15 16 17 18 19 20 21 22 X Y
Individual f Year 1.67 Biopsy 10
Log R
2
1
0
−1
−2
1 2 3 4 5 6 7 8 9 10 11 12 13 14 15 16 17 18 19 20 21 22 X Y
Individual f Year 1.67 Biopsy 10
mBAF
1
0.5
0
1 2 3 4 5 6 7 8 9 10 11 12 13 14 15 16 17 18 19 20 21 22 X Y
Individual f Year 1.67 Biopsy 10
mBAF
1
0.5
0
1 2 3 4 5 6 7 8 9 10 11 12 13 14 15 16 17 18 19 20 21 22 X Y
Individual f Year 1.67 Biopsy 10
GN
CNLOH
SD
HD
1 2 3 4 5 6 7 8 9 10 11 12 13 14 15 16 17 18 19 20 21 22 X Y

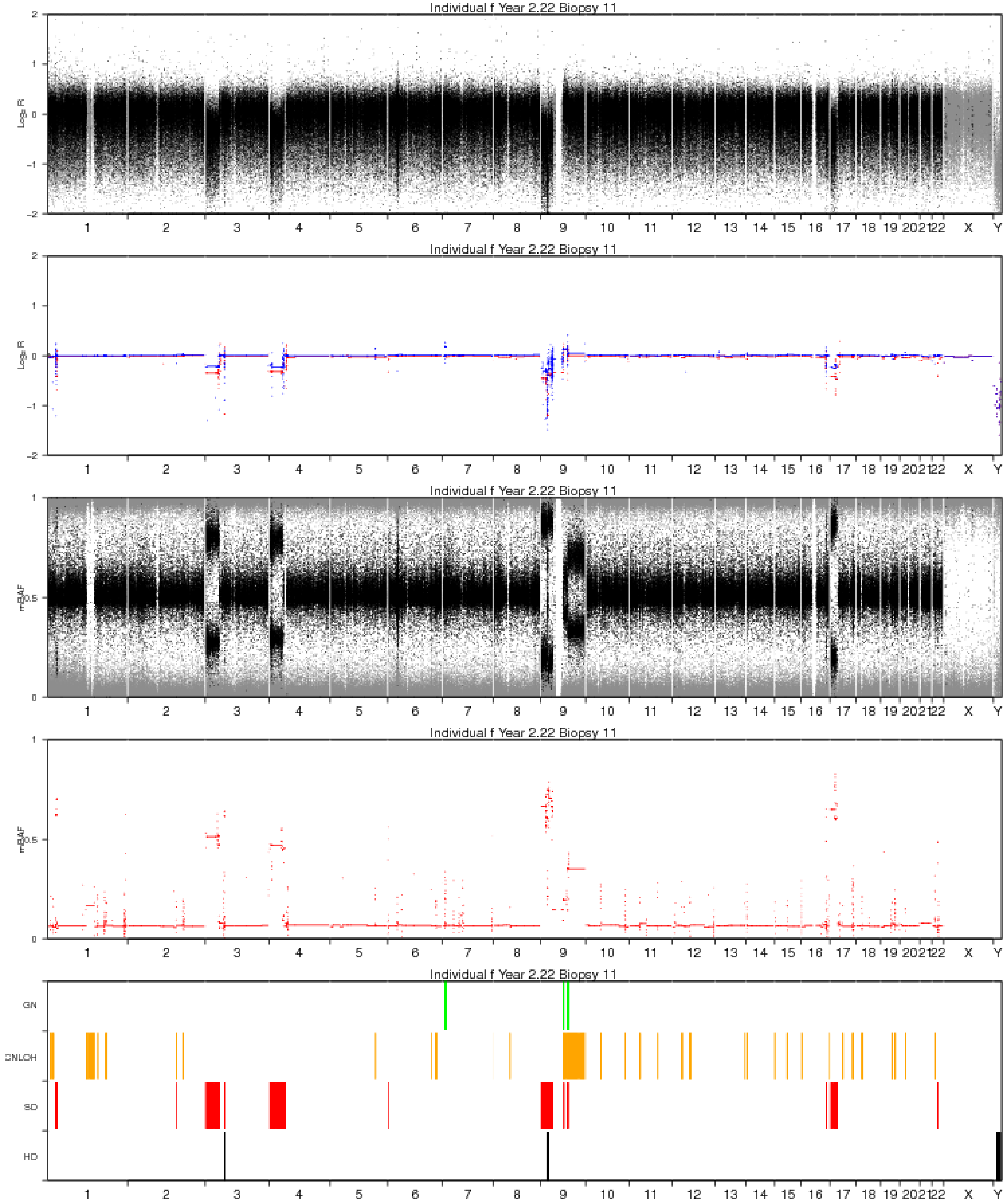
Individual f Year 2.22 Biopsy 11
Log R
1
2
3
4
5
6
7
8
9
10
11
12
13
14
15
16
17
18
19
20
21
22
X
Y
mBAF
GN
CNLOH
SD
HD

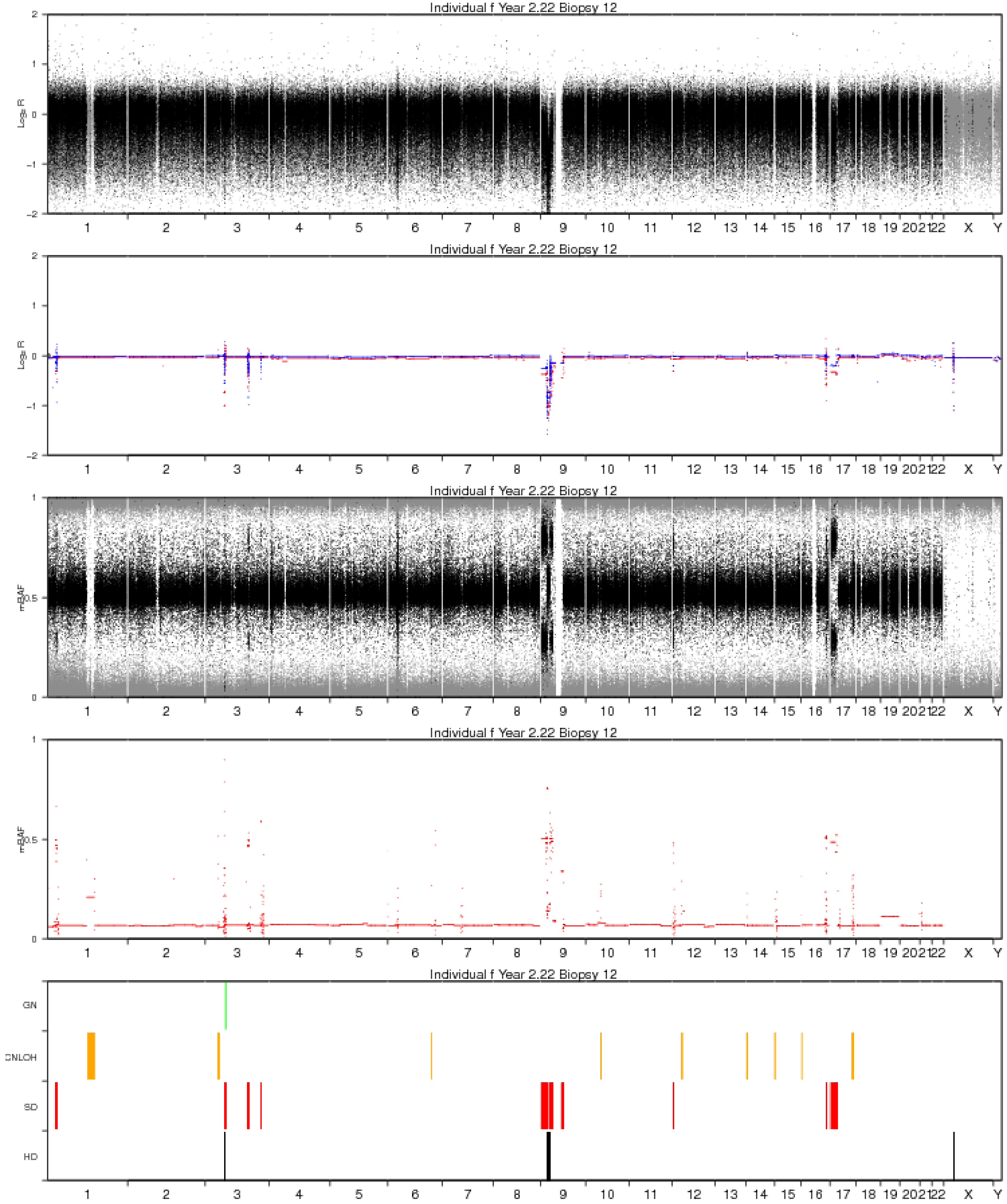
Individual f Year 2.22 Biopsy 12
Log2 R
mBAF
GN
CNLOH
SD
HD
1 2 3 4 5 6 7 8 9 10 11 12 13 14 15 16 17 18 19 20 21 22 X Y

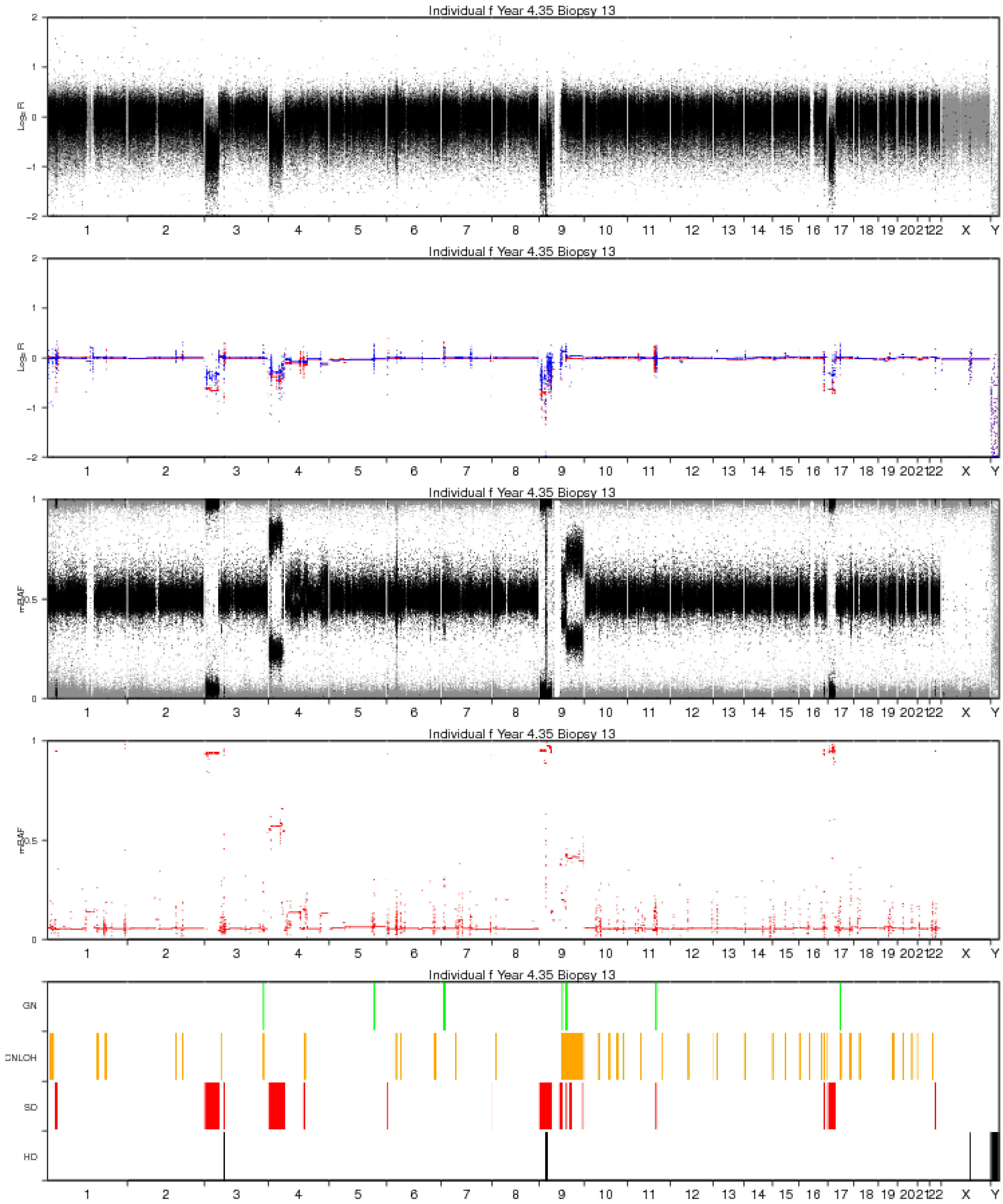

Individual f Year 4.35 Biopsy 13
Log₂ R
mBAF
GN
CNLOH
SD
HD
1 2 3 4 5 6 7 8 9 10 11 12 13 14 15 16 17 18 19 20 21 22 X Y

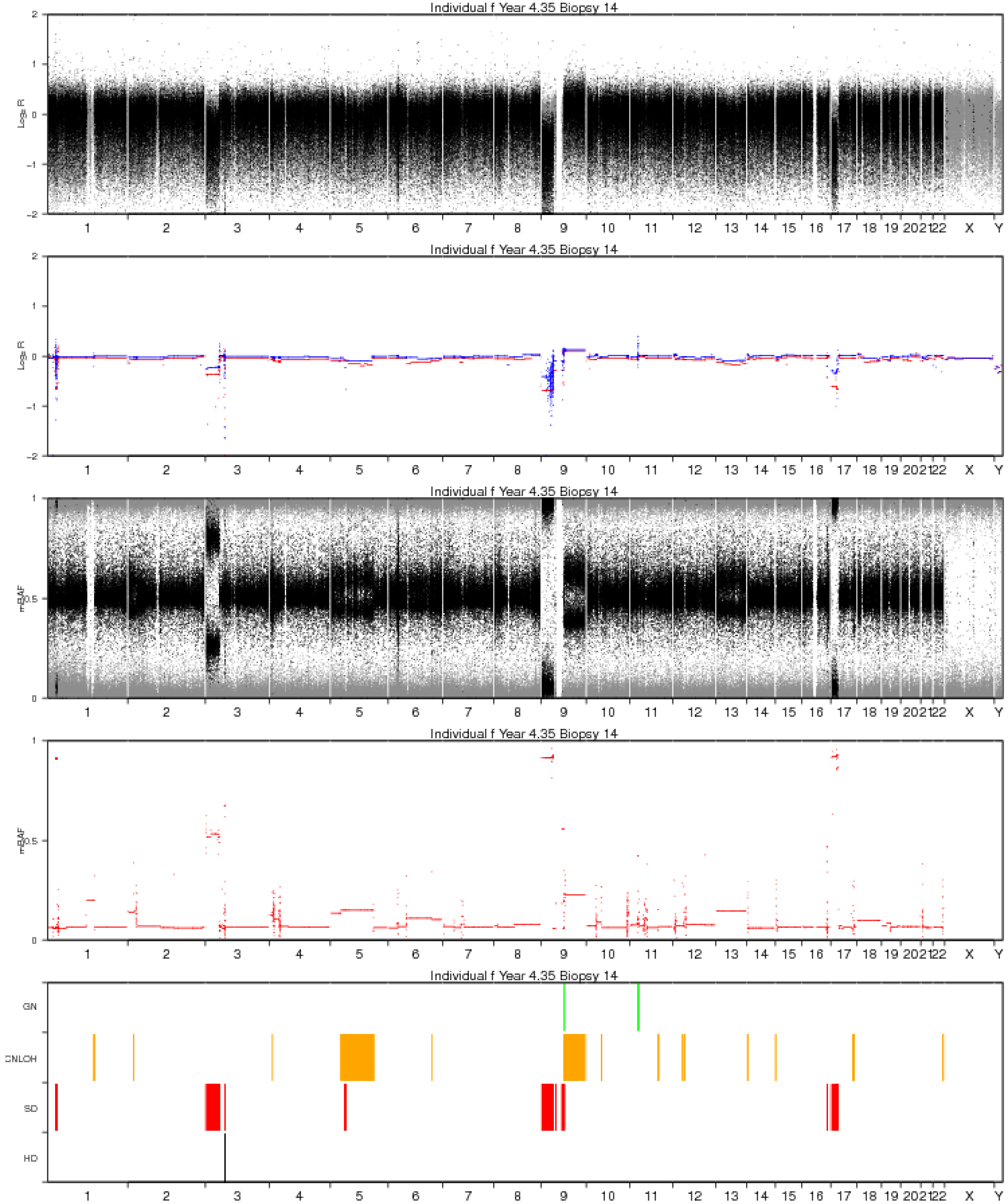
Individual f Year 4.35 Biopsy 14
Log R
mBAF
GN
CNLOH
SD
HD
1 2 3 4 5 6 7 8 9 10 11 12 13 14 15 16 17 18 19 20 21 22 X Y

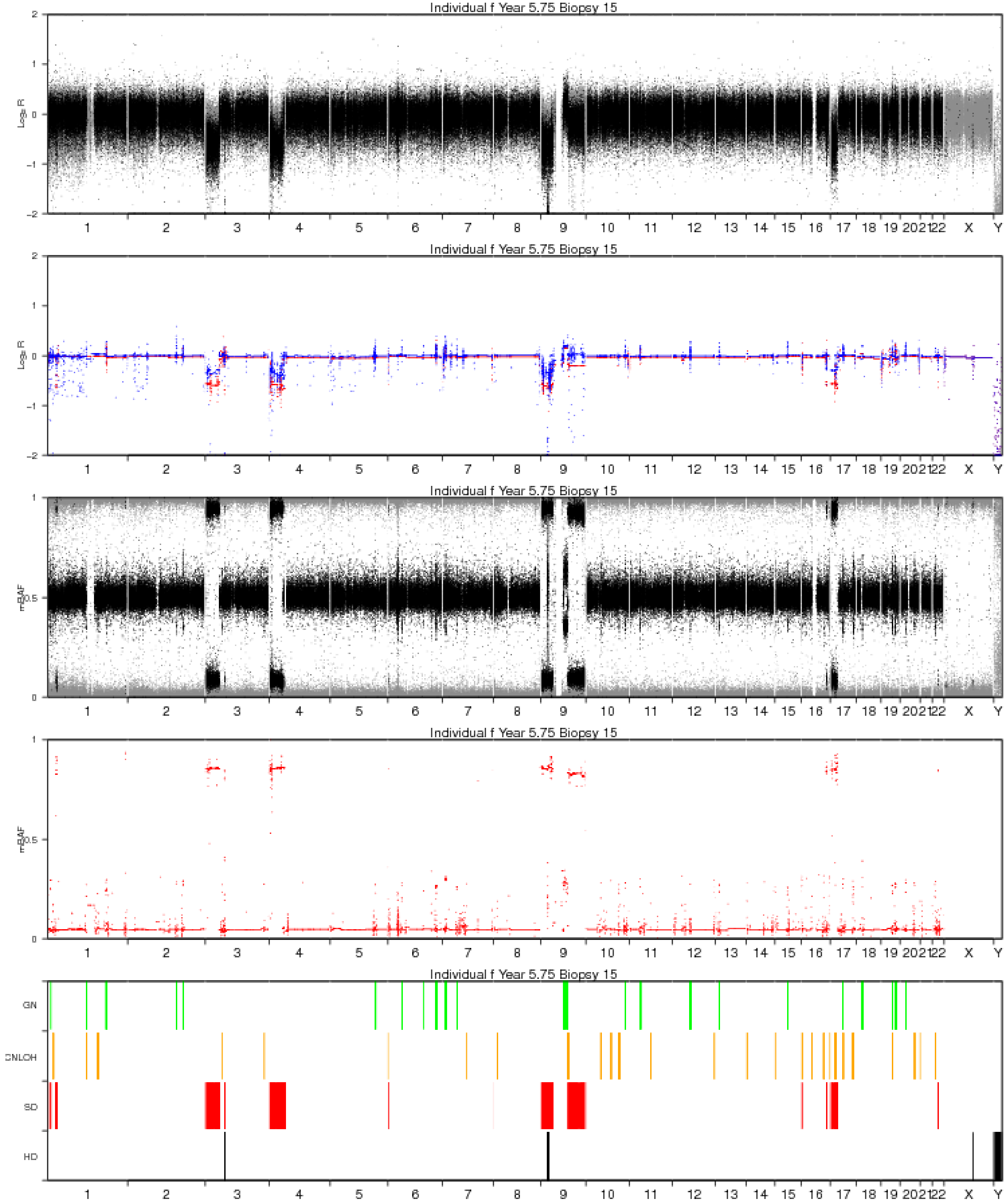

Individual f Year 5.75 Biopsy 15
Log₂ R
mBAF
GN
CNLOH
SD
HD
1 2 3 4 5 6 7 8 9 10 11 12 13 14 15 16 17 18 19 20 21 22 X Y

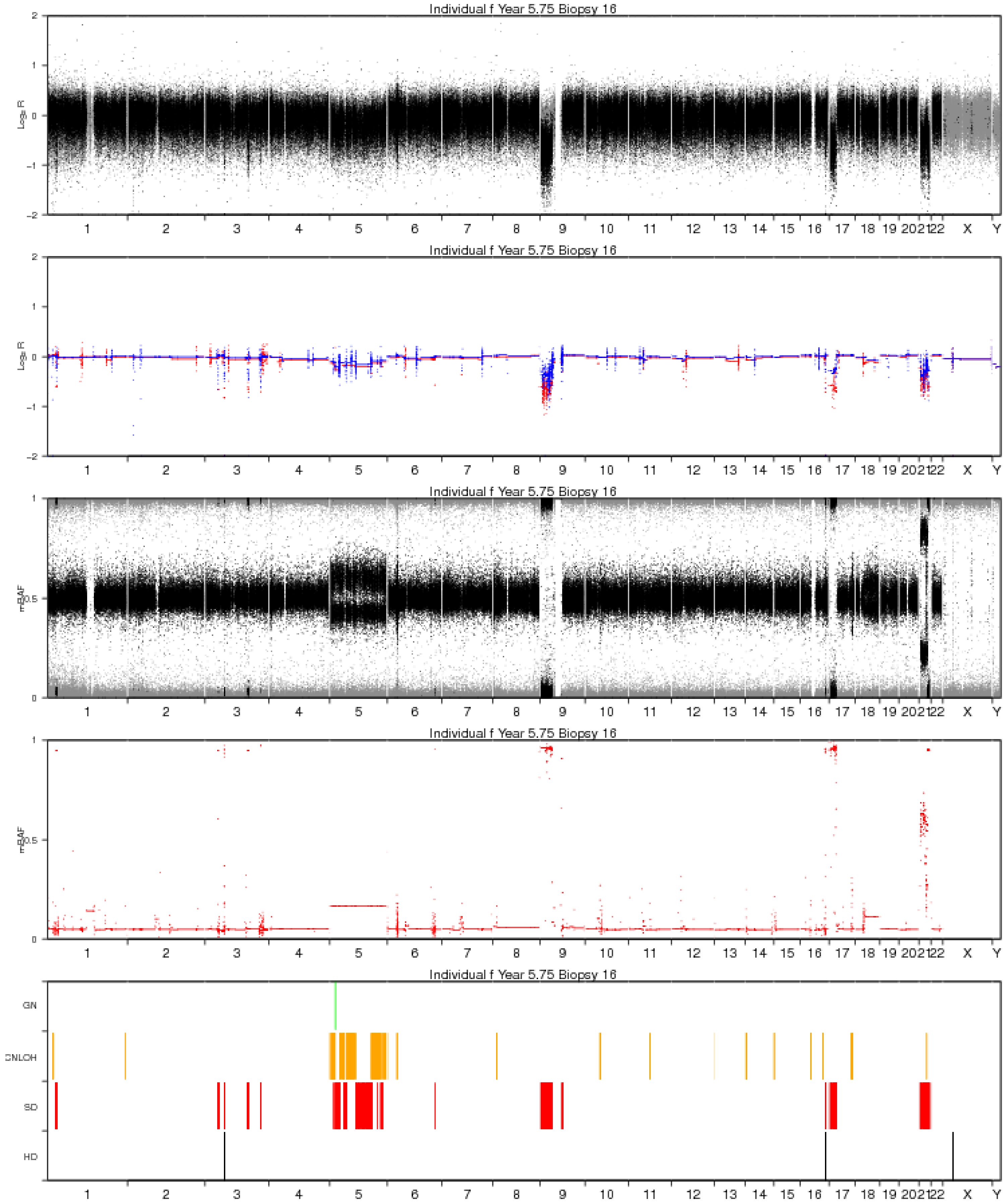
Individual f Year 5.75 Biopsy 16
Log R
2
1
0
−1
−2
1 2 3 4 5 6 7 8 9 10 11 12 13 14 15 16 17 18 19 2021 22 X Y
Individual f Year 5.75 Biopsy 16
Log R
2
1
0
−1
−2
1 2 3 4 5 6 7 8 9 10 11 12 13 14 15 16 17 18 19 2021 22 X Y
Individual f Year 5.75 Biopsy 16
mBAF
1
0.5
0
1 2 3 4 5 6 7 8 9 10 11 12 13 14 15 16 17 18 19 2021 22 X Y
Individual f Year 5.75 Biopsy 16
mBAF
1
0.5
0
1 2 3 4 5 6 7 8 9 10 11 12 13 14 15 16 17 18 19 2021 22 X Y
Individual f Year 5.75 Biopsy 16
GN
CNLOH
SD
HD
1 2 3 4 5 6 7 8 9 10 11 12 13 14 15 16 17 18 19 2021 22 X Y

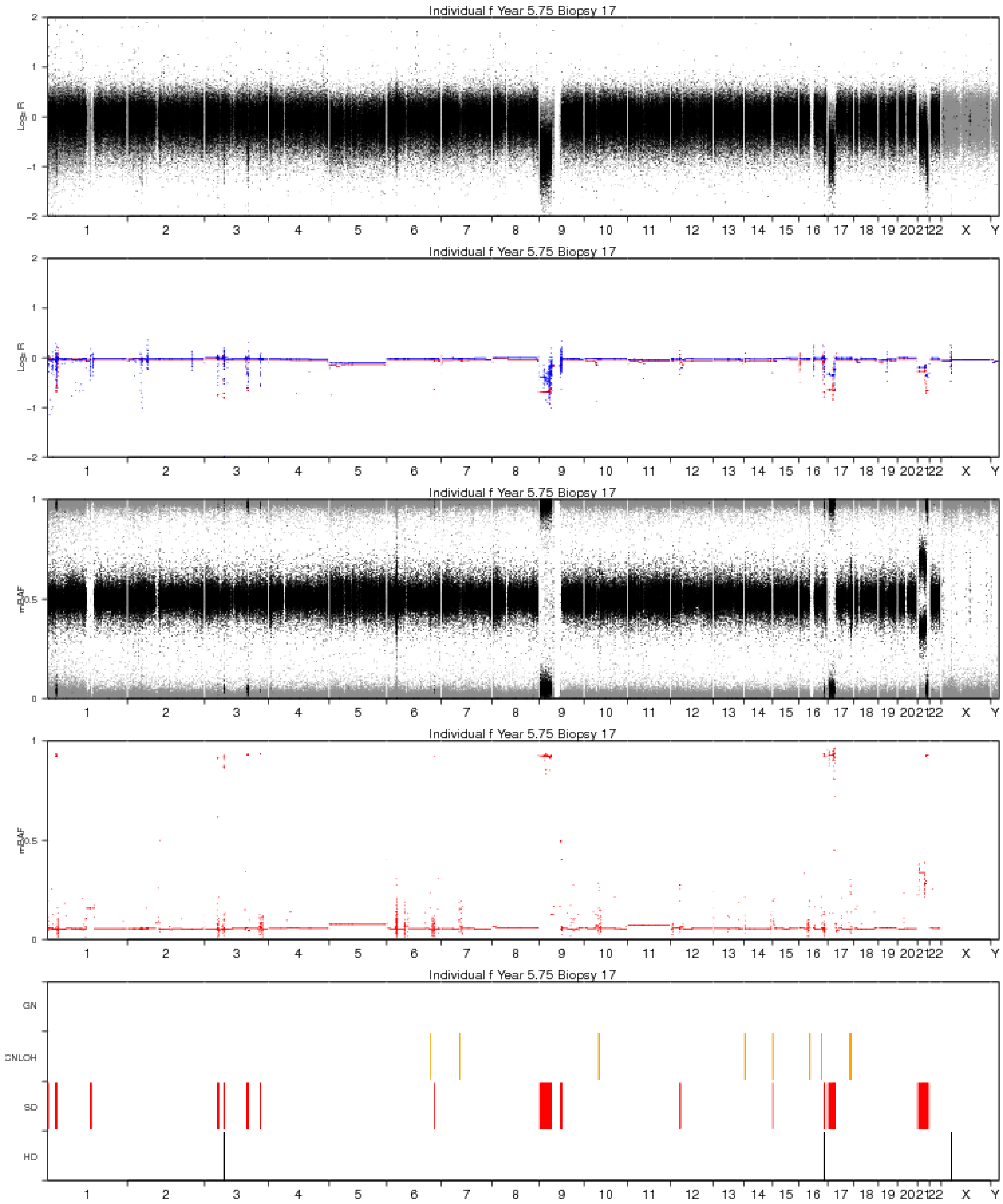
Individual f Year 5.75 Biopsy 17
Log R
mBAF
GN
CNLOH
SD
HD
1 2 3 4 5 6 7 8 9 10 11 12 13 14 15 16 17 18 19 20 21 22 X Y

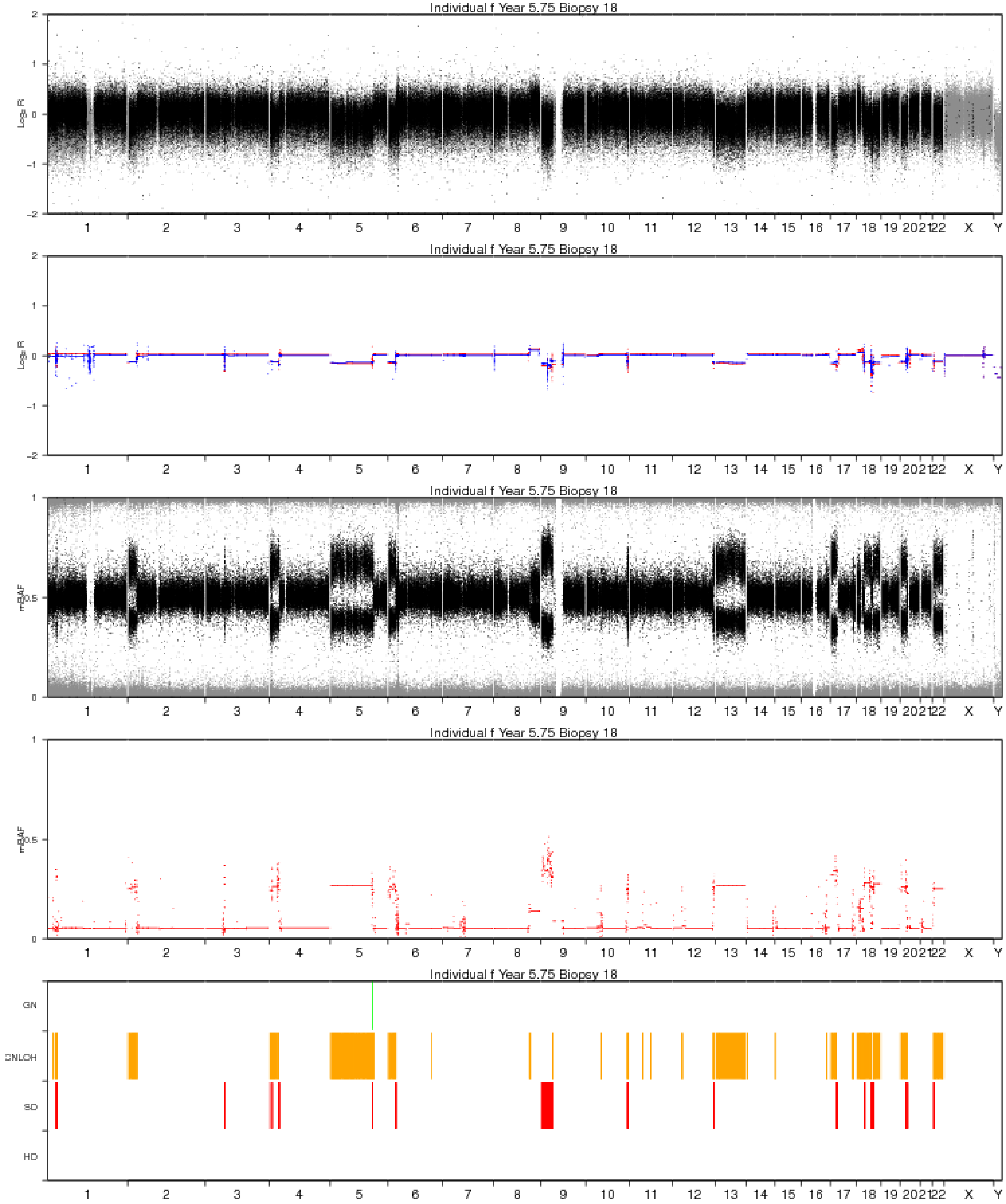
Individual f Year 5.75 Biopsy 18
Log R
mBAF
GN
CNLOH
SD
HD
1 2 3 4 5 6 7 8 9 10 11 12 13 14 15 16 17 18 19 20 21 22 X Y

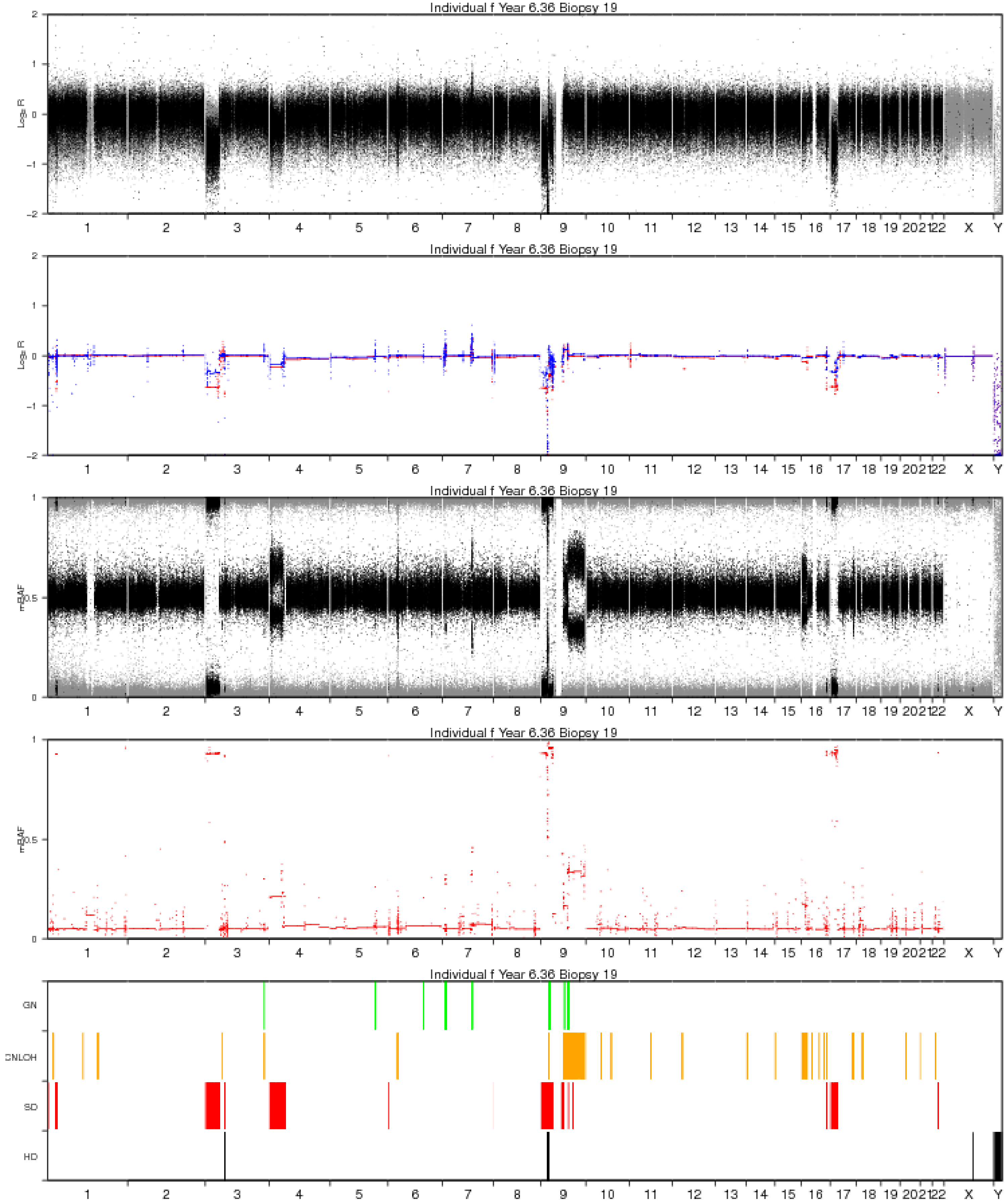
Individual f Year 6.36 Biopsy 19
Log R
mBAF
GN
CNLOH
SD
HD
1
2
3
4
5
6
7
8
9
10
11
12
13
14
15
16
17
18
19
20
21
22
X
Y

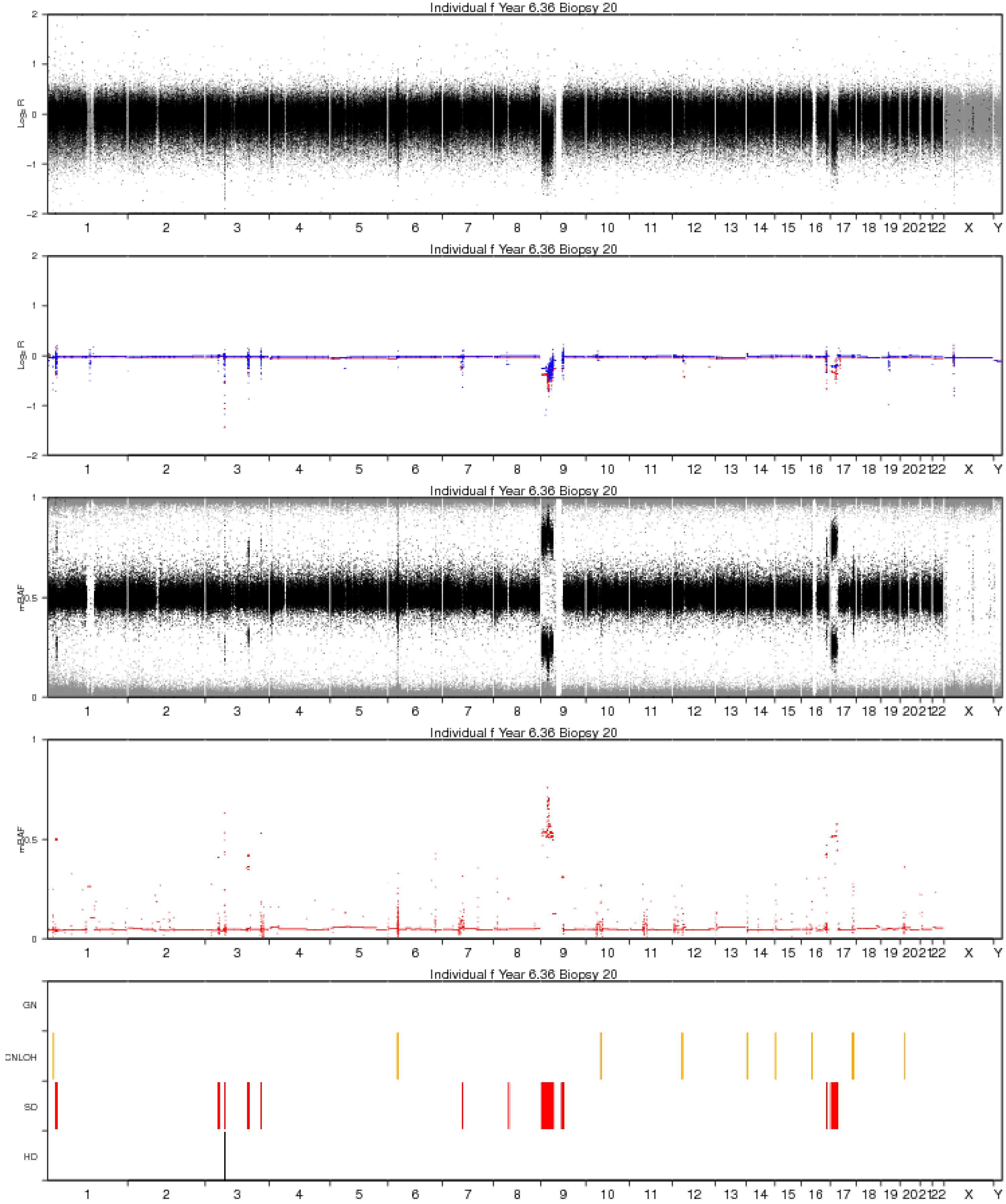
Individual f Year 6.36 Biopsy 20
Log2 R
2
1
0
−1
−2
1 2 3 4 5 6 7 8 9 10 11 12 13 14 15 16 17 18 19 20 21 22 X Y
Individual f Year 6.36 Biopsy 20
Log2 R
Individual f Year 6.36 Biopsy 20
mBAF
1
0.5
0
Individual f Year 6.36 Biopsy 20
mBAF
Individual f Year 6.36 Biopsy 20
GN
CNLOH
SD
HD

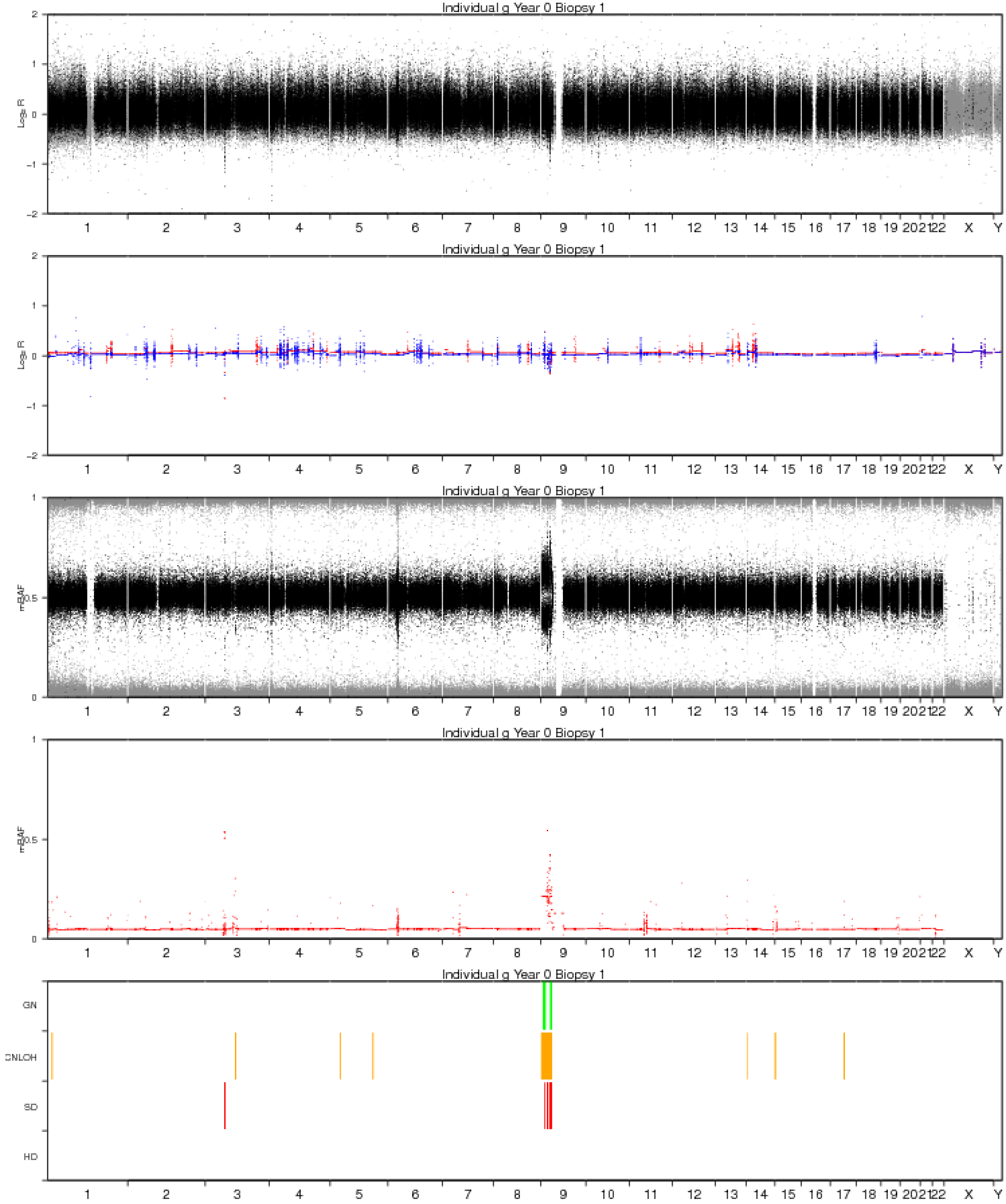

Individual g Year 0 Biopsy 1
Log R
mBAF
GN
CNLOH
SD
HD
1
2
3
4
5
6
7
8
9
10
11
12
13
14
15
16
17
18
19
20
21
22
X
Y

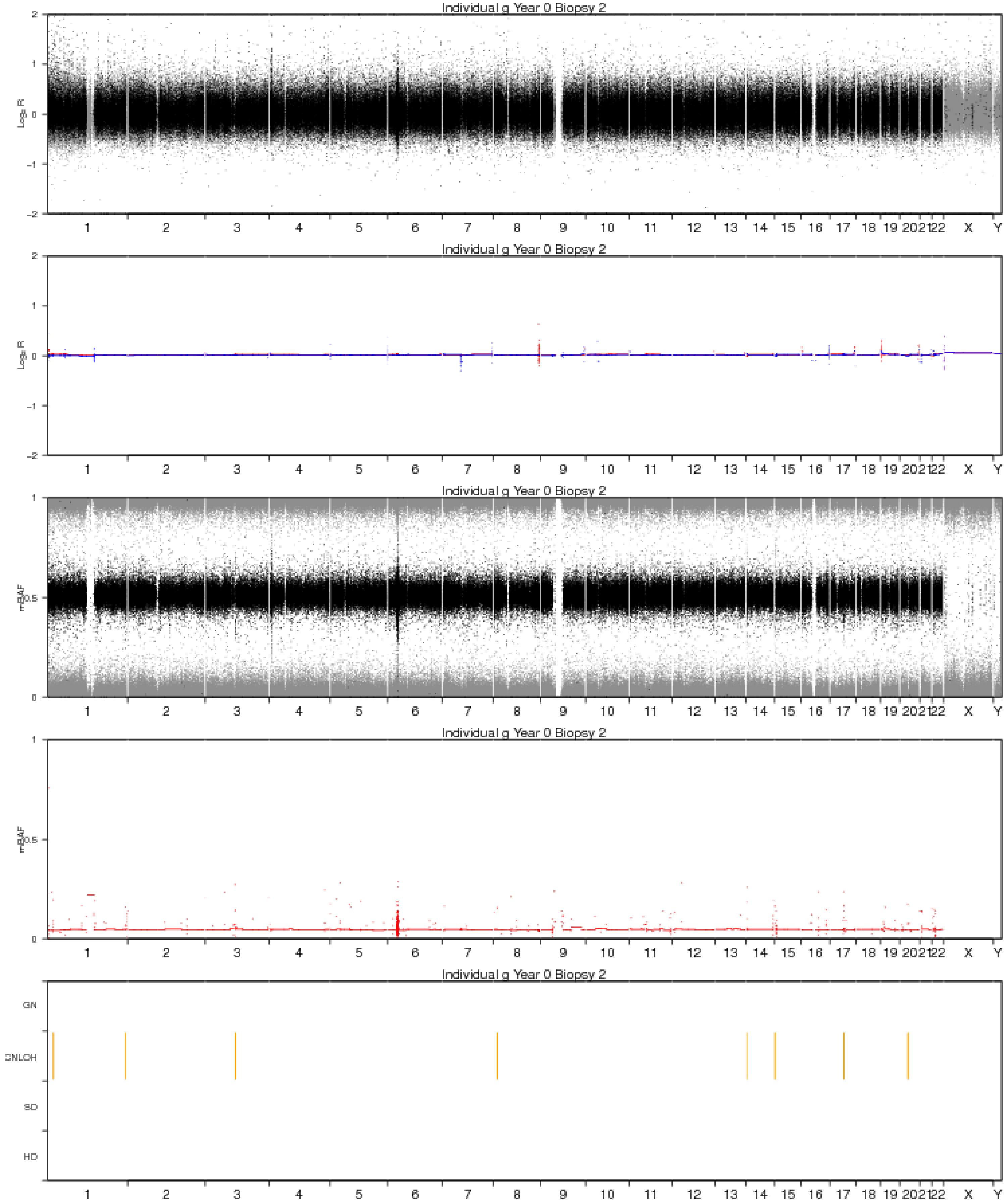
Individual g Year 0 Biopsy 2
Log R
2
1
0
−1
−2
1
2
3
4
5
6
7
8
9
10
11
12
13
14
15
16
17
18
19
20
21
22
X
Y
Individual g Year 0 Biopsy 2
Log R
Individual g Year 0 Biopsy 2
mBAF
1
0.5
0
Individual g Year 0 Biopsy 2
mBAF
Individual g Year 0 Biopsy 2
GN
CNLOH
SD
HD

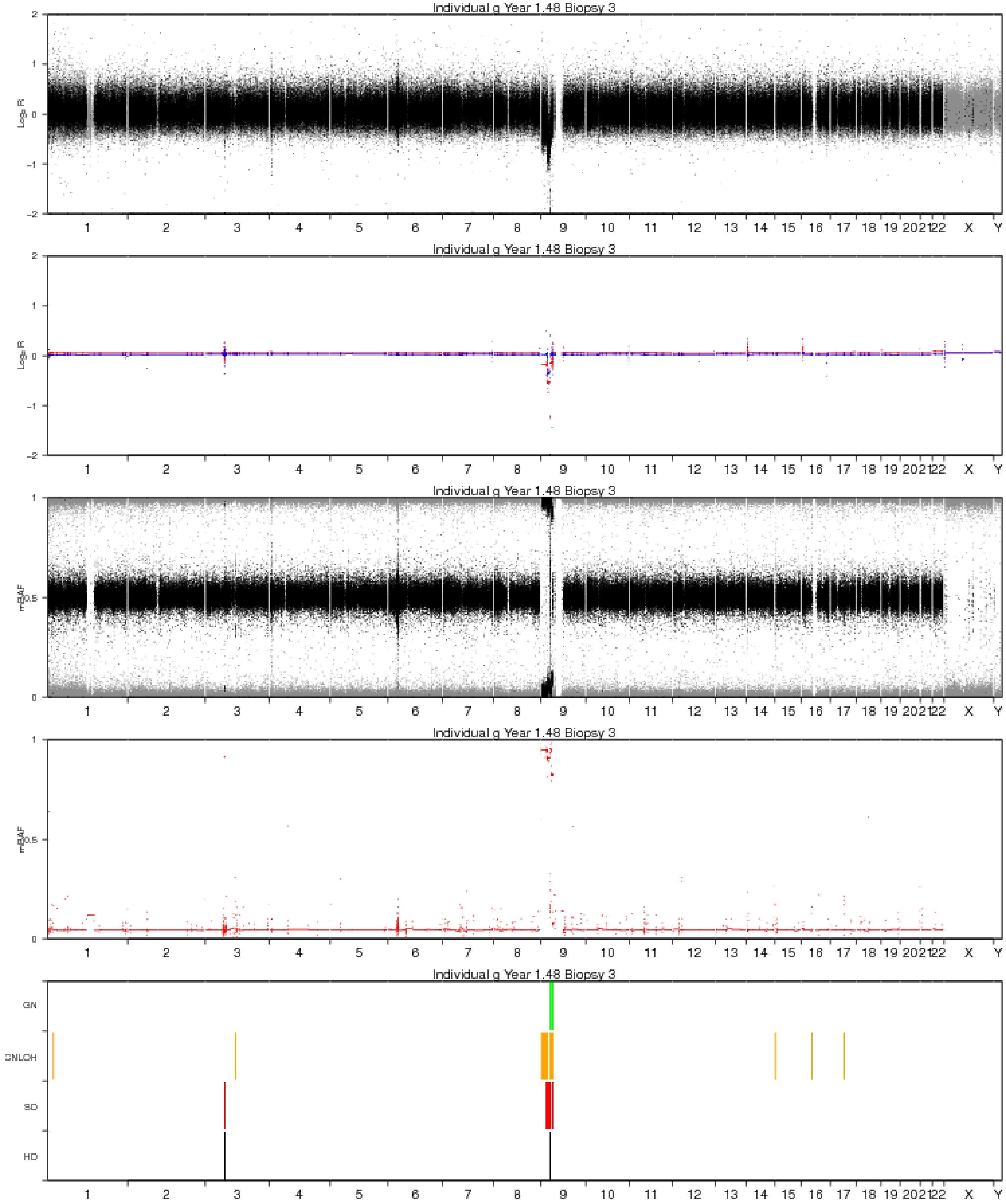
Individual g Year 1.48 Biopsy 3
Log R
2
1
0
−1
−2
1 2 3 4 5 6 7 8 9 10 11 12 13 14 15 16 17 18 19 20 21 22 X Y
Individual g Year 1.48 Biopsy 3
Log R
2
1
0
−1
−2
1 2 3 4 5 6 7 8 9 10 11 12 13 14 15 16 17 18 19 20 21 22 X Y
Individual g Year 1.48 Biopsy 3
mBAF
1
0.5
0
1 2 3 4 5 6 7 8 9 10 11 12 13 14 15 16 17 18 19 20 21 22 X Y
Individual g Year 1.48 Biopsy 3
mBAF
1
0.5
0
1 2 3 4 5 6 7 8 9 10 11 12 13 14 15 16 17 18 19 20 21 22 X Y
Individual g Year 1.48 Biopsy 3
GN
CNLOH
SD
HD
1 2 3 4 5 6 7 8 9 10 11 12 13 14 15 16 17 18 19 20 21 22 X Y

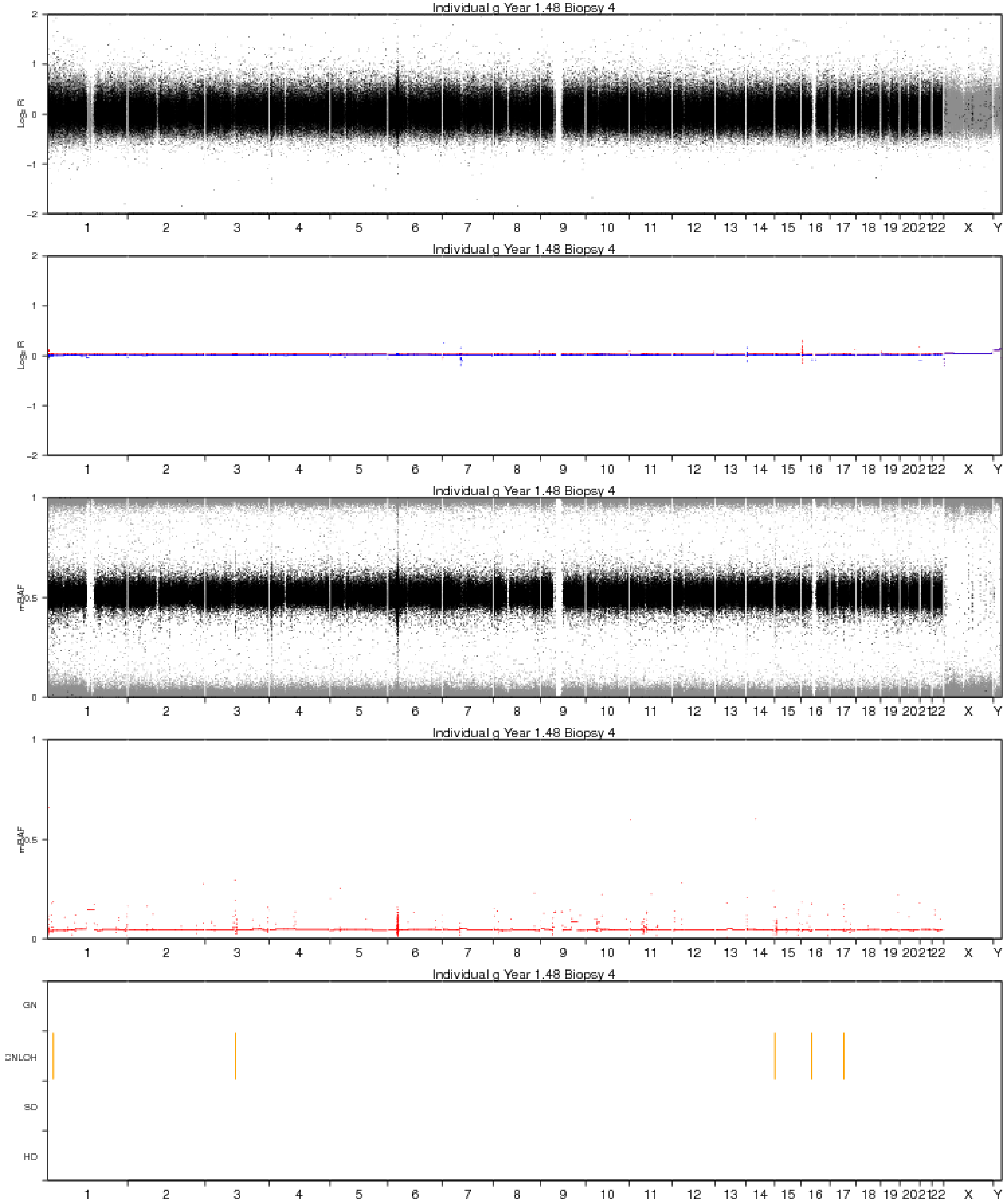
Individual g Year 1.48 Biopsy 4
Log R
1 2 3 4 5 6 7 8 9 10 11 12 13 14 15 16 17 18 19 20 21 22 X Y
Individual g Year 1.48 Biopsy 4
Log R
Individual g Year 1.48 Biopsy 4
mBAF
Individual g Year 1.48 Biopsy 4
mBAF
Individual g Year 1.48 Biopsy 4
GN
CNLOH
SD
HD

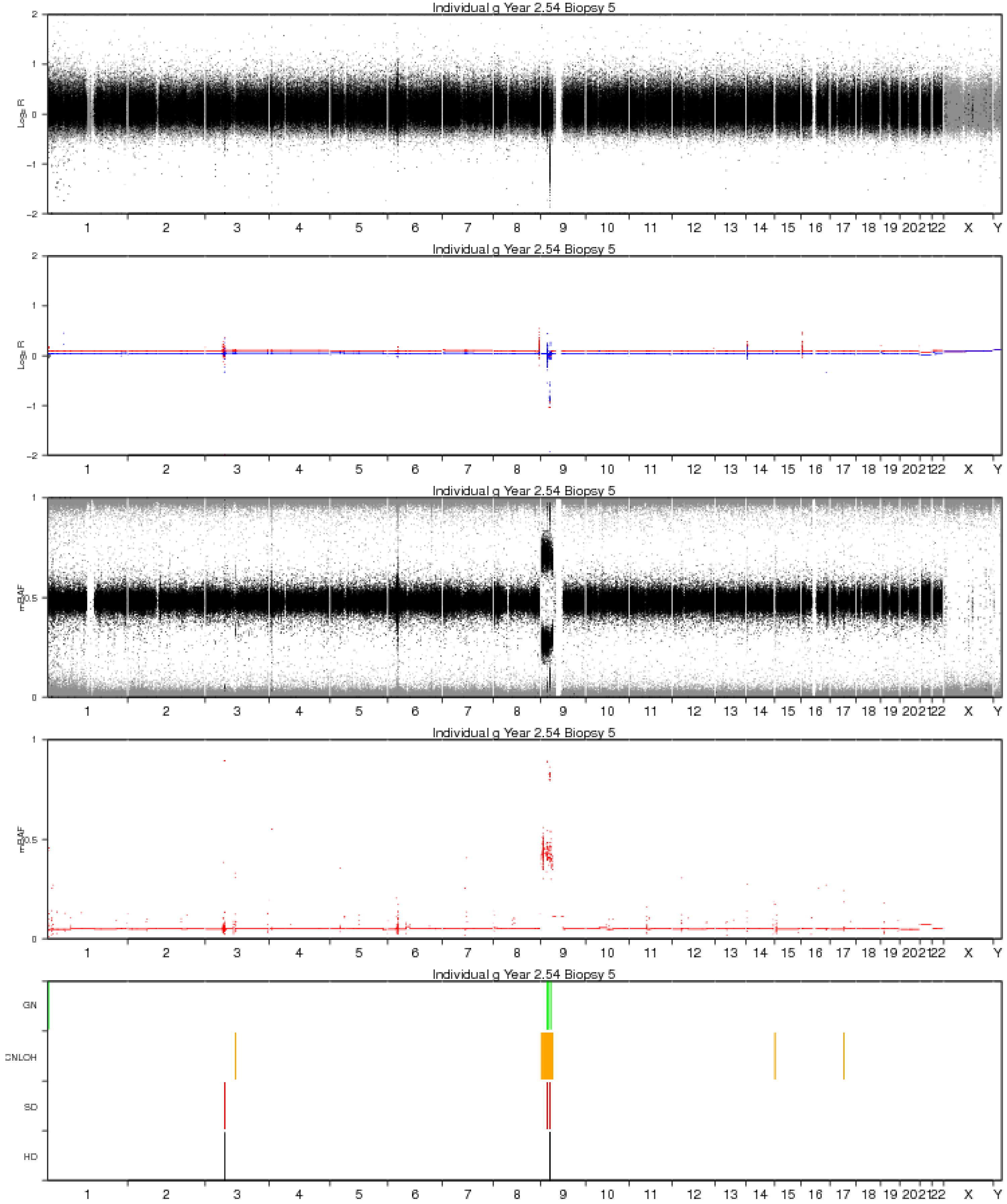

Individual g Year 2.54 Biopsy 5
Log R
2
1
0
−1
−2
1 2 3 4 5 6 7 8 9 10 11 12 13 14 15 16 17 18 19 20 21 22 X Y
Individual g Year 2.54 Biopsy 5
Log R
2
1
0
−1
−2
1 2 3 4 5 6 7 8 9 10 11 12 13 14 15 16 17 18 19 20 21 22 X Y
Individual g Year 2.54 Biopsy 5
mBAF
1
0.5
0
1 2 3 4 5 6 7 8 9 10 11 12 13 14 15 16 17 18 19 20 21 22 X Y
Individual g Year 2.54 Biopsy 5
mBAF
1
0.5
0
1 2 3 4 5 6 7 8 9 10 11 12 13 14 15 16 17 18 19 20 21 22 X Y
Individual g Year 2.54 Biopsy 5
GN
CNLOH
SD
HD
1 2 3 4 5 6 7 8 9 10 11 12 13 14 15 16 17 18 19 20 21 22 X Y

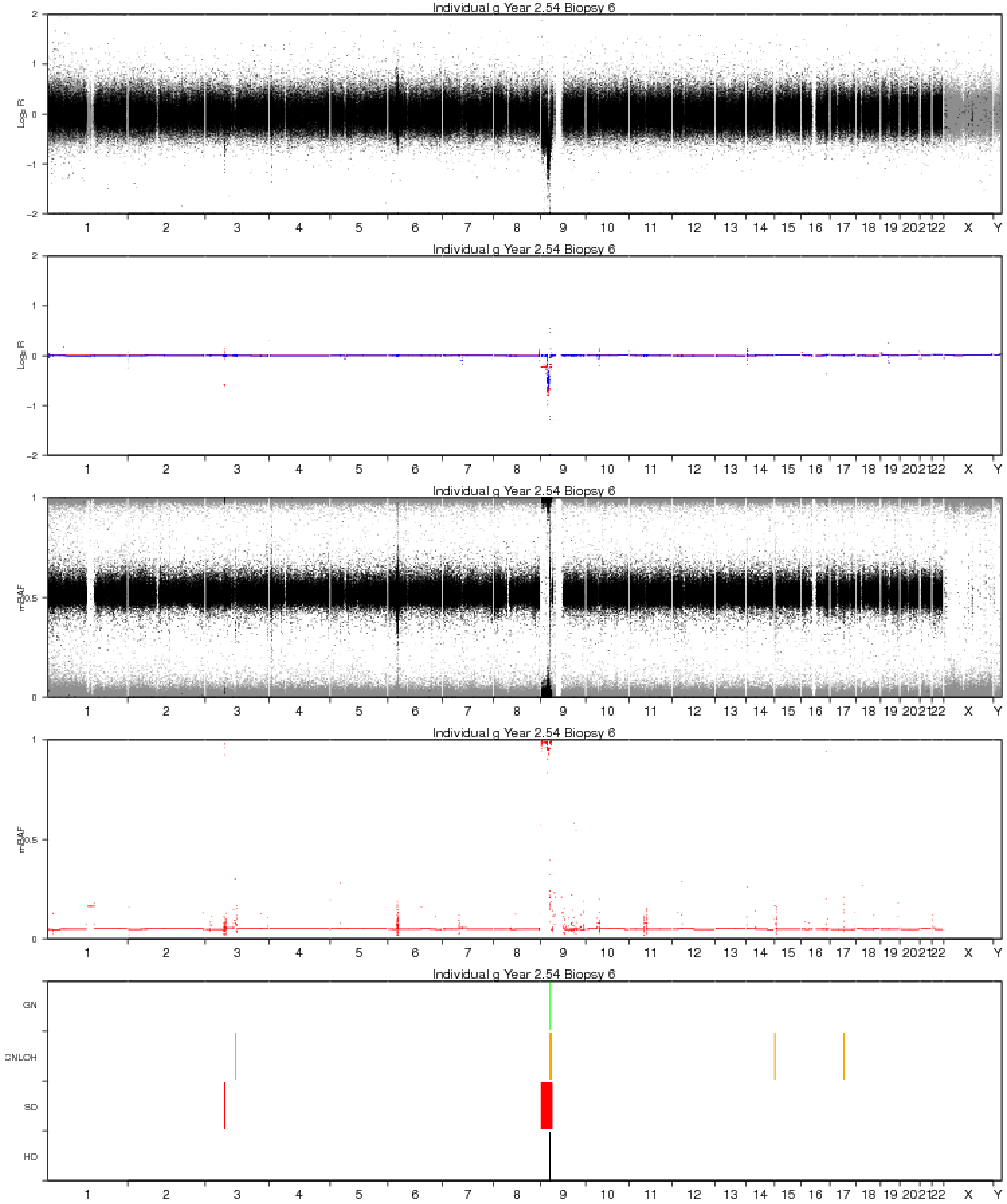
Individual g Year 2.54 Biopsy 6
Log R
mBAF
GN
CNLOH
SD
HD
1 2 3 4 5 6 7 8 9 10 11 12 13 14 15 16 17 18 19 20 21 22 X Y

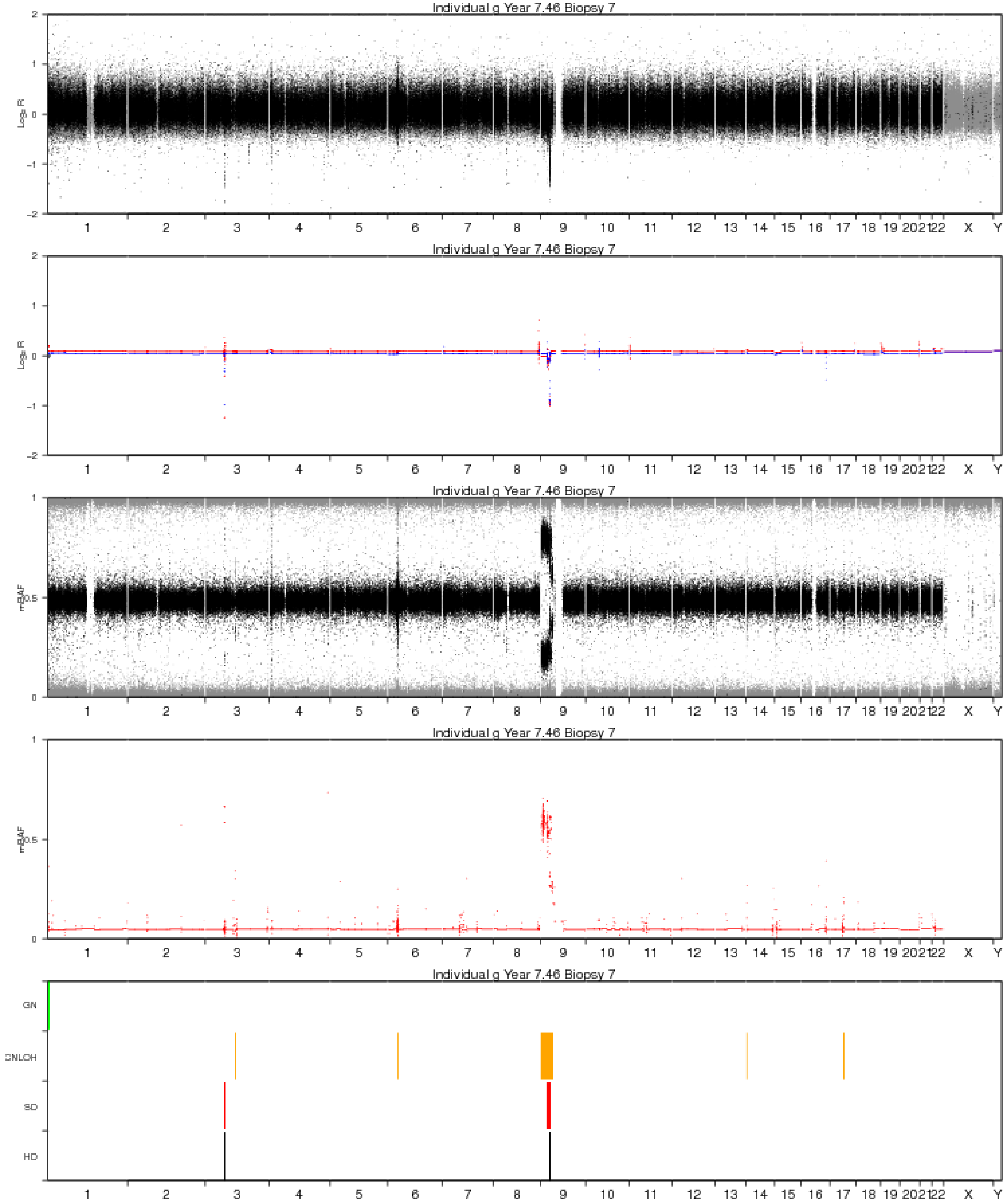
Individual g Year 7.46 Biopsy 7
Log R
mBAF
GN
CNLOH
SD
HD
1 2 3 4 5 6 7 8 9 10 11 12 13 14 15 16 17 18 19 20 21 22 X Y

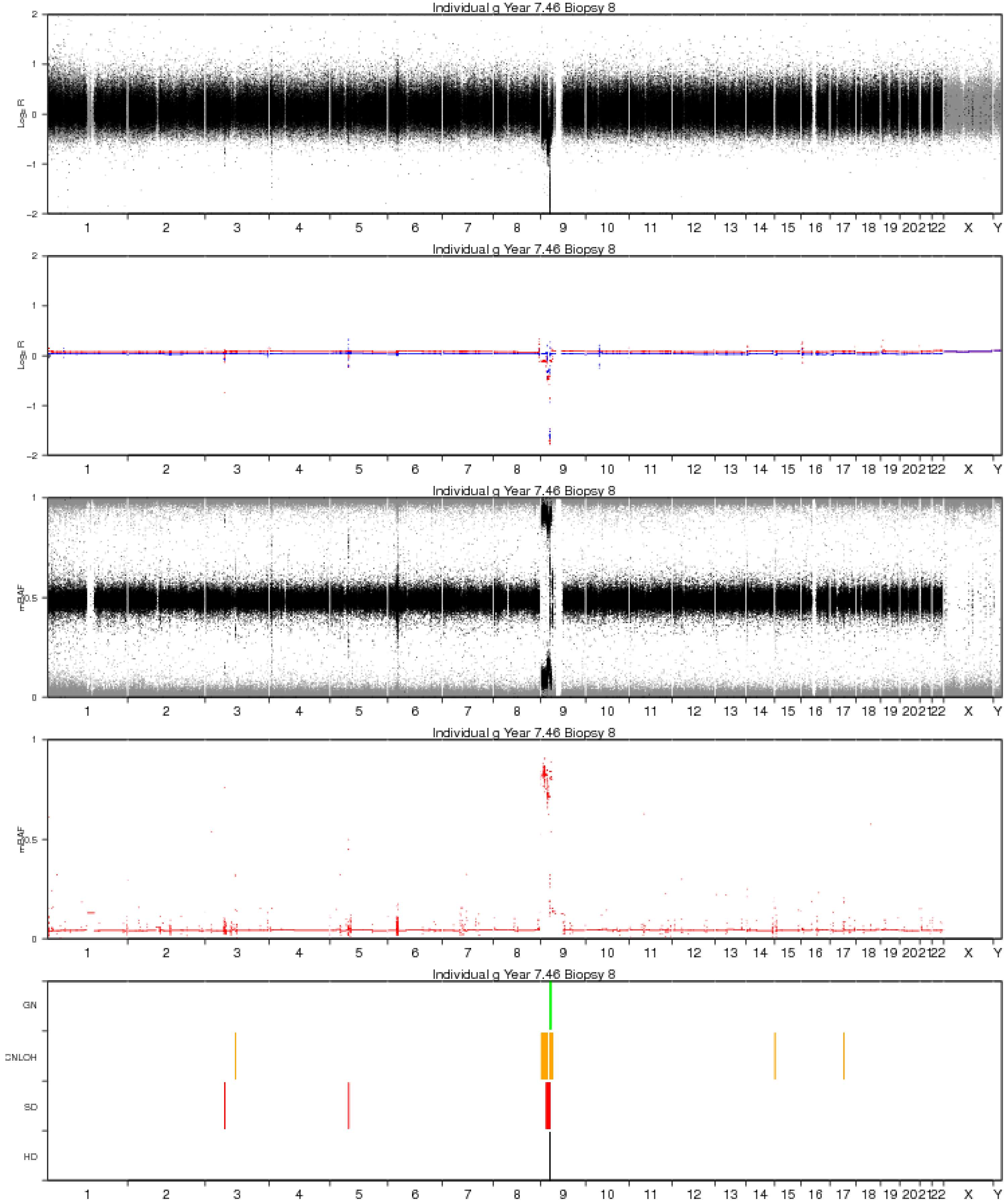

Individual g Year 7.46 Biopsy 8
Log R
mBAF
GN
CNLOH
SD
HD
1 2 3 4 5 6 7 8 9 10 11 12 13 14 15 16 17 18 19 20 21 22 X Y

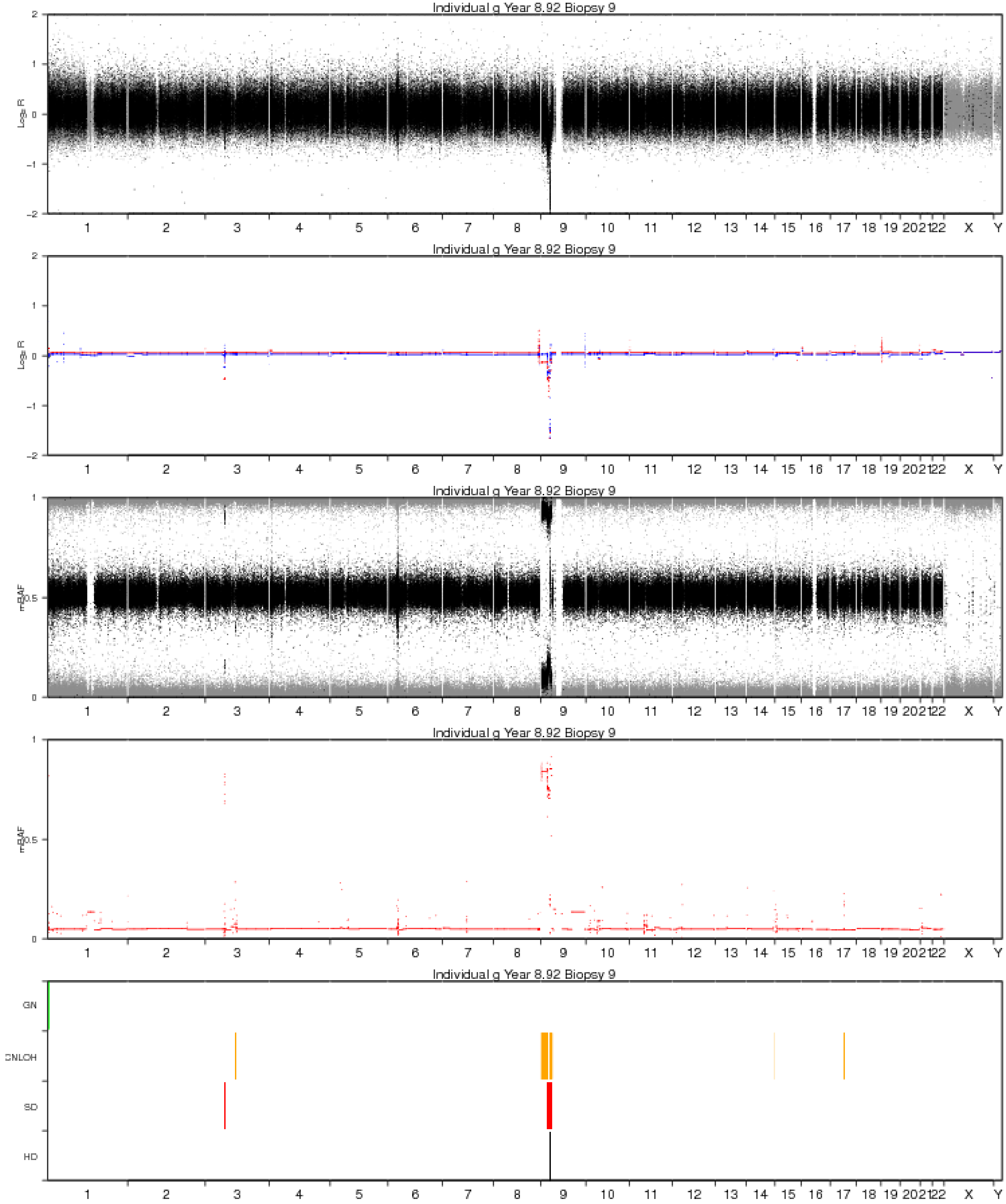

Individual g Year 8.92 Biopsy 9
Log R
mBAF
GN
CNLOH
SD
HD
1 2 3 4 5 6 7 8 9 10 11 12 13 14 15 16 17 18 19 20 21 22 X Y

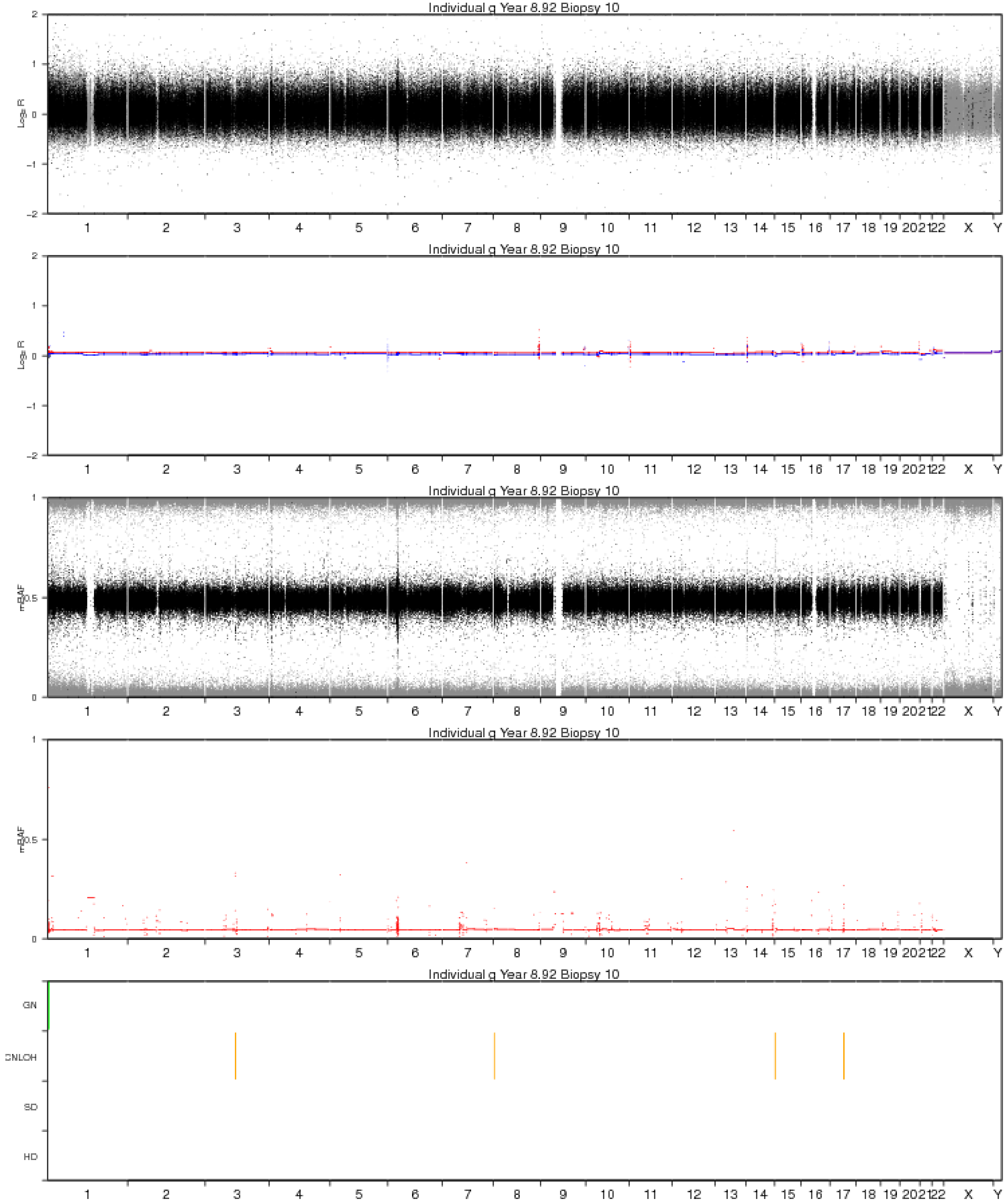
Individual g Year 8.92 Biopsy 10
Log2 R
2
1
0
−1
−2
1 2 3 4 5 6 7 8 9 10 11 12 13 14 15 16 17 18 19 20 21 22 X Y
Individual g Year 8.92 Biopsy 10
Log2 R
Individual g Year 8.92 Biopsy 10
mBAF
1
0.5
0
Individual g Year 8.92 Biopsy 10
mBAF
Individual g Year 8.92 Biopsy 10
GN
CNLOH
SD
HD

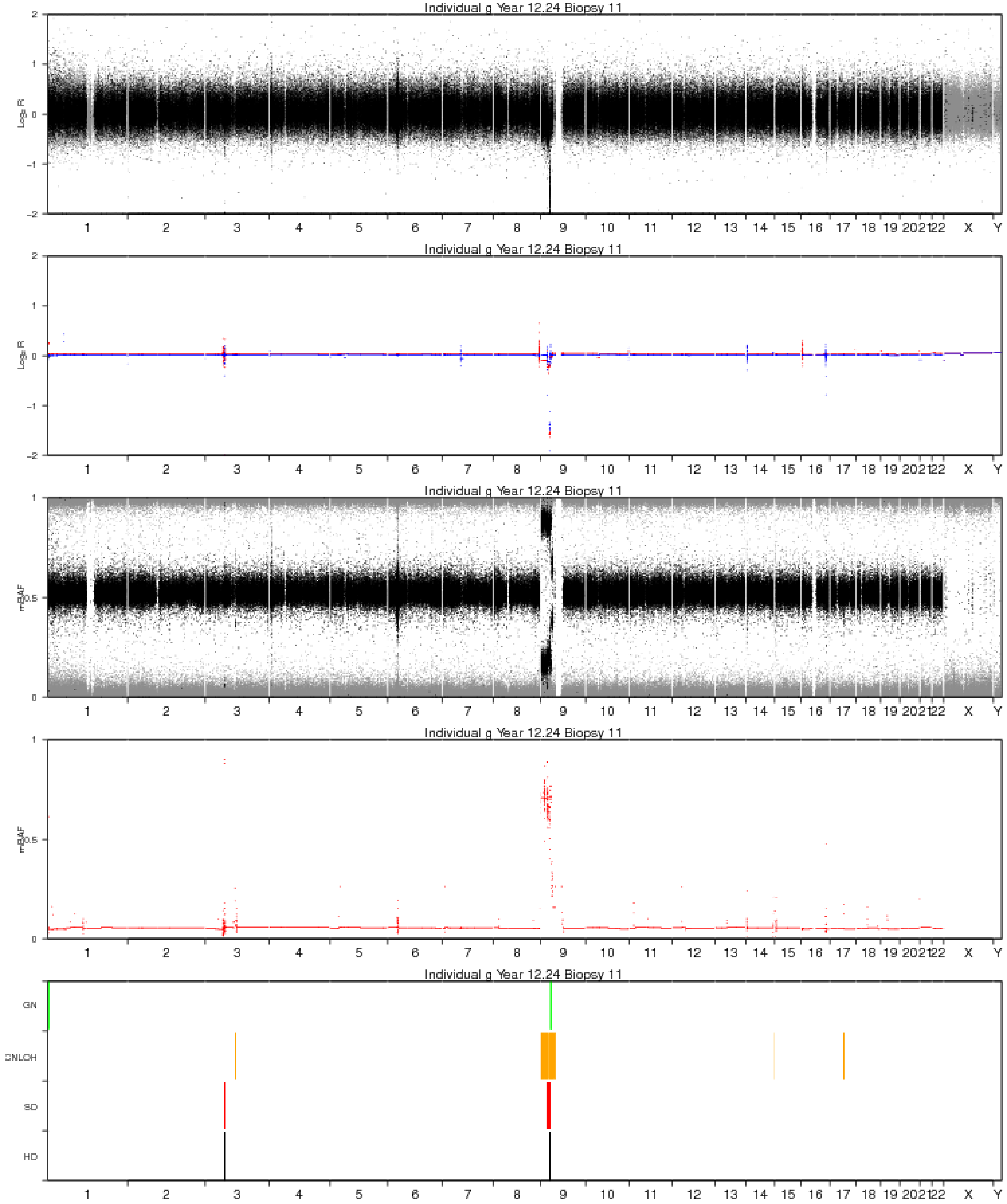

Individual g Year 12.24 Biopsy 11
Log₂ R
mBAF
GN
CNLOH
SD
HD
1 2 3 4 5 6 7 8 9 10 11 12 13 14 15 16 17 18 19 20 21 22 X Y

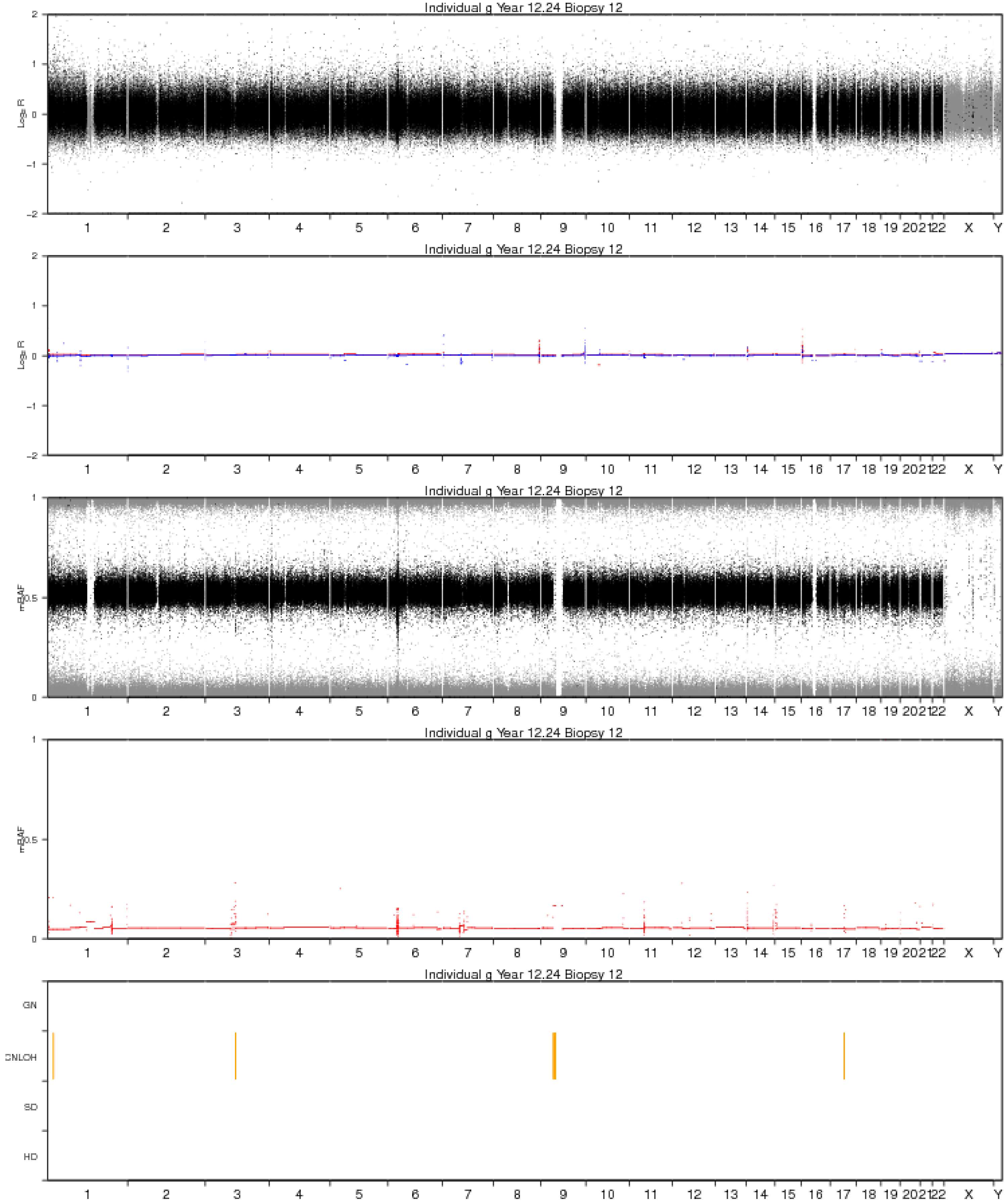
Individual g Year 12.24 Biopsy 12
Log R
mBAF
GN
CNLOH
SD
HD
1 2 3 4 5 6 7 8 9 10 11 12 13 14 15 16 17 18 19 20 21 22 X Y

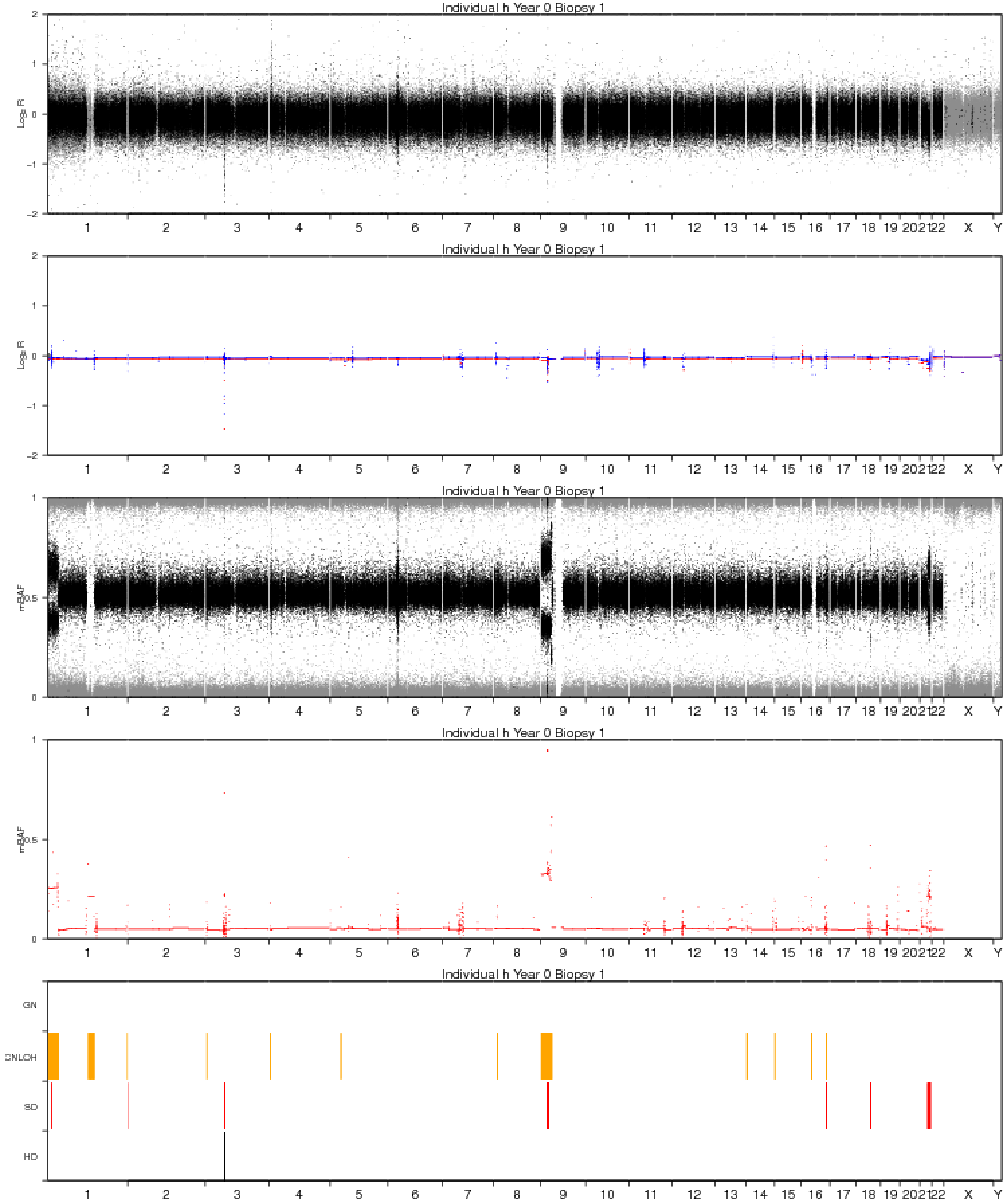

Individual h Year 0 Biopsy 1
Log R
2
1
0
−1
−2
1 2 3 4 5 6 7 8 9 10 11 12 13 14 15 16 17 18 19 2021 22 X Y
Individual h Year 0 Biopsy 1
Log R
2
1
0
−1
−2
1 2 3 4 5 6 7 8 9 10 11 12 13 14 15 16 17 18 19 2021 22 X Y
Individual h Year 0 Biopsy 1
mBAF
1
0.5
0
1 2 3 4 5 6 7 8 9 10 11 12 13 14 15 16 17 18 19 2021 22 X Y
Individual h Year 0 Biopsy 1
mBAF
1
0.5
0
1 2 3 4 5 6 7 8 9 10 11 12 13 14 15 16 17 18 19 2021 22 X Y
Individual h Year 0 Biopsy 1
GN
CNLOH
SD
HD
1 2 3 4 5 6 7 8 9 10 11 12 13 14 15 16 17 18 19 2021 22 X Y

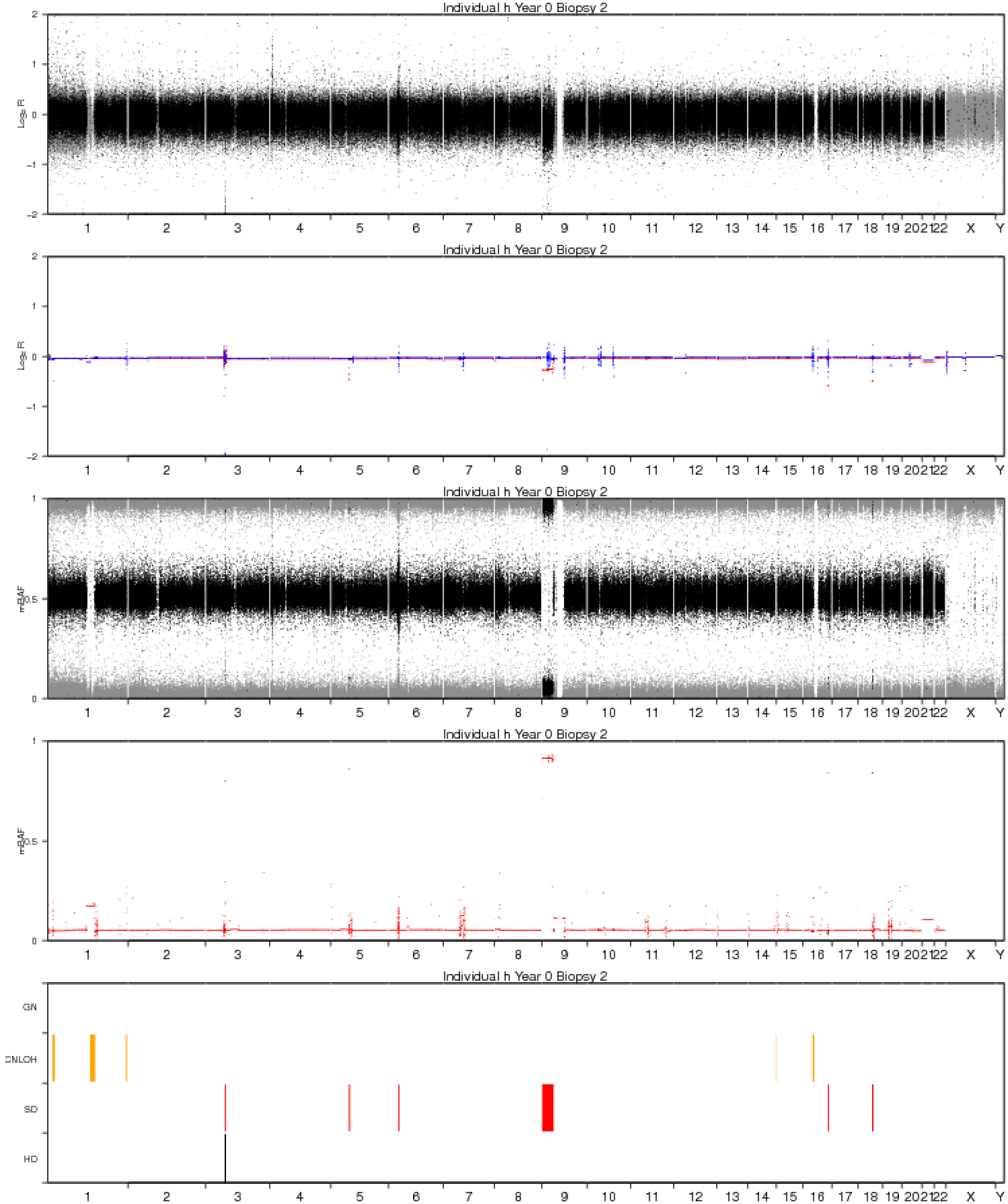
Individual h Year 0 Biopsy 2
Log2 R
mBAF
GN
CNLOH
SD
HD
1 2 3 4 5 6 7 8 9 10 11 12 13 14 15 16 17 18 19 20 21 22 X Y

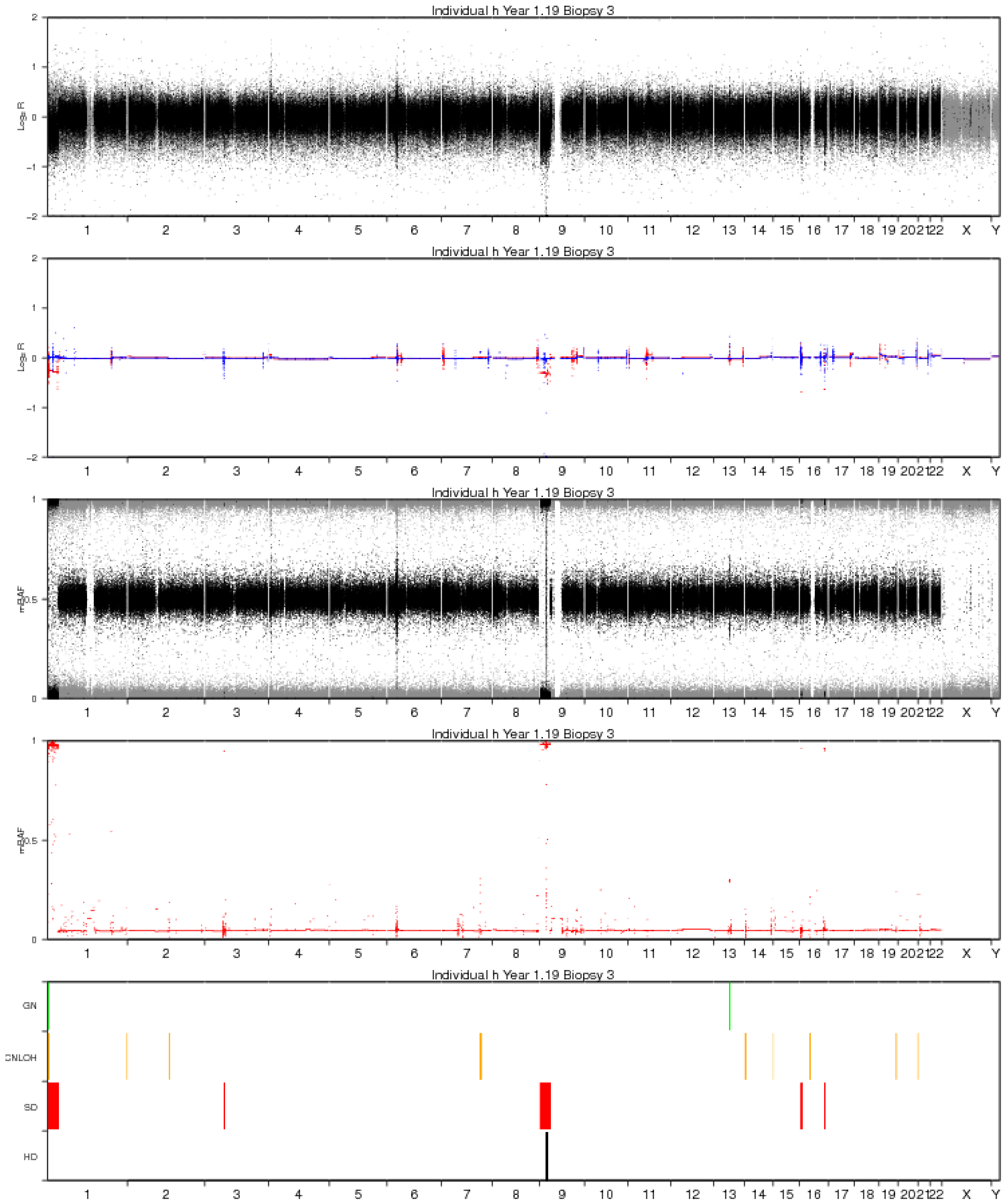

Individual h Year 1.19 Biopsy 3
Log R
2
1
0
−1
−2
1 2 3 4 5 6 7 8 9 10 11 12 13 14 15 16 17 18 19 20 21 22 X Y
Individual h Year 1.19 Biopsy 3
Log R
Individual h Year 1.19 Biopsy 3
mBAF
1
0.5
0
Individual h Year 1.19 Biopsy 3
mBAF
Individual h Year 1.19 Biopsy 3
GN
CNLOH
SD
HD

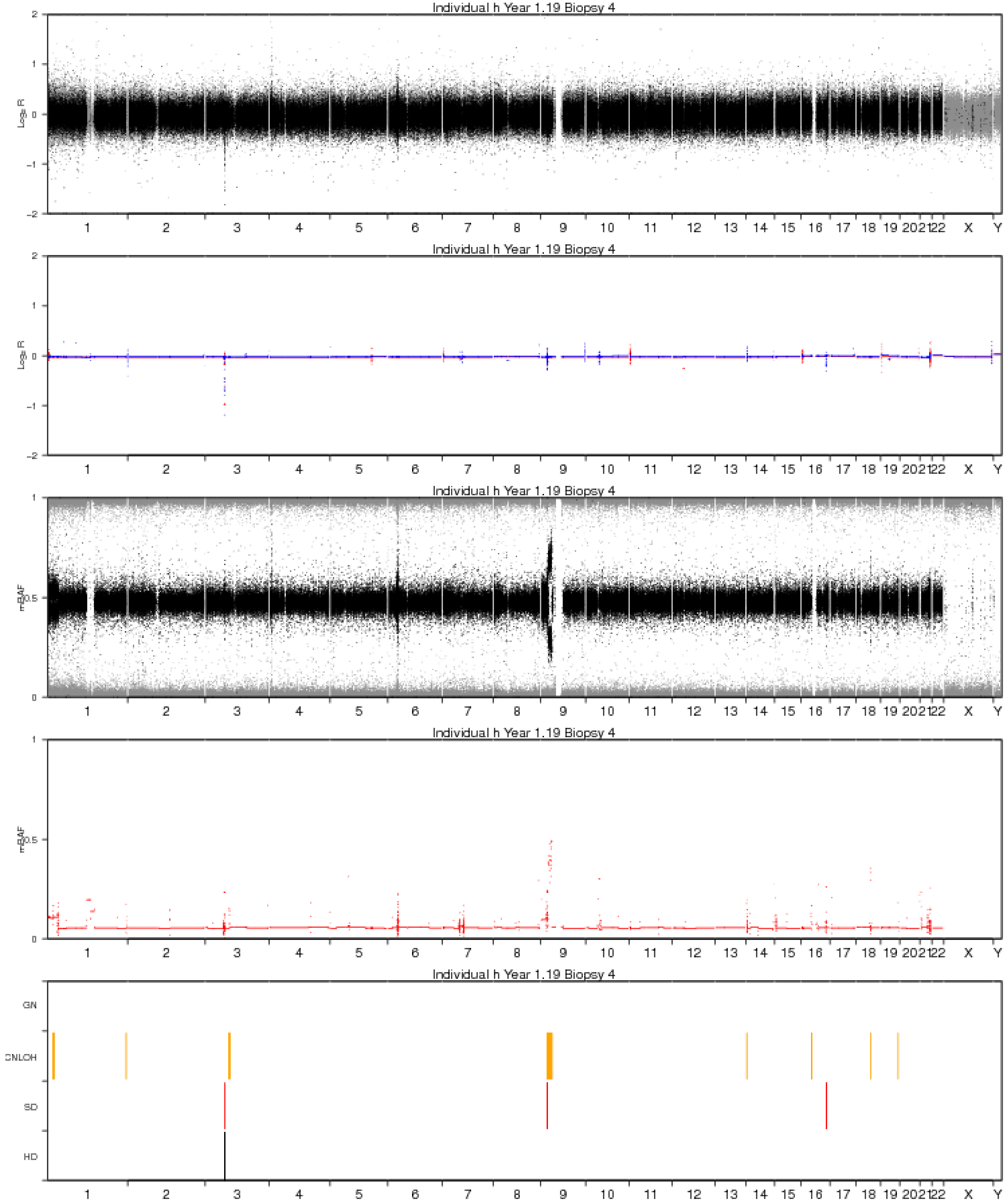

Individual h Year 1.19 Biopsy 4
Log R
mBAF
GN
CNLOH
SD
HD
1 2 3 4 5 6 7 8 9 10 11 12 13 14 15 16 17 18 19 20 21 22 X Y

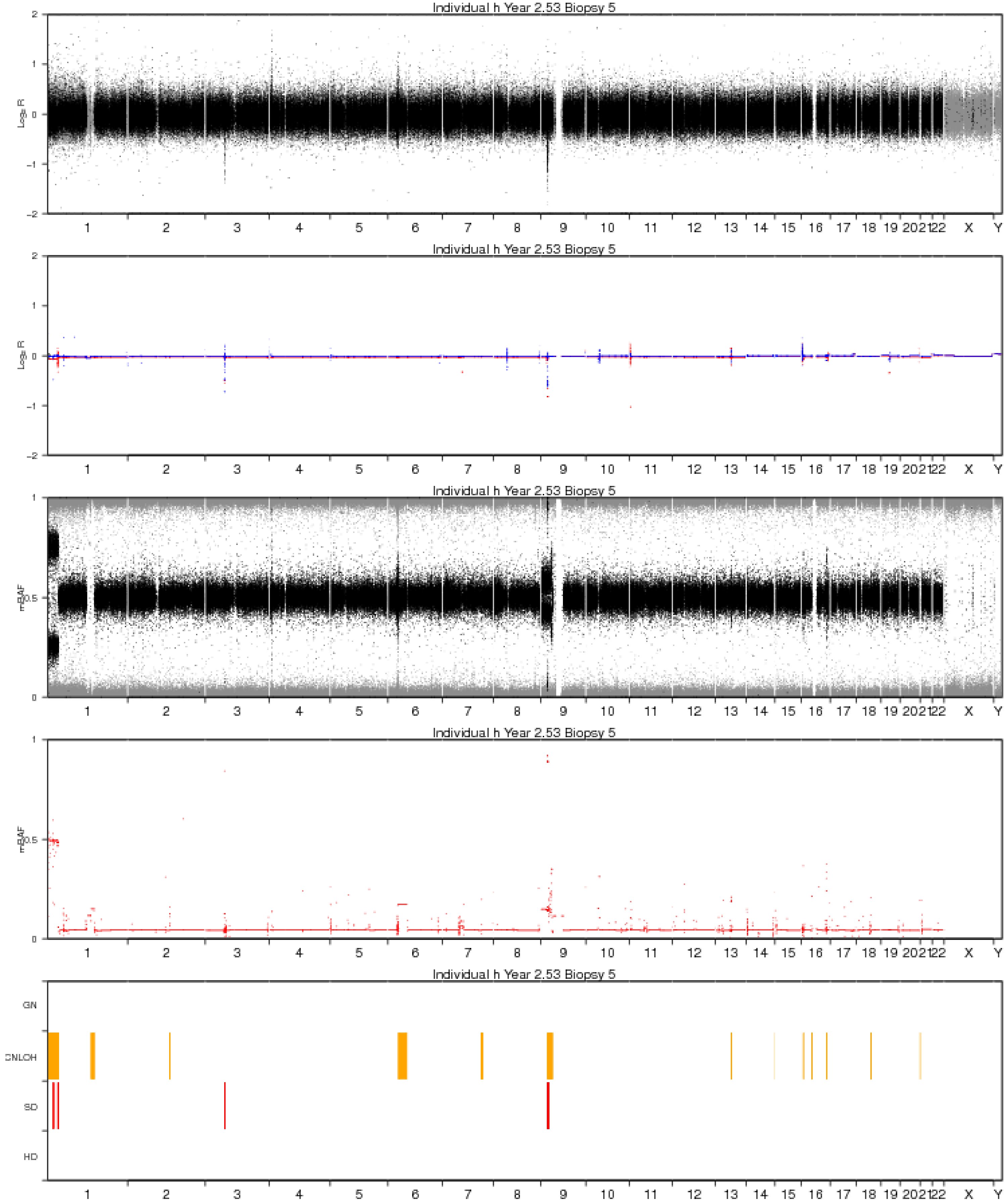
Individual h Year 2.53 Biopsy 5
Log R
mBAF
GN
CNLOH
SD
HD
1 2 3 4 5 6 7 8 9 10 11 12 13 14 15 16 17 18 19 20 21 22 X Y

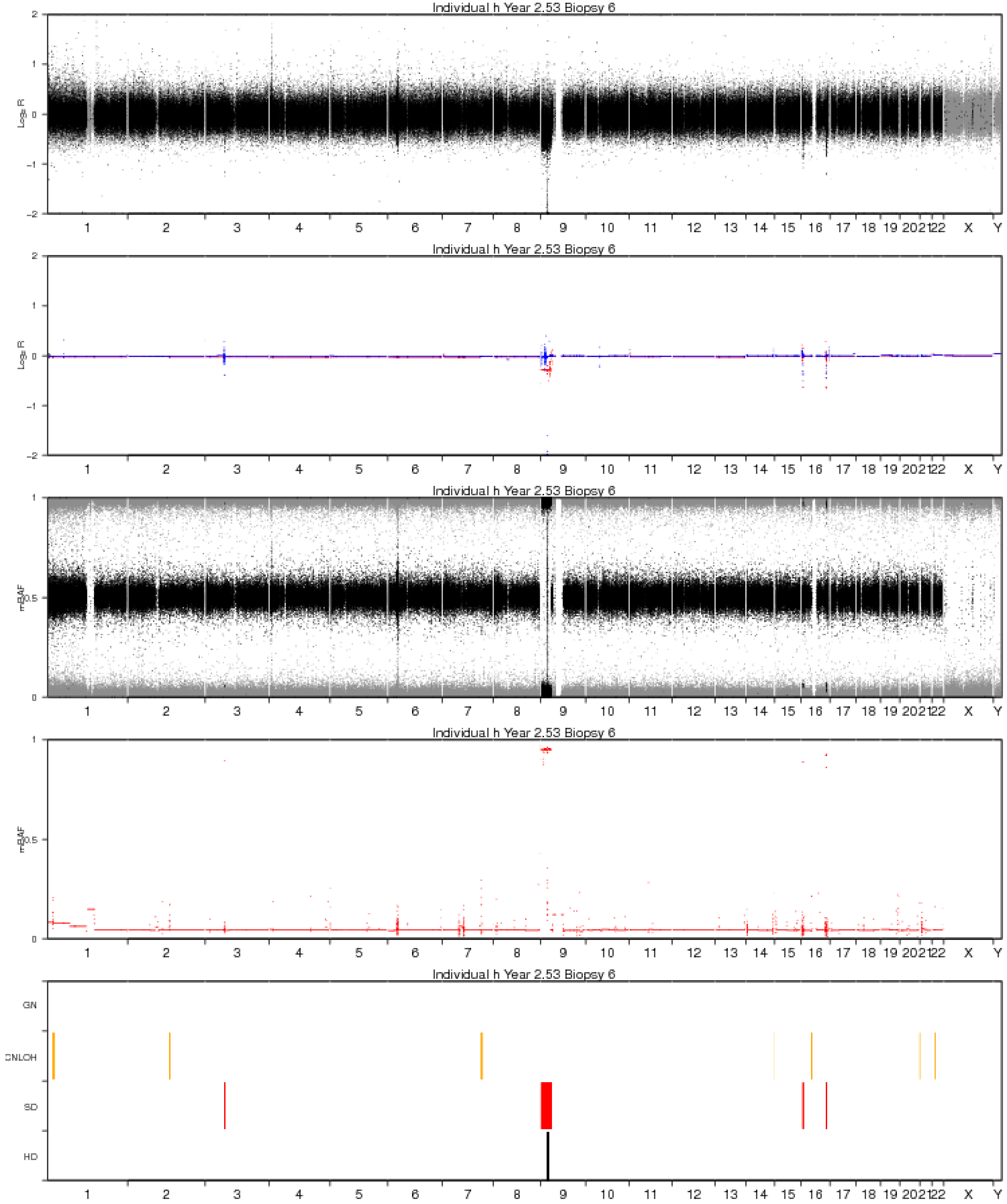
Individual h Year 2.53 Biopsy 6
Log R
2
1
0
−1
−2
1 2 3 4 5 6 7 8 9 10 11 12 13 14 15 16 17 18 19 20 21 22 X Y
Individual h Year 2.53 Biopsy 6
Log R
Individual h Year 2.53 Biopsy 6
mBAF
1
0.5
0
Individual h Year 2.53 Biopsy 6
mBAF
Individual h Year 2.53 Biopsy 6
GN
CNLOH
SD
HD

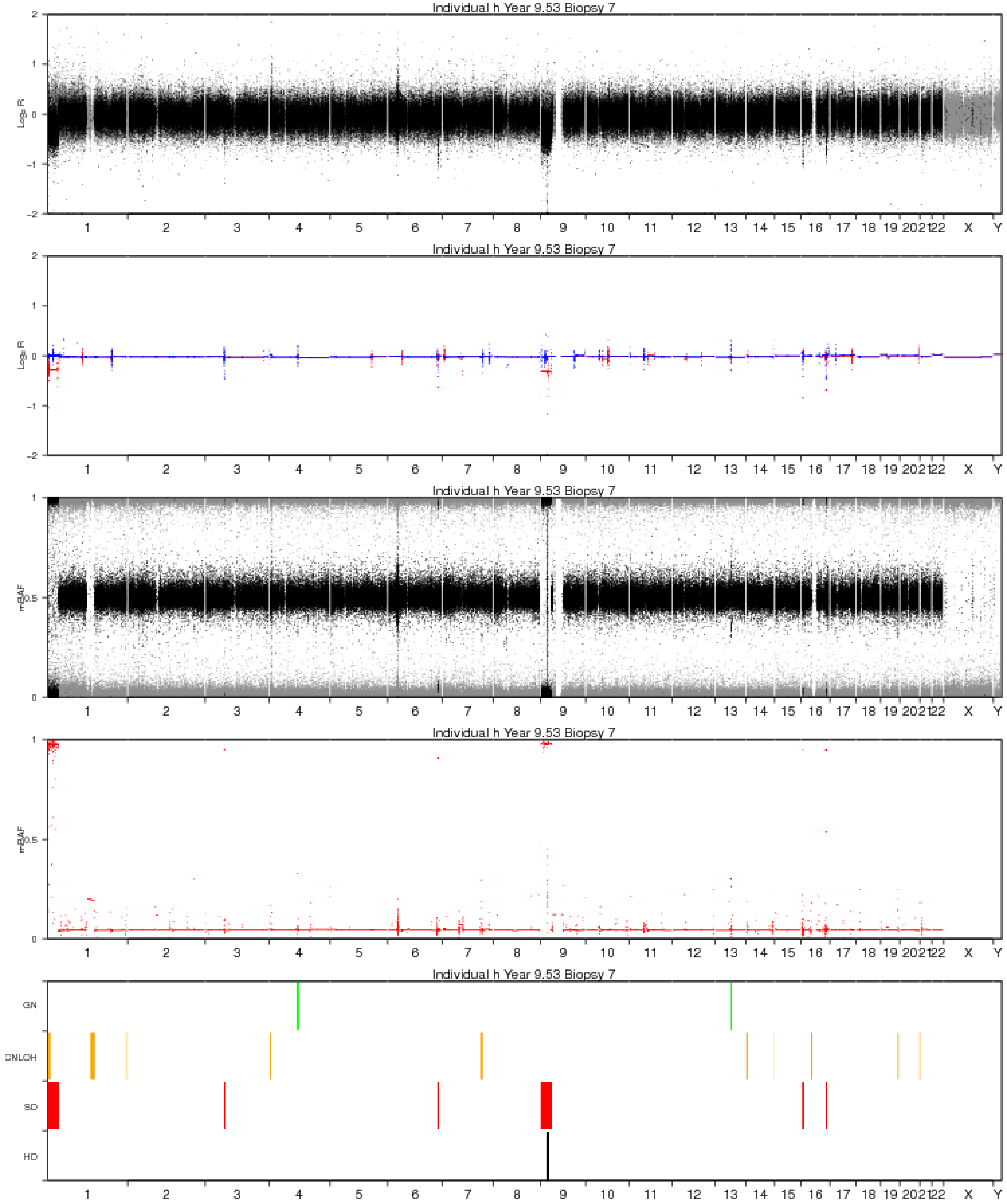

Individual h Year 9.53 Biopsy 7
Log R
2
1
0
−1
−2
1 2 3 4 5 6 7 8 9 10 11 12 13 14 15 16 17 18 19 20 21 22 X Y
Individual h Year 9.53 Biopsy 7
Log R
2
1
0
−1
−2
1 2 3 4 5 6 7 8 9 10 11 12 13 14 15 16 17 18 19 20 21 22 X Y
Individual h Year 9.53 Biopsy 7
mBAF
1
0.5
0
1 2 3 4 5 6 7 8 9 10 11 12 13 14 15 16 17 18 19 20 21 22 X Y
Individual h Year 9.53 Biopsy 7
mBAF
1
0.5
0
1 2 3 4 5 6 7 8 9 10 11 12 13 14 15 16 17 18 19 20 21 22 X Y
Individual h Year 9.53 Biopsy 7
GN
CNLOH
SD
HD
1 2 3 4 5 6 7 8 9 10 11 12 13 14 15 16 17 18 19 20 21 22 X Y

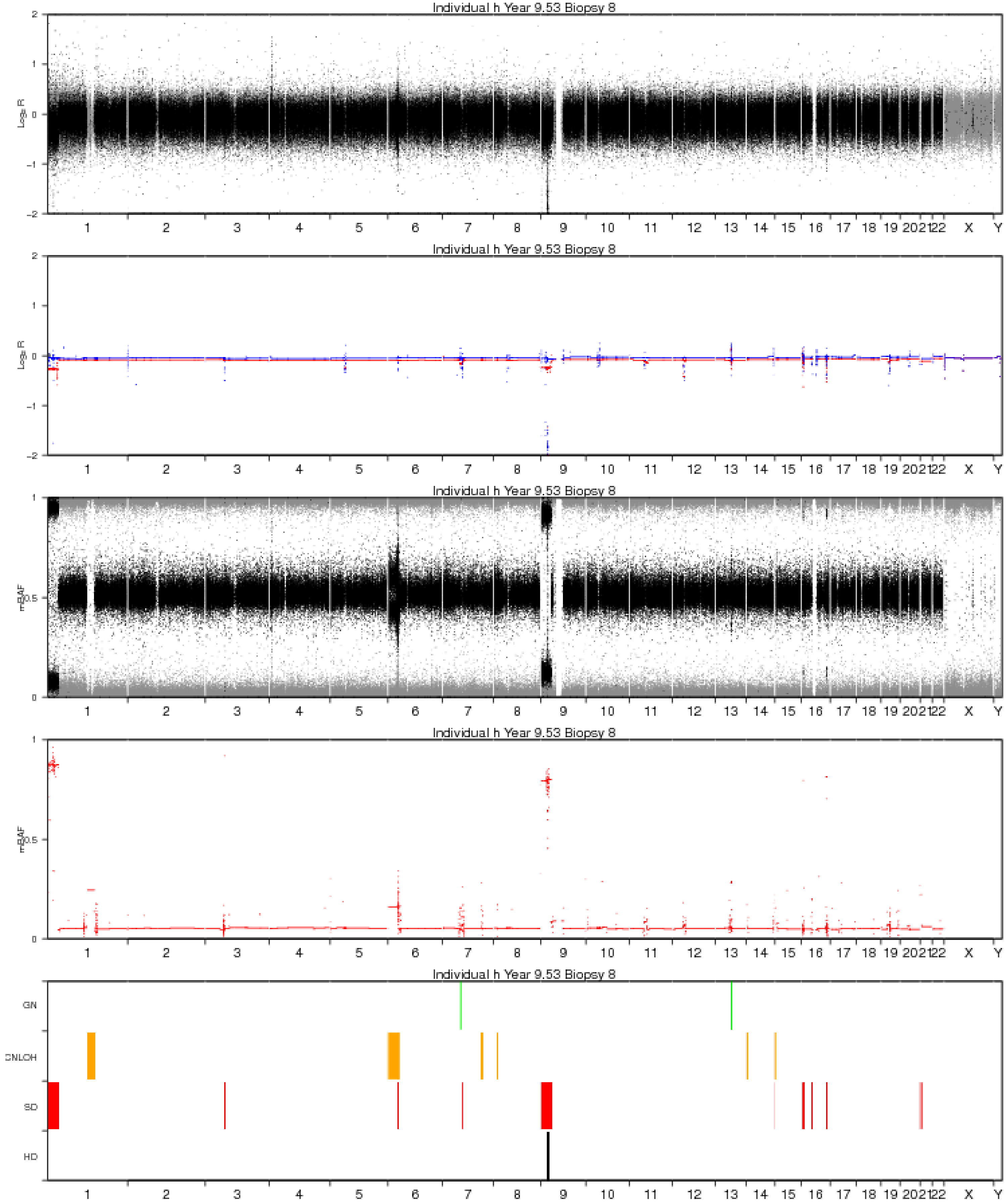
Individual h Year 9.53 Biopsy 8
Log R
mBAF
GN
CNLOH
SD
HD
1 2 3 4 5 6 7 8 9 10 11 12 13 14 15 16 17 18 19 20 21 22 X Y

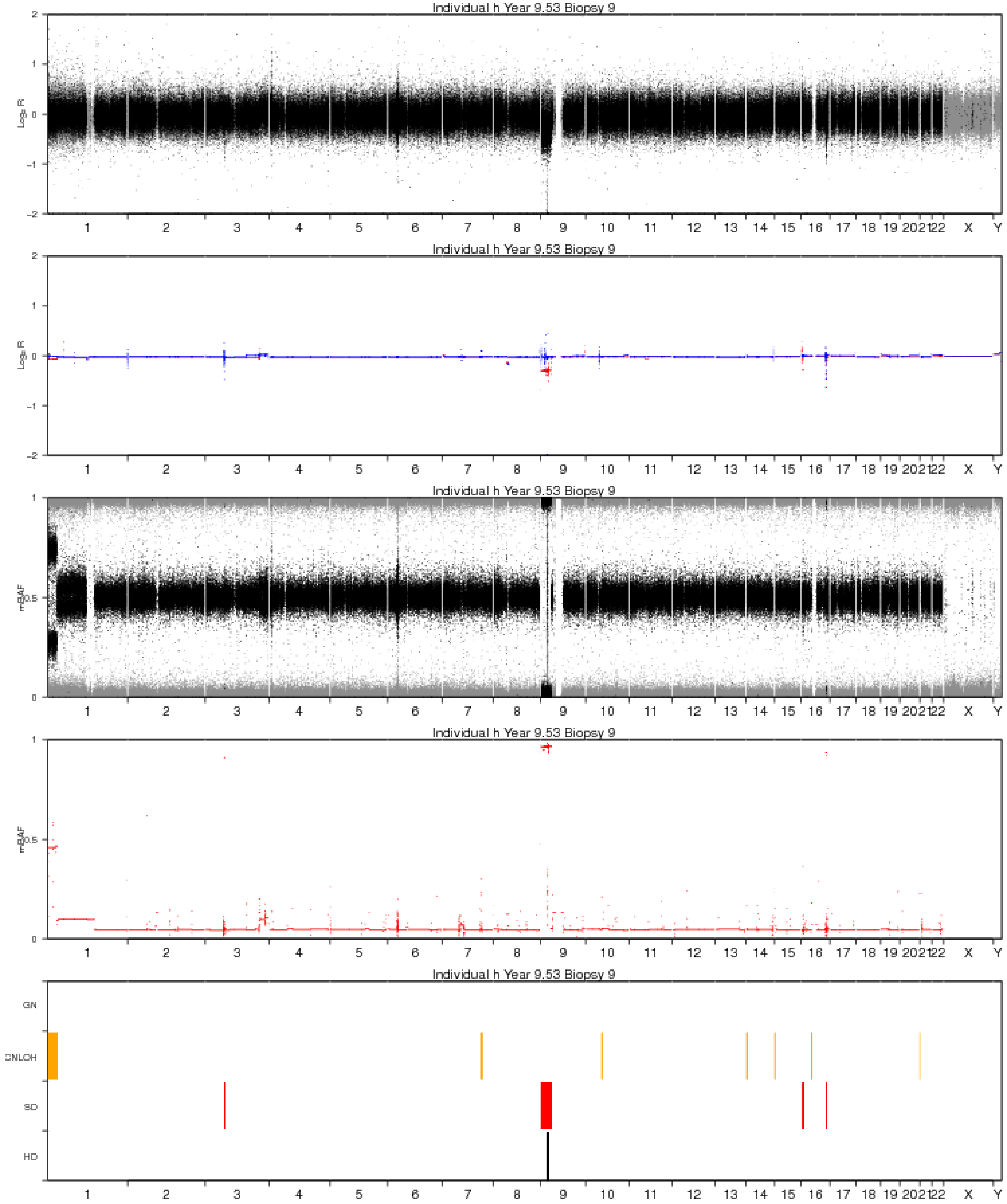
Individual h Year 9.53 Biopsy 9
Log R
mBAF
GN
CNLOH
SD
HD
1 2 3 4 5 6 7 8 9 10 11 12 13 14 15 16 17 18 19 20 21 22 X Y

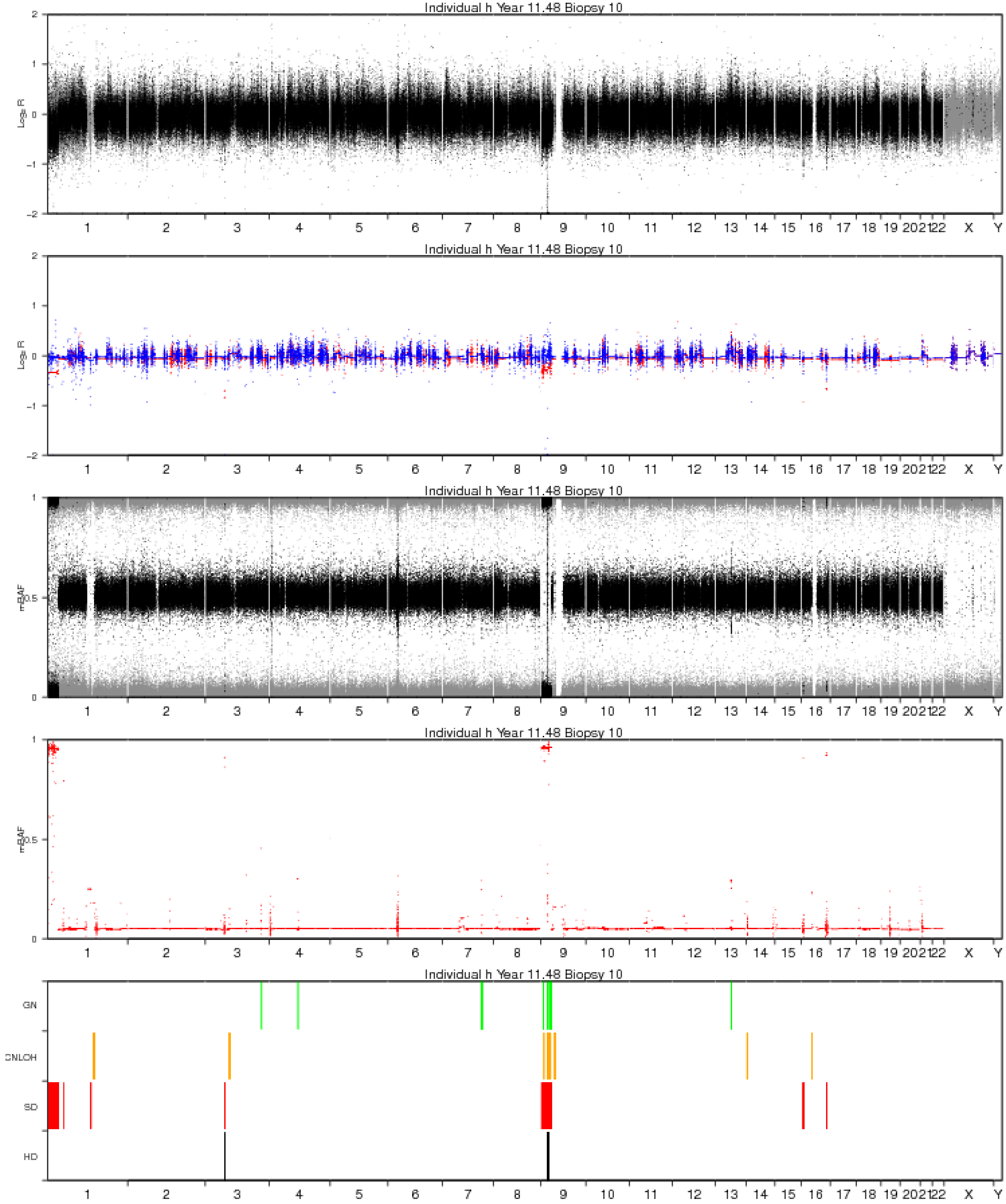
Individual h Year 11.48 Biopsy 10
Log R
mBAF
GN
CNLOH
SD
HD
1 2 3 4 5 6 7 8 9 10 11 12 13 14 15 16 17 18 19 20 21 22 X Y

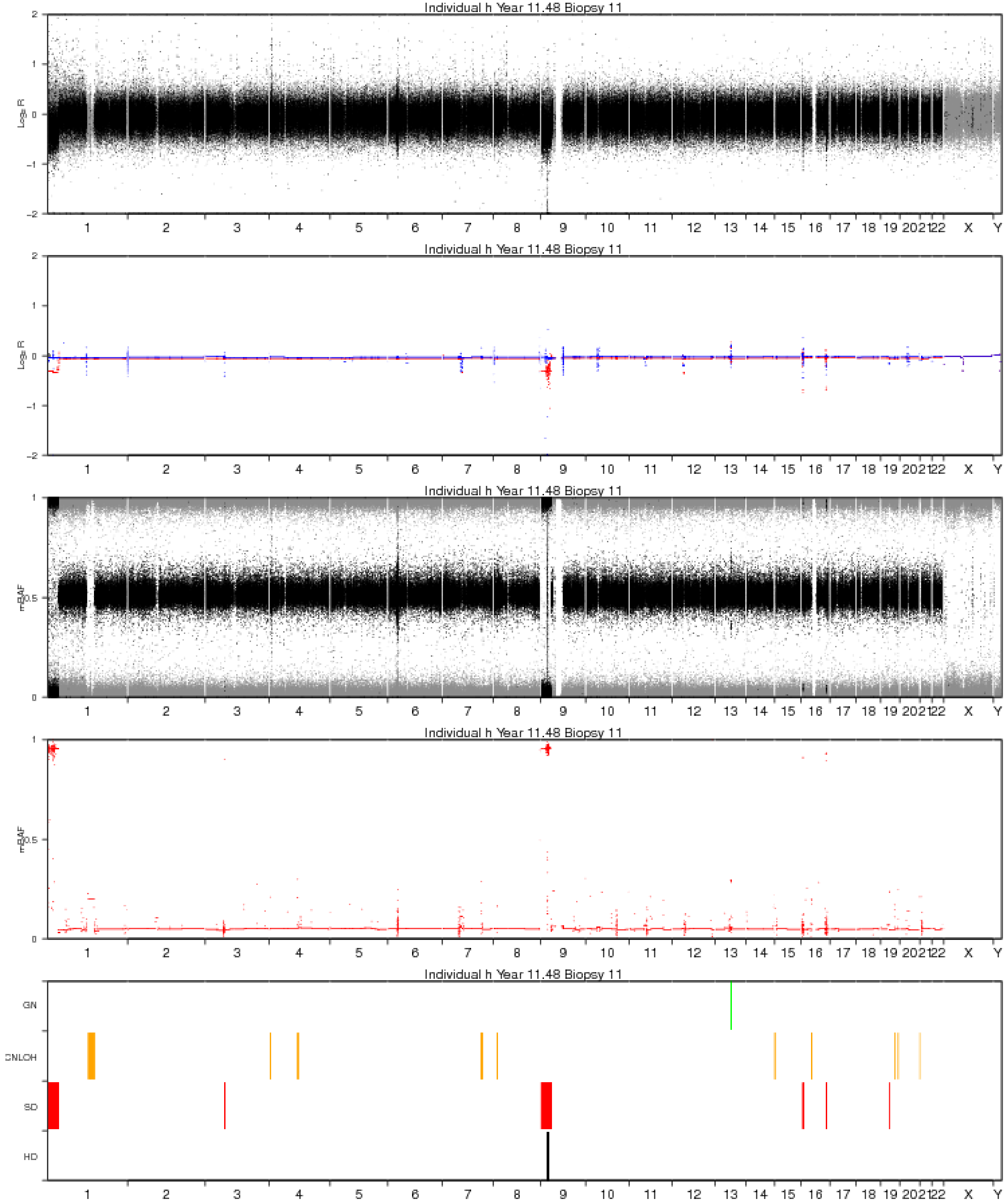
Individual h Year 11.48 Biopsy 11
Log R
mBAF
GN
CNLOH
SD
HD
1 2 3 4 5 6 7 8 9 10 11 12 13 14 15 16 17 18 19 20 21 22 X Y

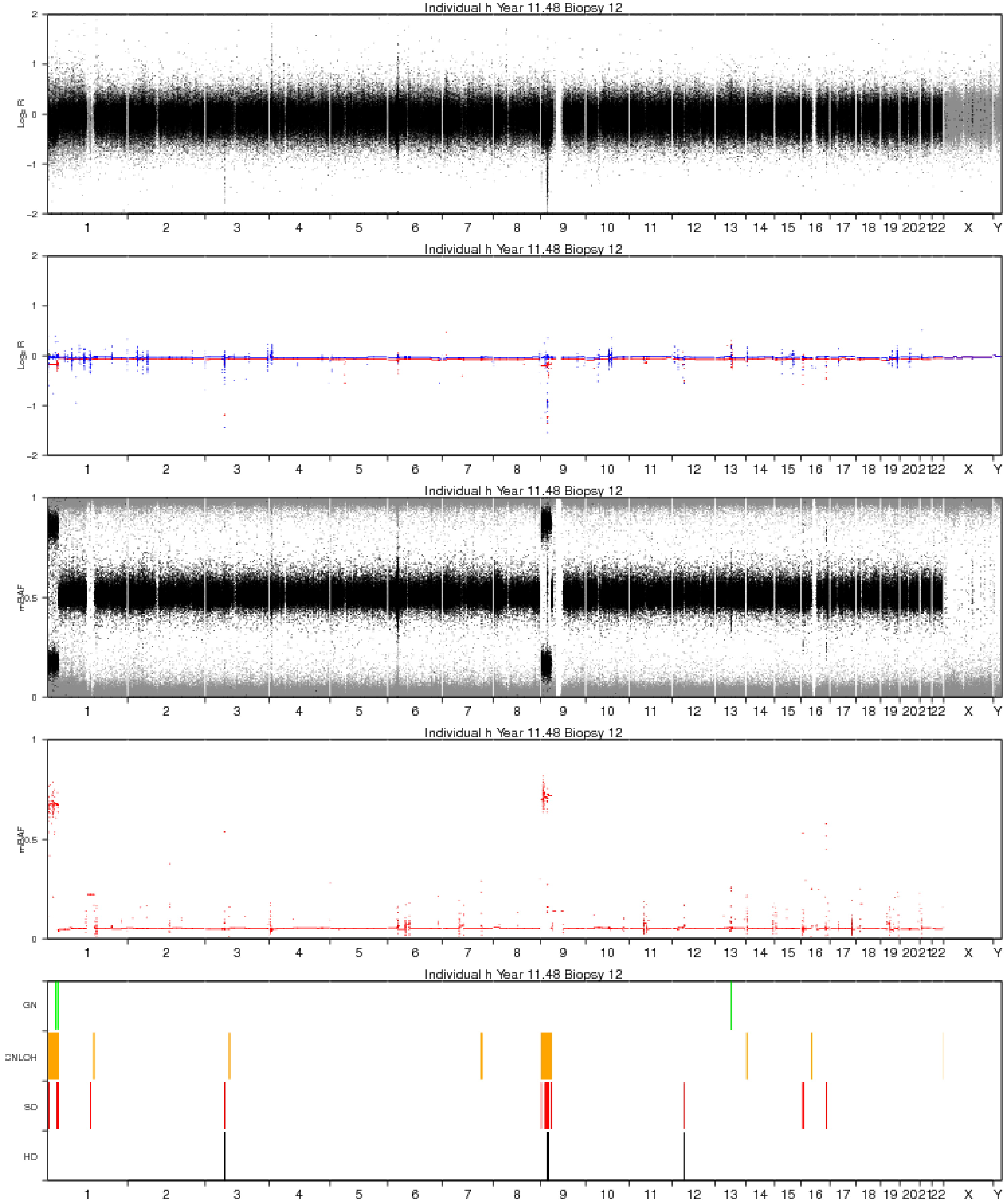
Individual h Year 11.48 Biopsy 12
Log R
mBAF
GN
CNLOH
SD
HD
1 2 3 4 5 6 7 8 9 10 11 12 13 14 15 16 17 18 19 20 21 22 X Y

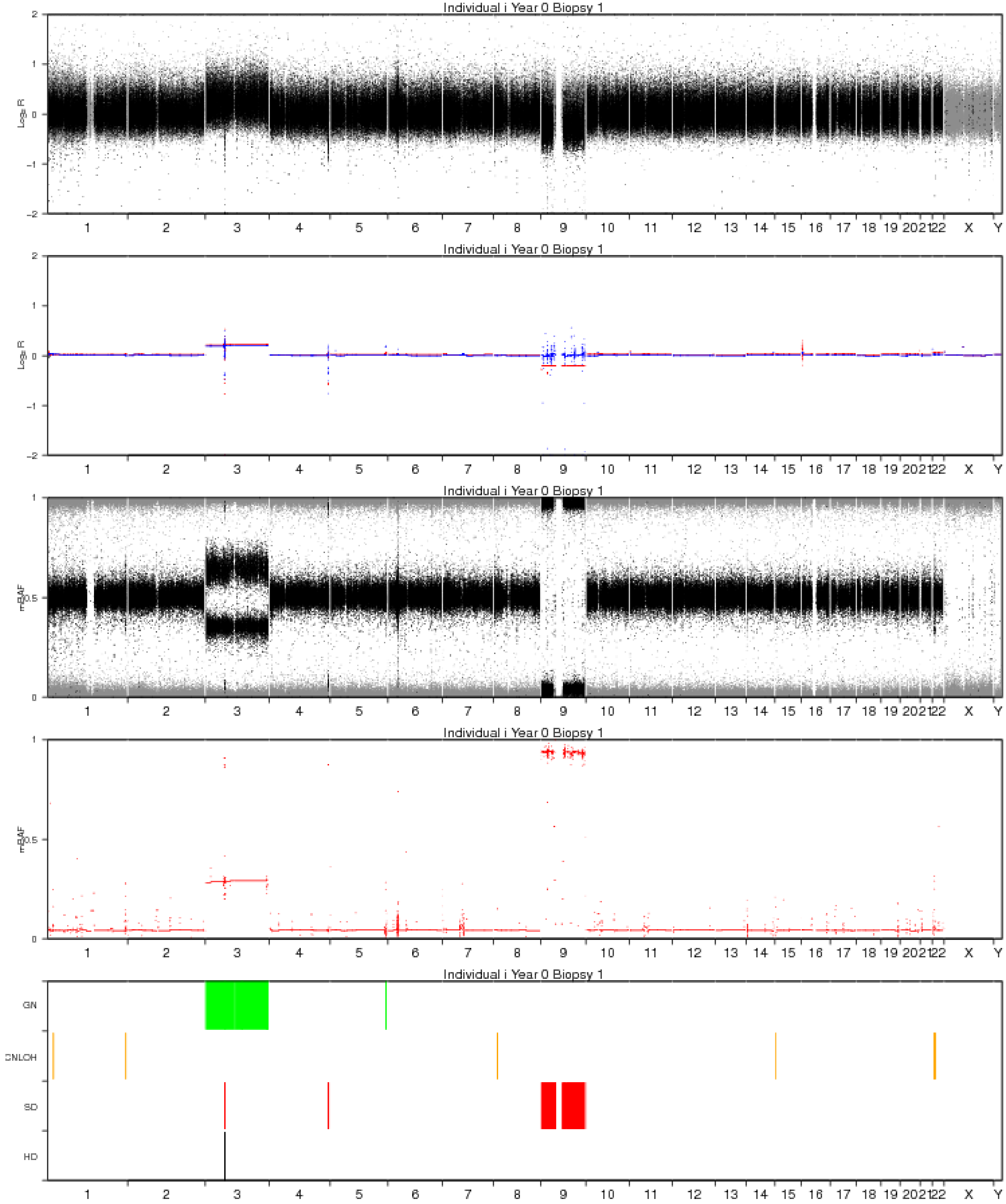
Individual i Year 0 Biopsy 1
Log R
mBAF
GN
CNLOH
SD
HD
1 2 3 4 5 6 7 8 9 10 11 12 13 14 15 16 17 18 19 20 21 22 X Y

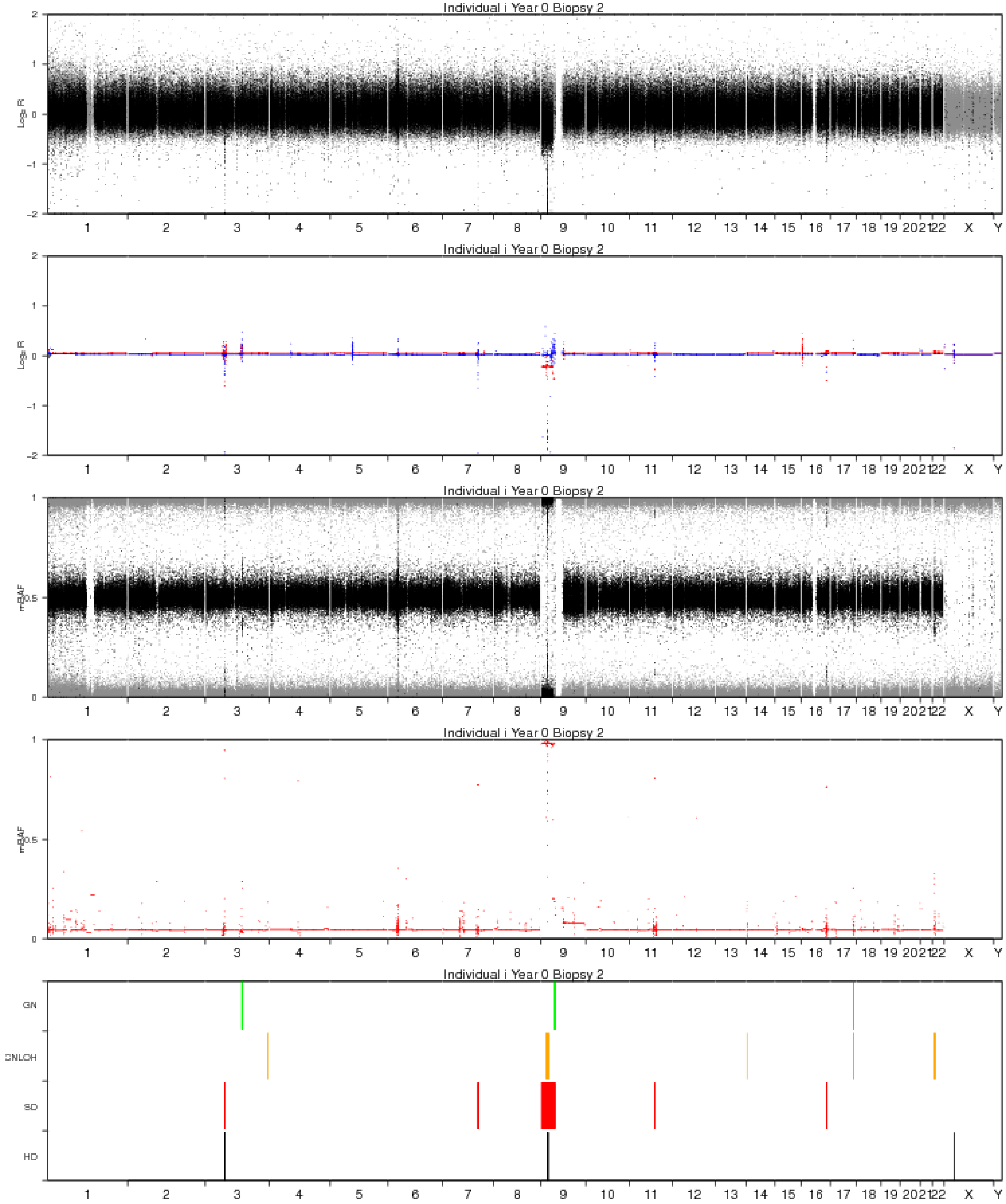
Individual i Year 0 Biopsy 2
Log R
mBAF
GN
CNLOH
SD
HD
1 2 3 4 5 6 7 8 9 10 11 12 13 14 15 16 17 18 19 20 21 22 X Y

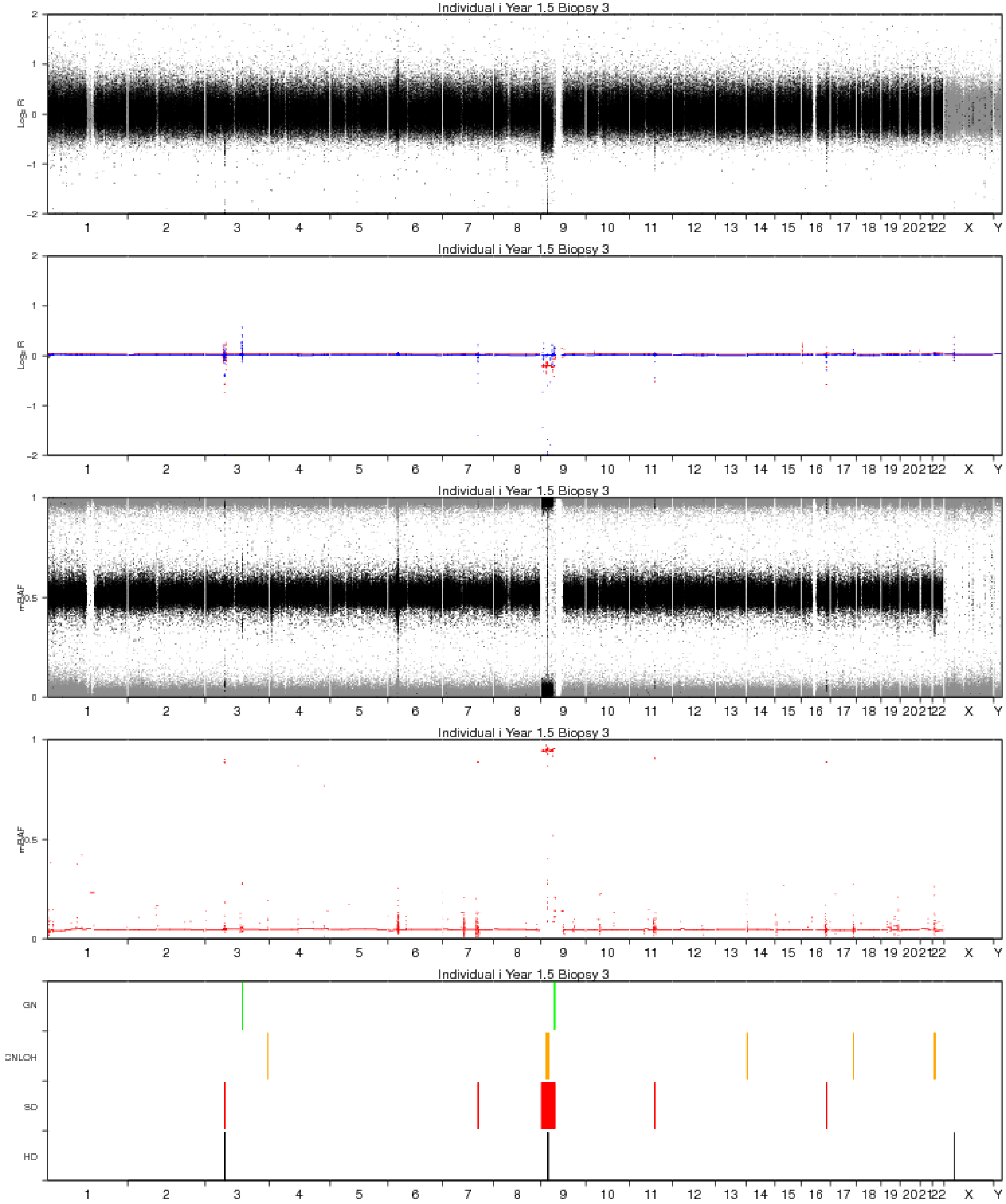

Individual i Year 1.5 Biopsy 3
Log2 R
mBAF
GN
CNLOH
SD
HD
1 2 3 4 5 6 7 8 9 10 11 12 13 14 15 16 17 18 19 20 21 22 X Y

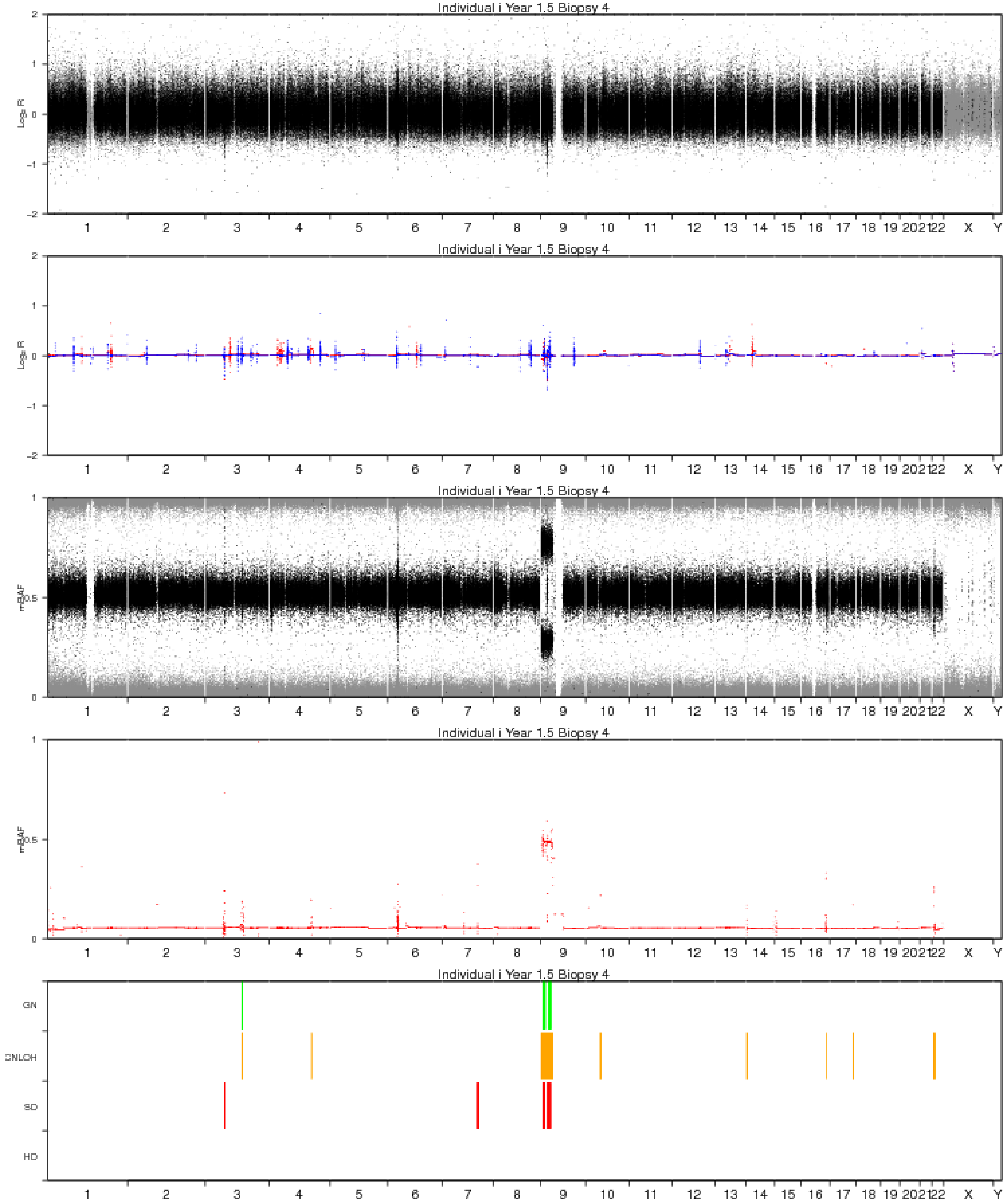
Individual i Year 1.5 Biopsy 4
Log₂ R
mBAF
1 2 3 4 5 6 7 8 9 10 11 12 13 14 15 16 17 18 19 20 21 22 X Y
GN
CNLOH
SD
HD

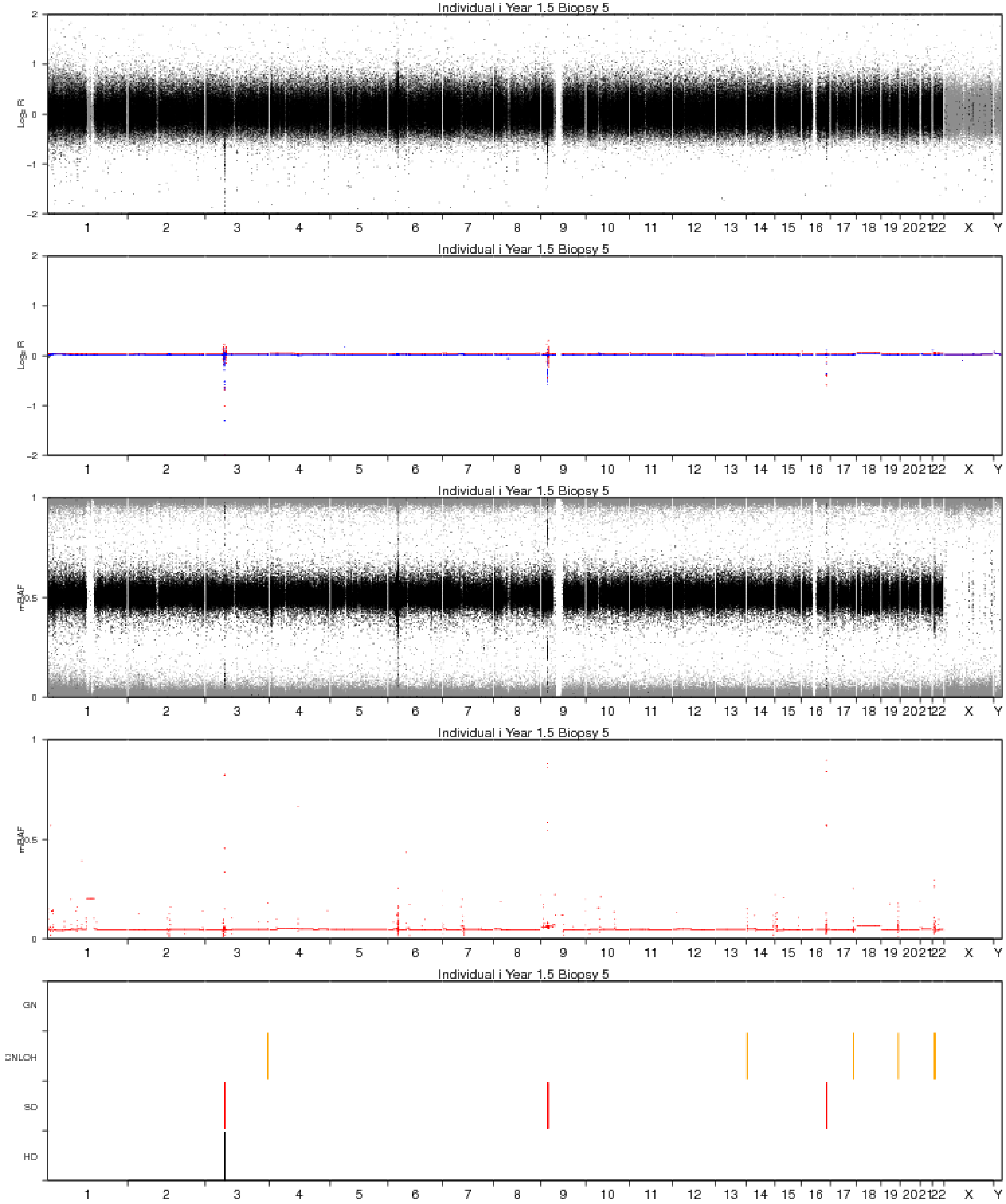

Individual i Year 1.5 Biopsy 5
Log R
mBAF
GN
CNLOH
SD
HD
1 2 3 4 5 6 7 8 9 10 11 12 13 14 15 16 17 18 19 20 21 22 X Y

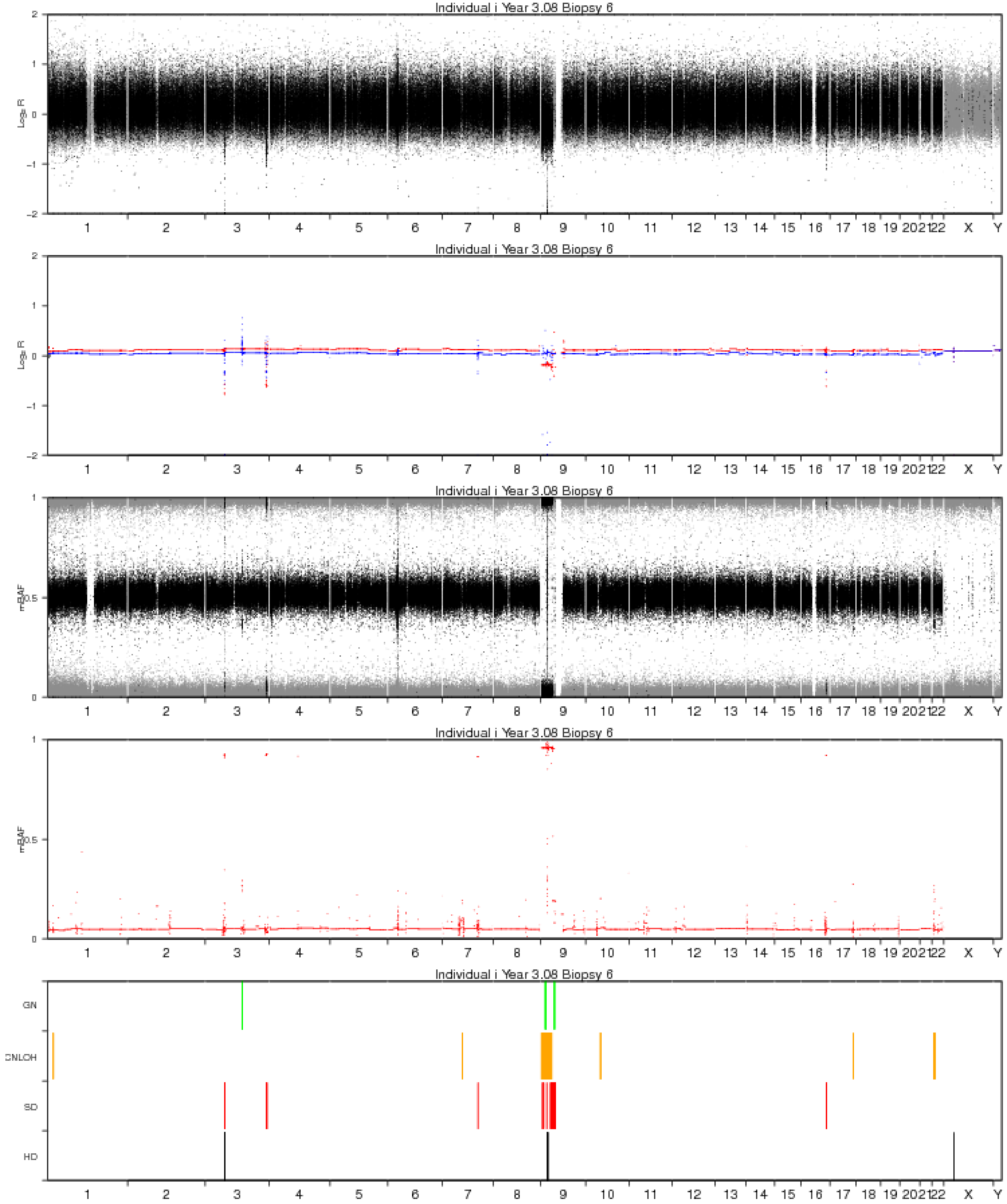
Individual i Year 3.08 Biopsy 6
Log2 R
2
1
0
−1
−2
1 2 3 4 5 6 7 8 9 10 11 12 13 14 15 16 17 18 19 20 21 22 X Y
Individual i Year 3.08 Biopsy 6
Log2 R
Individual i Year 3.08 Biopsy 6
mBAF
1
0.5
0
Individual i Year 3.08 Biopsy 6
mBAF
Individual i Year 3.08 Biopsy 6
GN
CNLOH
SD
HD

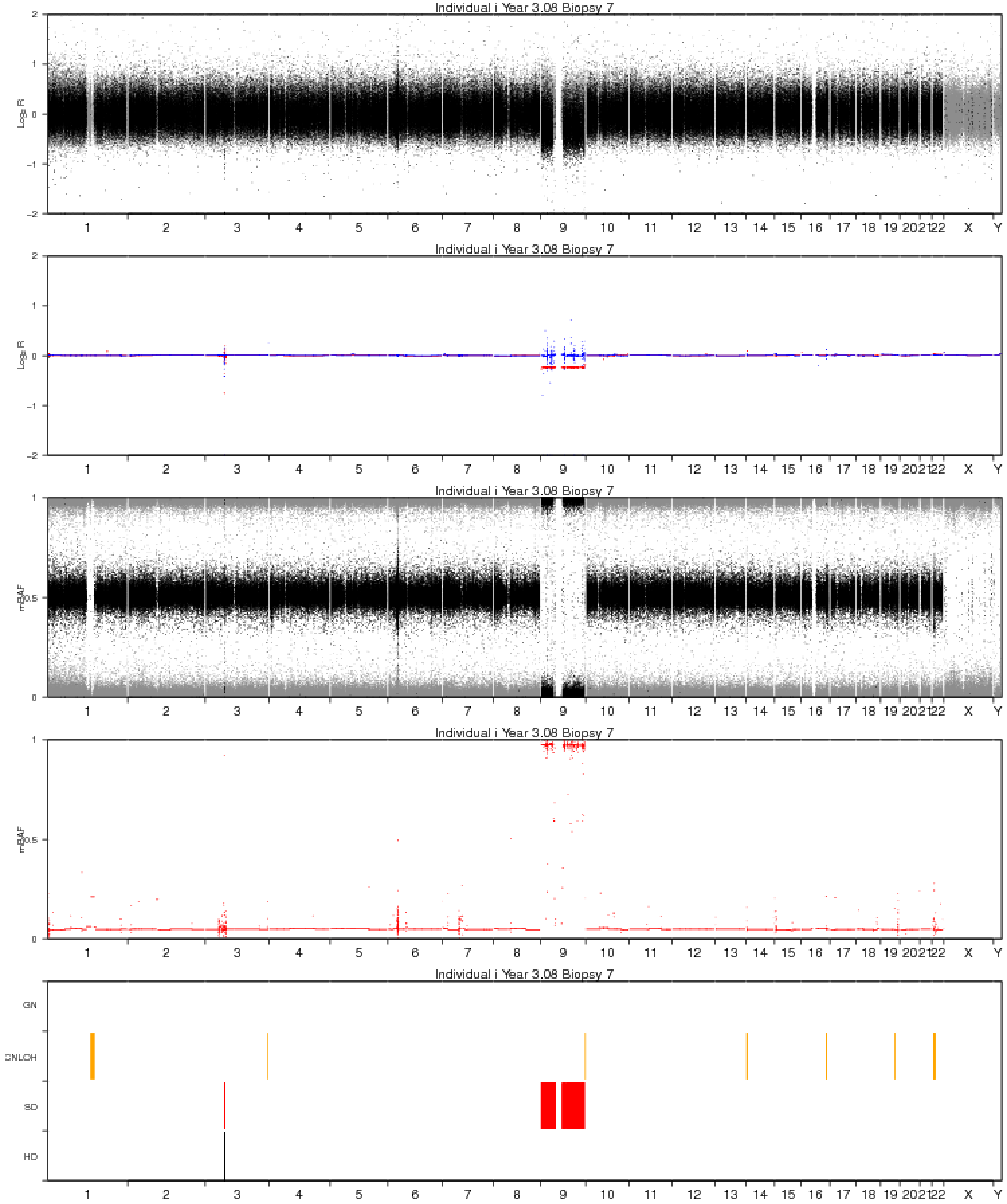
Individual i Year 3.08 Biopsy 7
Log R
mBAF
GN
CNLOH
SD
HD
1 2 3 4 5 6 7 8 9 10 11 12 13 14 15 16 17 18 19 20 21 22 X Y

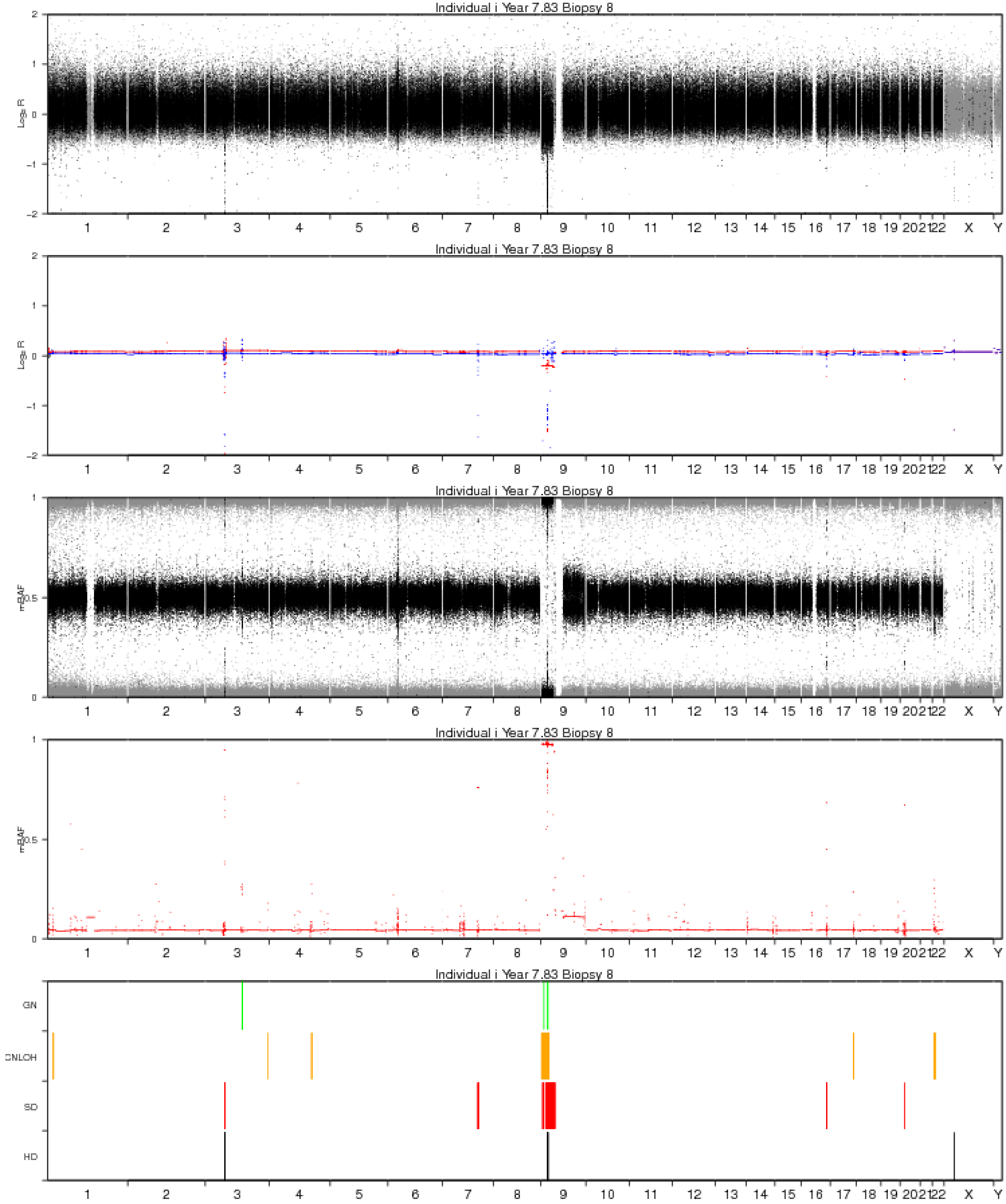

Individual i Year 7.83 Biopsy 8
Log R
mBAF
GN
CNLOH
SD
HD
1 2 3 4 5 6 7 8 9 10 11 12 13 14 15 16 17 18 19 20 21 22 X Y

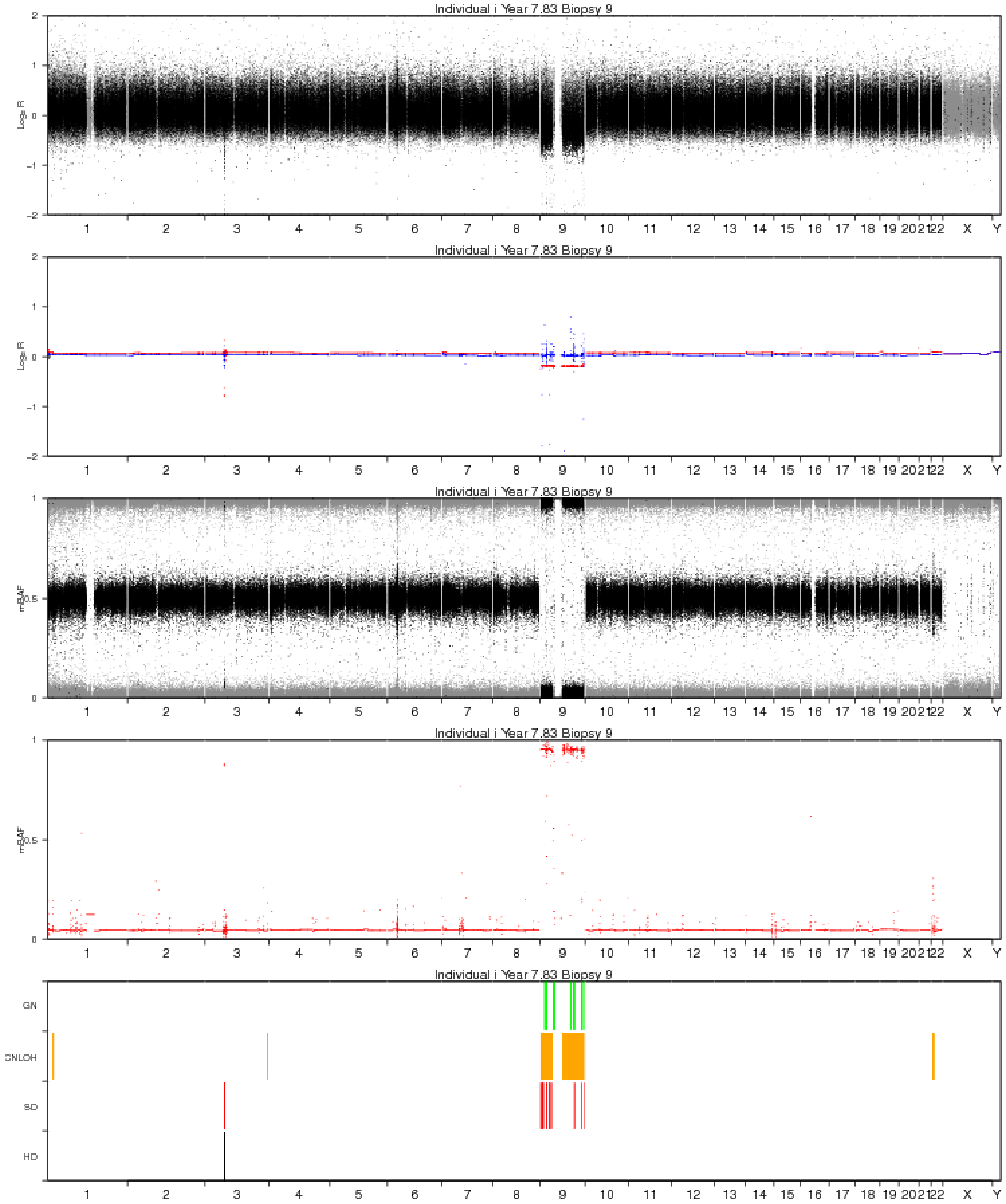
Individual i Year 7.83 Biopsy 9
Log R
mBAF
GN
CNLOH
SD
HD
1
2
3
4
5
6
7
8
9
10
11
12
13
14
15
16
17
18
19
20
21
22
X
Y

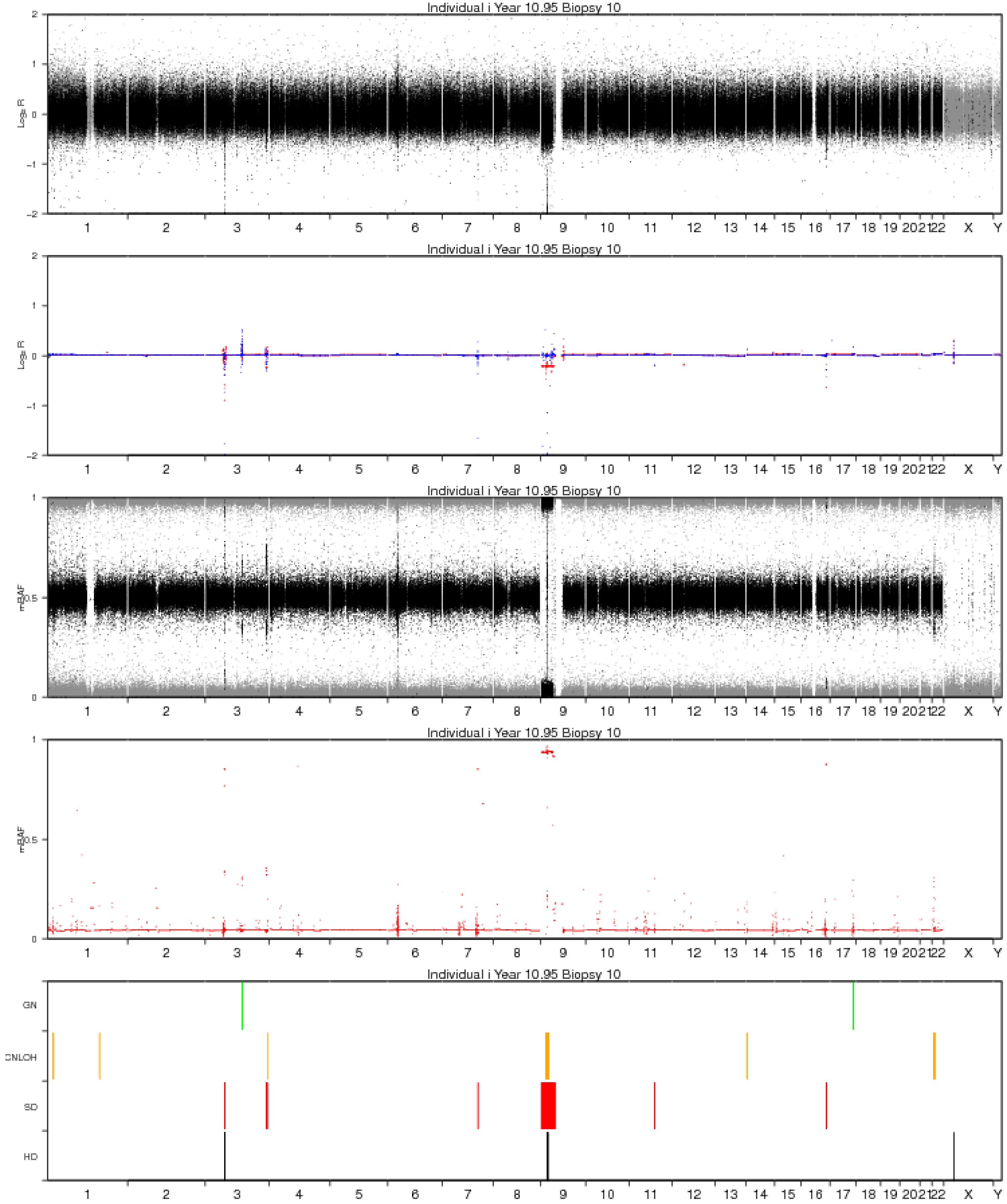
Individual i Year 10.95 Biopsy 10
Log R
mBAF
GN
CNLOH
SD
HD
1 2 3 4 5 6 7 8 9 10 11 12 13 14 15 16 17 18 19 20 21 22 X Y

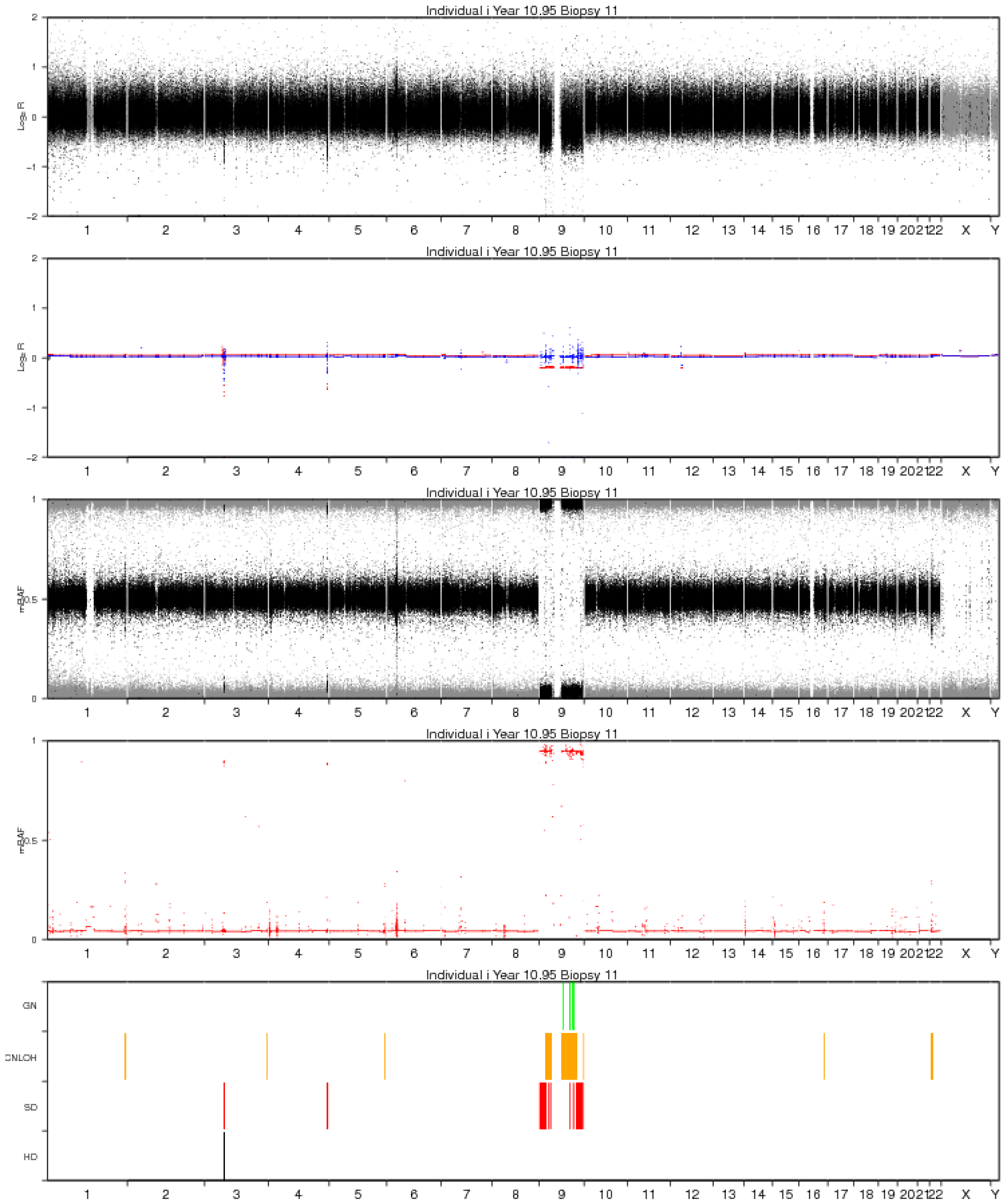

Individual i Year 10.95 Biopsy 11
Log R
mBAF
GN
CNLOH
SD
HD
1
2
3
4
5
6
7
8
9
10
11
12
13
14
15
16
17
18
19
20
21
22
X
Y

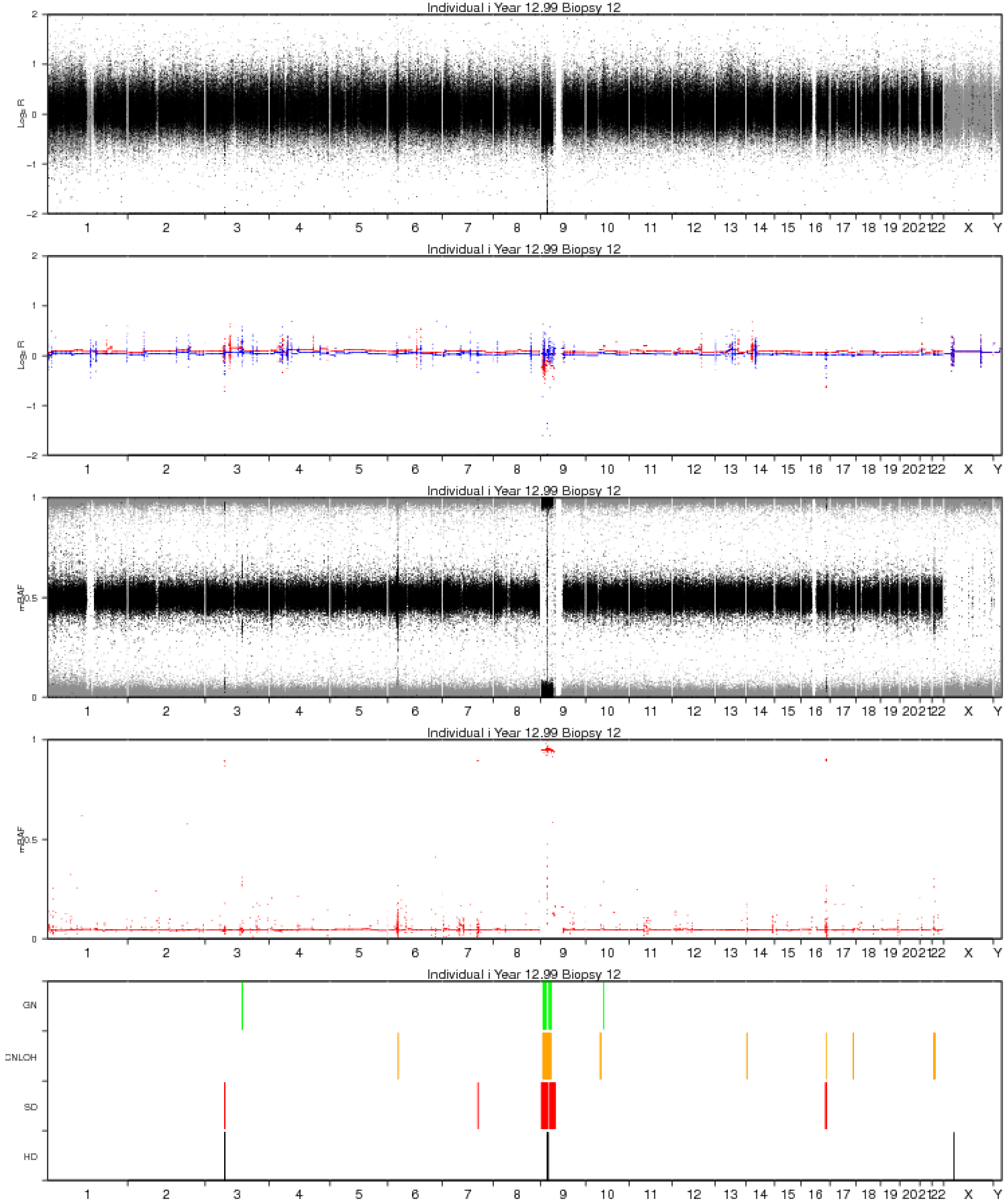
Individual i Year 12.99 Biopsy 12
Log R
mBAF
GN
CNLOH
SD
HD
1 2 3 4 5 6 7 8 9 10 11 12 13 14 15 16 17 18 19 20 21 22 X Y

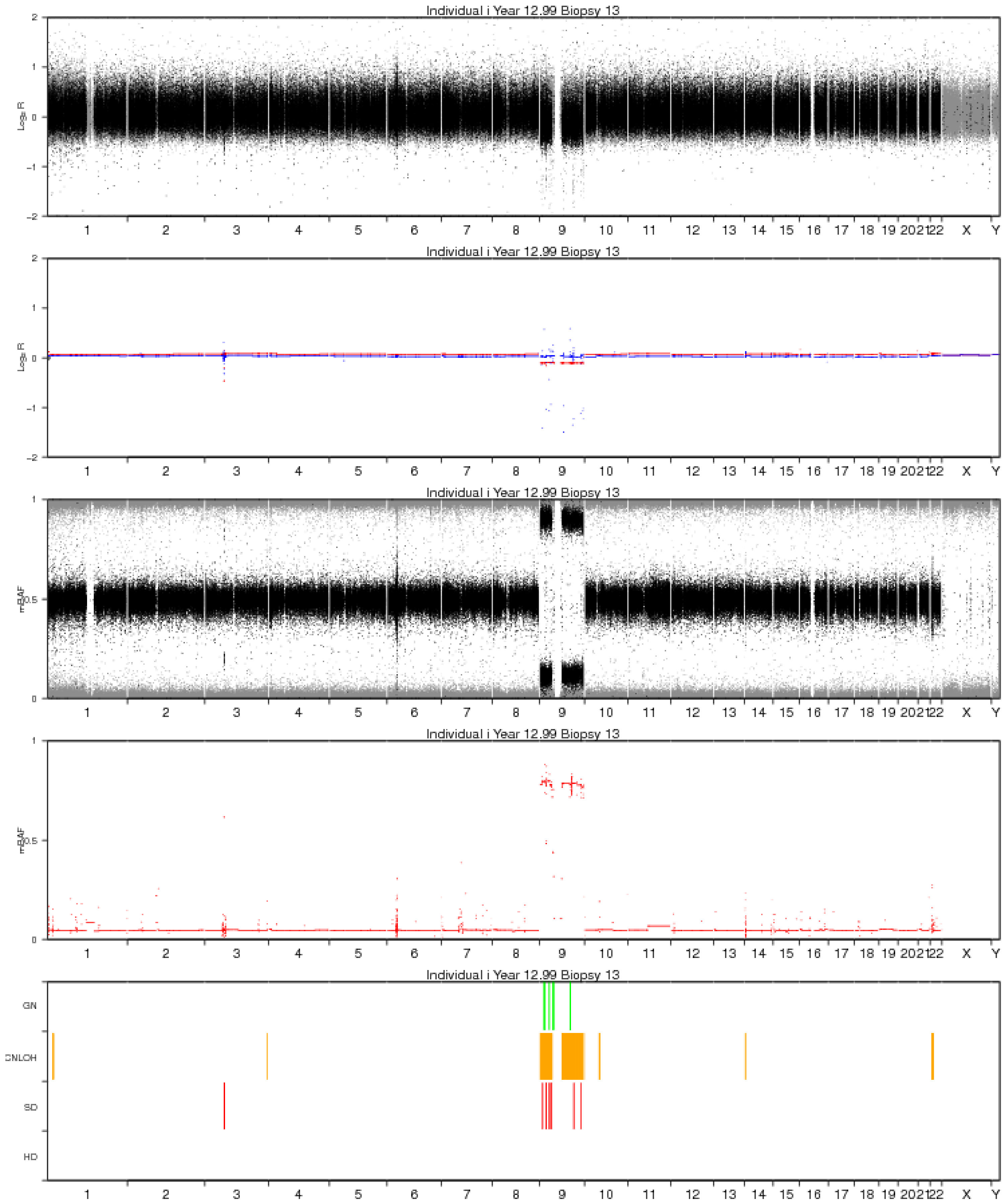
Individual i Year 12.99 Biopsy 13
Log R
mBAF
GN
CNLOH
SD
HD
1 2 3 4 5 6 7 8 9 10 11 12 13 14 15 16 17 18 19 20 21 22 X Y

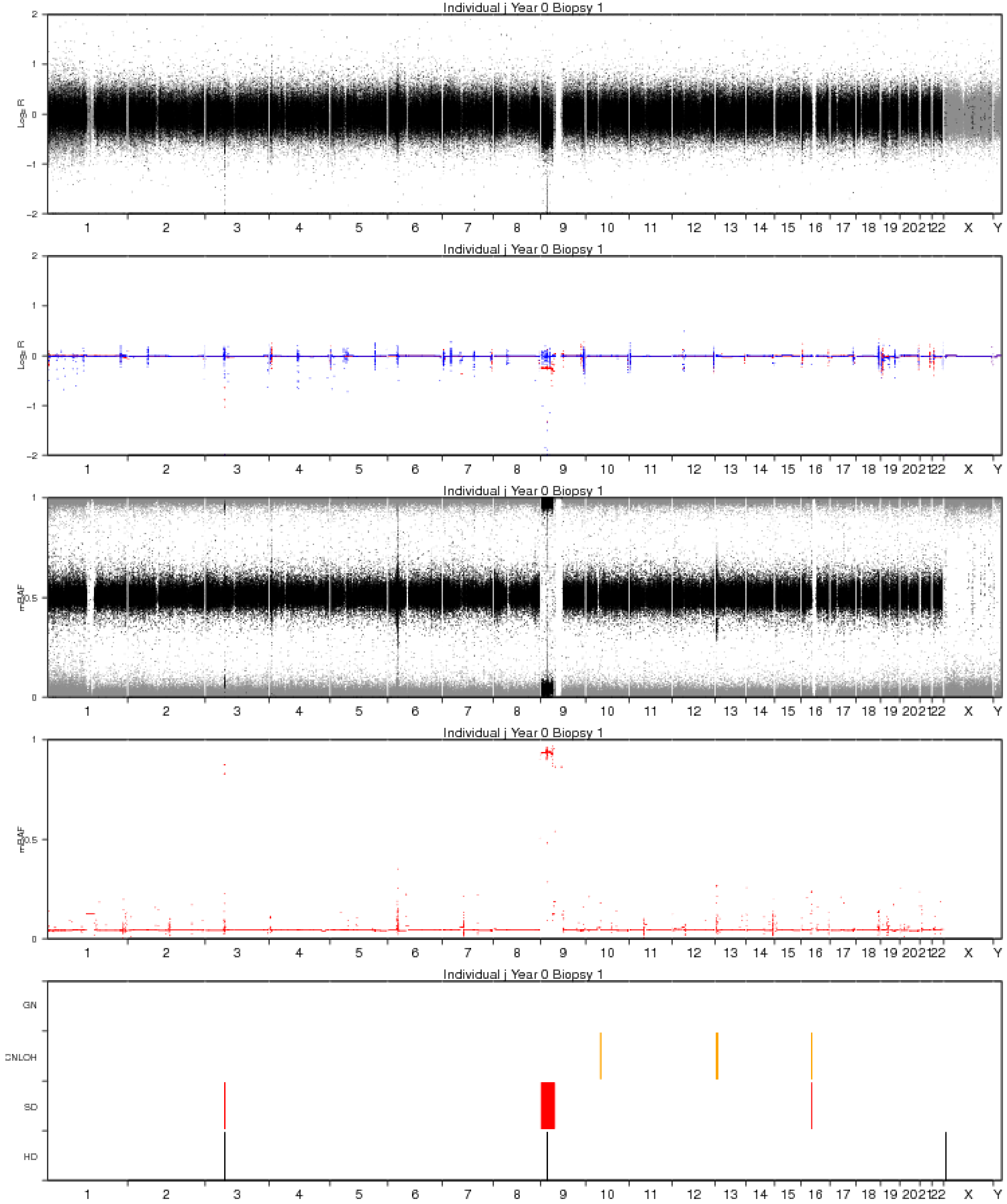

Individual j Year 0 Biopsy 1
Log2 R
mBAF
GN
CNLOH
SD
HD
1 2 3 4 5 6 7 8 9 10 11 12 13 14 15 16 17 18 19 20 21 22 X Y

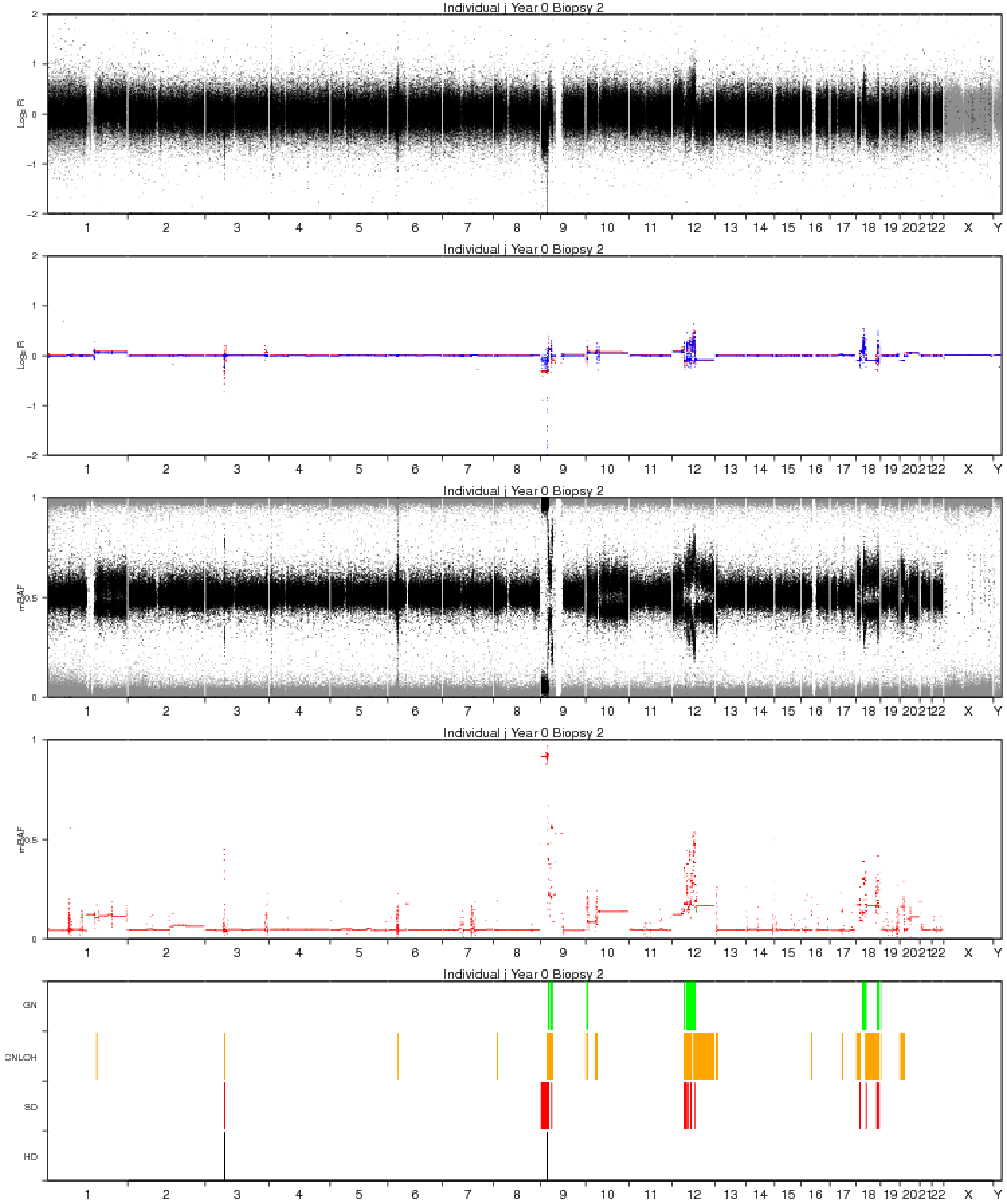
Individual j Year 0 Biopsy 2
Log R
mBAF
GN
CNLOH
SD
HD
1
2
3
4
5
6
7
8
9
10
11
12
13
14
15
16
17
18
19
20
21
22
X
Y

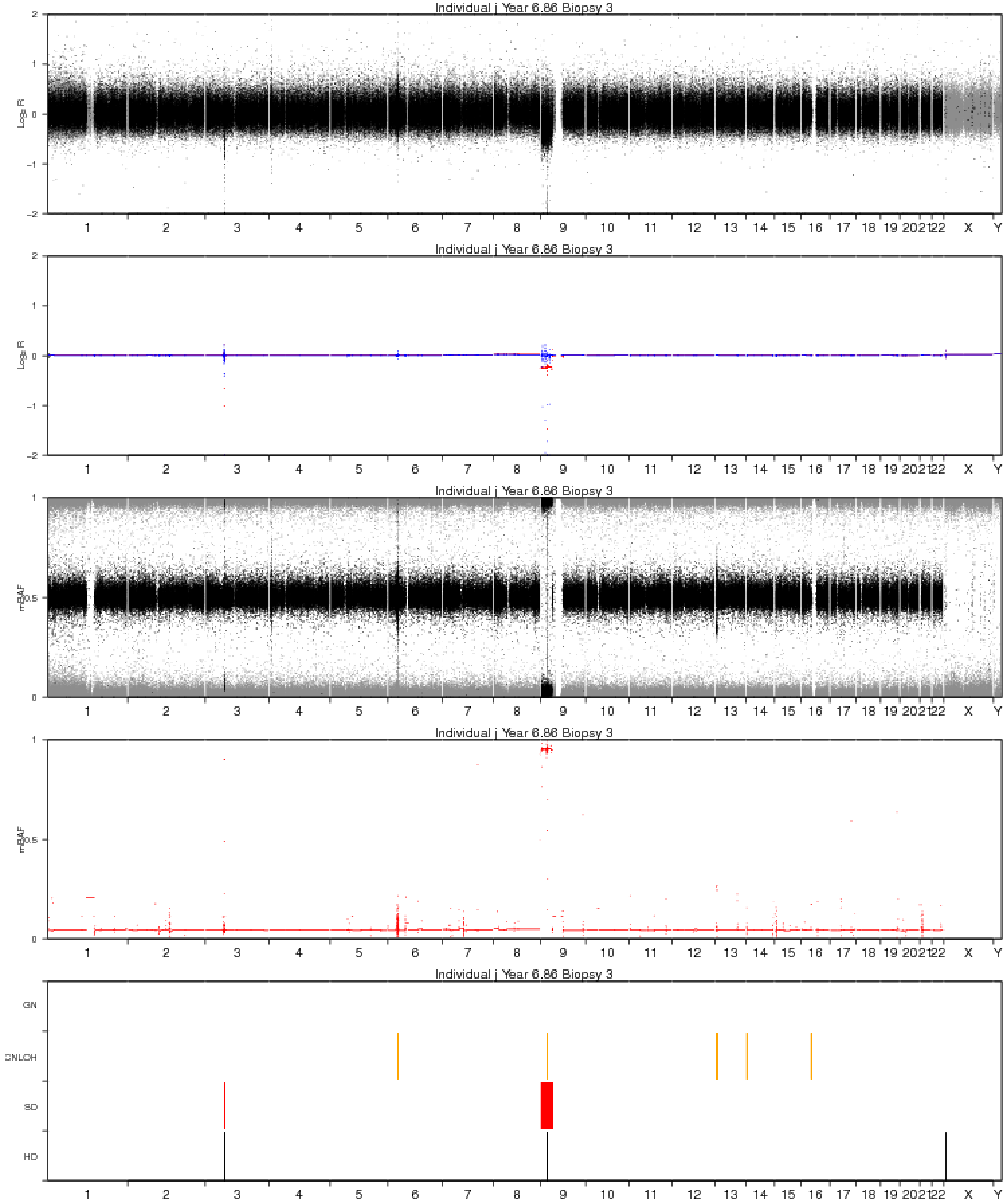

Individual j Year 6.86 Biopsy 3
Log R
mBAF
GN
CNLOH
SD
HD
1 2 3 4 5 6 7 8 9 10 11 12 13 14 15 16 17 18 19 20 21 22 X Y

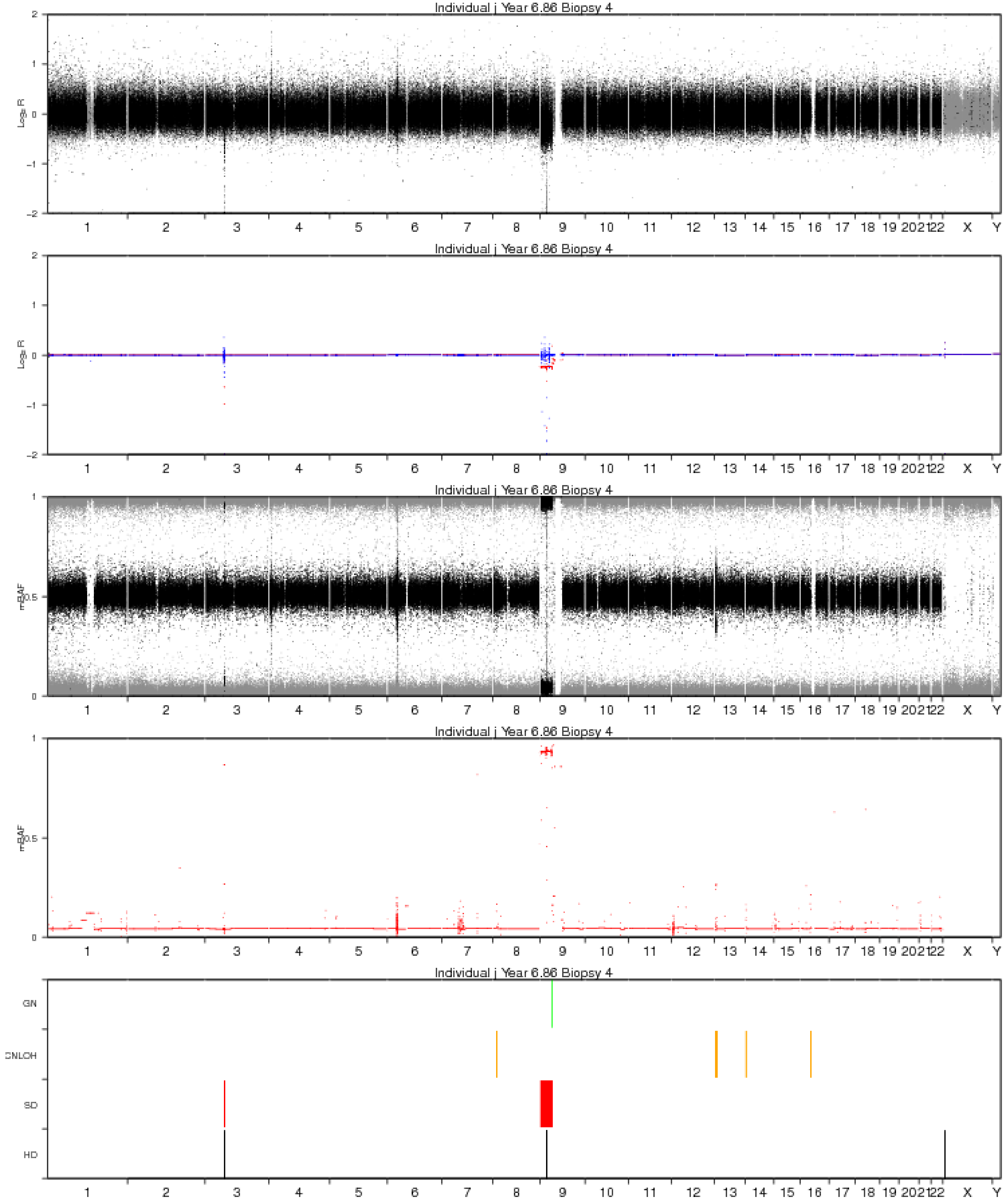
Individual j Year 6.86 Biopsy 4
Log R
mBAF
GN
CNLOH
SD
HD
1 2 3 4 5 6 7 8 9 10 11 12 13 14 15 16 17 18 19 20 21 22 X Y

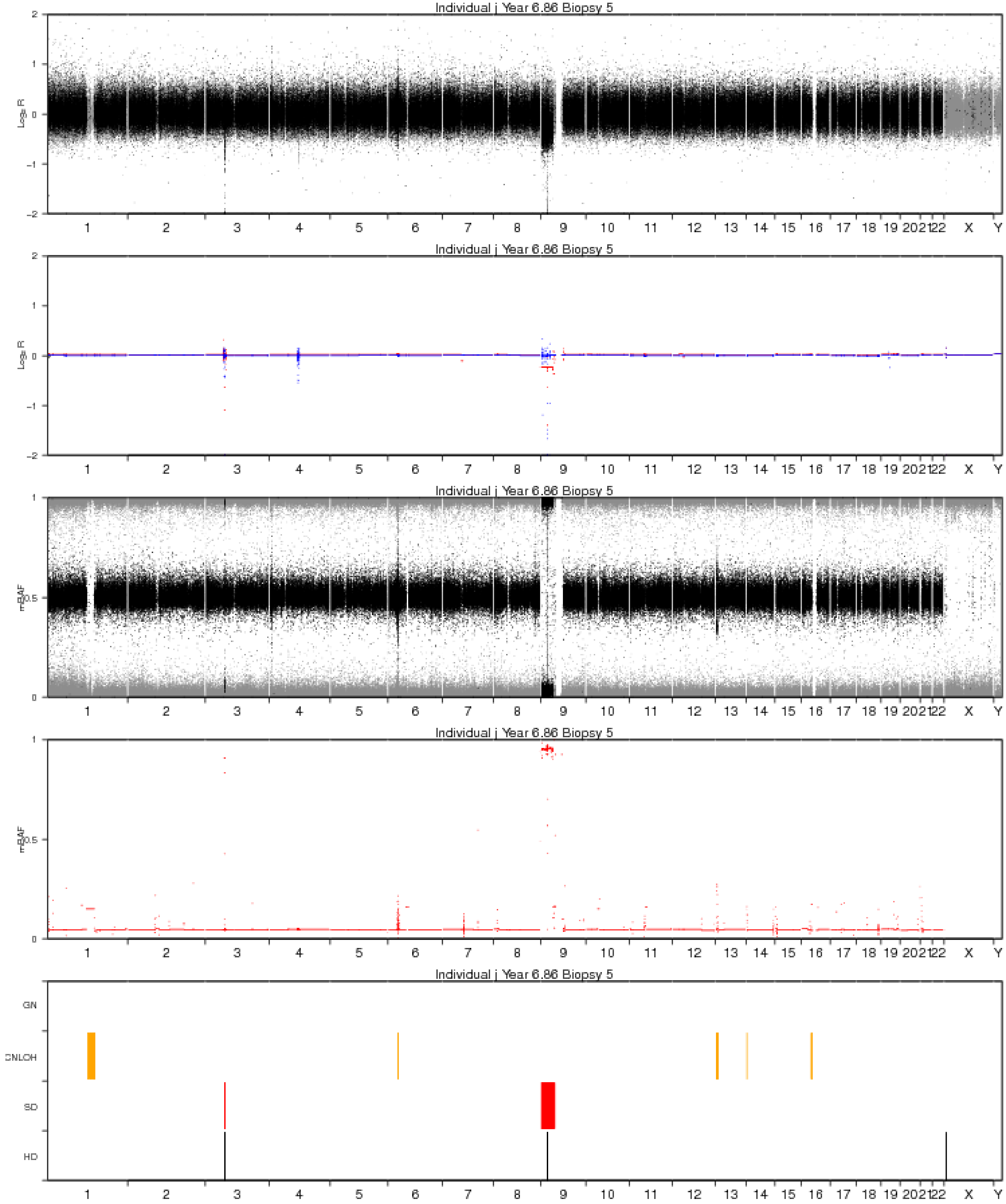

Individual j Year 6.86 Biopsy 5
Log R
mBAF
GN
CNLOH
SD
HD
1 2 3 4 5 6 7 8 9 10 11 12 13 14 15 16 17 18 19 20 21 22 X Y

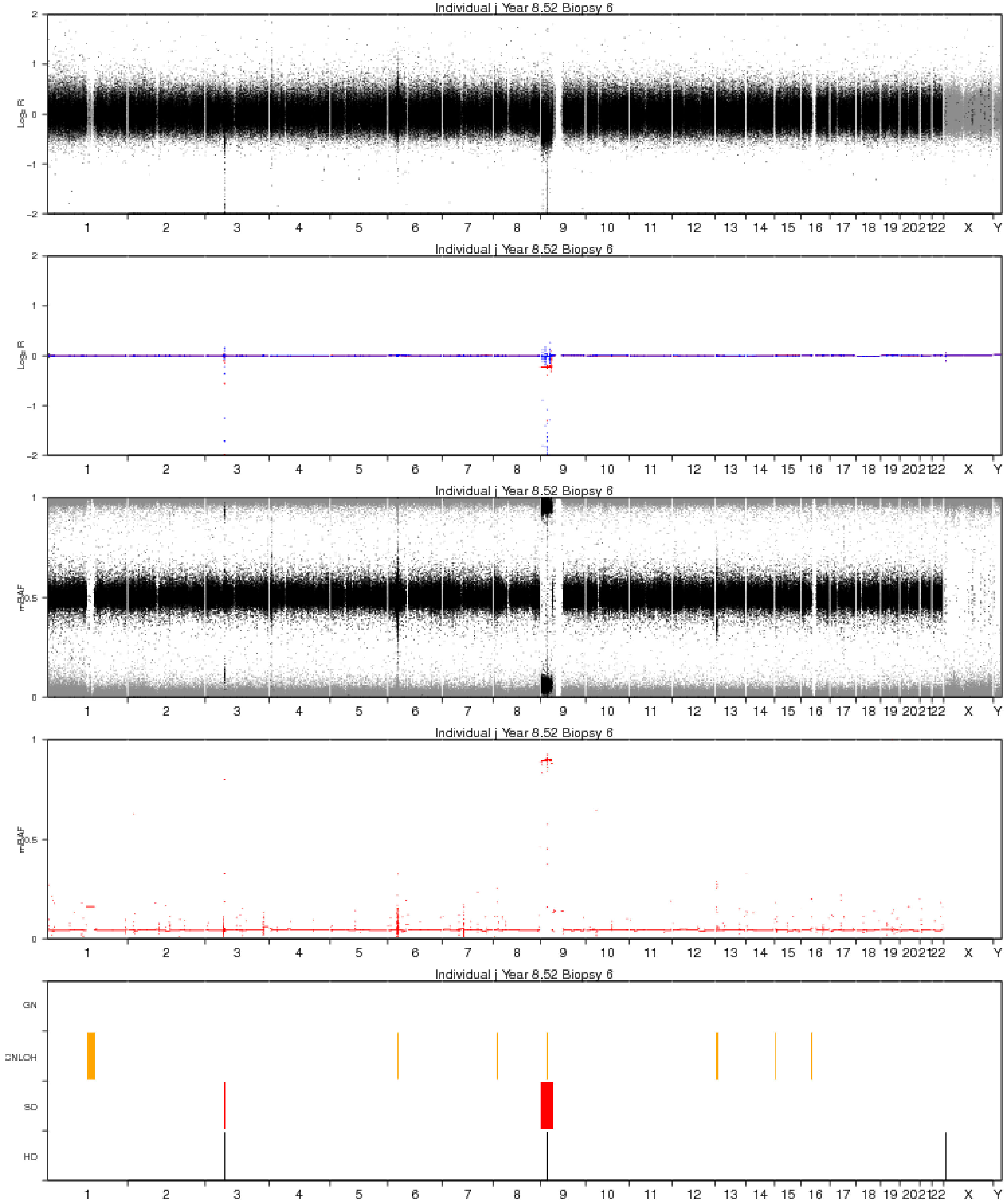

Individual j Year 8.52 Biopsy 6
Log R
mBAF
GN
CNLOH
SD
HD
1 2 3 4 5 6 7 8 9 10 11 12 13 14 15 16 17 18 19 20 21 22 X Y

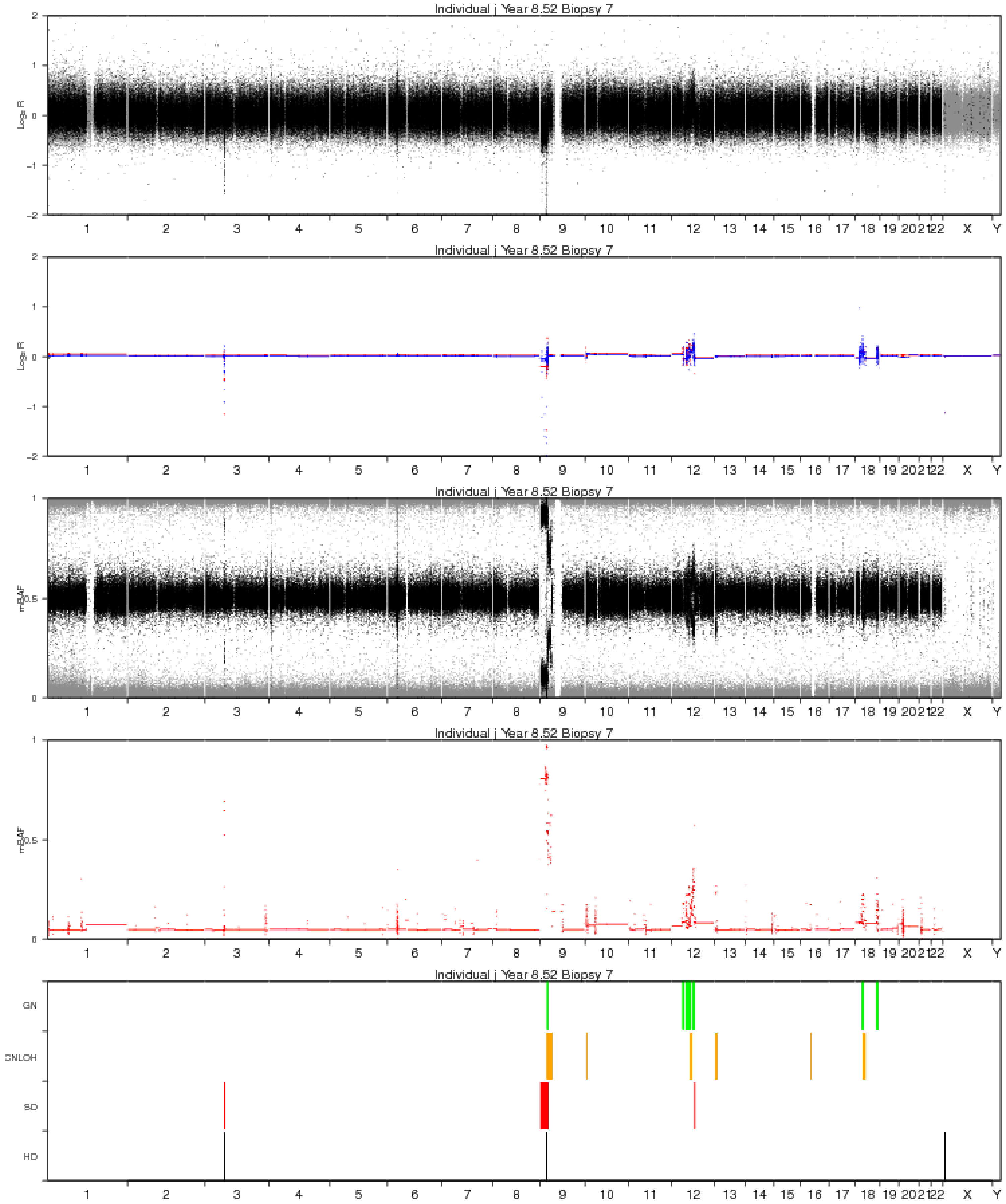
Individual j Year 8.52 Biopsy 7
Log R
2
1
0
−1
−2
1 2 3 4 5 6 7 8 9 10 11 12 13 14 15 16 17 18 19 20 21 22 X Y
Individual j Year 8.52 Biopsy 7
Log R
2
1
0
−1
−2
1 2 3 4 5 6 7 8 9 10 11 12 13 14 15 16 17 18 19 20 21 22 X Y
Individual j Year 8.52 Biopsy 7
mBAF
1
0.5
0
1 2 3 4 5 6 7 8 9 10 11 12 13 14 15 16 17 18 19 20 21 22 X Y
Individual j Year 8.52 Biopsy 7
mBAF
1
0.5
0
1 2 3 4 5 6 7 8 9 10 11 12 13 14 15 16 17 18 19 20 21 22 X Y
Individual j Year 8.52 Biopsy 7
GN
CNLOH
SD
HD
1 2 3 4 5 6 7 8 9 10 11 12 13 14 15 16 17 18 19 20 21 22 X Y

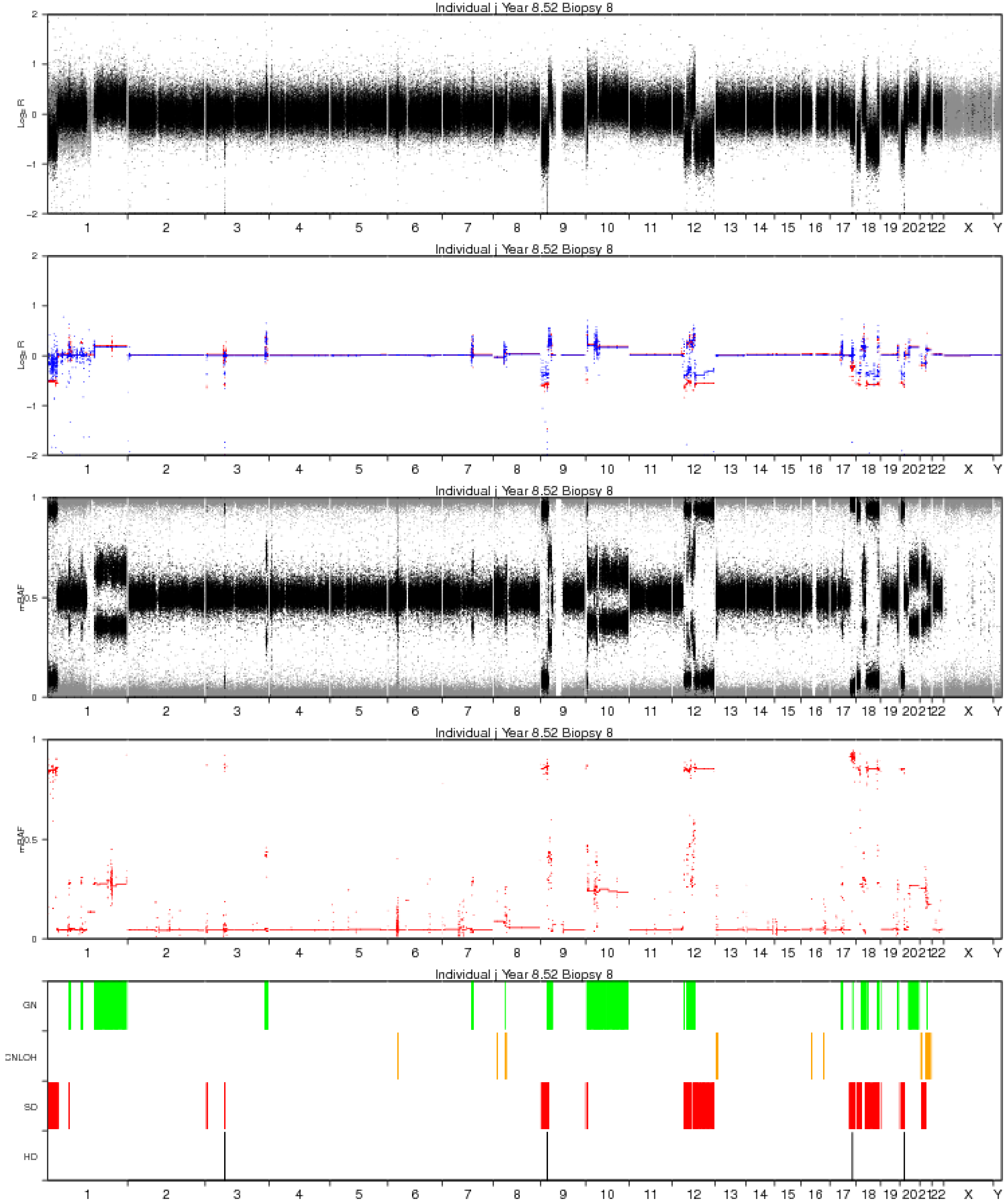

Individual j Year 8.52 Biopsy 8
Log2 R
2 1 0 −1 −2
1 2 3 4 5 6 7 8 9 10 11 12 13 14 15 16 17 18 19 20 21 22 X Y
Individual j Year 8.52 Biopsy 8
Log2 R
2 1 0 −1 −2
1 2 3 4 5 6 7 8 9 10 11 12 13 14 15 16 17 18 19 20 21 22 X Y
Individual j Year 8.52 Biopsy 8
mBAF
1 0.5 0
1 2 3 4 5 6 7 8 9 10 11 12 13 14 15 16 17 18 19 20 21 22 X Y
Individual j Year 8.52 Biopsy 8
mBAF
1 0.5 0
1 2 3 4 5 6 7 8 9 10 11 12 13 14 15 16 17 18 19 20 21 22 X Y
Individual j Year 8.52 Biopsy 8
GN
CNLOH
SD
HD
1 2 3 4 5 6 7 8 9 10 11 12 13 14 15 16 17 18 19 20 21 22 X Y

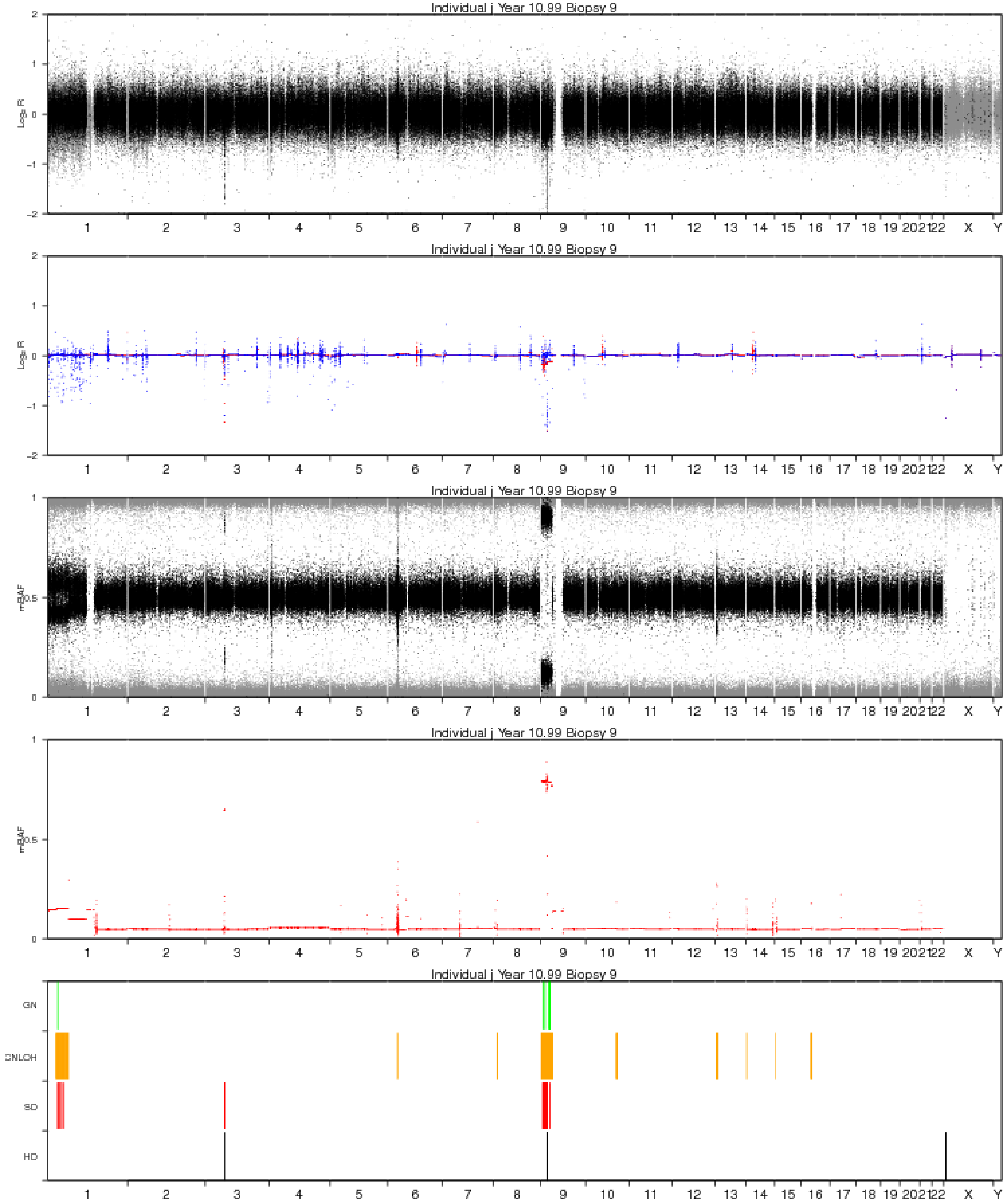
Individual j Year 10.99 Biopsy 9
Log R
2
1
0
−1
−2
1 2 3 4 5 6 7 8 9 10 11 12 13 14 15 16 17 18 19 20 21 22 X Y
Individual j Year 10.99 Biopsy 9
Log R
2
1
0
−1
−2
1 2 3 4 5 6 7 8 9 10 11 12 13 14 15 16 17 18 19 20 21 22 X Y
Individual j Year 10.99 Biopsy 9
mBAF
1
0.5
0
1 2 3 4 5 6 7 8 9 10 11 12 13 14 15 16 17 18 19 20 21 22 X Y
Individual j Year 10.99 Biopsy 9
mBAF
1
0.5
0
1 2 3 4 5 6 7 8 9 10 11 12 13 14 15 16 17 18 19 20 21 22 X Y
Individual j Year 10.99 Biopsy 9
GN
CNLOH
SD
HD
1 2 3 4 5 6 7 8 9 10 11 12 13 14 15 16 17 18 19 20 21 22 X Y

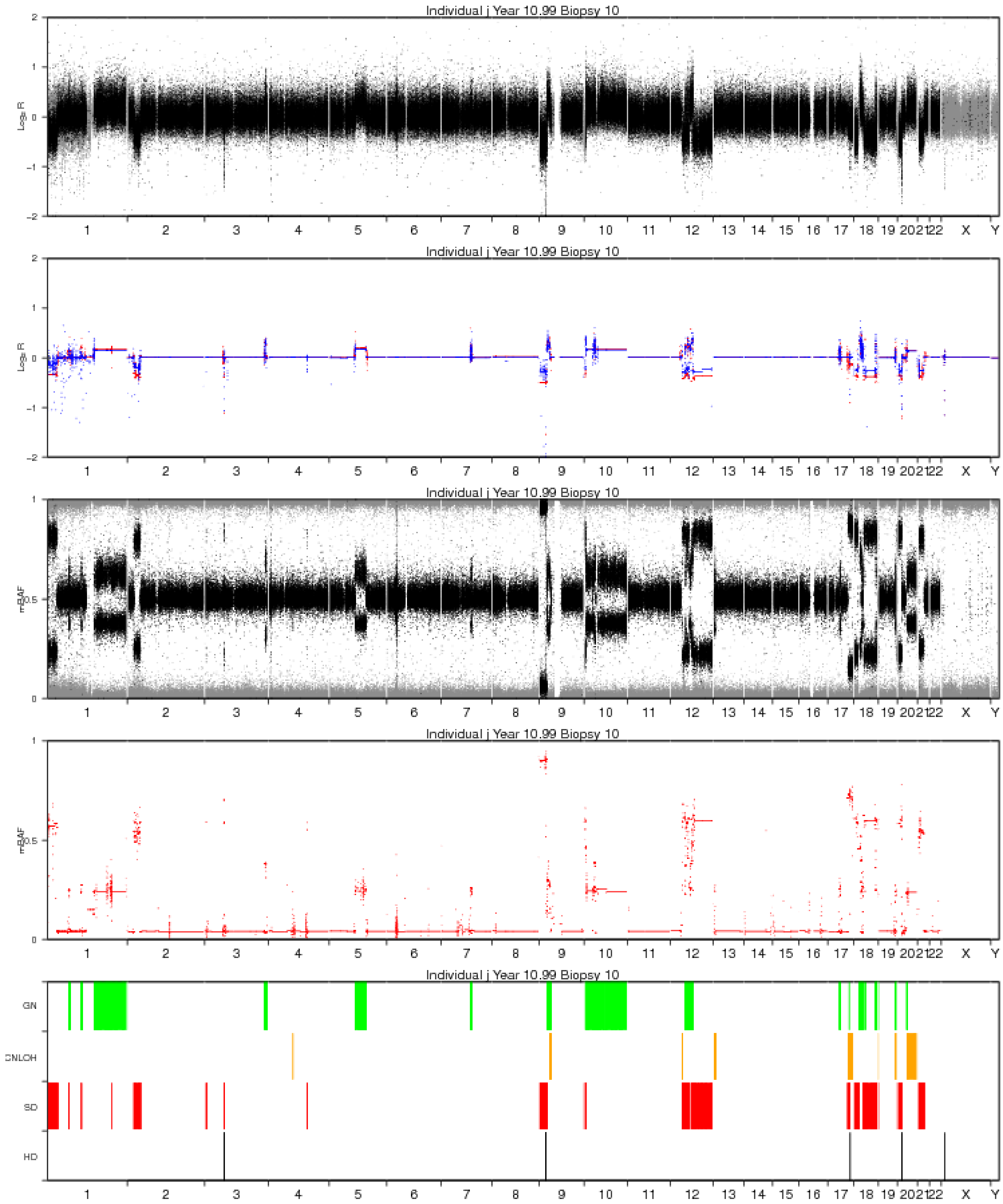

Individual j Year 10.99 Biopsy 10
Log₂ R
mBAF
GN
CNLOH
SD
HD
1 2 3 4 5 6 7 8 9 10 11 12 13 14 15 16 17 18 19 20 21 22 X Y

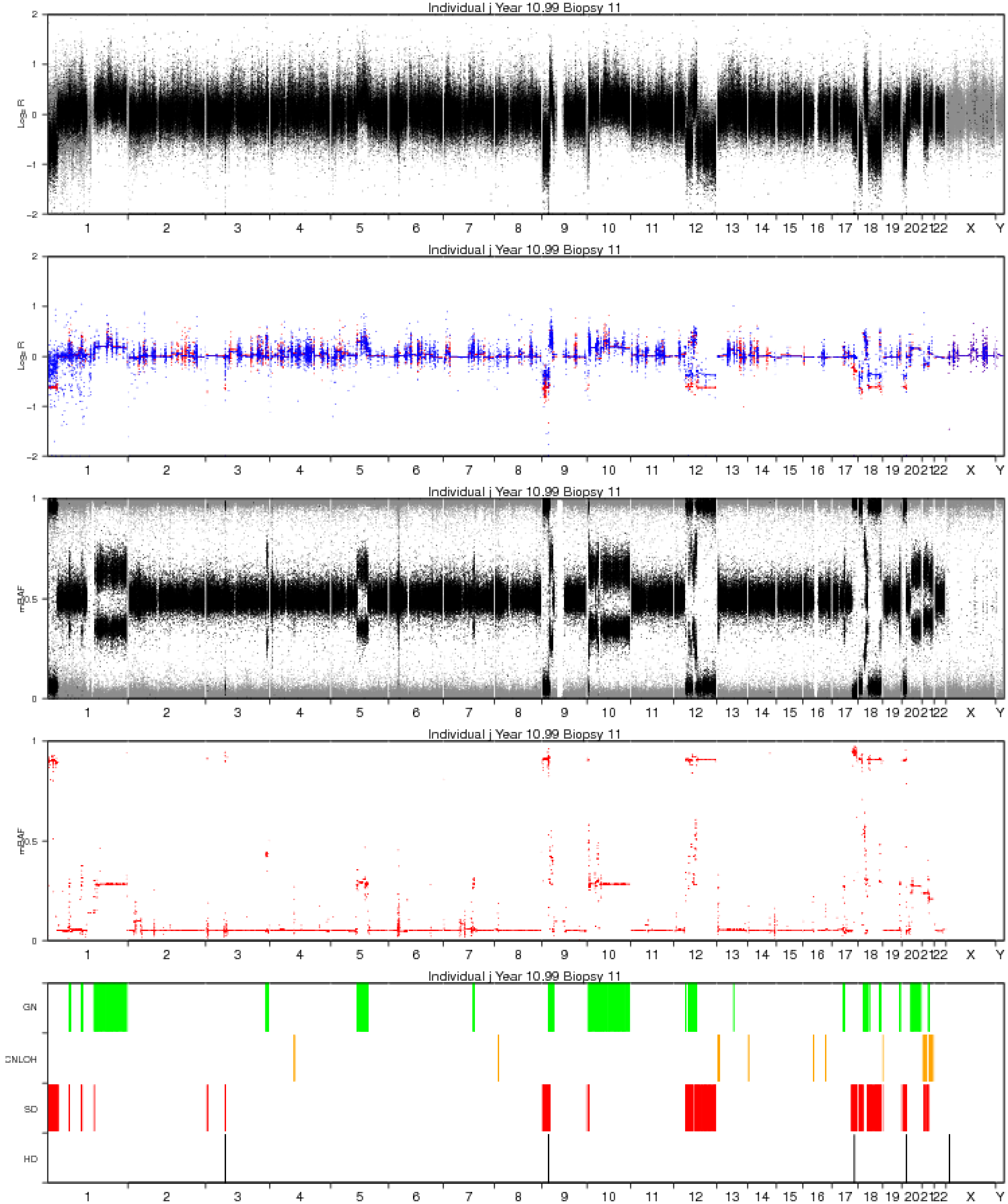
Individual j Year 10.99 Biopsy 11
Log R
mBAF
GN
CNLOH
SD
HD
1 2 3 4 5 6 7 8 9 10 11 12 13 14 15 16 17 18 19 20 21 22 X Y

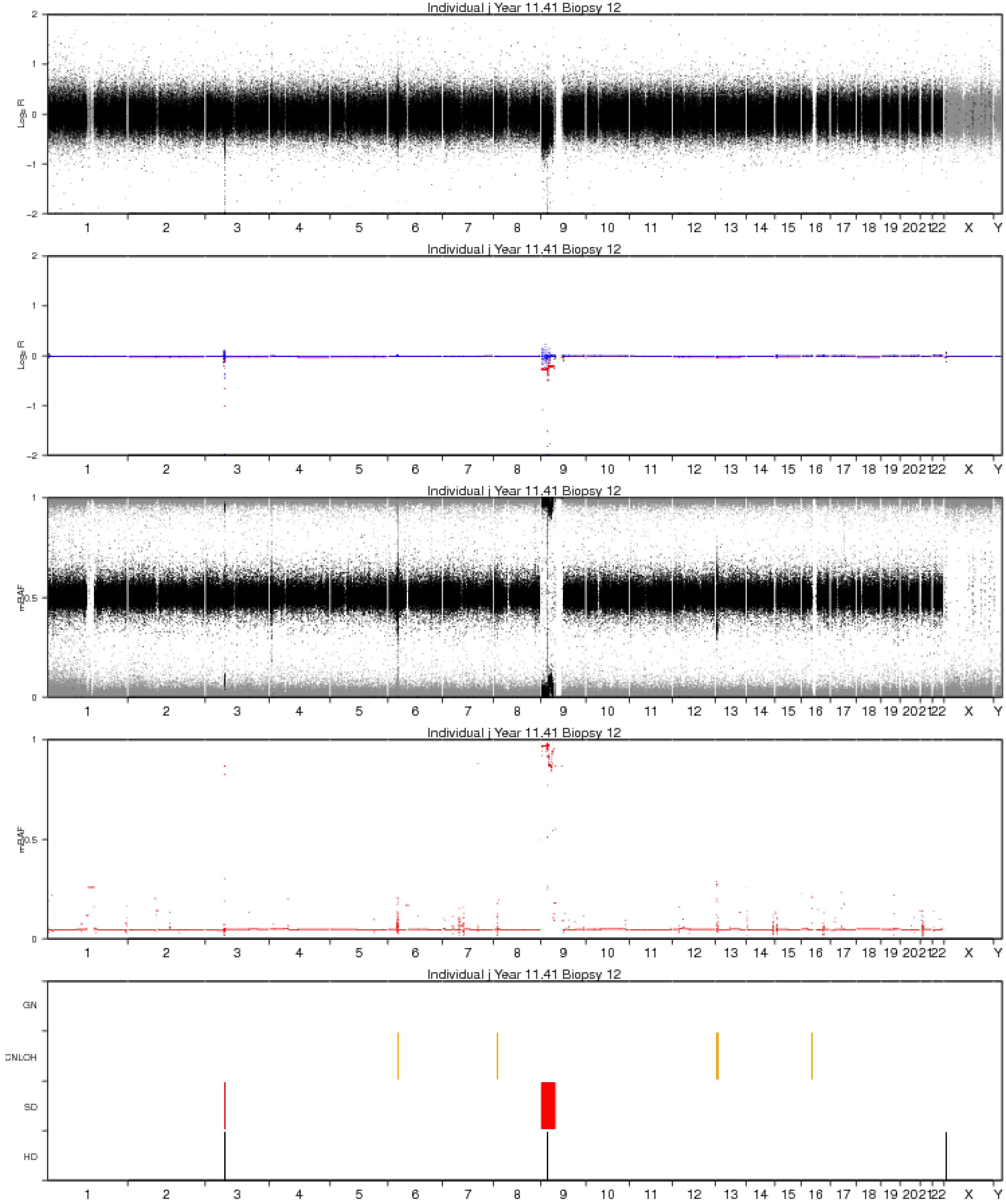
Individual j Year 11.41 Biopsy 12
Log R
mBAF
GN
CNLOH
SD
HD
1 2 3 4 5 6 7 8 9 10 11 12 13 14 15 16 17 18 19 20 21 22 X Y

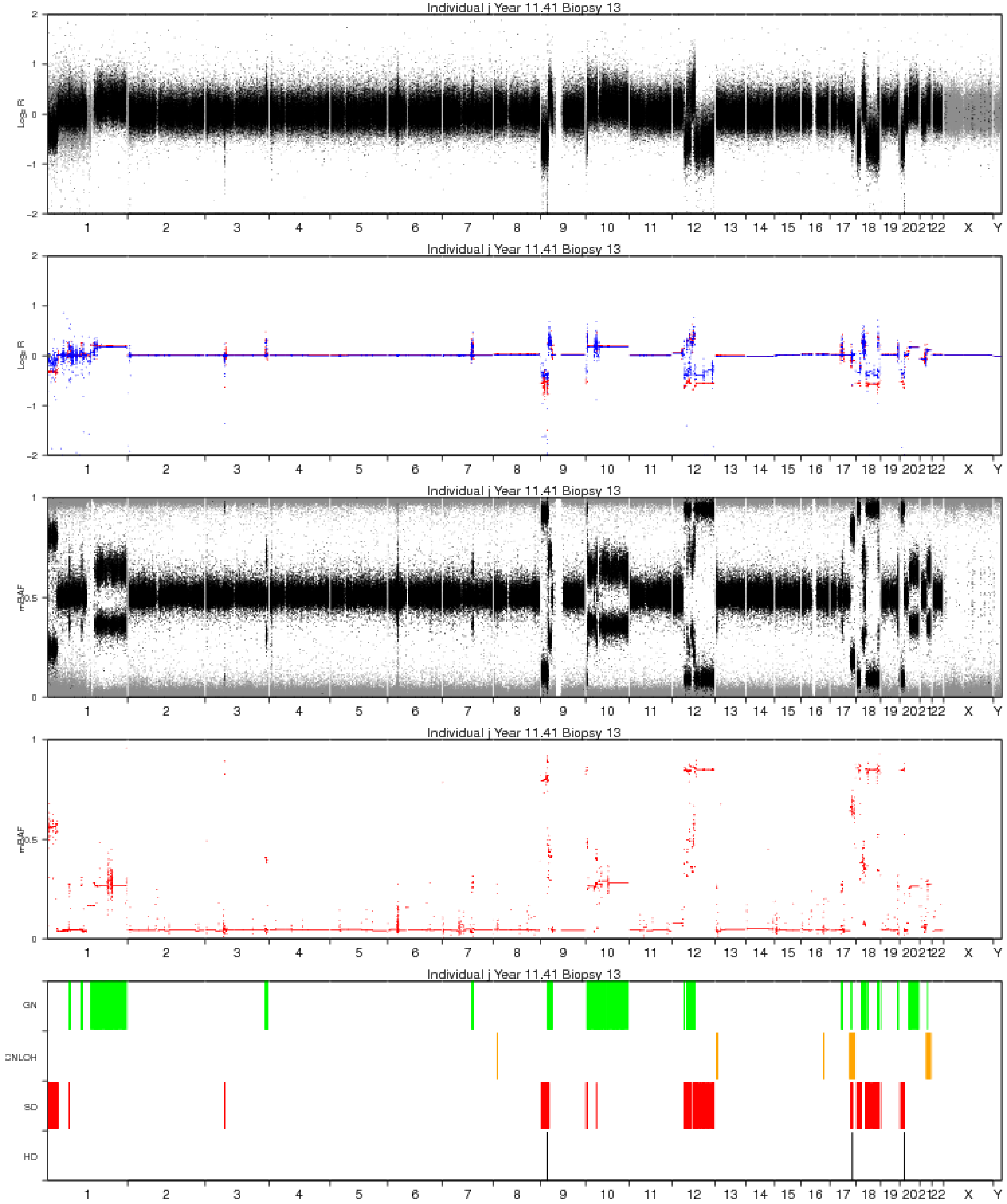
Individual j Year 11.41 Biopsy 13
Log R
mBAF
GN
CNLOH
SD
HD
1
2
3
4
5
6
7
8
9
10
11
12
13
14
15
16
17
18
19
20
21
22
X
Y

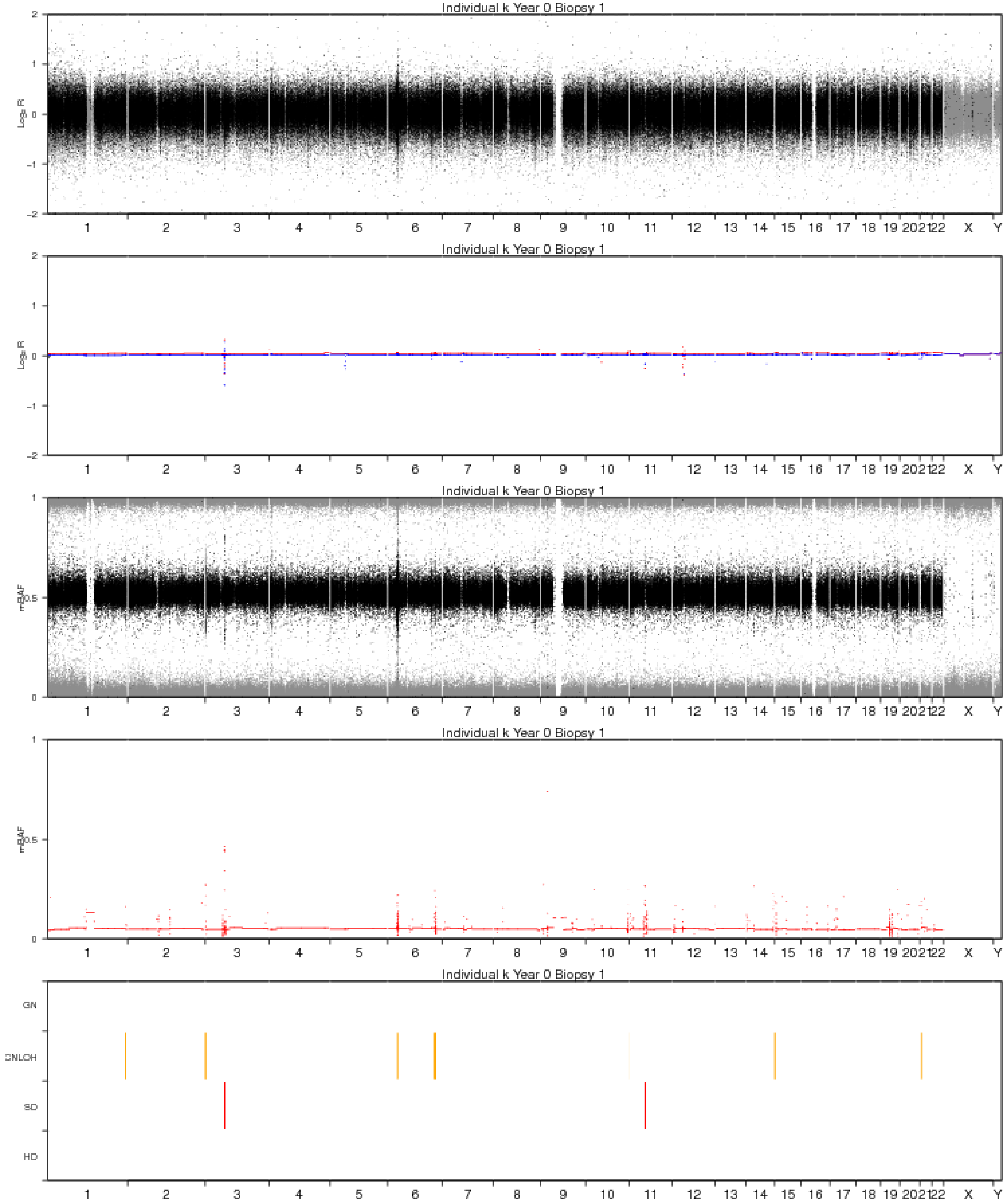
Individual k Year 0 Biopsy 1
Log2 R
mBAF
GN
CNLOH
SD
HD
1 2 3 4 5 6 7 8 9 10 11 12 13 14 15 16 17 18 19 20 21 22 X Y

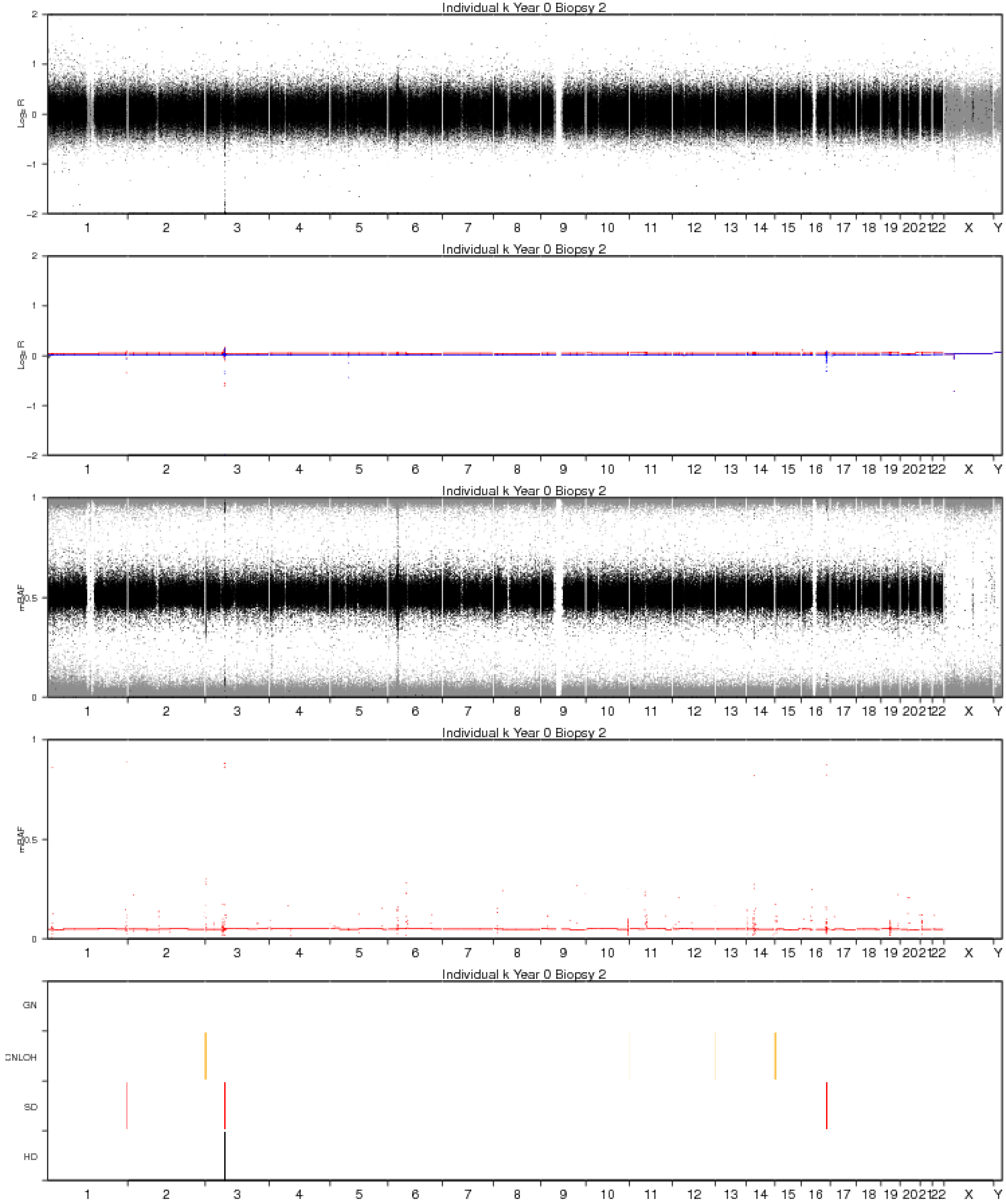
Individual k Year 0 Biopsy 2
Log R
mBAF
GN
CNLOH
SD
HD
1
2
3
4
5
6
7
8
9
10
11
12
13
14
15
16
17
18
19
20
21
22
X
Y

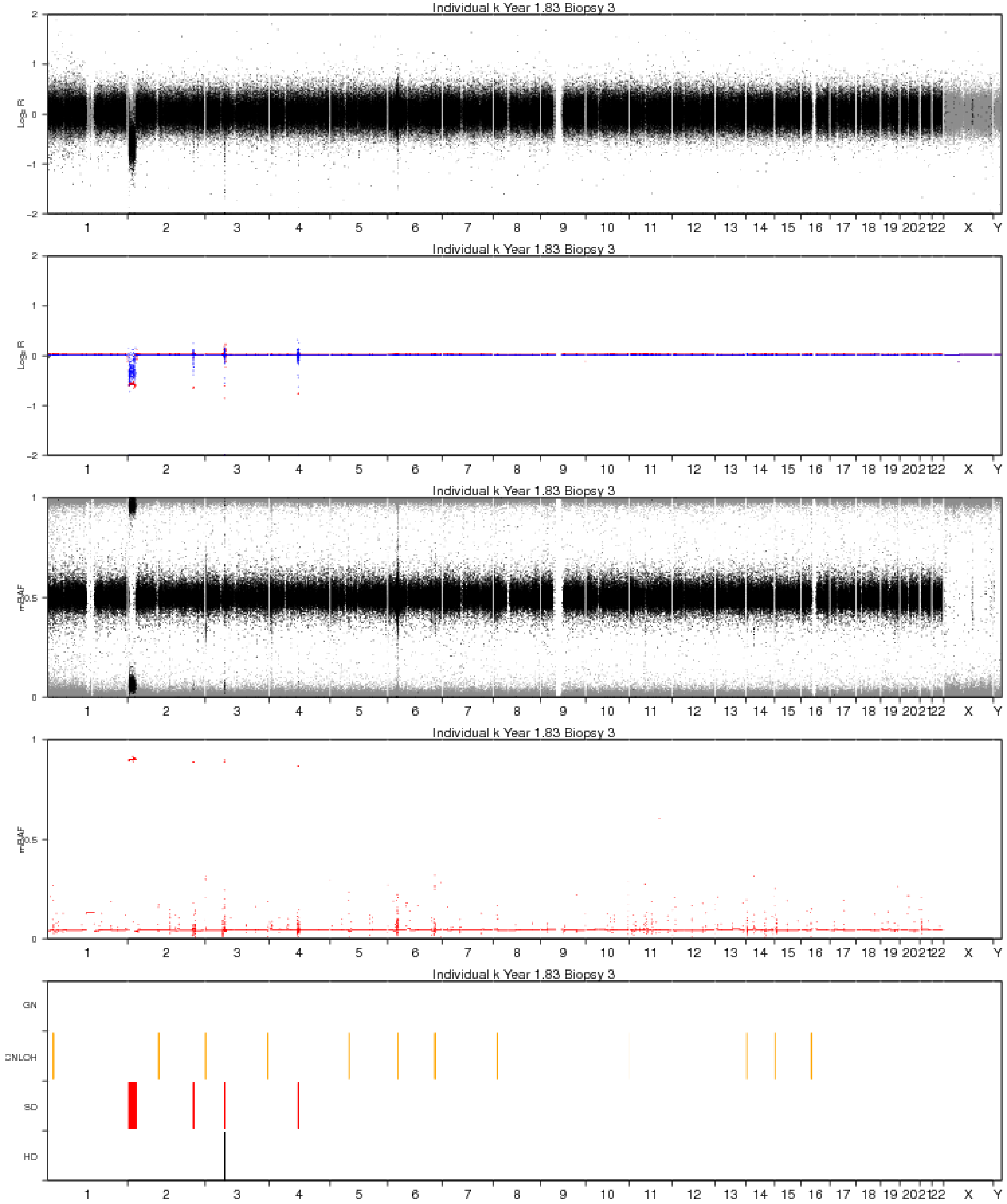
Individual k Year 1.83 Biopsy 3
Log R
mBAF
GN
CNLOH
SD
HD
1 2 3 4 5 6 7 8 9 10 11 12 13 14 15 16 17 18 19 20 21 22 X Y

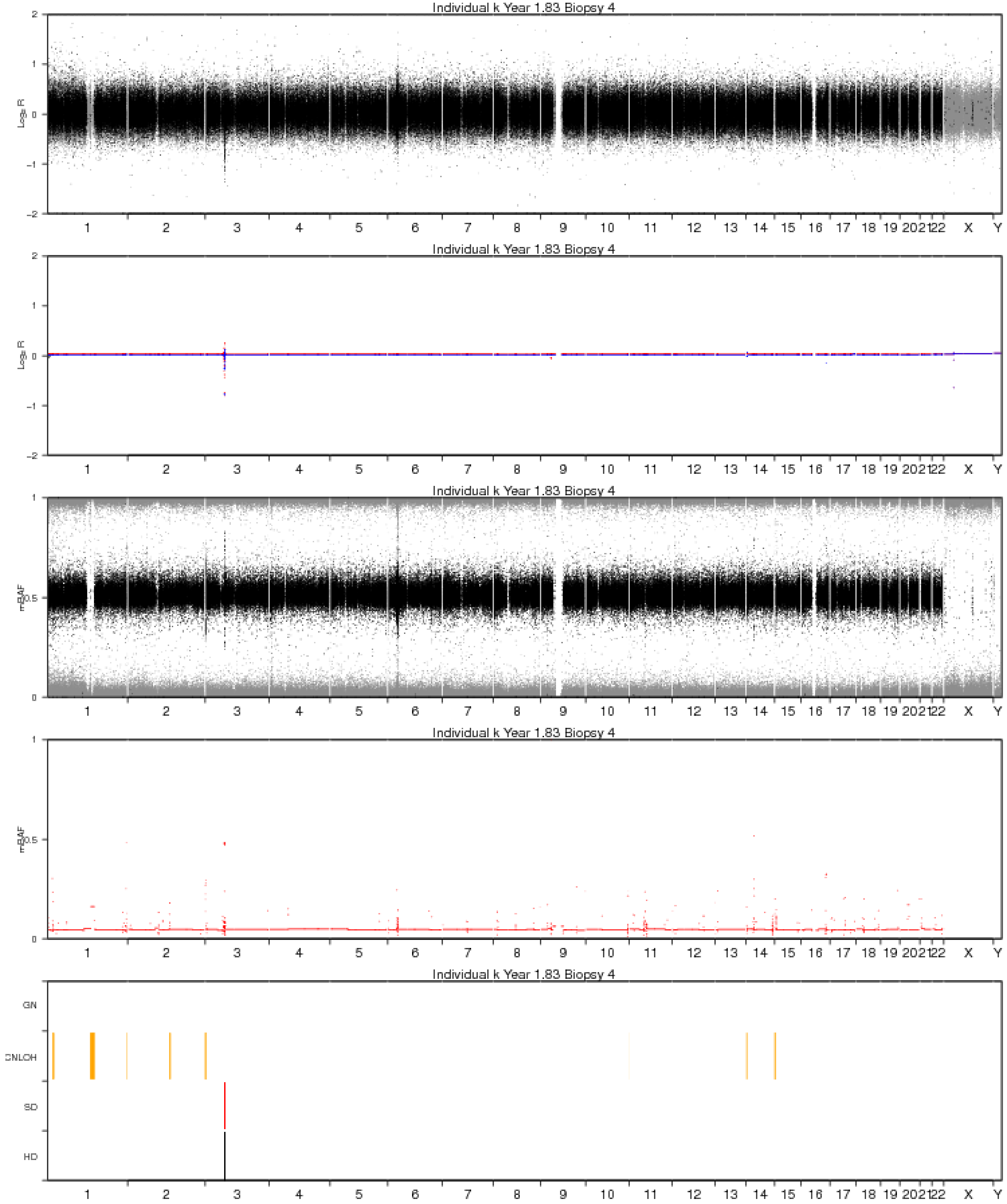
Individual k Year 1.83 Biopsy 4
Log R
mBAF
GN
CNLOH
SD
HD
1 2 3 4 5 6 7 8 9 10 11 12 13 14 15 16 17 18 19 20 21 22 X Y

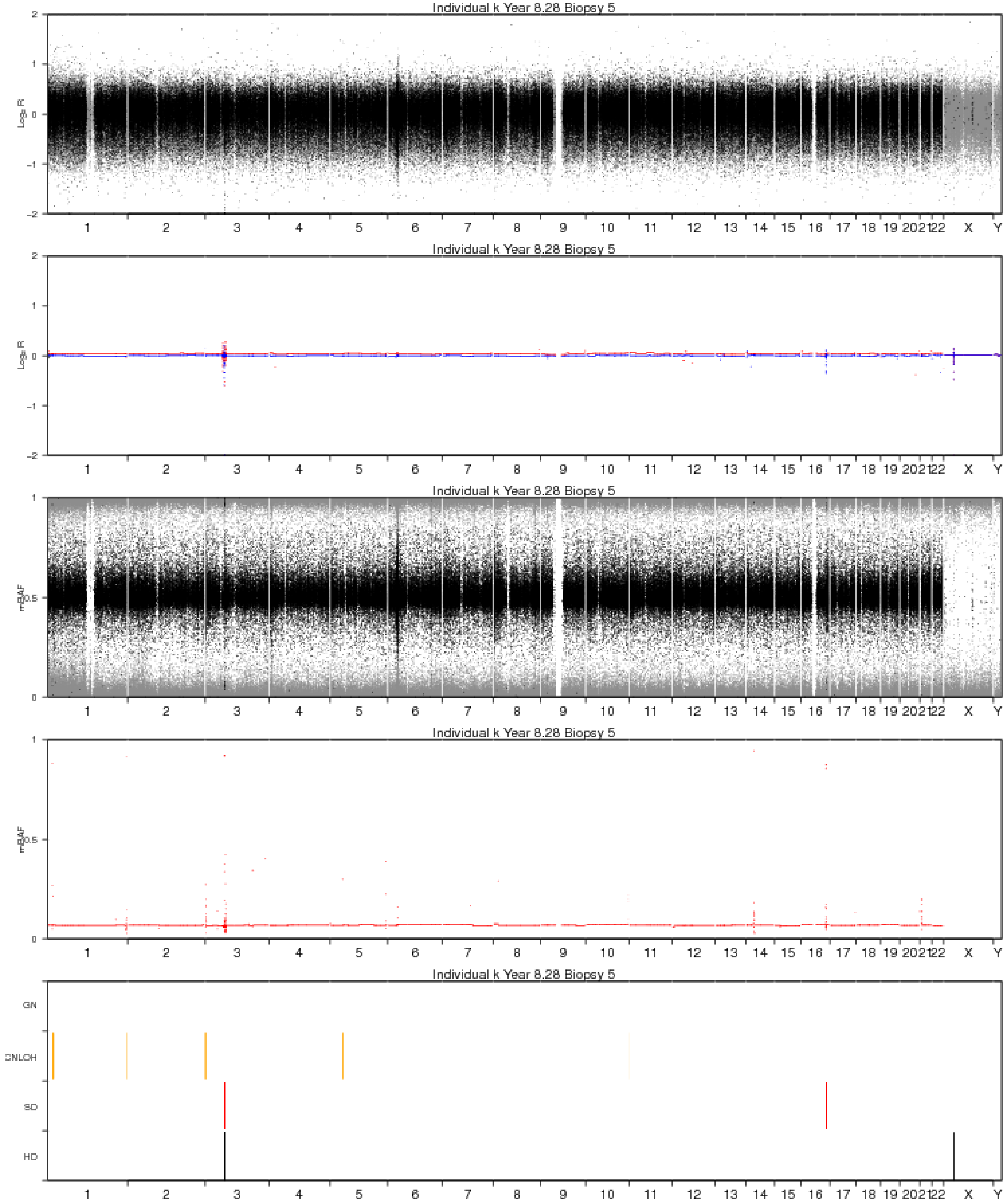
Individual k Year 8.28 Biopsy 5
Log R
mBAF
GN
CNLOH
SD
HD
1 2 3 4 5 6 7 8 9 10 11 12 13 14 15 16 17 18 19 20 21 22 X Y

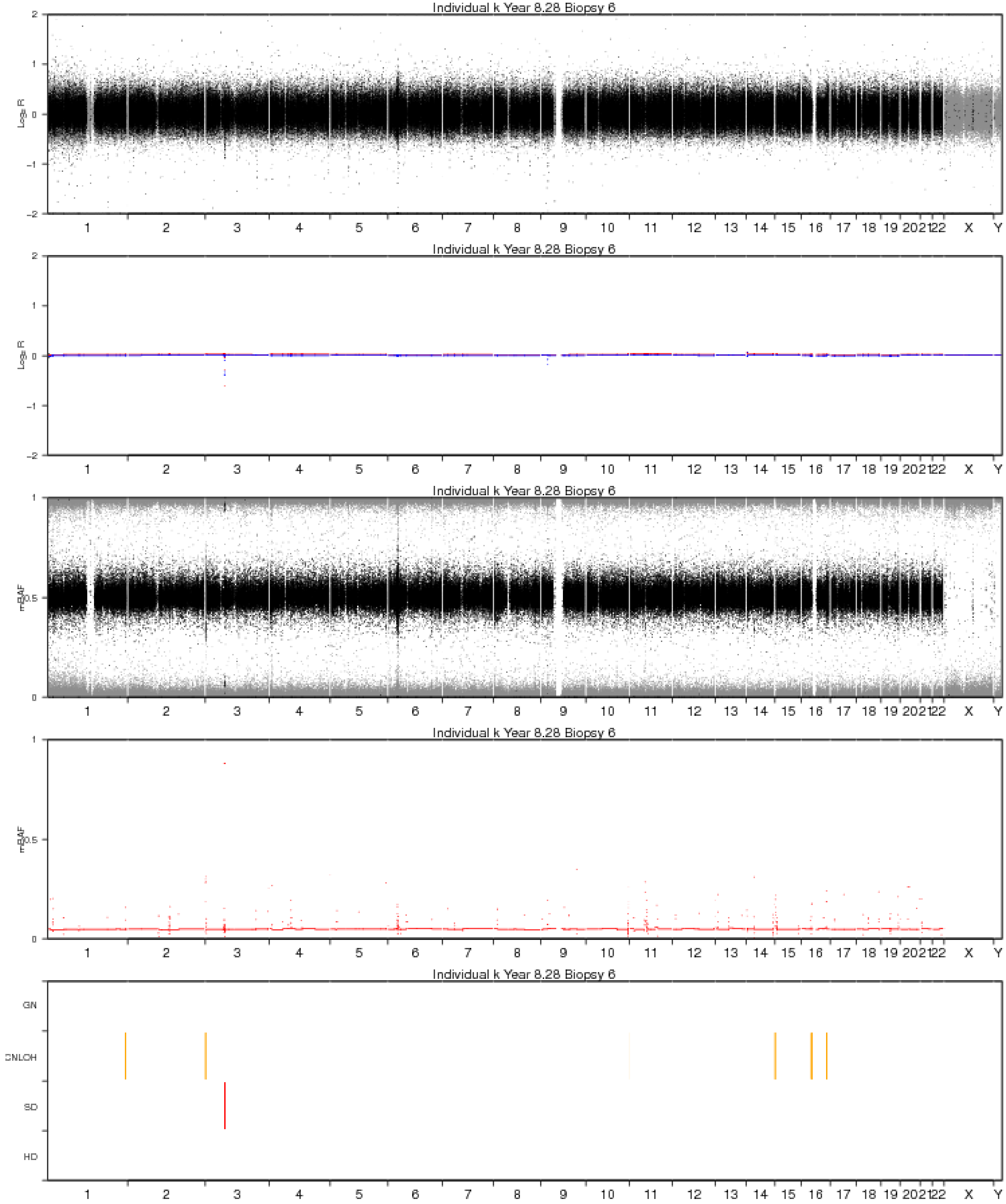

Individual k Year 8.28 Biopsy 6
Log R
mBAF
GN
CNLOH
SD
HD
1 2 3 4 5 6 7 8 9 10 11 12 13 14 15 16 17 18 19 20 21 22 X Y

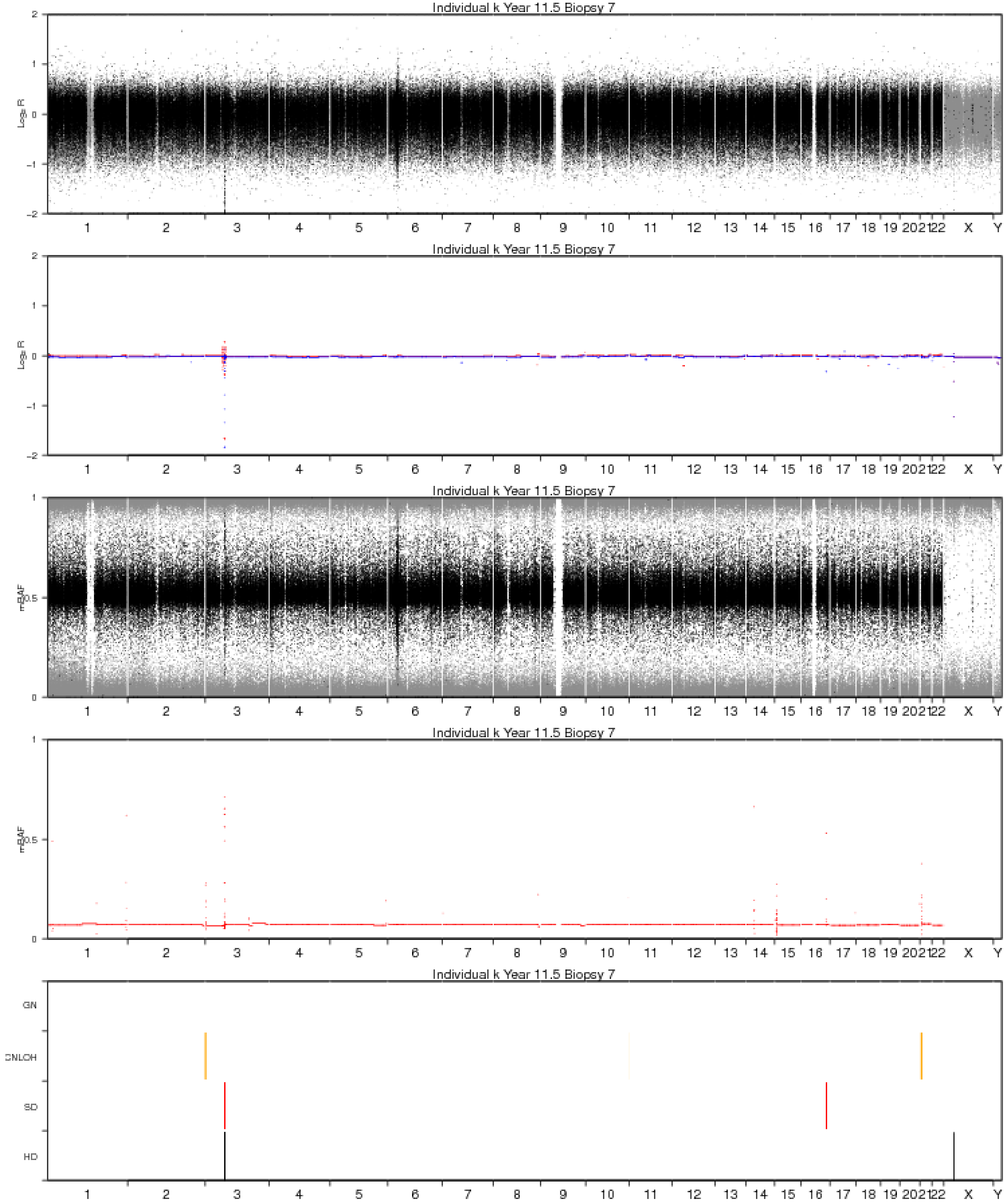
Individual k Year 11.5 Biopsy 7
Log₂ R
mBAF
GN
CNLOH
SD
HD
1 2 3 4 5 6 7 8 9 10 11 12 13 14 15 16 17 18 19 20 21 22 X Y

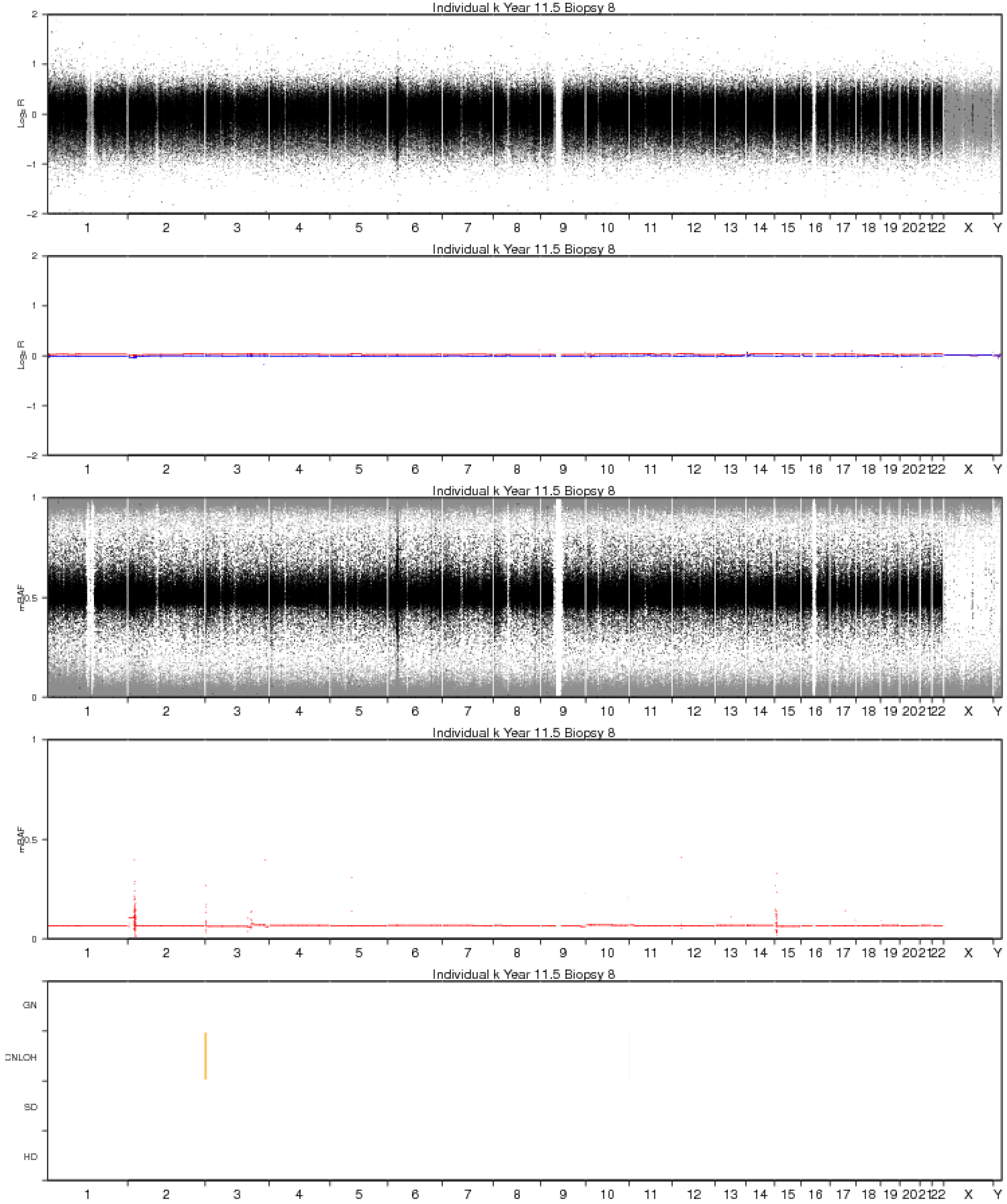

Individual k Year 11.5 Biopsy 8
Log R
2
1
0
−1
−2
1 2 3 4 5 6 7 8 9 10 11 12 13 14 15 16 17 18 19 20 21 22 X Y
Individual k Year 11.5 Biopsy 8
Log R
2
1
0
−1
−2
1 2 3 4 5 6 7 8 9 10 11 12 13 14 15 16 17 18 19 20 21 22 X Y
Individual k Year 11.5 Biopsy 8
mBAF
1
0.5
0
1 2 3 4 5 6 7 8 9 10 11 12 13 14 15 16 17 18 19 20 21 22 X Y
Individual k Year 11.5 Biopsy 8
mBAF
1
0.5
0
1 2 3 4 5 6 7 8 9 10 11 12 13 14 15 16 17 18 19 20 21 22 X Y
Individual k Year 11.5 Biopsy 8
GN
CNLOH
SD
HD
1 2 3 4 5 6 7 8 9 10 11 12 13 14 15 16 17 18 19 20 21 22 X Y

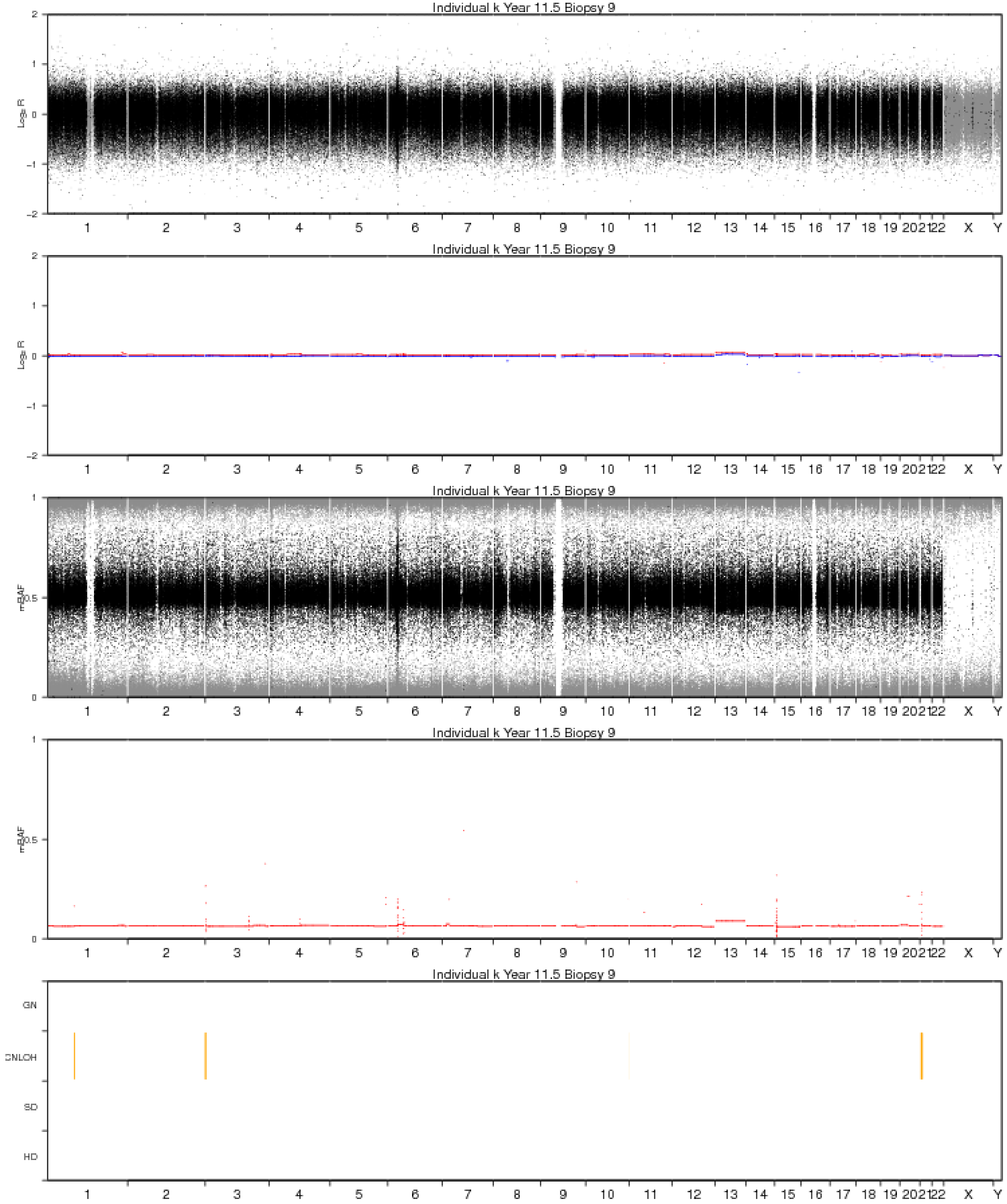
Individual k Year 11.5 Biopsy 9
Log R
mBAF
GN
CNLOH
SD
HD
1 2 3 4 5 6 7 8 9 10 11 12 13 14 15 16 17 18 19 20 21 22 X Y

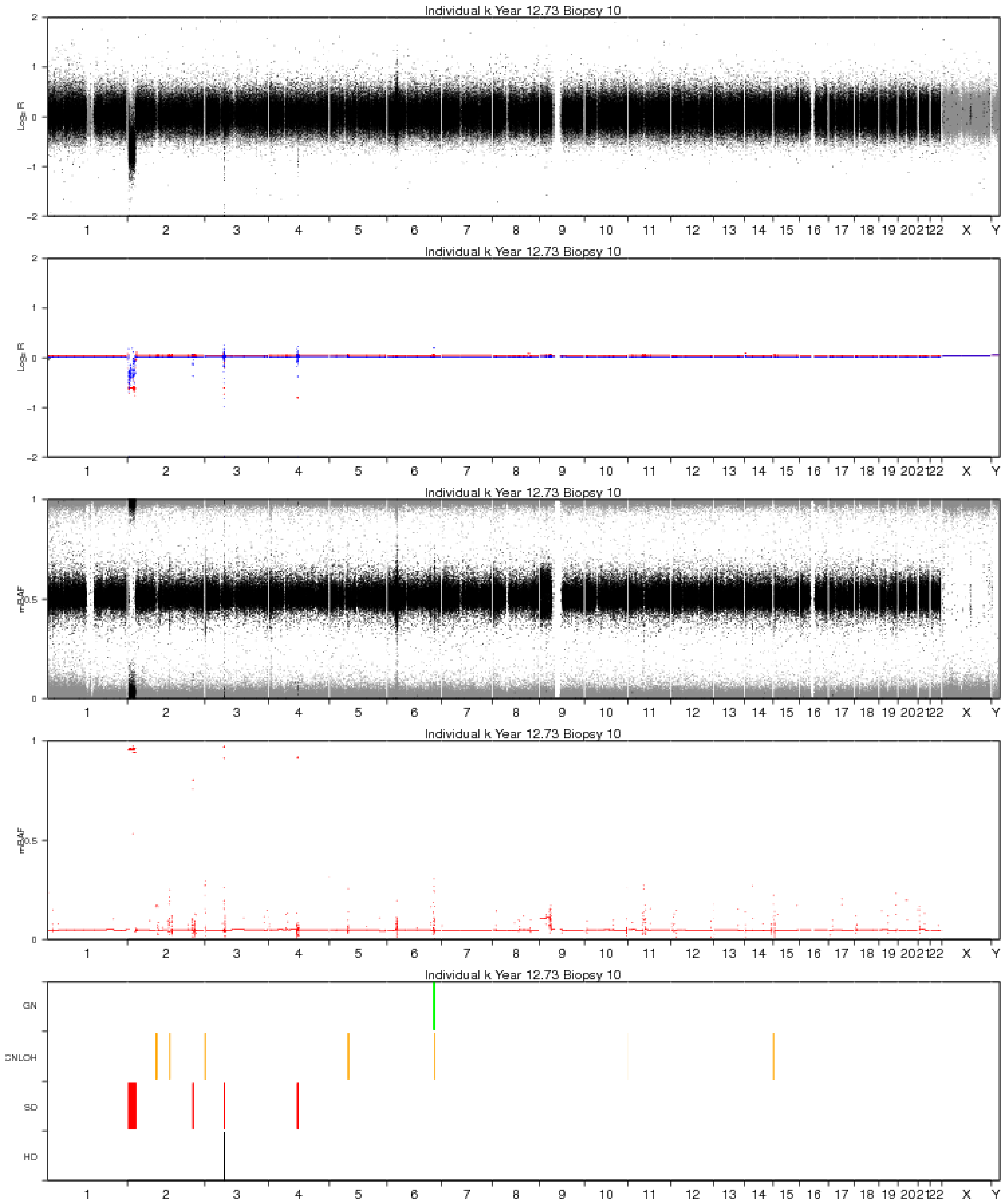
Individual k Year 12.73 Biopsy 10
Log R
2
1
0
−1
−2
1 2 3 4 5 6 7 8 9 10 11 12 13 14 15 16 17 18 19 20 21 22 X Y
Individual k Year 12.73 Biopsy 10
Log R
Individual k Year 12.73 Biopsy 10
mBAF
1
0.5
0
Individual k Year 12.73 Biopsy 10
mBAF
Individual k Year 12.73 Biopsy 10
GN
CNLOH
SD
HD

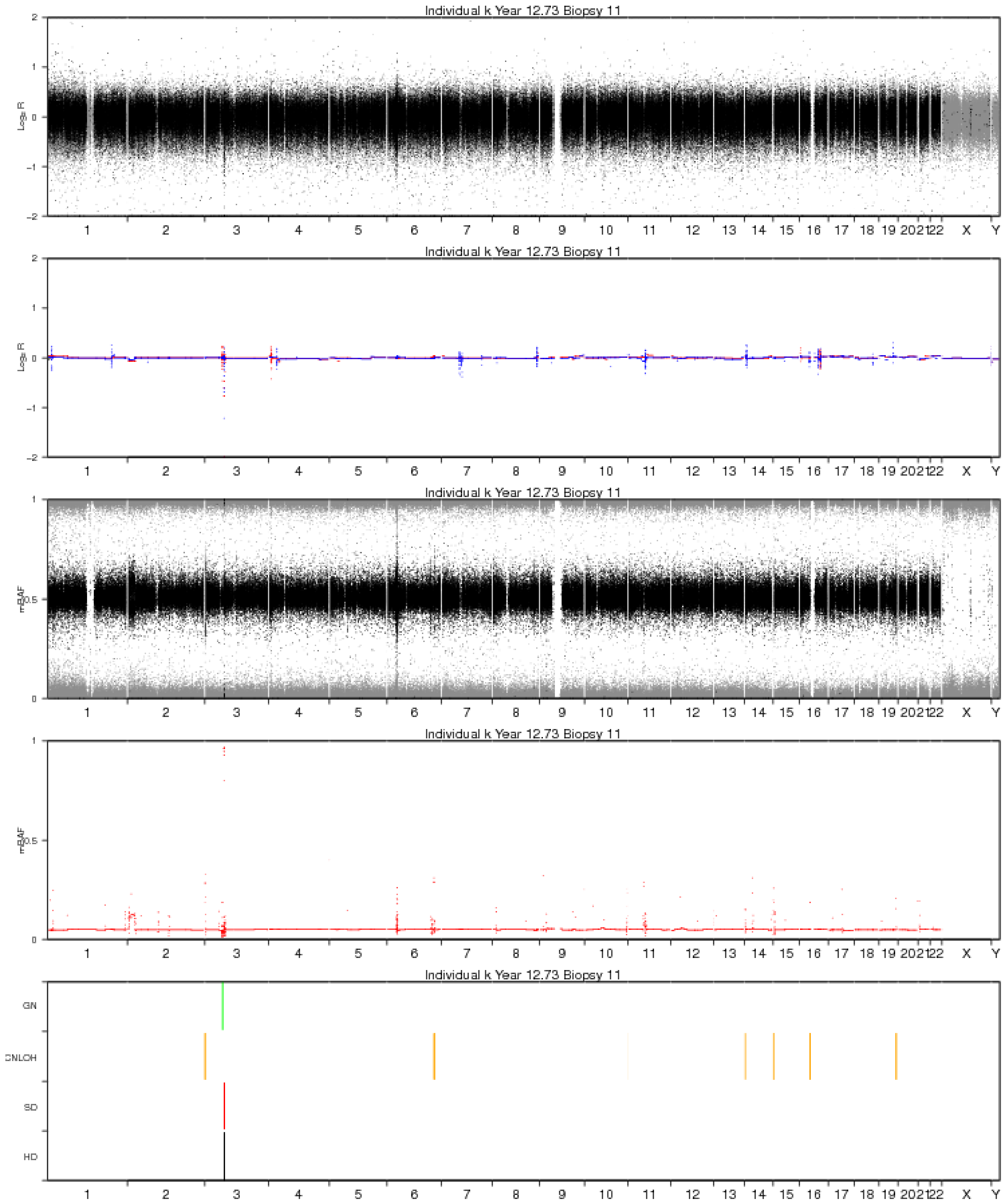
Individual k Year 12.73 Biopsy 11
Log R
2
1
0
−1
−2
1 2 3 4 5 6 7 8 9 10 11 12 13 14 15 16 17 18 19 20 21 22 X Y
Individual k Year 12.73 Biopsy 11
Log R
Individual k Year 12.73 Biopsy 11
mBAF
1
0.5
0
Individual k Year 12.73 Biopsy 11
mBAF
Individual k Year 12.73 Biopsy 11
GN
CNLOH
SD
HD

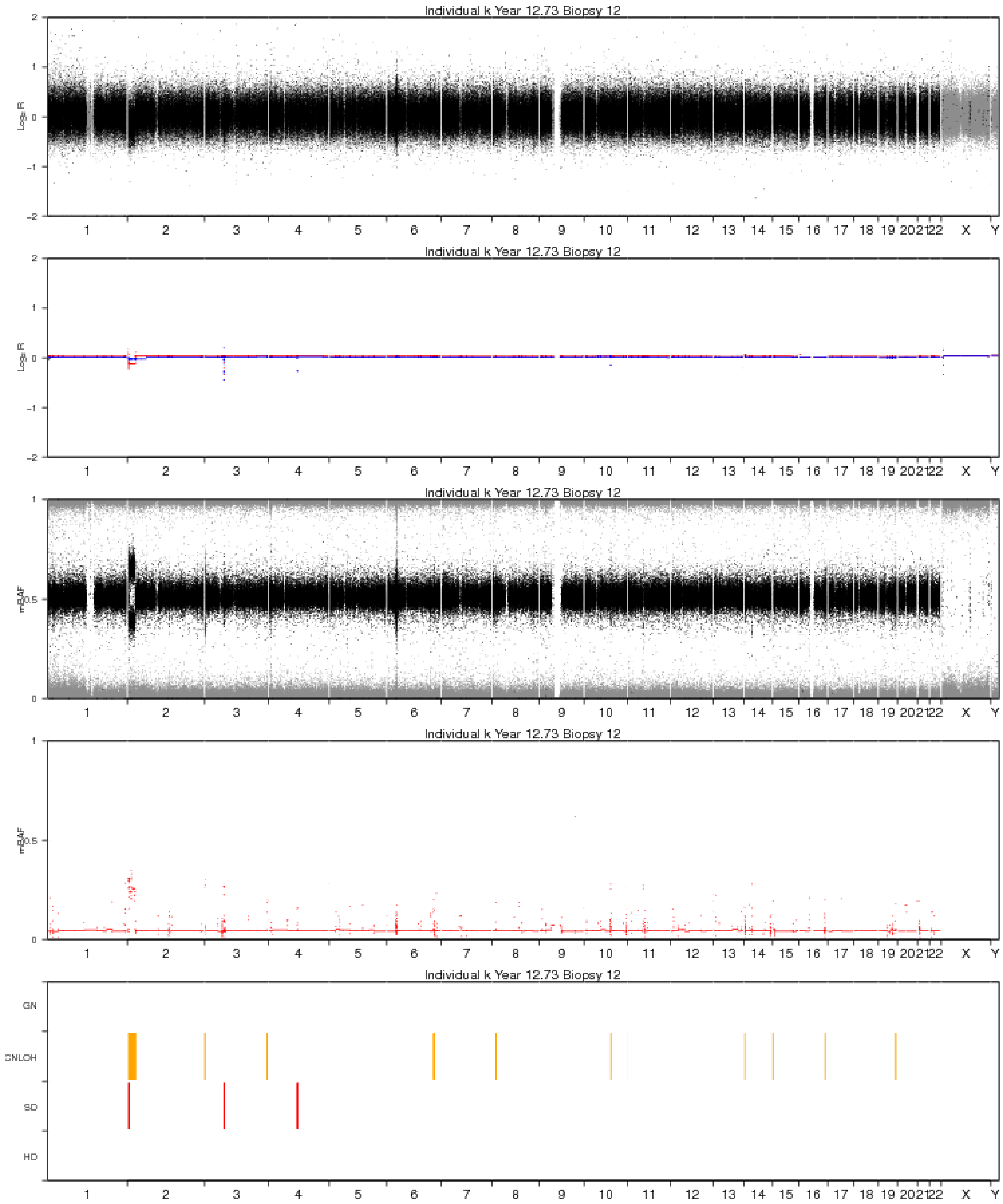
Individual k Year 12.73 Biopsy 12
Log R
mBAF
GN
CNLOH
SD
HD
1
2
3
4
5
6
7
8
9
10
11
12
13
14
15
16
17
18
19
20
21
22
X
Y

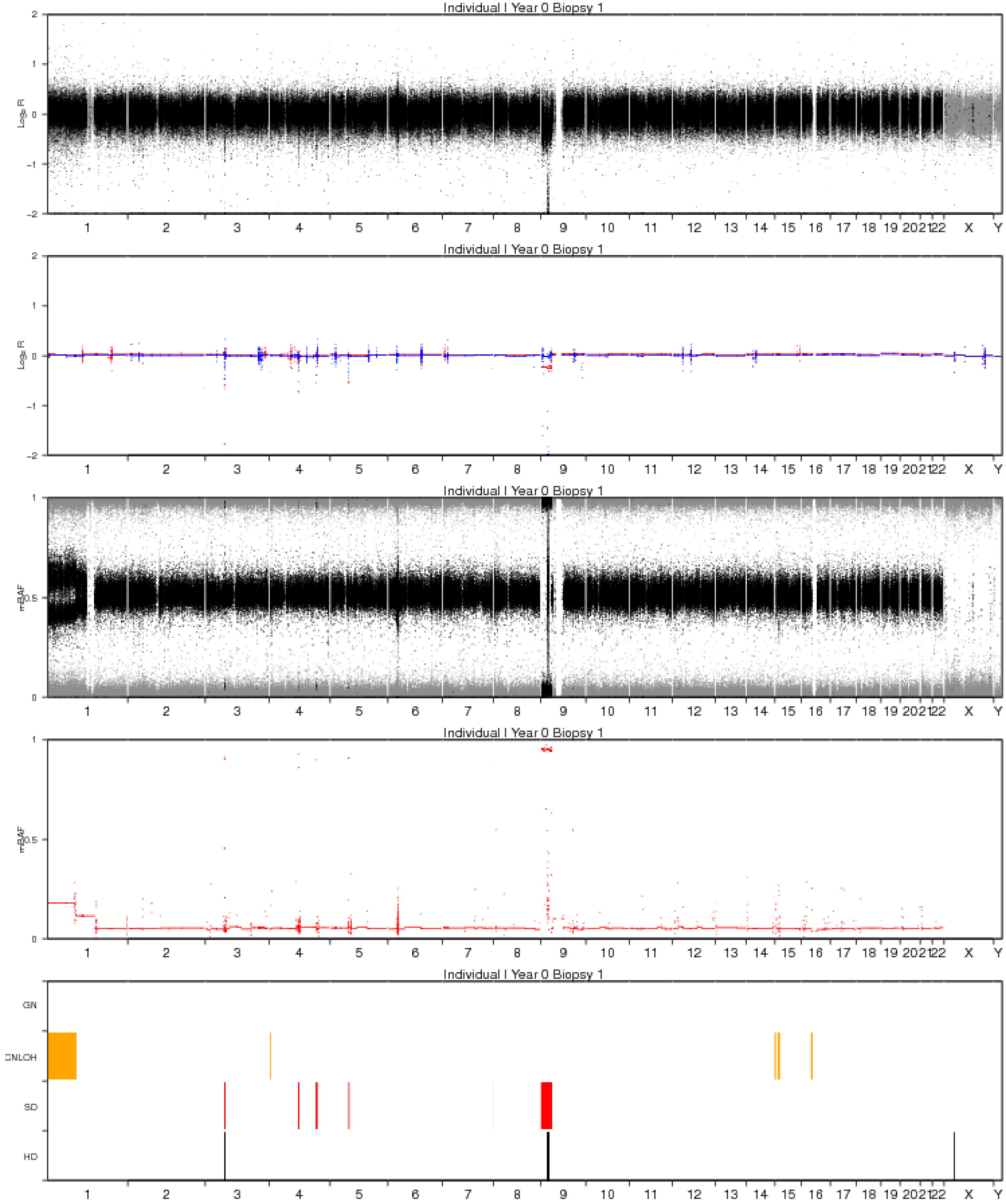
Individual I Year 0 Biopsy 1
Log R
mBAF
GN
CNLOH
SD
HD
1 2 3 4 5 6 7 8 9 10 11 12 13 14 15 16 17 18 19 20 21 22 X Y

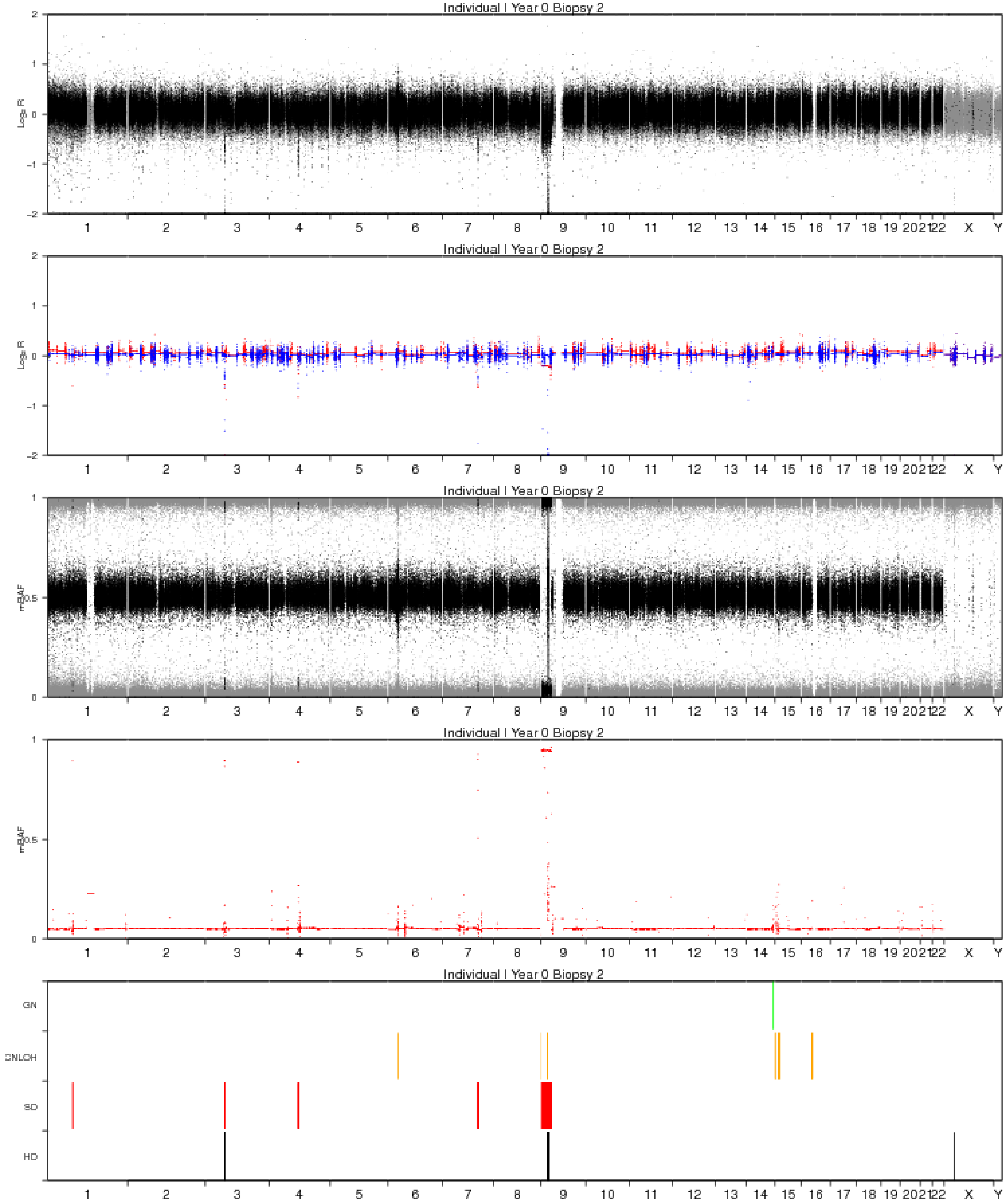
Individual I Year 0 Biopsy 2
Log2 R
mBAF
GN
CNLOH
SD
HD
1 2 3 4 5 6 7 8 9 10 11 12 13 14 15 16 17 18 19 20 21 22 X Y

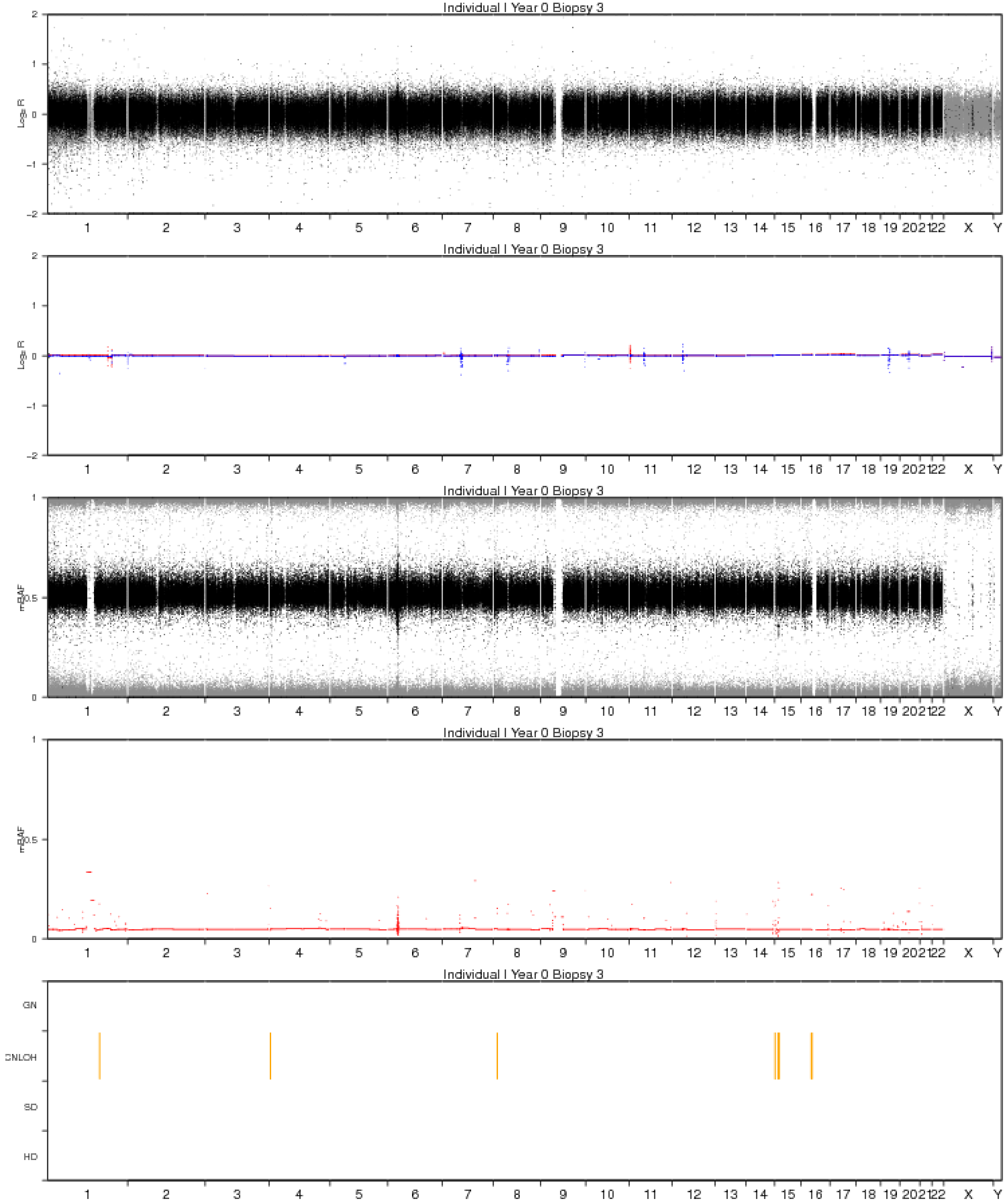
Individual I Year 0 Biopsy 3
Log R
2
1
0
−1
−2
mBAF
1
0.5
0
GN
CNLOH
SD
HD
1
2
3
4
5
6
7
8
9
10
11
12
13
14
15
16
17
18
19
20
21
22
X
Y

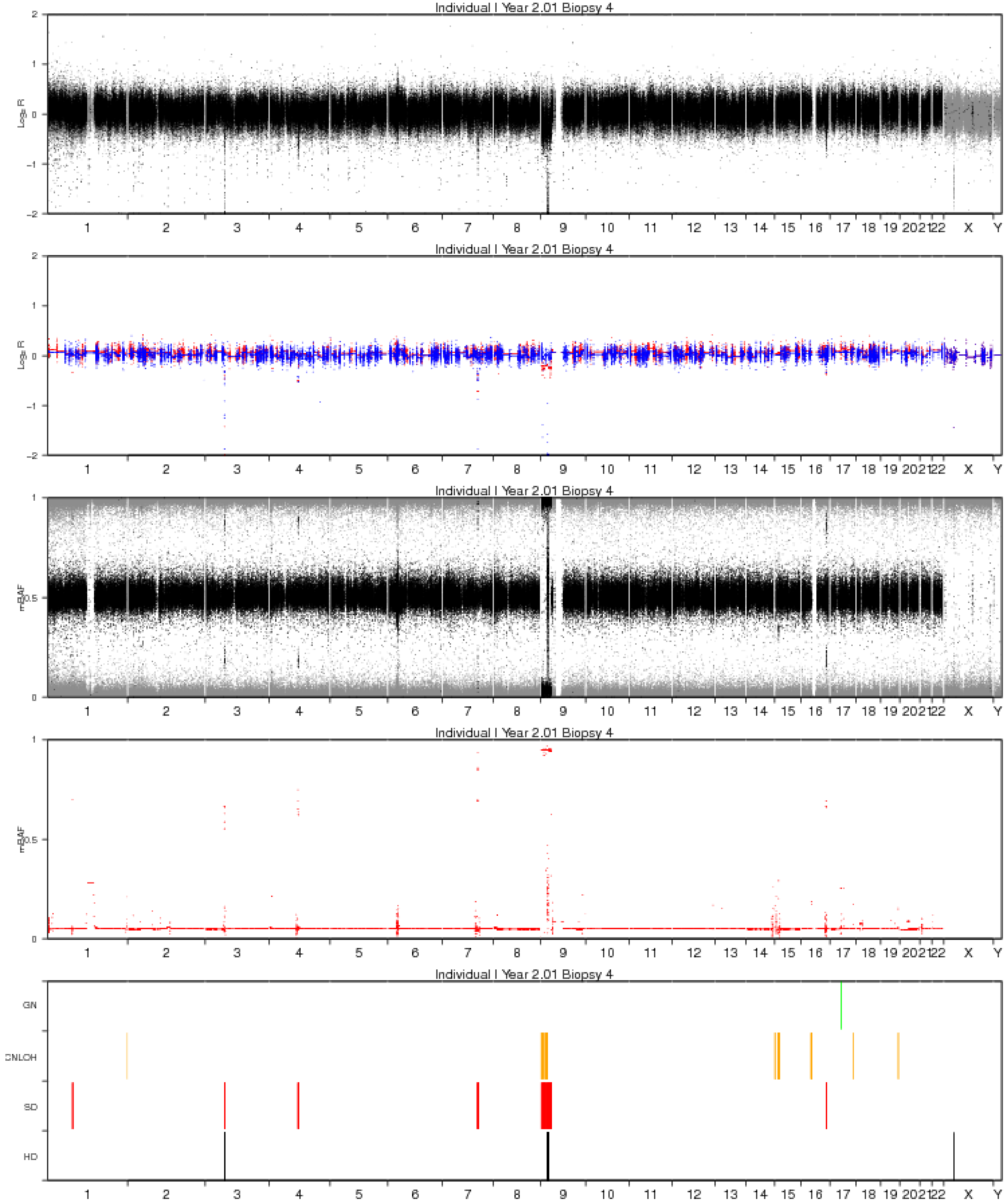
Individual I Year 2.01 Biopsy 4
Log2 R
2 1 0 −1 −2
1 2 3 4 5 6 7 8 9 10 11 12 13 14 15 16 17 18 19 20 21 22 X Y
Individual I Year 2.01 Biopsy 4
Log2 R
2 1 0 −1 −2
1 2 3 4 5 6 7 8 9 10 11 12 13 14 15 16 17 18 19 20 21 22 X Y
Individual I Year 2.01 Biopsy 4
mBAF
1 0.5 0
1 2 3 4 5 6 7 8 9 10 11 12 13 14 15 16 17 18 19 20 21 22 X Y
Individual I Year 2.01 Biopsy 4
mBAF
1 0.5 0
1 2 3 4 5 6 7 8 9 10 11 12 13 14 15 16 17 18 19 20 21 22 X Y
Individual I Year 2.01 Biopsy 4
GN
CNLOH
SD
HD
1 2 3 4 5 6 7 8 9 10 11 12 13 14 15 16 17 18 19 20 21 22 X Y

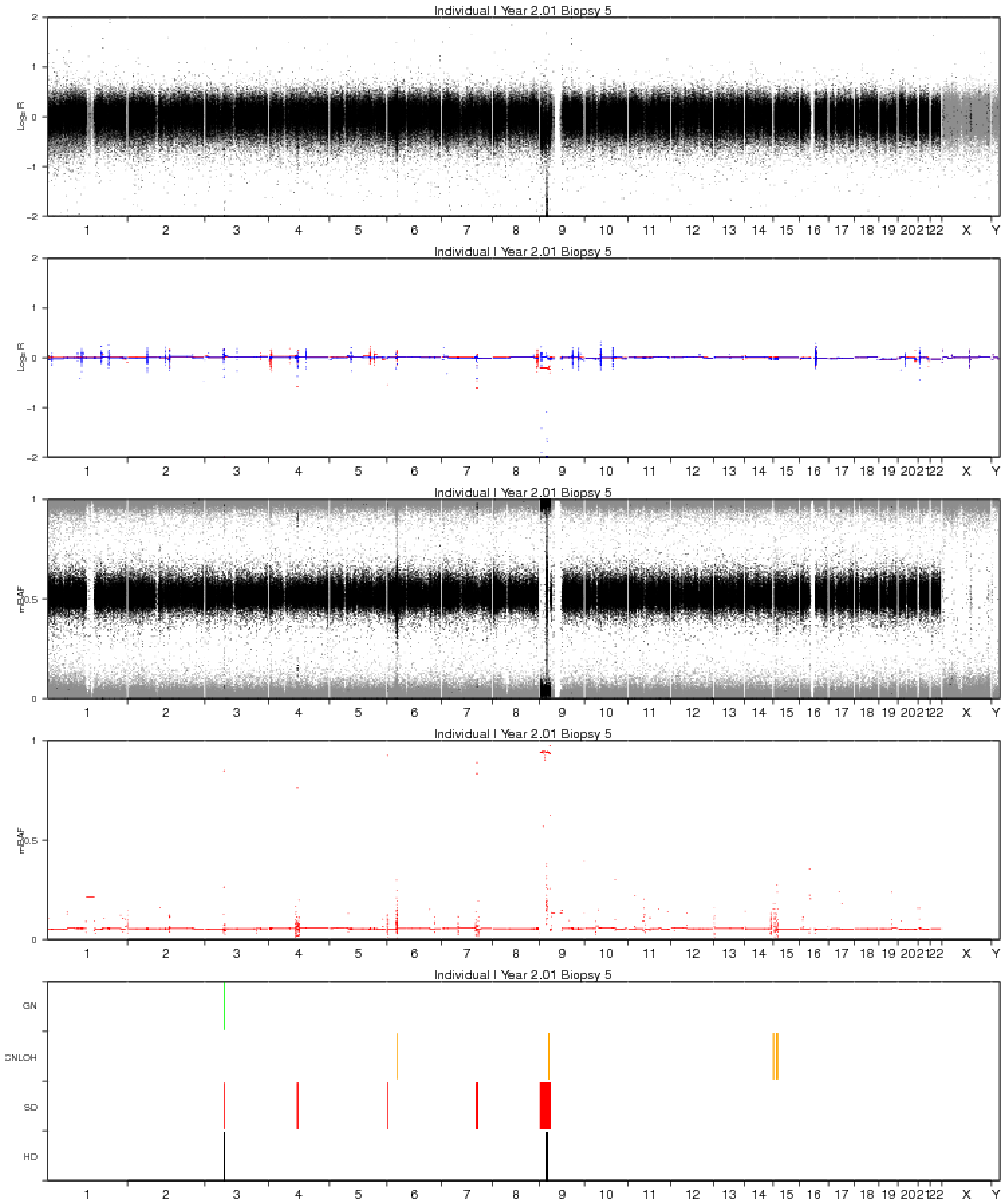
Individual I Year 2.01 Biopsy 5
Log R
mBAF
GN
CNLOH
SD
HD
1 2 3 4 5 6 7 8 9 10 11 12 13 14 15 16 17 18 19 20 21 22 X Y

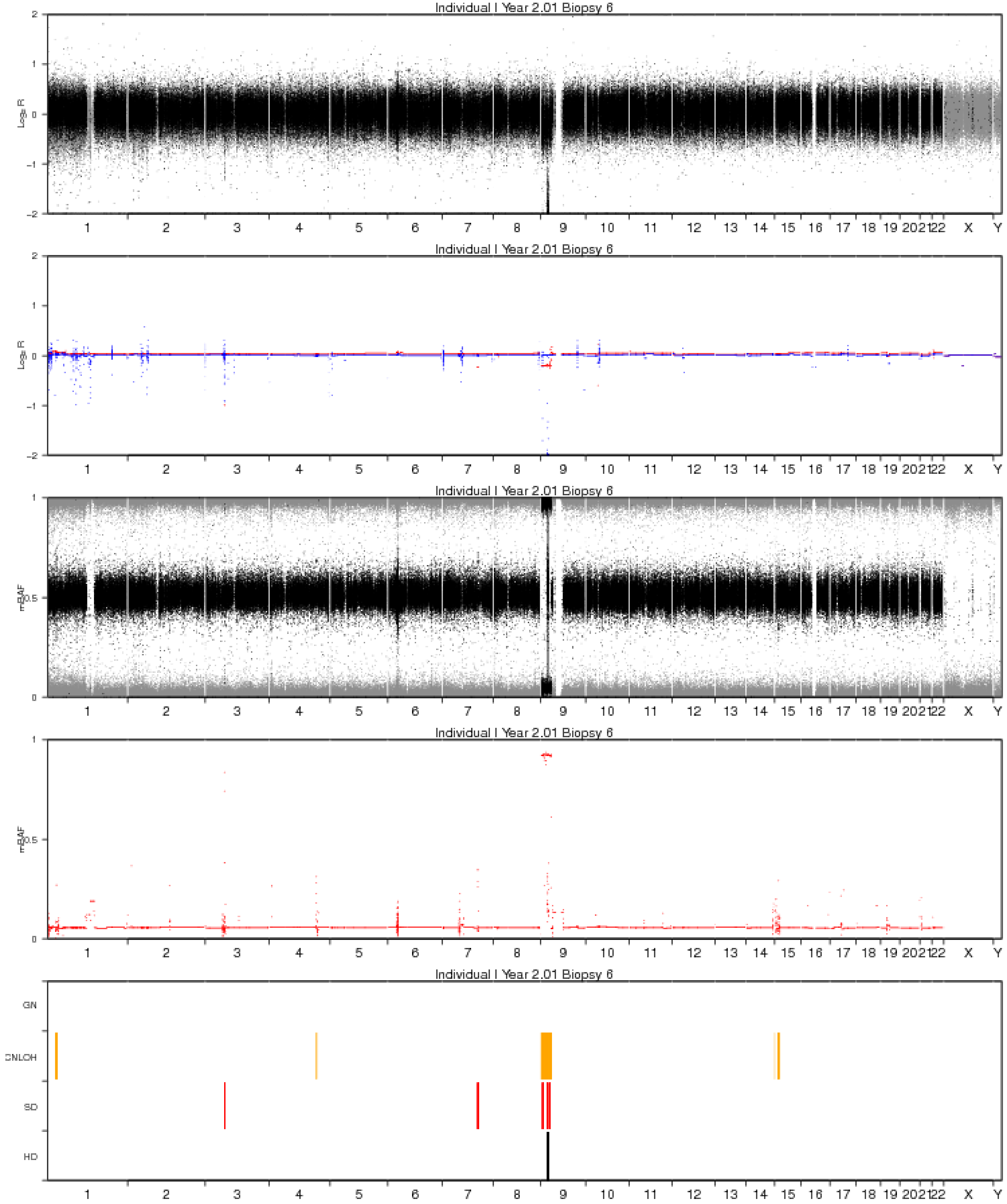
Individual I Year 2.01 Biopsy 6
Log R
mBAF
GN
CNLOH
SD
HD
1 2 3 4 5 6 7 8 9 10 11 12 13 14 15 16 17 18 19 20 21 22 X Y

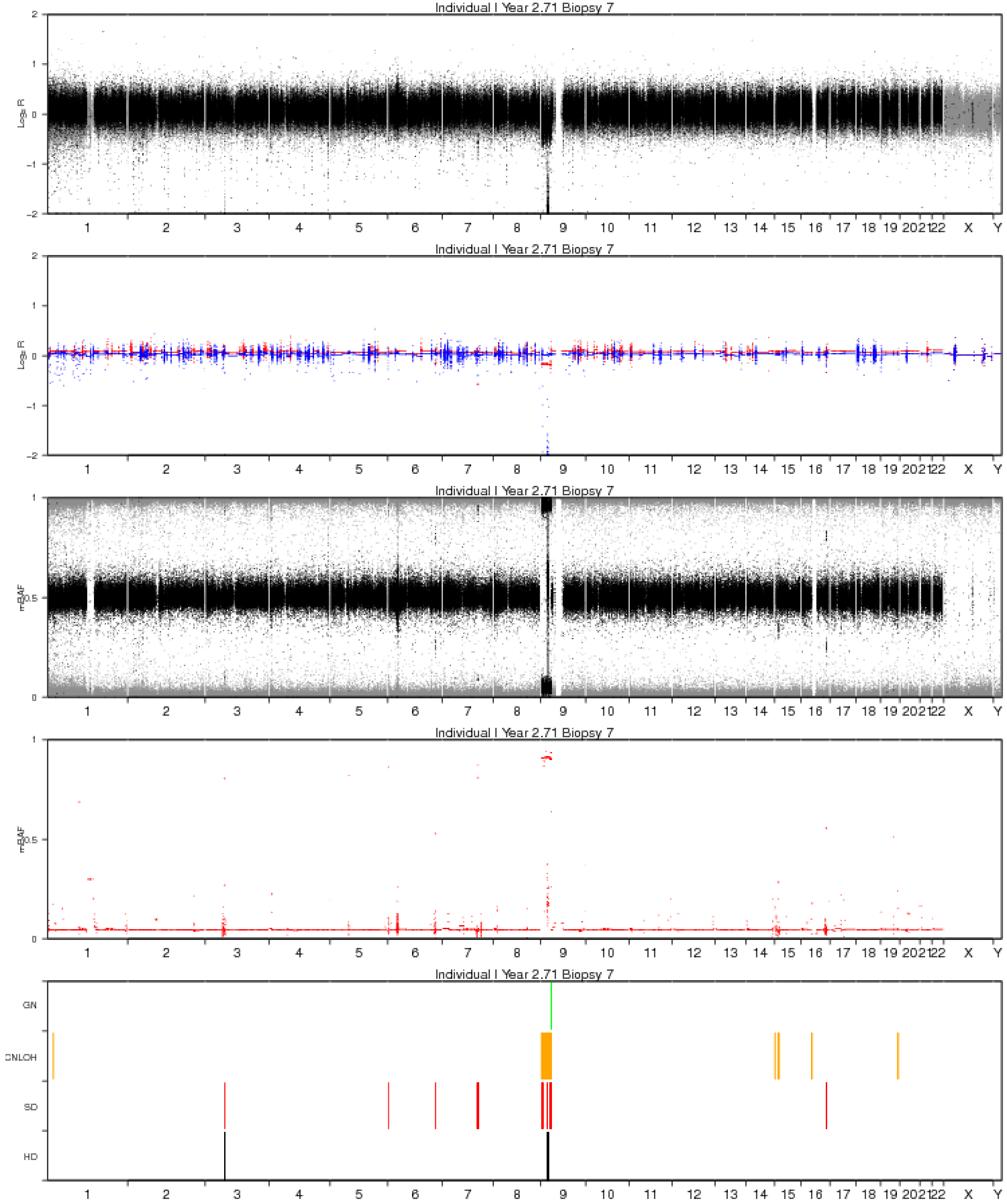
Individual I Year 2.71 Biopsy 7
Log R
mBAF
GN
CNLOH
SD
HD
1 2 3 4 5 6 7 8 9 10 11 12 13 14 15 16 17 18 19 20 21 22 X Y

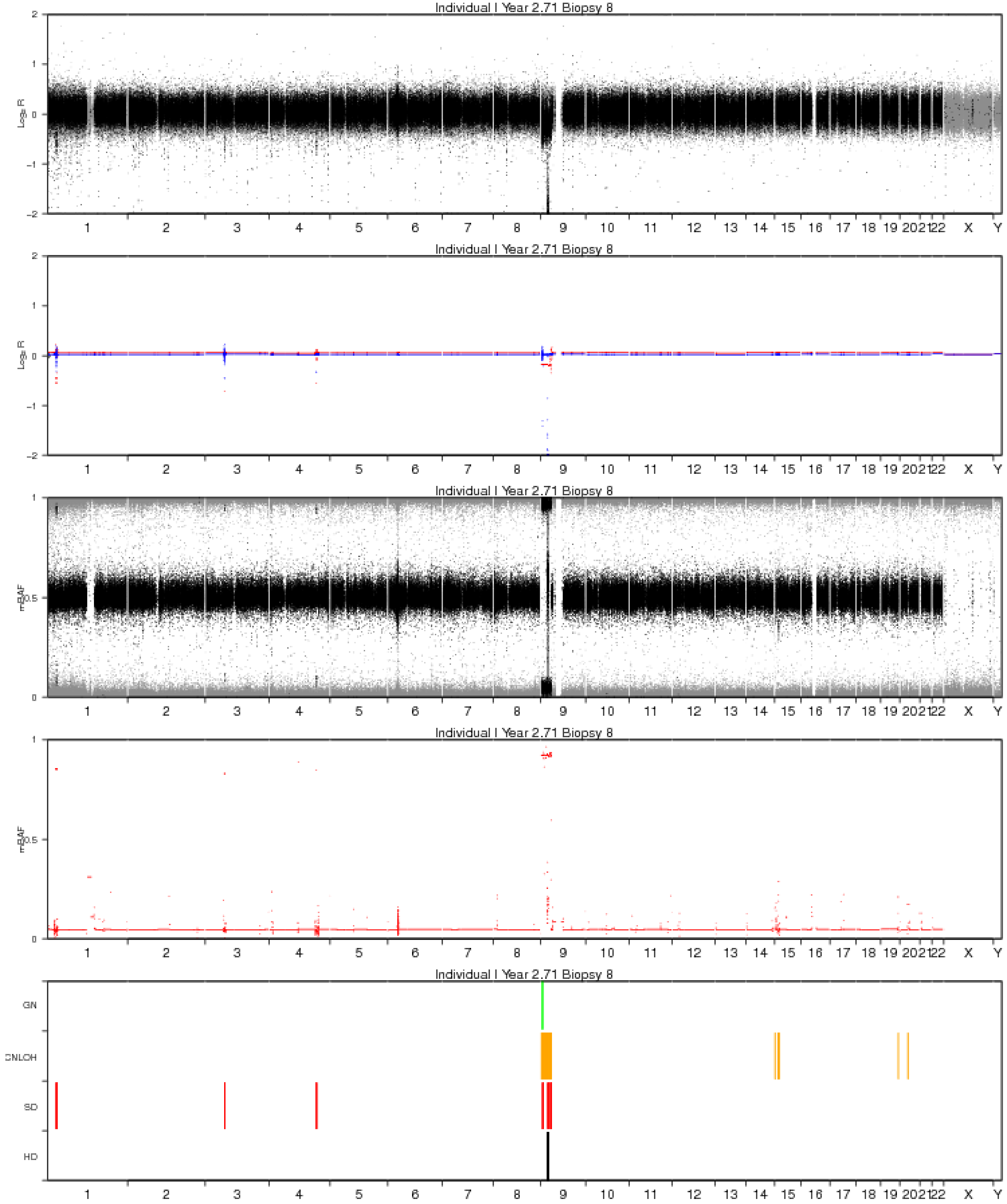
Individual I Year 2.71 Biopsy 8
Log2 R
mBAF
GN
CNLOH
SD
HD
1 2 3 4 5 6 7 8 9 10 11 12 13 14 15 16 17 18 19 20 21 22 X Y

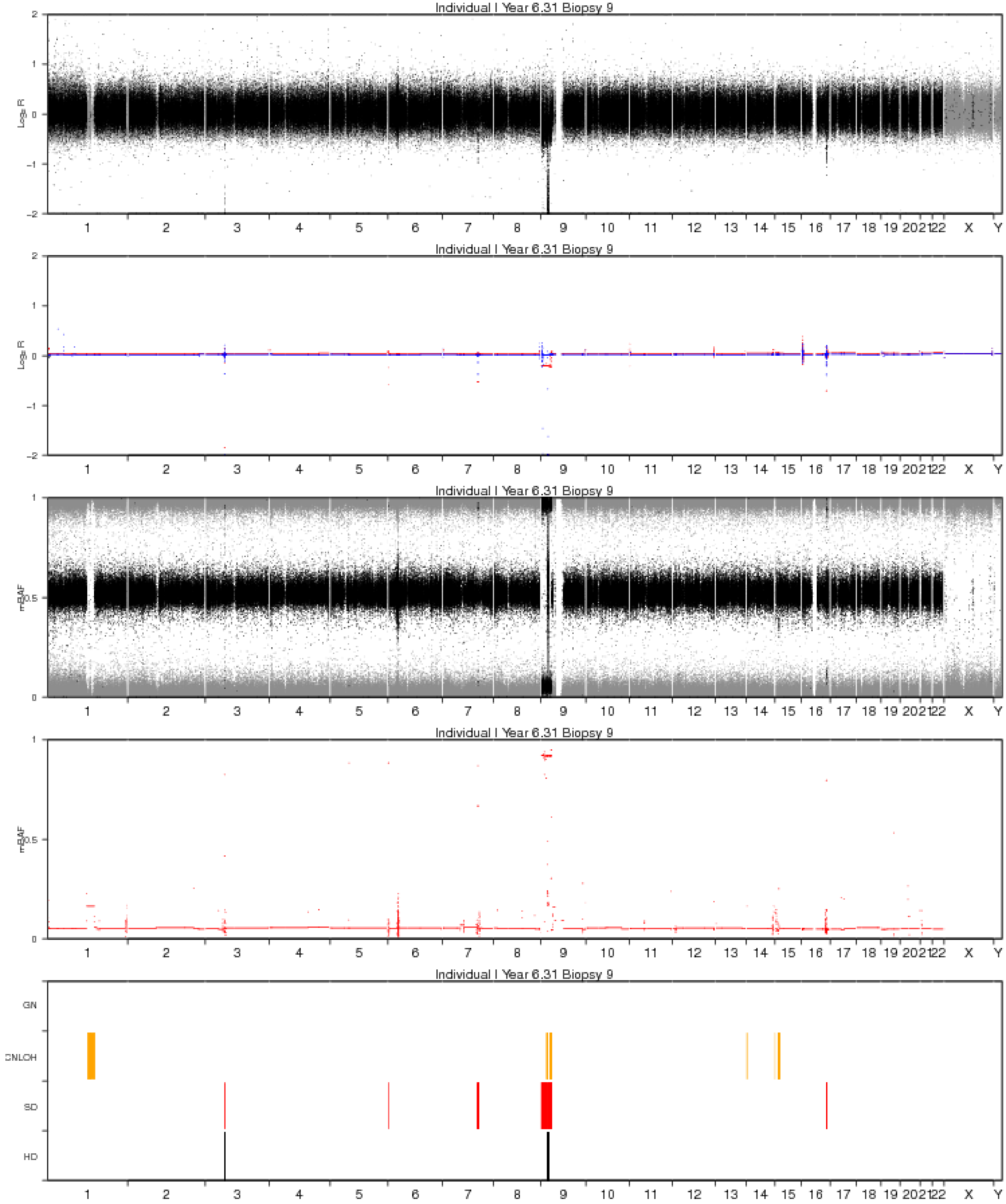

Individual I Year 6.31 Biopsy 9
Log R
mBAF
GN
CNLOH
SD
HD
1 2 3 4 5 6 7 8 9 10 11 12 13 14 15 16 17 18 19 20 21 22 X Y

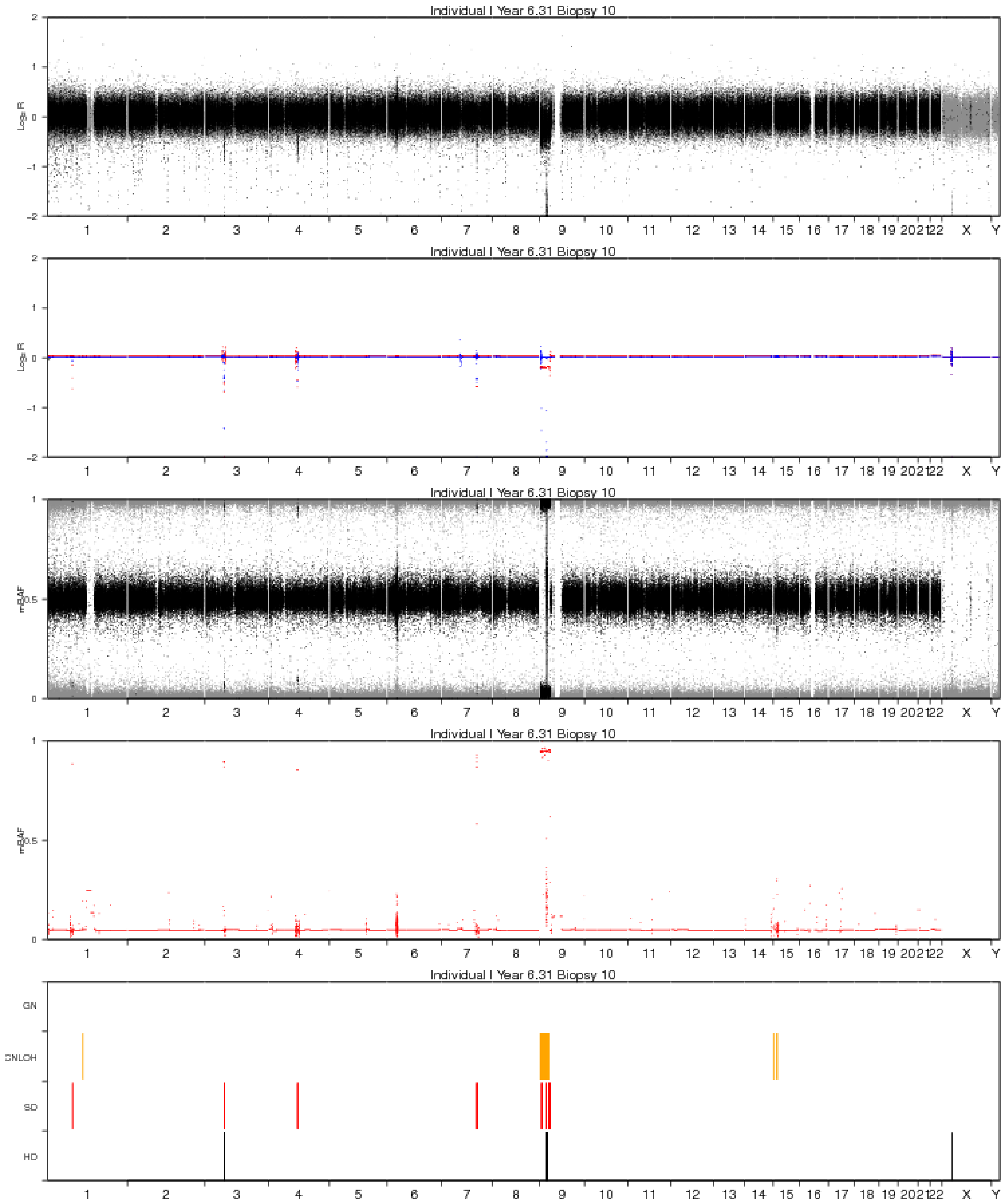
Individual I Year 6.31 Biopsy 10
Log R
2
1
0
−1
−2
1 2 3 4 5 6 7 8 9 10 11 12 13 14 15 16 17 18 19 20 21 22 X Y
Individual I Year 6.31 Biopsy 10
Log R
2
1
0
−1
−2
1 2 3 4 5 6 7 8 9 10 11 12 13 14 15 16 17 18 19 20 21 22 X Y
Individual I Year 6.31 Biopsy 10
mBAF
1
0.5
0
1 2 3 4 5 6 7 8 9 10 11 12 13 14 15 16 17 18 19 20 21 22 X Y
Individual I Year 6.31 Biopsy 10
mBAF
1
0.5
0
1 2 3 4 5 6 7 8 9 10 11 12 13 14 15 16 17 18 19 20 21 22 X Y
Individual I Year 6.31 Biopsy 10
GN
CNLOH
SD
HD
1 2 3 4 5 6 7 8 9 10 11 12 13 14 15 16 17 18 19 20 21 22 X Y

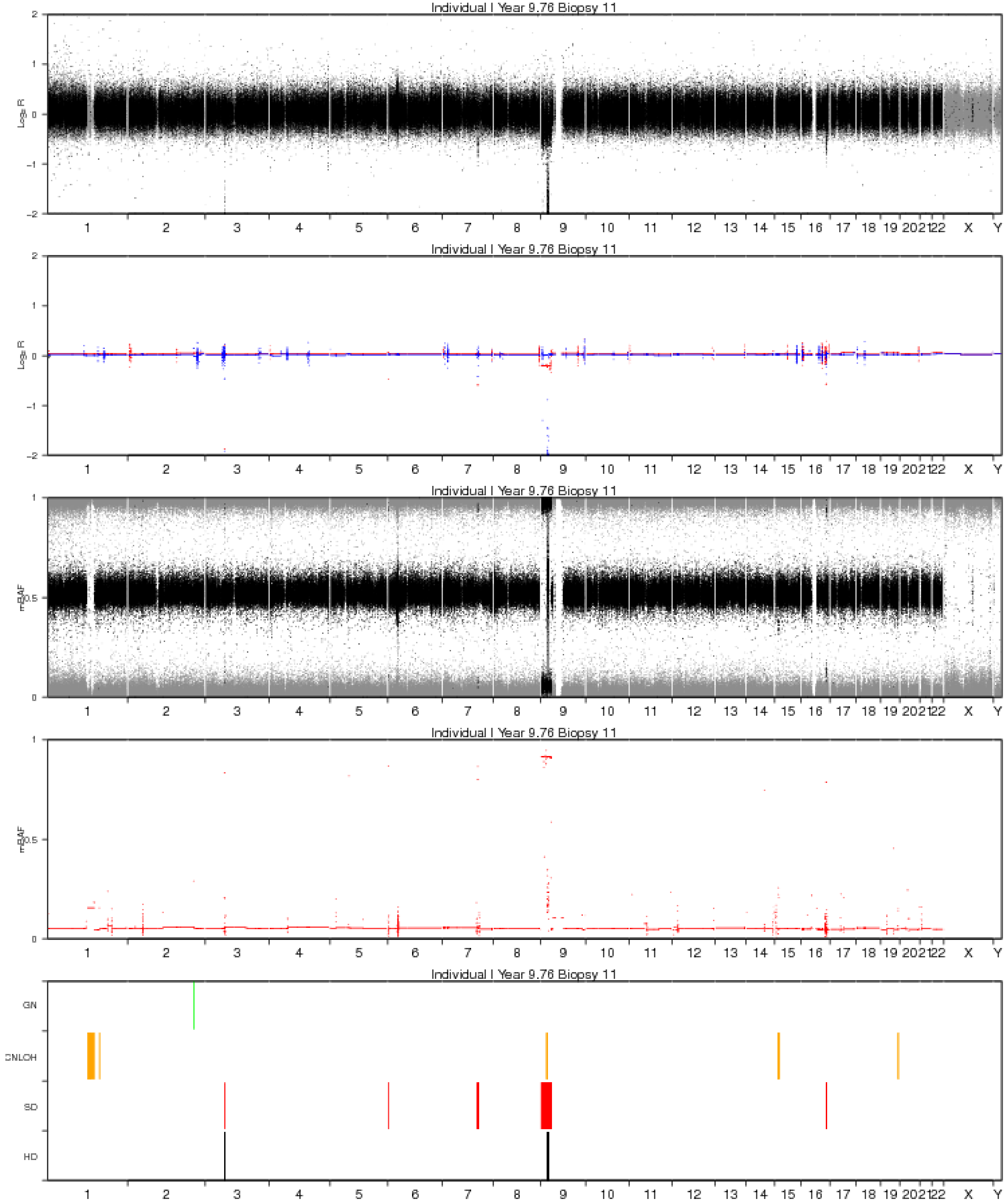
Individual I Year 9.76 Biopsy 11
Log R
mBAF
GN
CNLOH
SD
HD
1 2 3 4 5 6 7 8 9 10 11 12 13 14 15 16 17 18 19 20 21 22 X Y

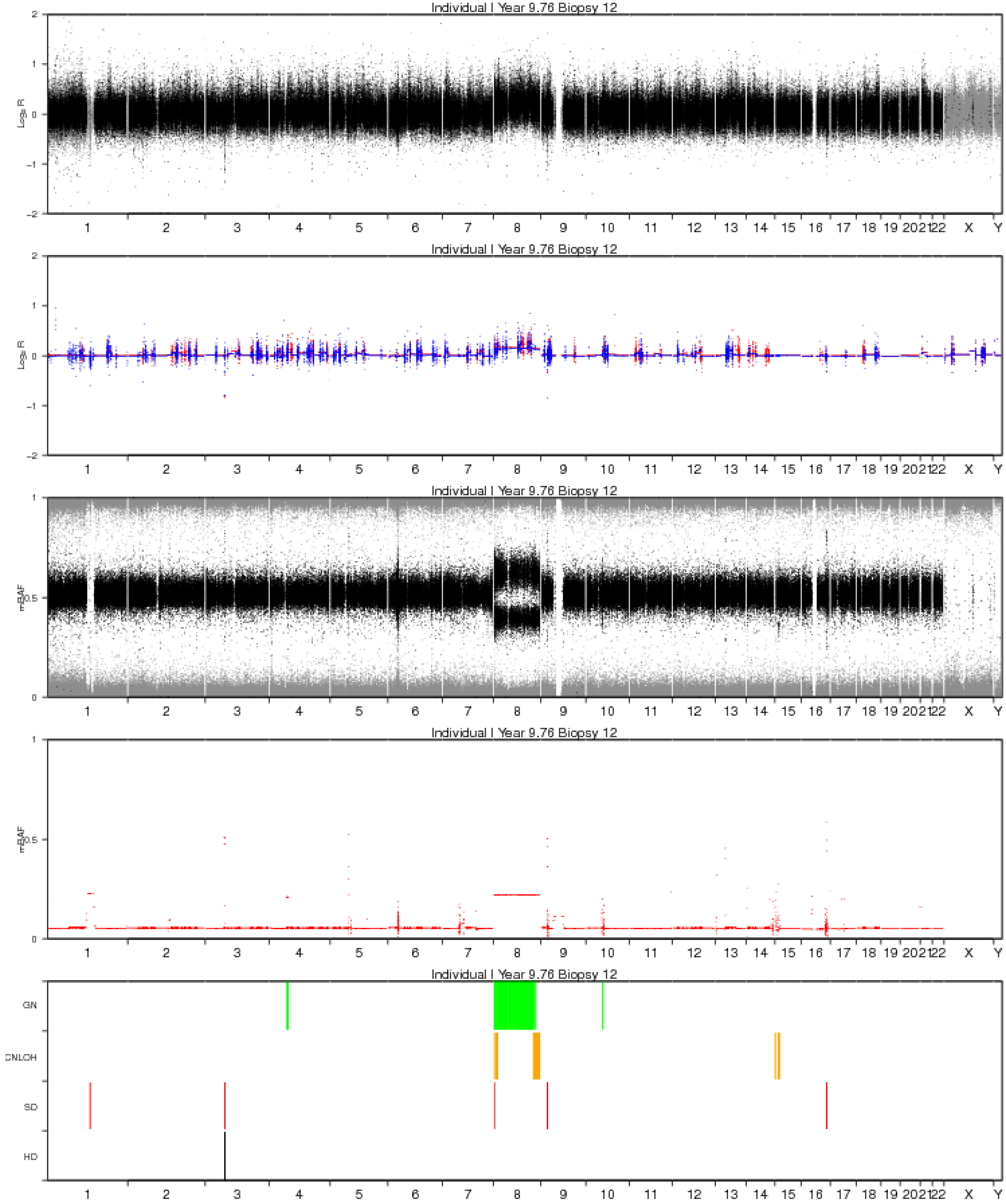
Individual I Year 9.76 Biopsy 12
Log R
mBAF
GN
CNLOH
SD
HD
1 2 3 4 5 6 7 8 9 10 11 12 13 14 15 16 17 18 19 20 21 22 X Y

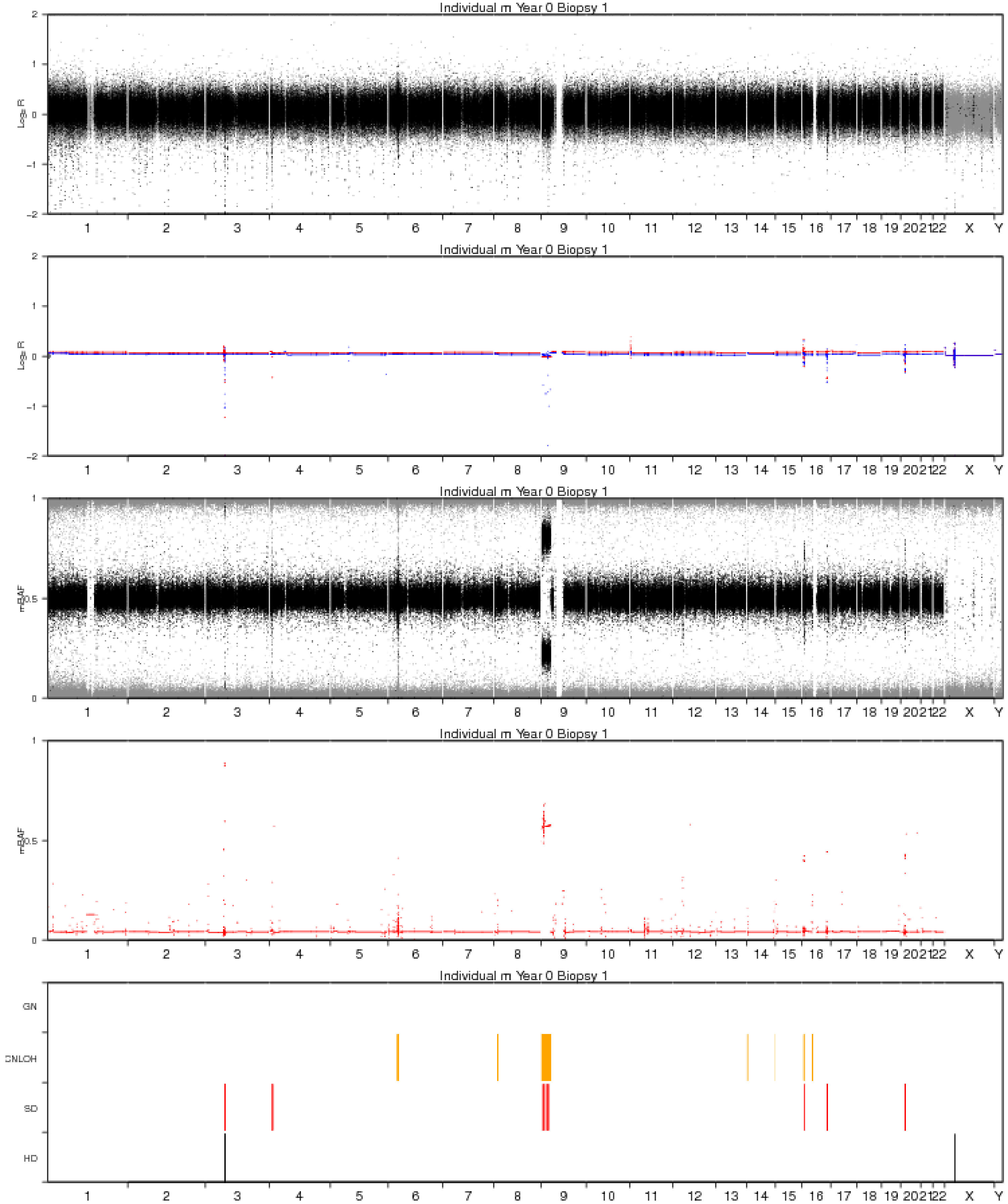
Individual m Year 0 Biopsy 1
Log R
mBAF
GN
CNLOH
SD
HD
1 2 3 4 5 6 7 8 9 10 11 12 13 14 15 16 17 18 19 20 21 22 X Y

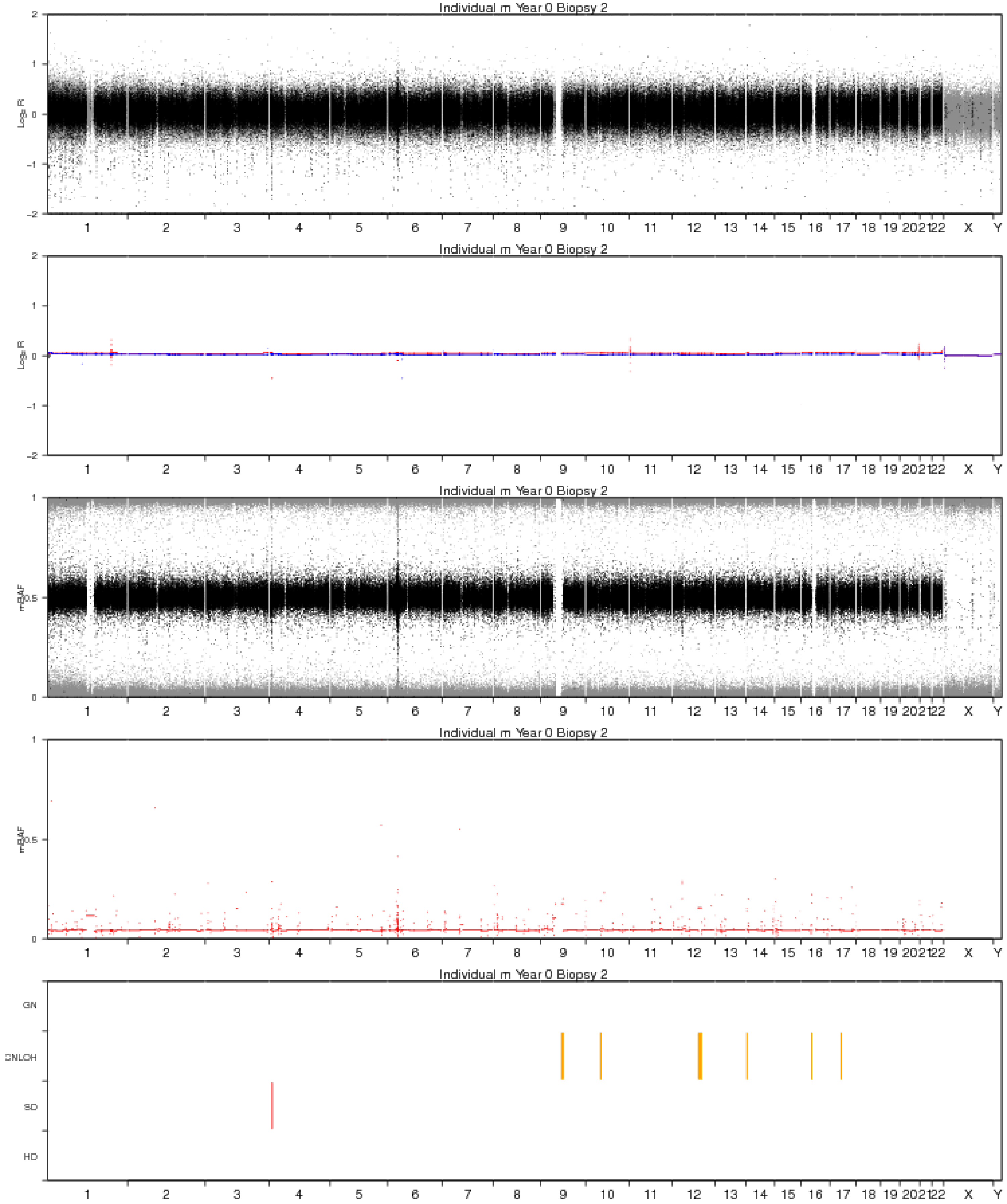
Individual m Year 0 Biopsy 2
Log R
2
1
0
−1
−2
1 2 3 4 5 6 7 8 9 10 11 12 13 14 15 16 17 18 19 20 21 22 X Y
Individual m Year 0 Biopsy 2
Log R
Individual m Year 0 Biopsy 2
mBAF
1
0.5
0
Individual m Year 0 Biopsy 2
mBAF
Individual m Year 0 Biopsy 2
GN
CNLOH
SD
HD

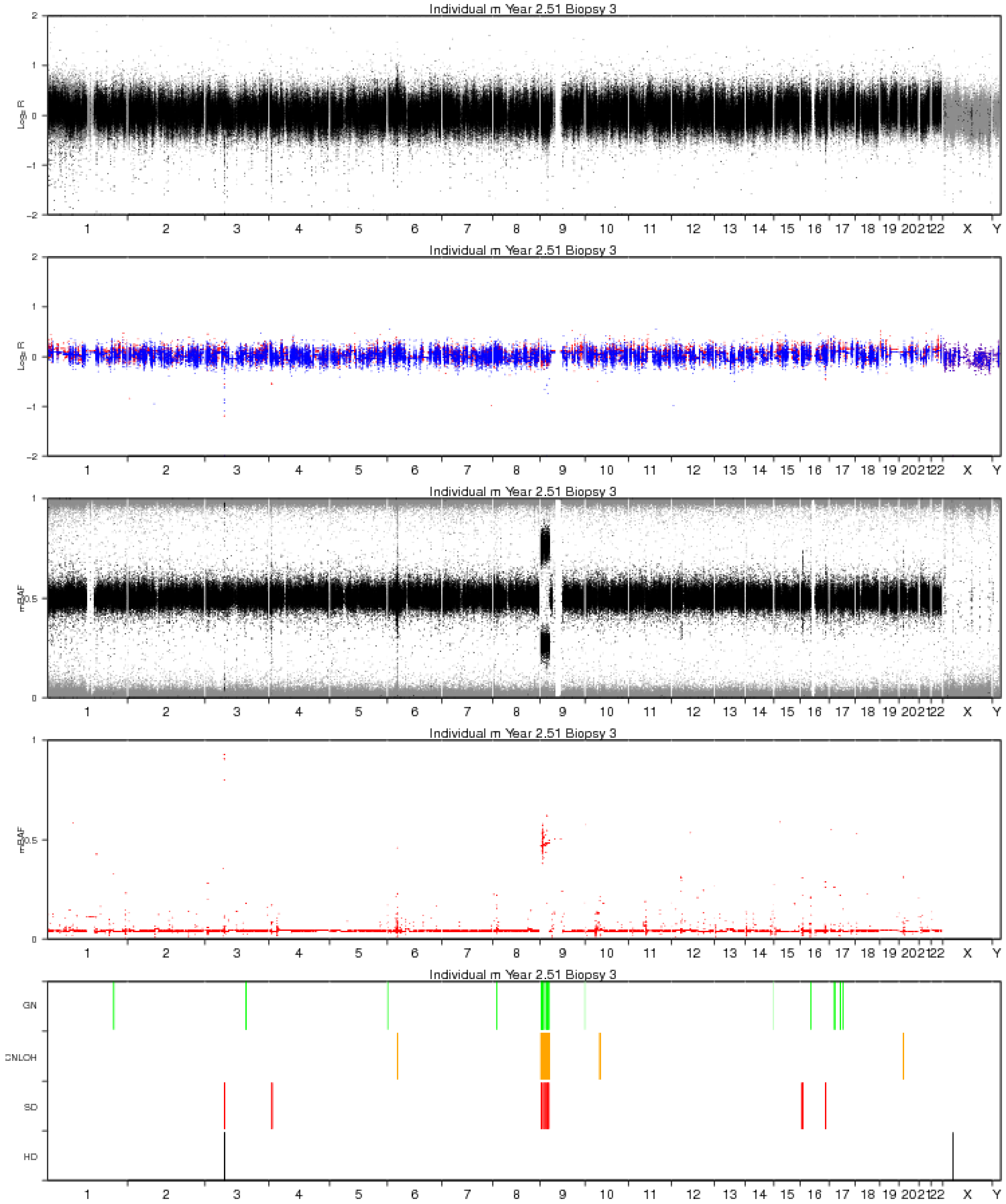

Individual m Year 2.51 Biopsy 3
Log2 R
2
1
0
-1
-2
1 2 3 4 5 6 7 8 9 10 11 12 13 14 15 16 17 18 19 20 21 22 X Y
Individual m Year 2.51 Biopsy 3
Log2 R
2
1
0
-1
-2
1 2 3 4 5 6 7 8 9 10 11 12 13 14 15 16 17 18 19 20 21 22 X Y
Individual m Year 2.51 Biopsy 3
mBAF
1
0.5
0
1 2 3 4 5 6 7 8 9 10 11 12 13 14 15 16 17 18 19 20 21 22 X Y
Individual m Year 2.51 Biopsy 3
mBAF
1
0.5
0
1 2 3 4 5 6 7 8 9 10 11 12 13 14 15 16 17 18 19 20 21 22 X Y
Individual m Year 2.51 Biopsy 3
GN
CNLOH
SD
HD
1 2 3 4 5 6 7 8 9 10 11 12 13 14 15 16 17 18 19 20 21 22 X Y

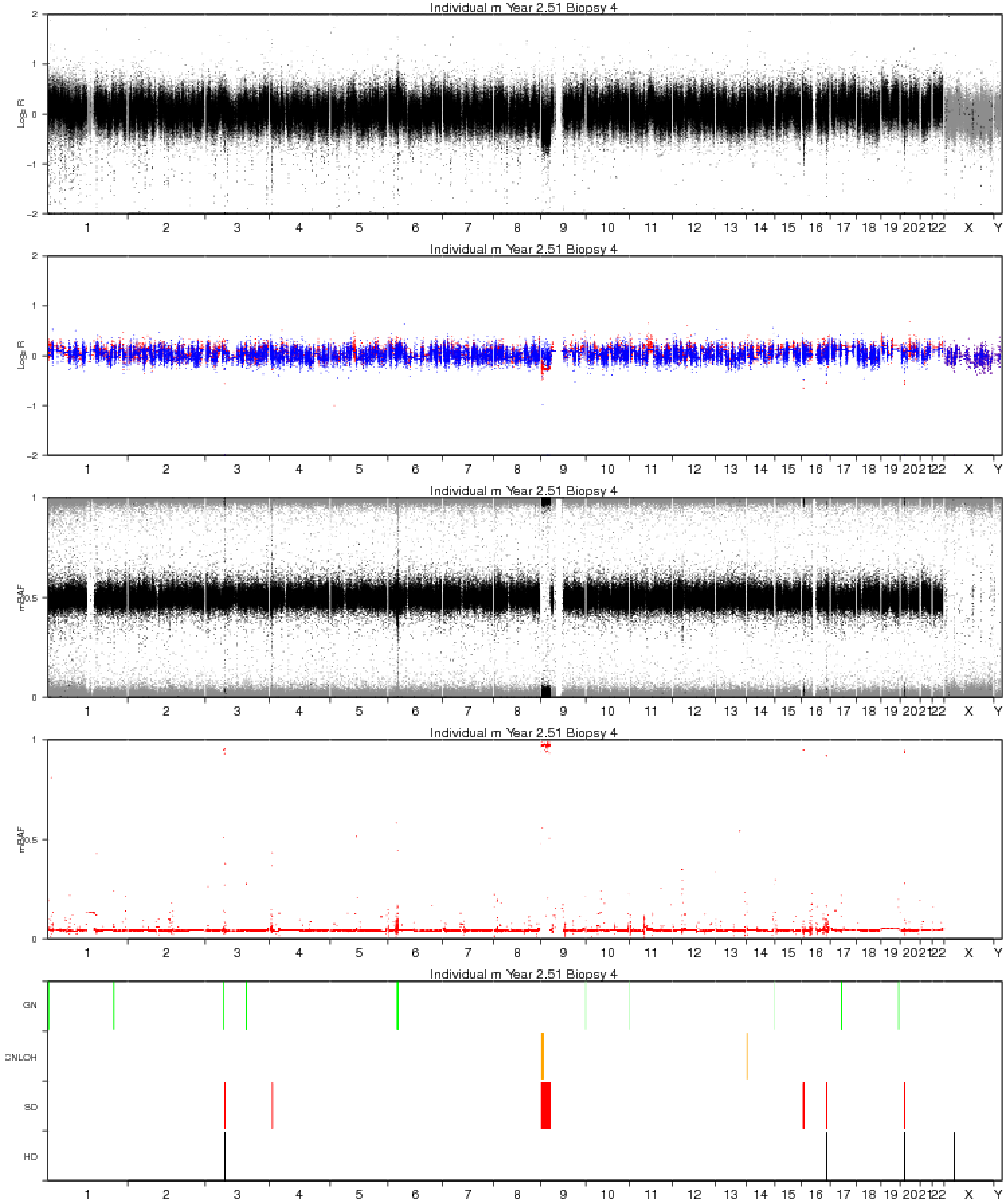
Individual m Year 2.51 Biopsy 4
Log R
mBAF
GN
CNLOH
SD
HD
1 2 3 4 5 6 7 8 9 10 11 12 13 14 15 16 17 18 19 20 21 22 X Y

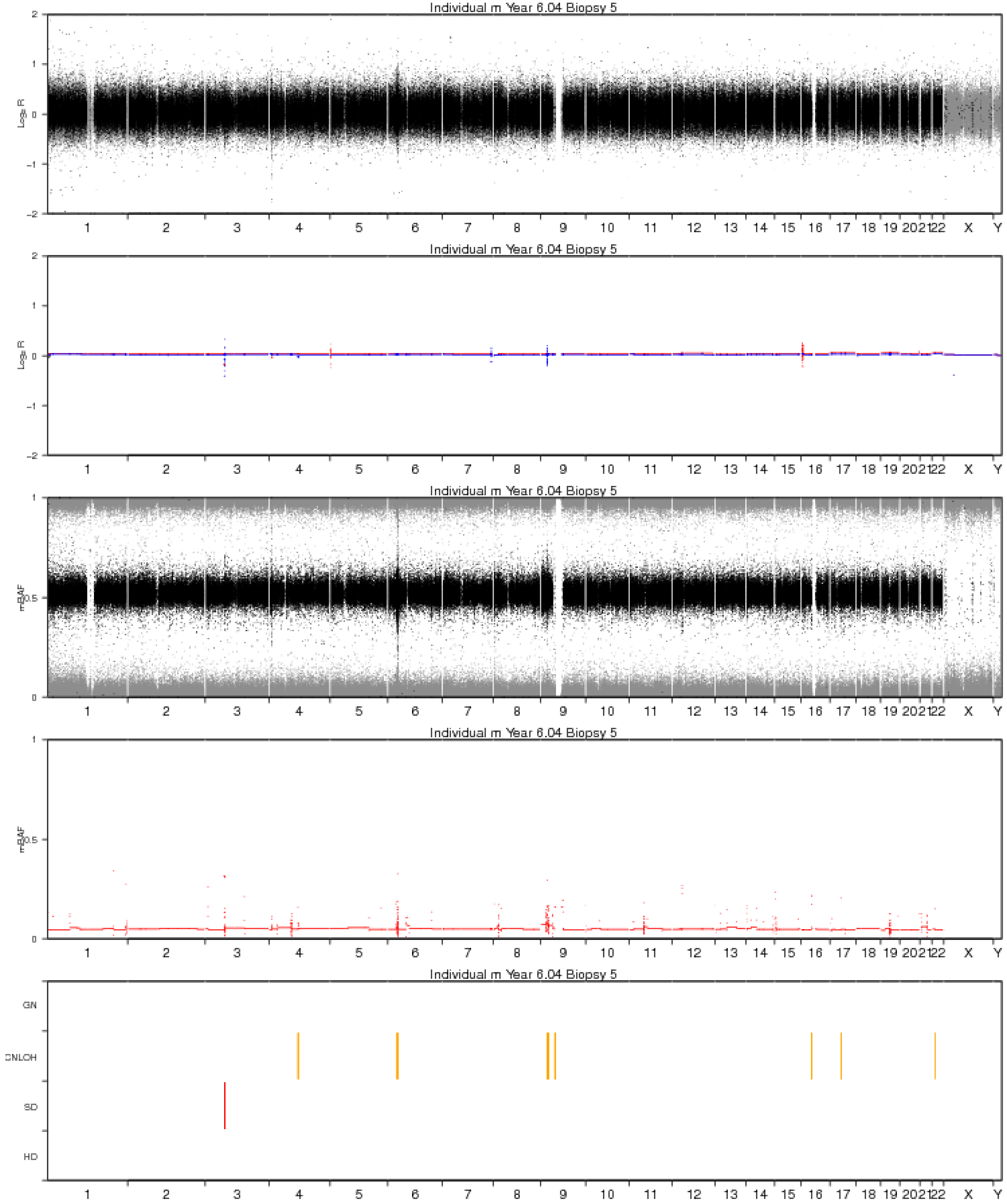
Individual m Year 6.04 Biopsy 5
Log2 R
mBAF
GN
CNLOH
SD
HD
1
2
3
4
5
6
7
8
9
10
11
12
13
14
15
16
17
18
19
20
21
22
X
Y

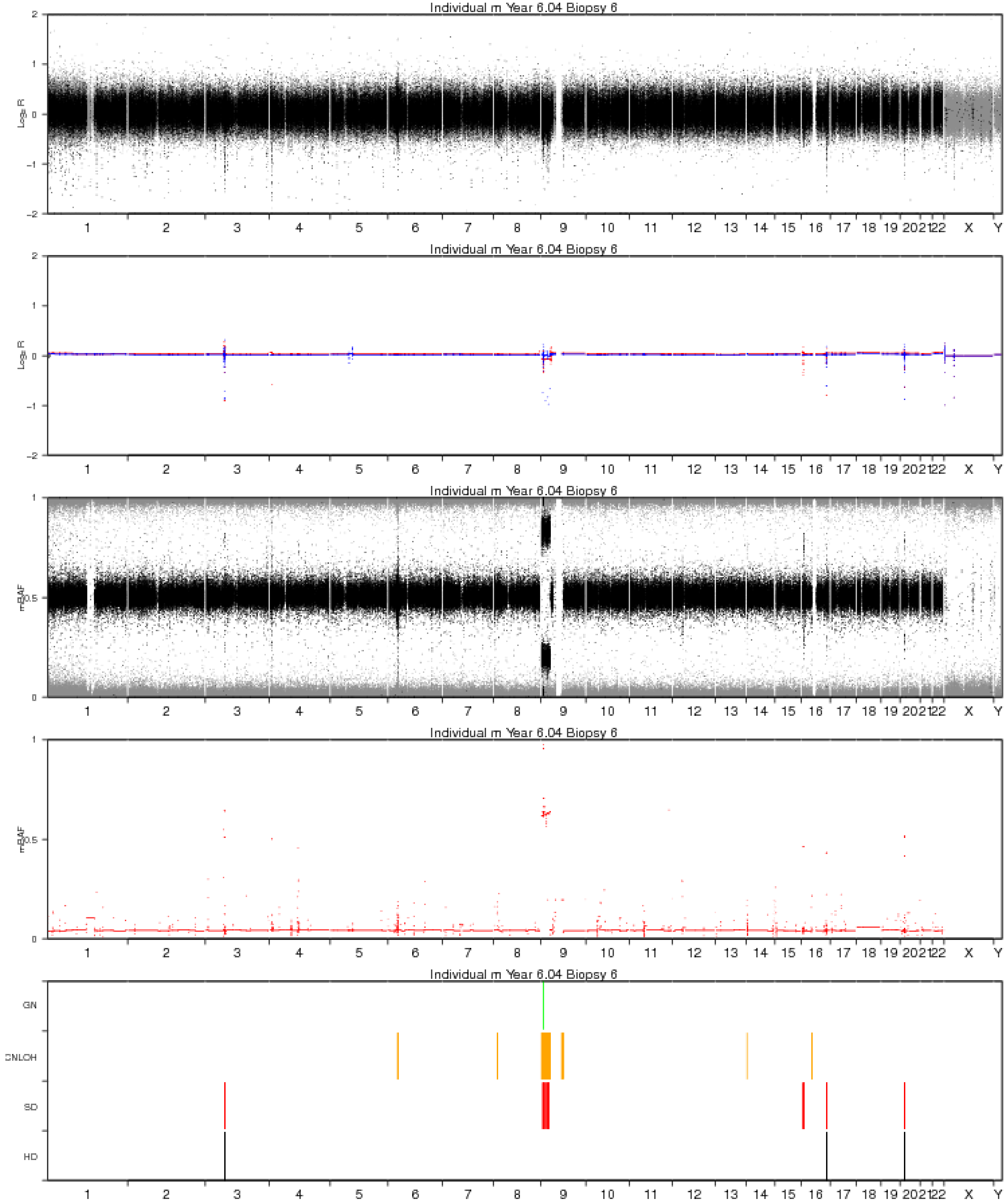
Individual m Year 6.04 Biopsy 6
Log R
mBAF
GN
CNLOH
SD
HD
1 2 3 4 5 6 7 8 9 10 11 12 13 14 15 16 17 18 19 20 21 22 X Y

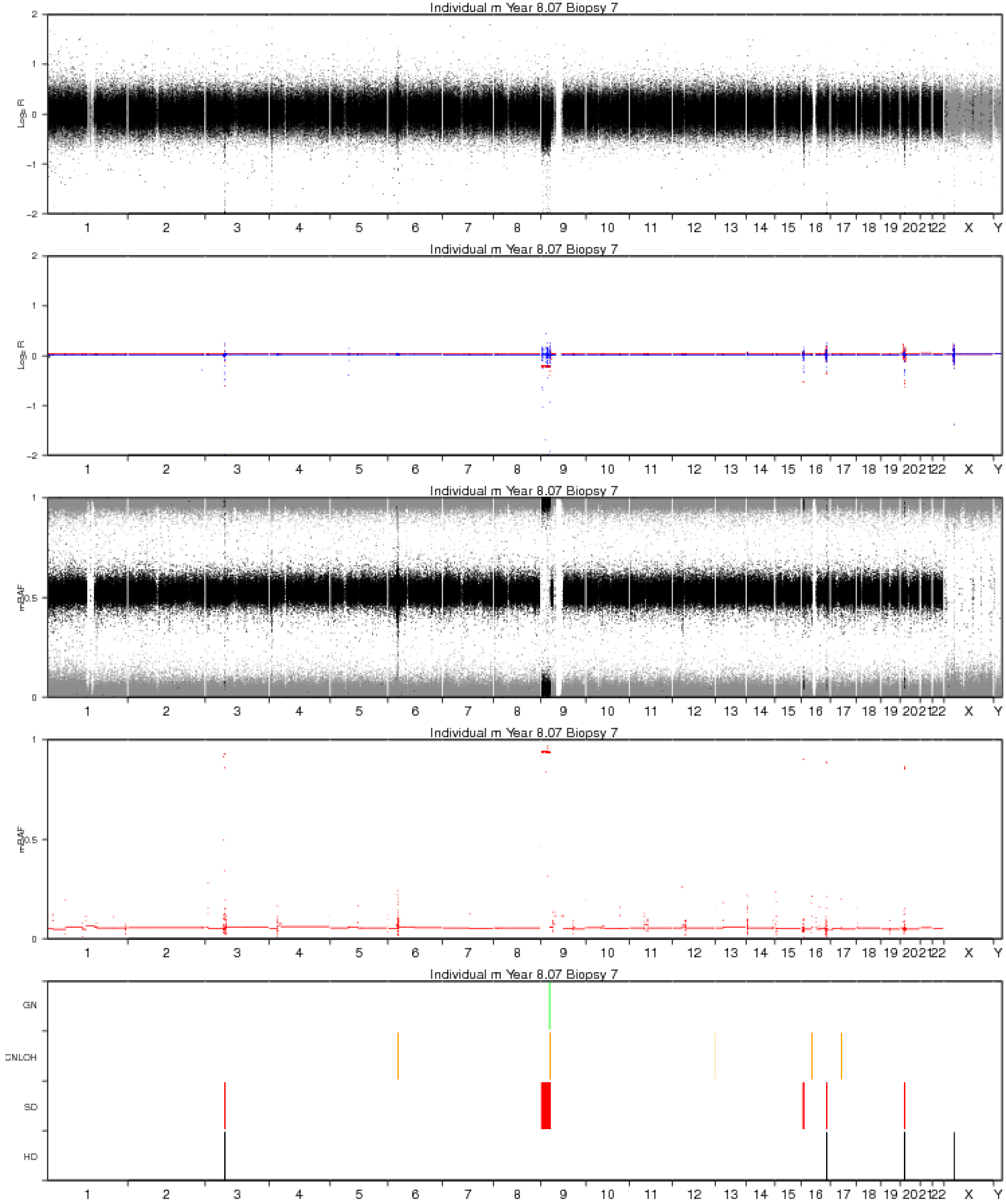

Individual m Year 8.07 Biopsy 7
Log R
mBAF
GN
CNLOH
SD
HD
1
2
3
4
5
6
7
8
9
10
11
12
13
14
15
16
17
18
19
20
21
22
X
Y

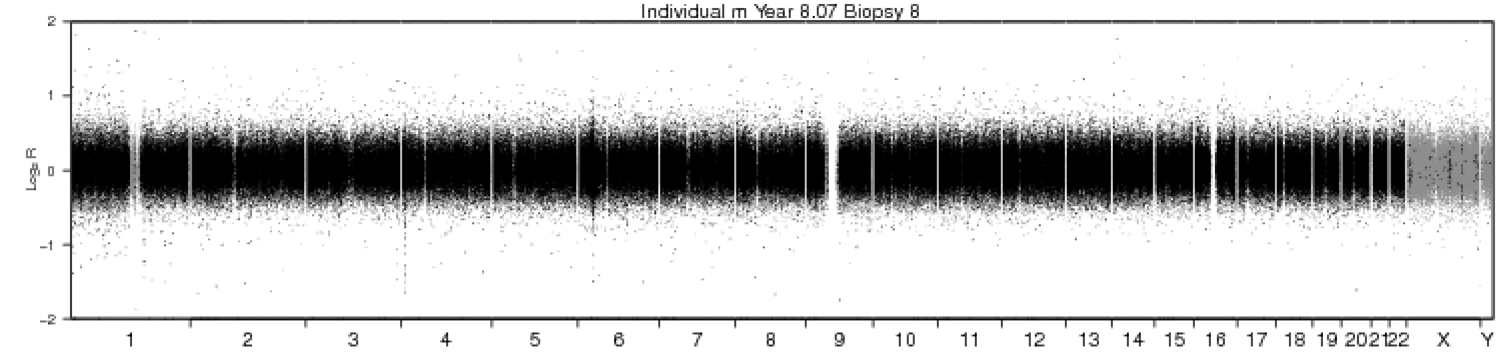
Individual m Year 8.07 Biopsy 8
Log2 R
2
1
0
−1
−2
1 2 3 4 5 6 7 8 9 10 11 12 13 14 15 16 17 18 19 20 21 22 X Y

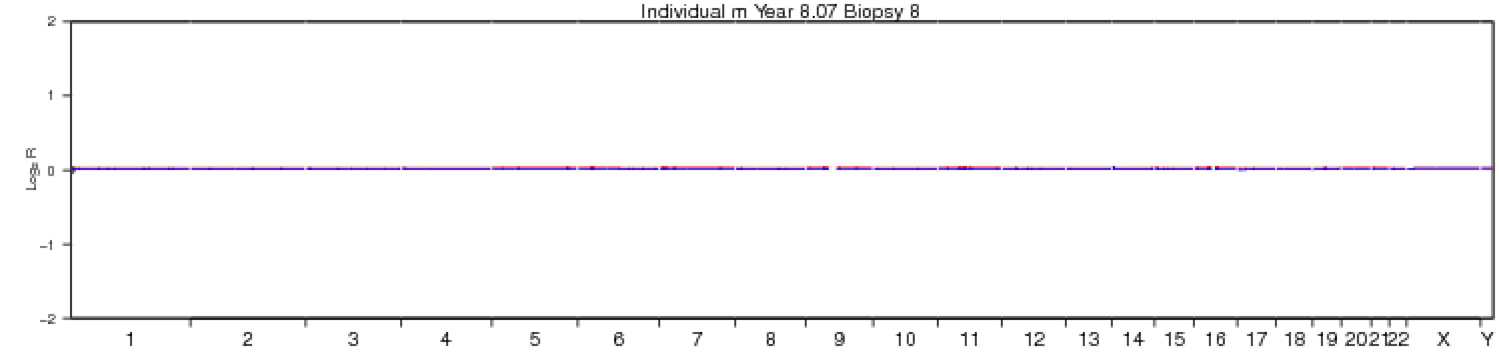
Individual m Year 8.07 Biopsy 8
Log2 R
2
1
0
−1
−2
1 2 3 4 5 6 7 8 9 10 11 12 13 14 15 16 17 18 19 20 21 22 X Y

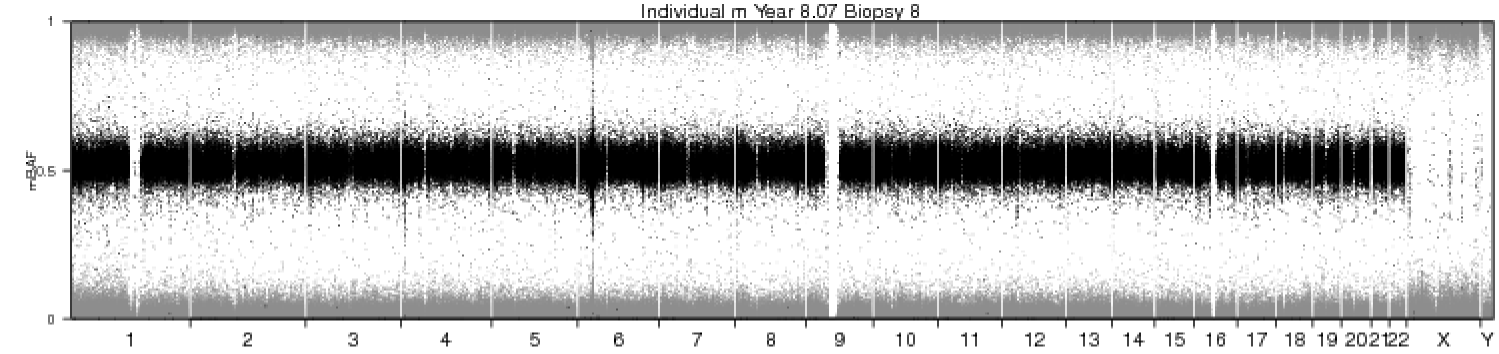
Individual m Year 8.07 Biopsy 8
mBAF
1
0.5
0
1 2 3 4 5 6 7 8 9 10 11 12 13 14 15 16 17 18 19 20 21 22 X Y

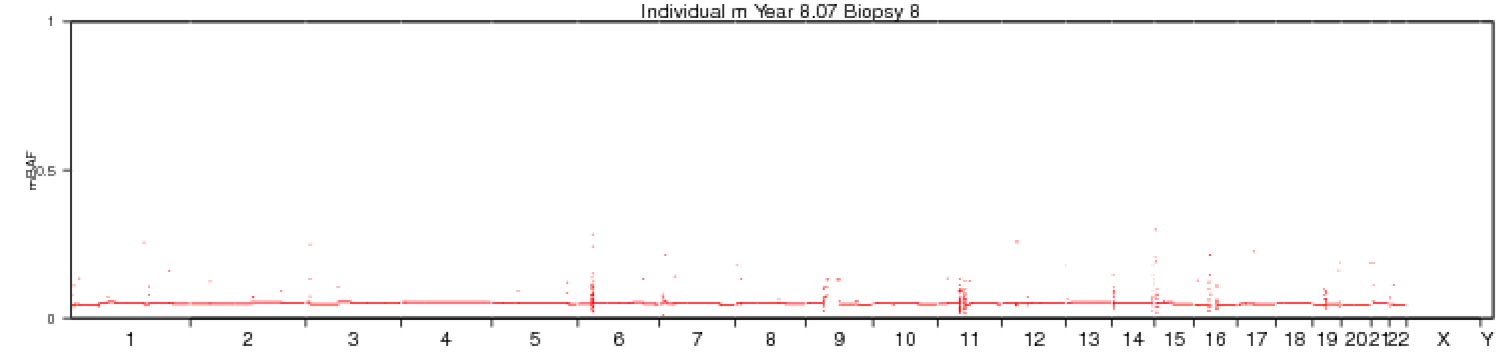
Individual m Year 8.07 Biopsy 8
mBAF
1
0.5
0
1 2 3 4 5 6 7 8 9 10 11 12 13 14 15 16 17 18 19 20 21 22 X Y

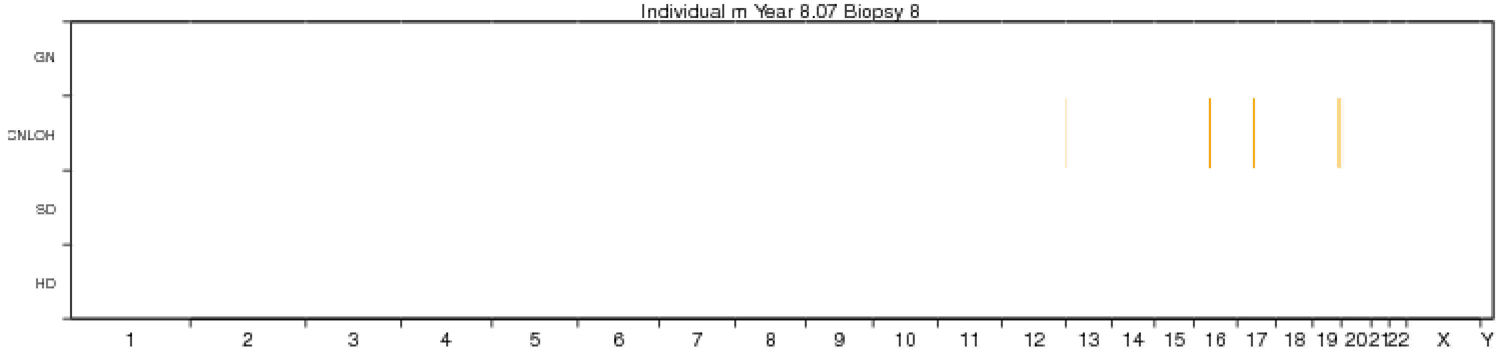
Individual m Year 8.07 Biopsy 8
GN
CNLOH
SD
HD
1 2 3 4 5 6 7 8 9 10 11 12 13 14 15 16 17 18 19 20 21 22 X Y

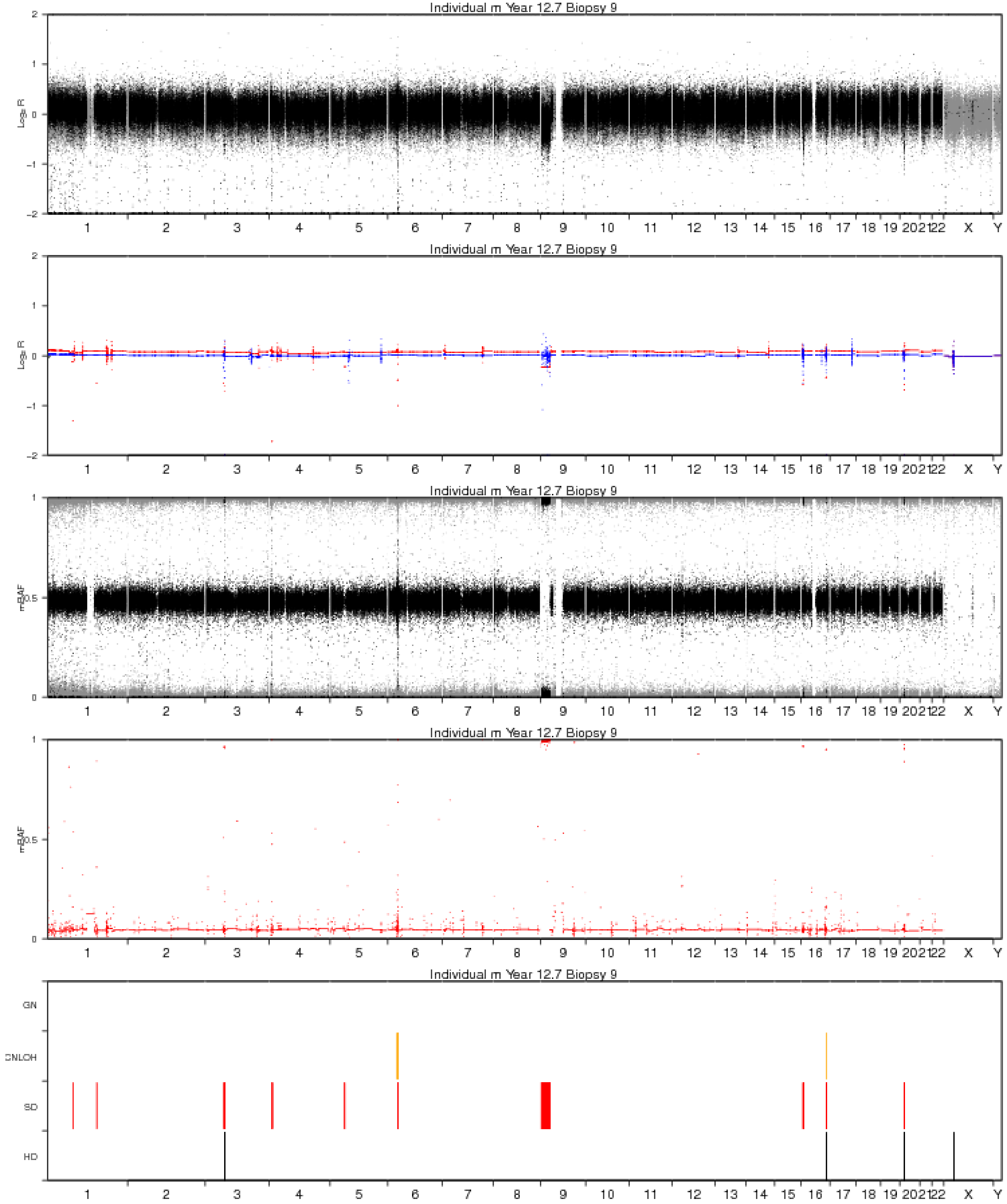
Individual m Year 12.7 Biopsy 9
Log R
2
1
0
−1
−2
1 2 3 4 5 6 7 8 9 10 11 12 13 14 15 16 17 18 19 20 21 22 X Y
Individual m Year 12.7 Biopsy 9
Log R
2
1
0
−1
−2
1 2 3 4 5 6 7 8 9 10 11 12 13 14 15 16 17 18 19 20 21 22 X Y
Individual m Year 12.7 Biopsy 9
mBAF
1
0.5
0
1 2 3 4 5 6 7 8 9 10 11 12 13 14 15 16 17 18 19 20 21 22 X Y
Individual m Year 12.7 Biopsy 9
mBAF
1
0.5
0
1 2 3 4 5 6 7 8 9 10 11 12 13 14 15 16 17 18 19 20 21 22 X Y
Individual m Year 12.7 Biopsy 9
GN
CNLOH
SD
HD
1 2 3 4 5 6 7 8 9 10 11 12 13 14 15 16 17 18 19 20 21 22 X Y

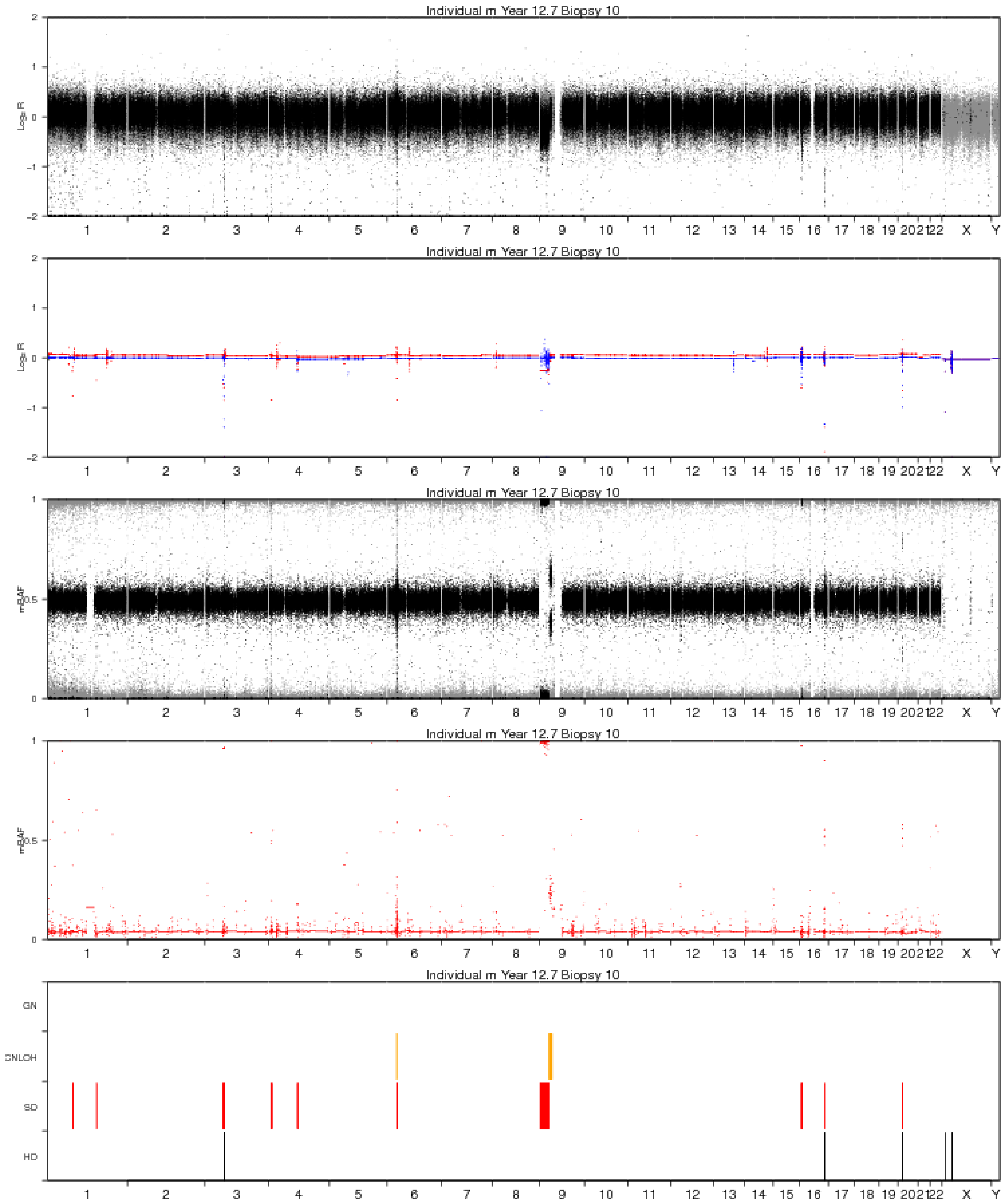
Individual m Year 12.7 Biopsy 10
Log2 R
mBAF
GN
CNLOH
SD
HD
1 2 3 4 5 6 7 8 9 10 11 12 13 14 15 16 17 18 19 20 21 22 X Y
