# Supplementary figures and images for: NSAIDs Modulate Clonal Evolution in Barrett's Esophagus
Source: PLoS Genet. 2013 Jun 13;9(6):e1003553. doi: 10.1371/journal.pgen.1003553 (PMC3681672; doi:10.1371/journal.pgen.1003553)

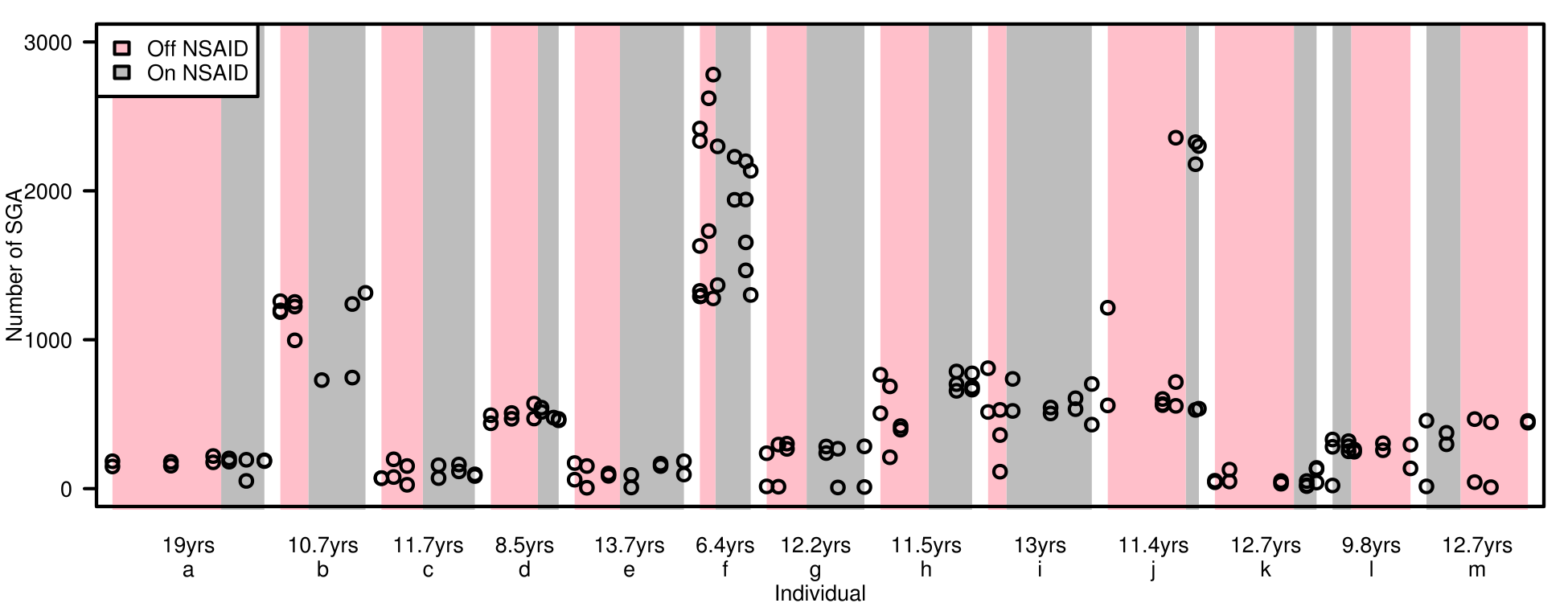

Supplement: Figure S1 — Number of SGA events in every biopsy over time for each individual show that the genomes of biopsies maintain equilibrium in the number of SGA events, with few exceptions (note biopsies with high variation in the number of SGA events in individuals b, f, and j). (TIF) [file pgen.1003553.s002.tif]

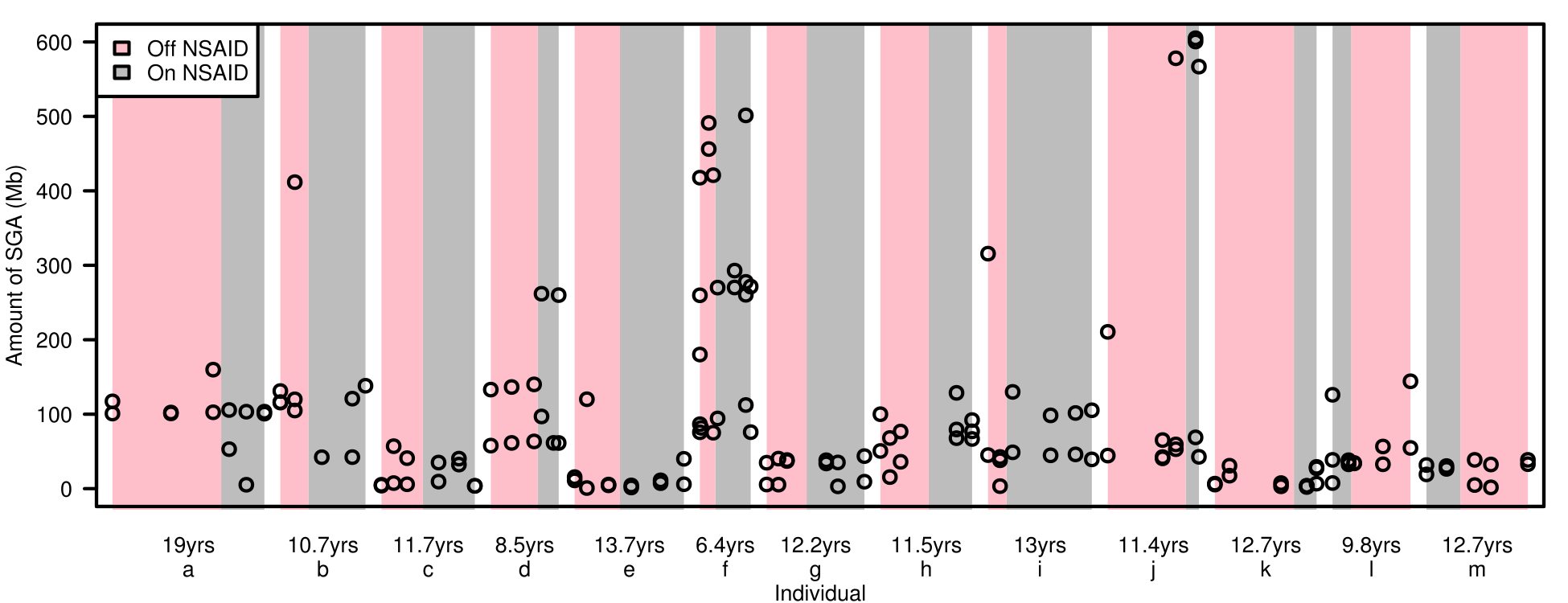

Supplement: Figure S2 — The total amount of SGA (in Mb) in every biopsy over time for each individual show that the genomes of biopsies maintain equilibrium in the total amount of SGA, with few exceptions (note biopsies with high total SGA in individuals b, d, f, and j). (TIF) [file pgen.1003553.s003.tif]

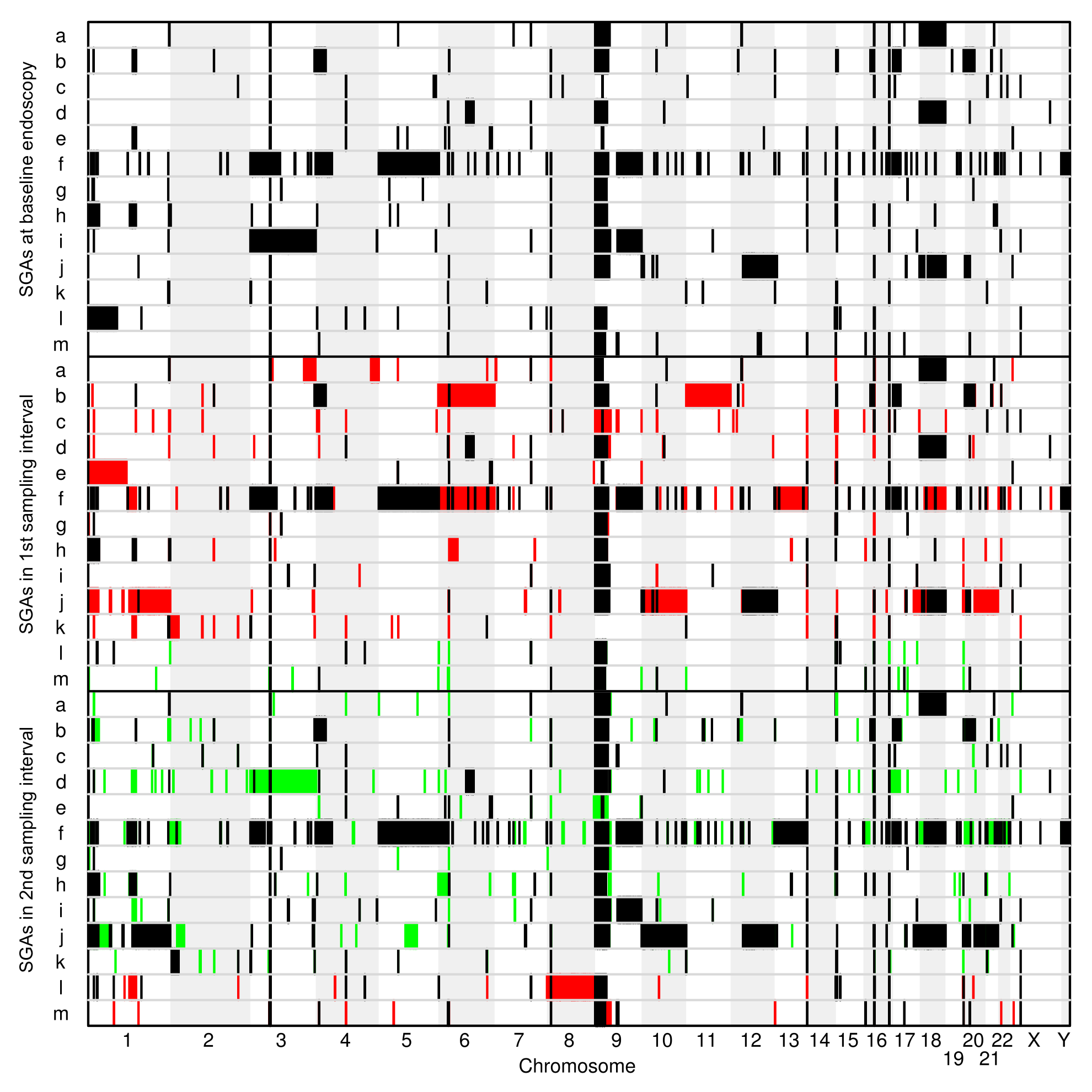

Supplement: Figure S3 — Linear chromosome plot of detected somatic genomic abnormalities (SGAs) in biopsies from the baseline endoscopy (top panel), the first sampling period (off-NSAIDs for a–k and on-NSAIDs for l, m; middle panel), and the second sampling period (on-NSAIDs for a–k and off-NSAIDs for l, m; bottom panel). This plot shows the genomic location and size of newly detected SGAs during off-NSAID (red) and on-NSAID (green) periods that are summarized in Figure 3A. Black bars represent SGAs observed in a prior sampling period or at baseline; top panel – black represents SGAs detected in any biopsy from the baseline endoscopy; middle panel – black represents SGAs detected in any biopsy from baseline endoscopy that is also detected in at least one biopsy in the first sampling period; bottom panel – black represents SGAs detected in any biopsy from baseline or first sampling period, or both, that is also detected in at least one biopsy in the second sampling period. In the middle panel, red and green bars represent newly acquired SGAs that are detected in at least one biopsy in the first sampling period, but not detected at baseline. In the bottom panel, red and green bars represent newly acquired SGAs that are detected in at least one biopsy in the second sampling period, but not detected in any biopsy from baseline or first sampling period. (TIF) [file pgen.1003553.s004.tif]

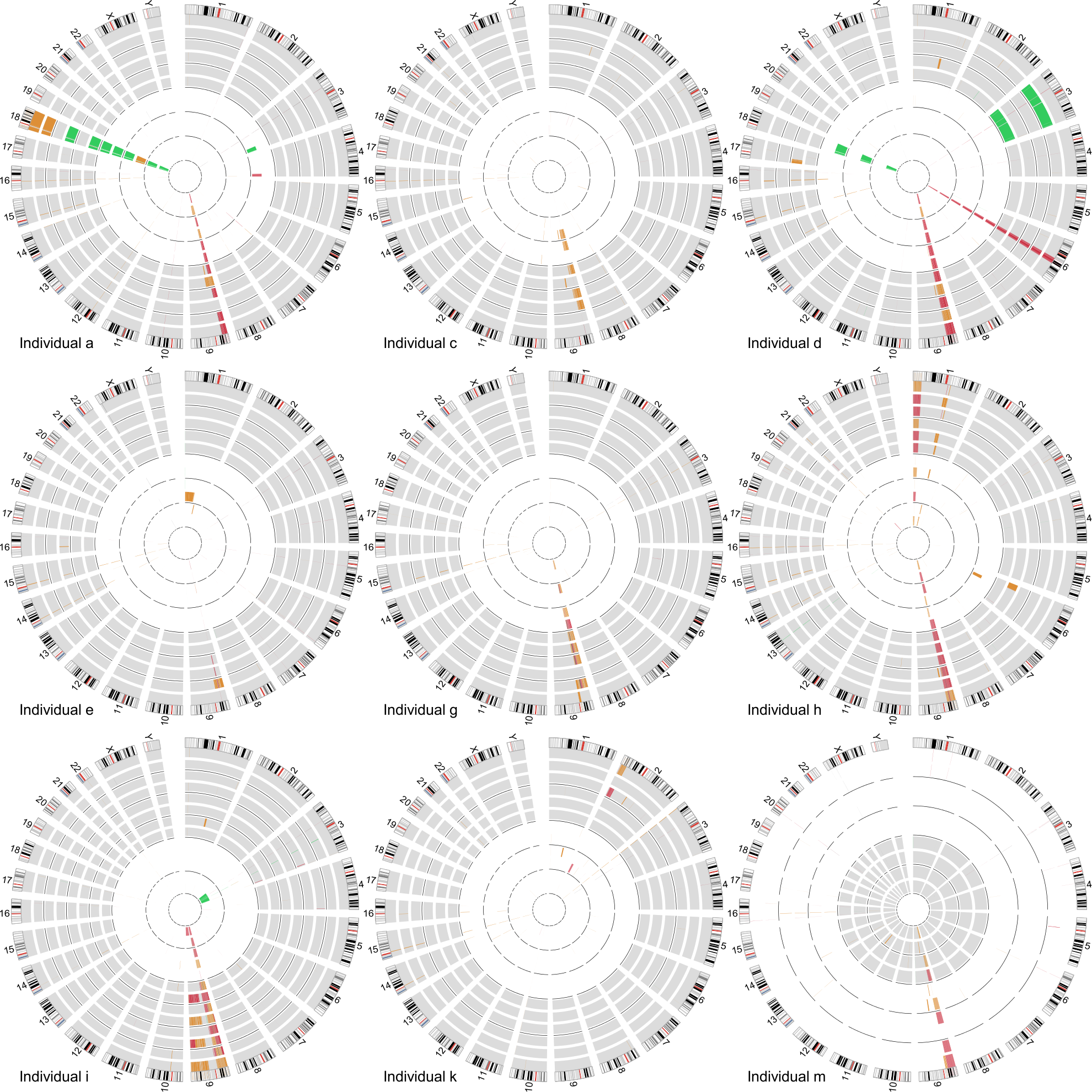

Supplement: Figure S4 — Circos plots of individuals a, c, d, e, g, h, i, k, and m. Each ring represents whole-genome SGA data from a different biopsy. Thin black line rings separate endoscopies (time points), white background shows time periods off-NSAIDs and gray background shows time periods on-NSAIDs. Within the rings, black segments designate homozygous deletion, red single copy loss, orange copy-neutral LOH, and green shows copy gain. (TIF) [file pgen.1003553.s005.tif]

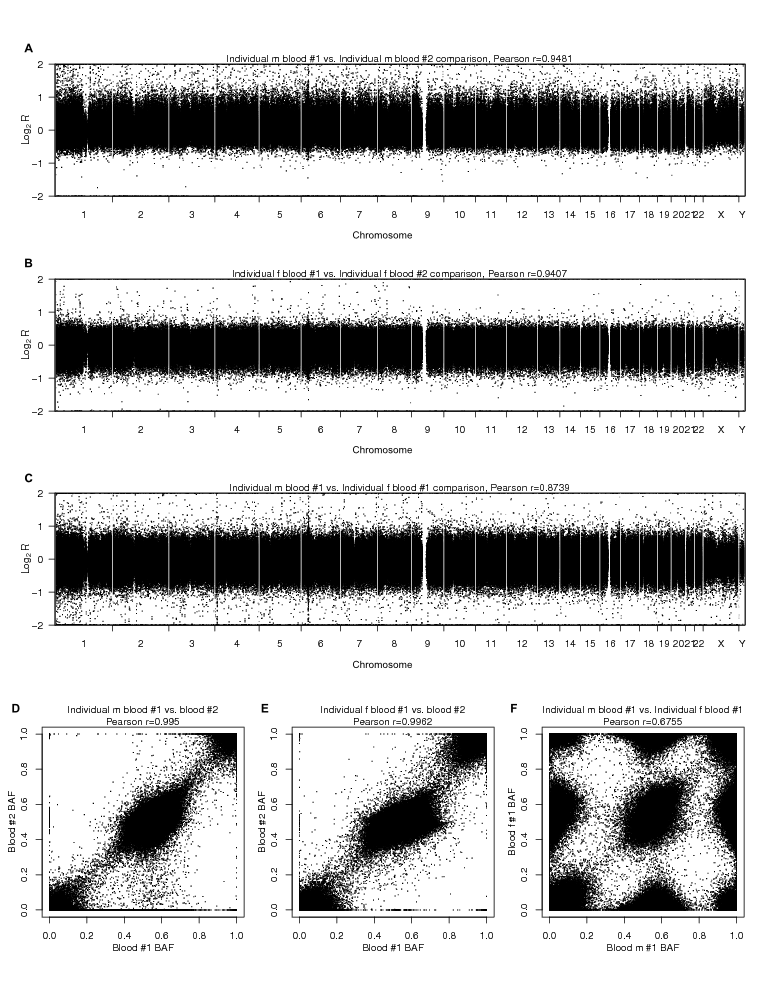

Supplement: Figure S11 — Log2 R ratio between replicate blood samples from the same individual (m and f, panels A and B, respectively) showed no detectable large-scale alterations and high Pearson correlation of SNP probe total intensity R (Pearson r = 0.9481 and r = 0.9407, for individual m and f, respectively). B allele frequency showed high correlation within replicate blood samples from the same individual (Pearson r = 0.995 and r = 0.9962, for individuals m and f, panels D and E, respectively). Log2 R ratio between two blood samples from two different individuals (m and f) showed no detectable large-scale alterations and decreased Pearson correlation between SNP probes total intensity R (Pearson r = 0.8739, panel C). B allele frequency between blood samples from different individuals showed that SNP probes can fall into all nine possible genotype categories reflecting the difference between germline genotype of individuals (Pearson r = 0.6755, panel F). Supplementary Table S5 describes blood sample details. (TIF) [file pgen.1003553.s012.tif]

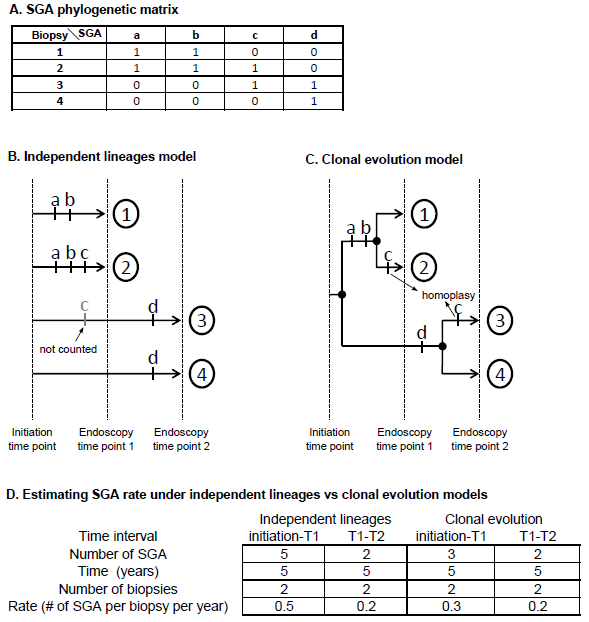

Supplement: Figure S12 — An individual-specific SGA matrix (panel A) is used as example to show the conceptual difference between using summary statistics and using phylogenies to compute the rate of acquisition of new SGA. The individual-specific SGA phylogenetic matrix shows the SGA molecular states absence/presence (0/1) for 4 biopsies (numbered 1–4, rows) and for 4 SGA characters/events (lettered a–d, columns). According to an independent lineages model, each biopsy samples a clone that is evolving independently. Here de novo SGA are counted simply by counting SGA events that are never observed in previous time periods (panel B). According to a clonal evolution model, acquisition of SGA events is assumed to occur strictly on a phylogenetic tree that maximizes the number of shared SGA events (for example, events a and b are observed in biopsies 1 and 2, therefore they most likely occurred on a branch prior to their most recent common ancestor; similarly event d most likely occurred once on the branch leading to the most recent common ancestor of biopsies 3 and 4, and minimizes the number of homoplasies (for example, event c is observed in biopsies 2 and 3, therefore it appears twice on branches of the phylogeny most likely due to convergent evolution) (panel C). There is uncertainty in estimating the dates of most recent common ancestors (internal nodes in the phylogeny) therefore we used BEAST with a minimal set of assumptions to estimate the rates of acquisition of SGA events during off-NSAID and on-NSAID time periods. We show how the two models may differ when estimating the SGA rate during two time periods (panel D). We have used both models when estimating SGA rate off- and on-NSAID (Figures S13 and 6), and found consistent results that NSAIDs reduce acquisition of SGAs. (TIF) [file pgen.1003553.s013.tif]

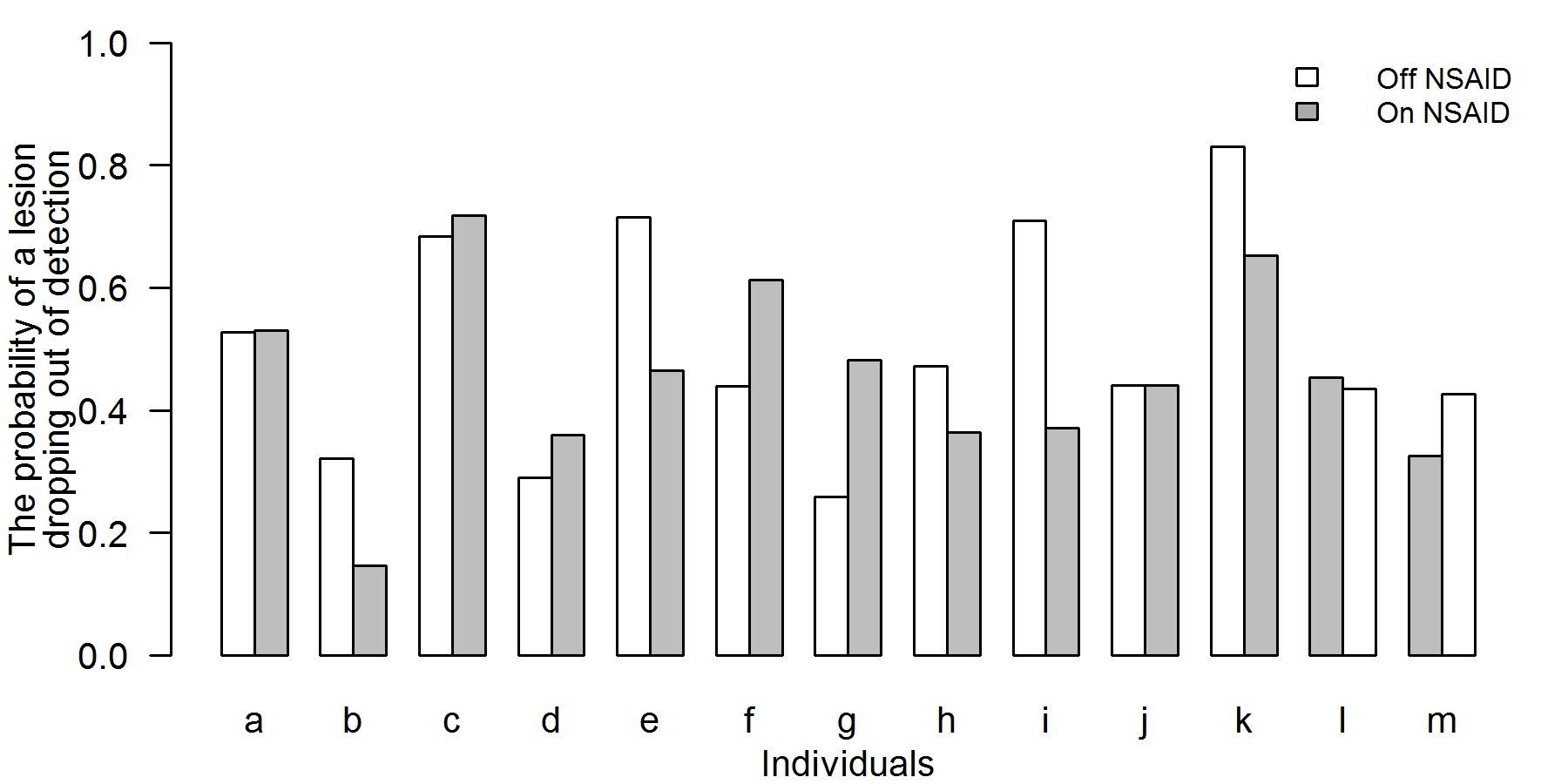

Supplement: Figure S14 — Dropout SGA events analysis. For each individual, the probability of a lesion dropping out of detection 1-P was estimated (y-axis). In 7 out of 13 individuals the probability of a lesion dropping out of detection was higher on-NSAIDs. (TIF) [file pgen.1003553.s015.tif]

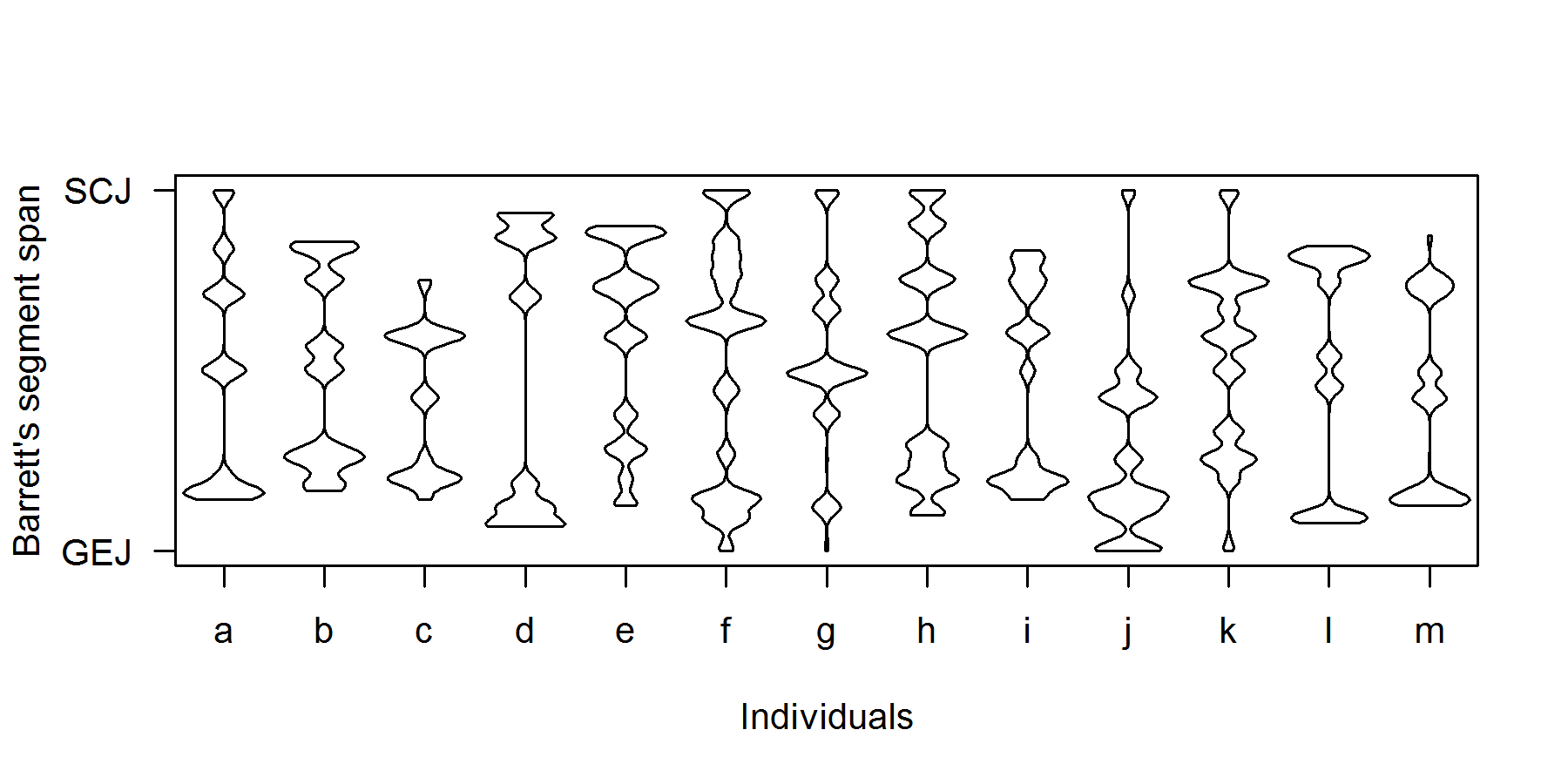

Supplement: Figure S15 — Spatial distribution of SGA load within Barrett's segments. No consistent and striking differences were found between SGA load in the upper level of the Barrett's segment (SCJ, squamocolumnar junction) compared to the lower level (GEJ, gastroesophageal junction). Note that previous analyses of the Seattle Barrett's Esophagus cohort have not found any preferential level of occurrence of adenocarcinoma in relation to SCJ and GEJ boundaries of Barrett's segments. (TIF) [file pgen.1003553.s016.tif]
