# Supplementary material for: NSAIDs Modulate Clonal Evolution in Barrett's Esophagus
Source: PLoS Genet. 2013 Jun 13;9(6):e1003553. doi: 10.1371/journal.pgen.1003553 (PMC3681672; doi:10.1371/journal.pgen.1003553)
Supplement: Figure S6 — Genetic divergence, estimated as average pairwise SGA-based Hamming distance, between biopsies over time and space (y-axis). For each individual (individuals a–m, rows), column 1 shows genetic divergences among biopsies within time points over individual follow-up time (the x-axis represents follow-up time); column 2 shows genetic divergences only among biopsies that are within ±1 cm of each other regardless of the time point of sampling (x-axis represents temporal distance in years); and column 3 shows genetic divergences only among biopsies that are within the same time point (x-axis represents spatial distance between pairs of biopsies in cm). (PDF) [file pgen.1003553.s007.pdf]

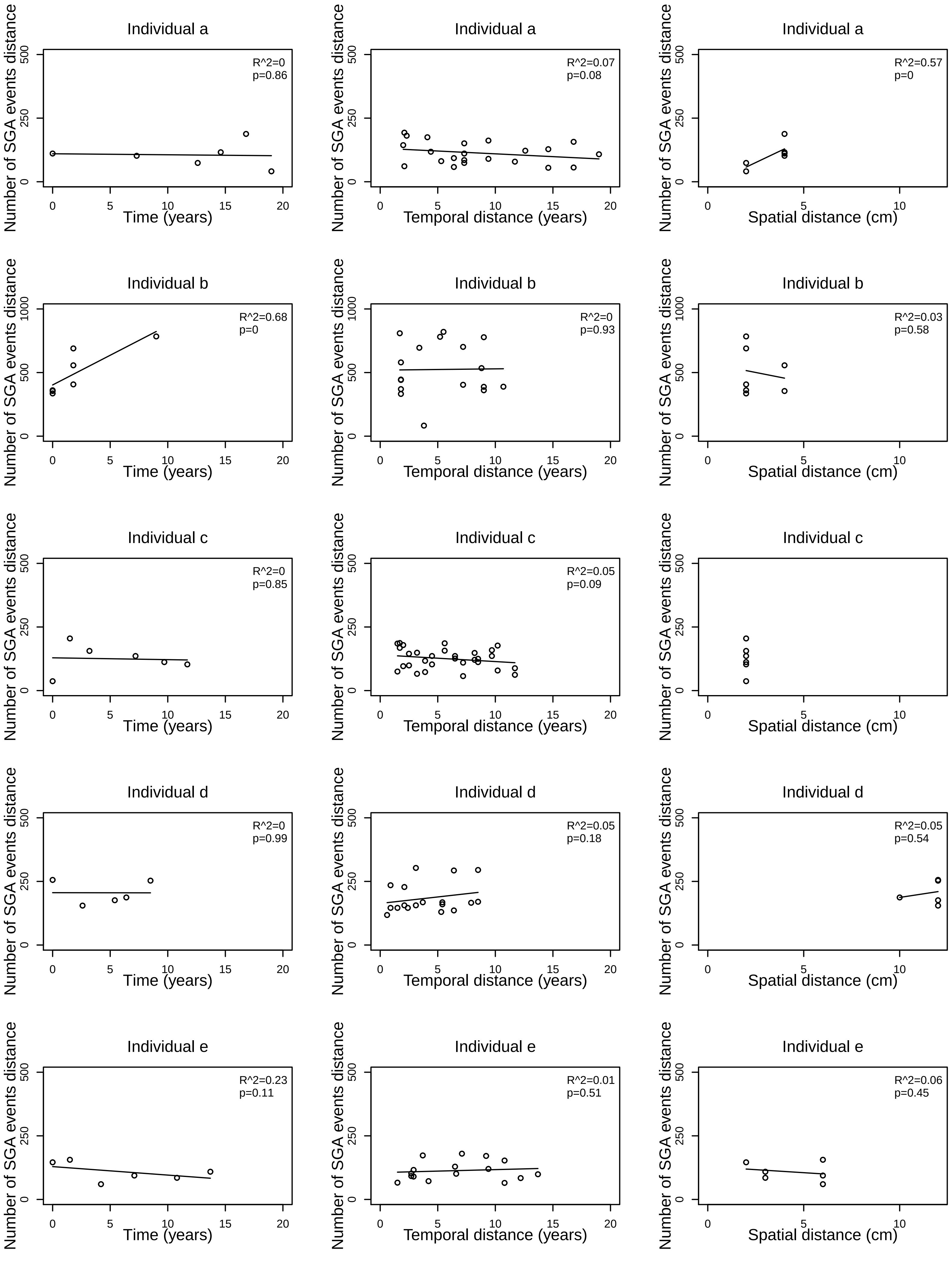

Individual a
R^2=0
p=0.86
Time (years)
Number of SGA events distance
Individual a
R^2=0.07
p=0.08
Temporal distance (years)
Number of SGA events distance
Individual a
R^2=0.57
p=0
Spatial distance (cm)
Number of SGA events distance
Individual b
R^2=0.68
p=0
Time (years)
Number of SGA events distance
Individual b
R^2=0
p=0.93
Temporal distance (years)
Number of SGA events distance
Individual b
R^2=0.03
p=0.58
Spatial distance (cm)
Number of SGA events distance
Individual c
R^2=0
p=0.85
Time (years)
Number of SGA events distance
Individual c
R^2=0.05
p=0.09
Temporal distance (years)
Number of SGA events distance
Individual c
Spatial distance (cm)
Number of SGA events distance
Individual d
R^2=0
p=0.99
Time (years)
Number of SGA events distance
Individual d
R^2=0.05
p=0.18
Temporal distance (years)
Number of SGA events distance
Individual d
R^2=0.05
p=0.54
Spatial distance (cm)
Number of SGA events distance
Individual e
R^2=0.23
p=0.11
Time (years)
Number of SGA events distance
Individual e
R^2=0.01
p=0.51
Temporal distance (years)
Number of SGA events distance
Individual e
R^2=0.06
p=0.45
Spatial distance (cm)
Number of SGA events distance

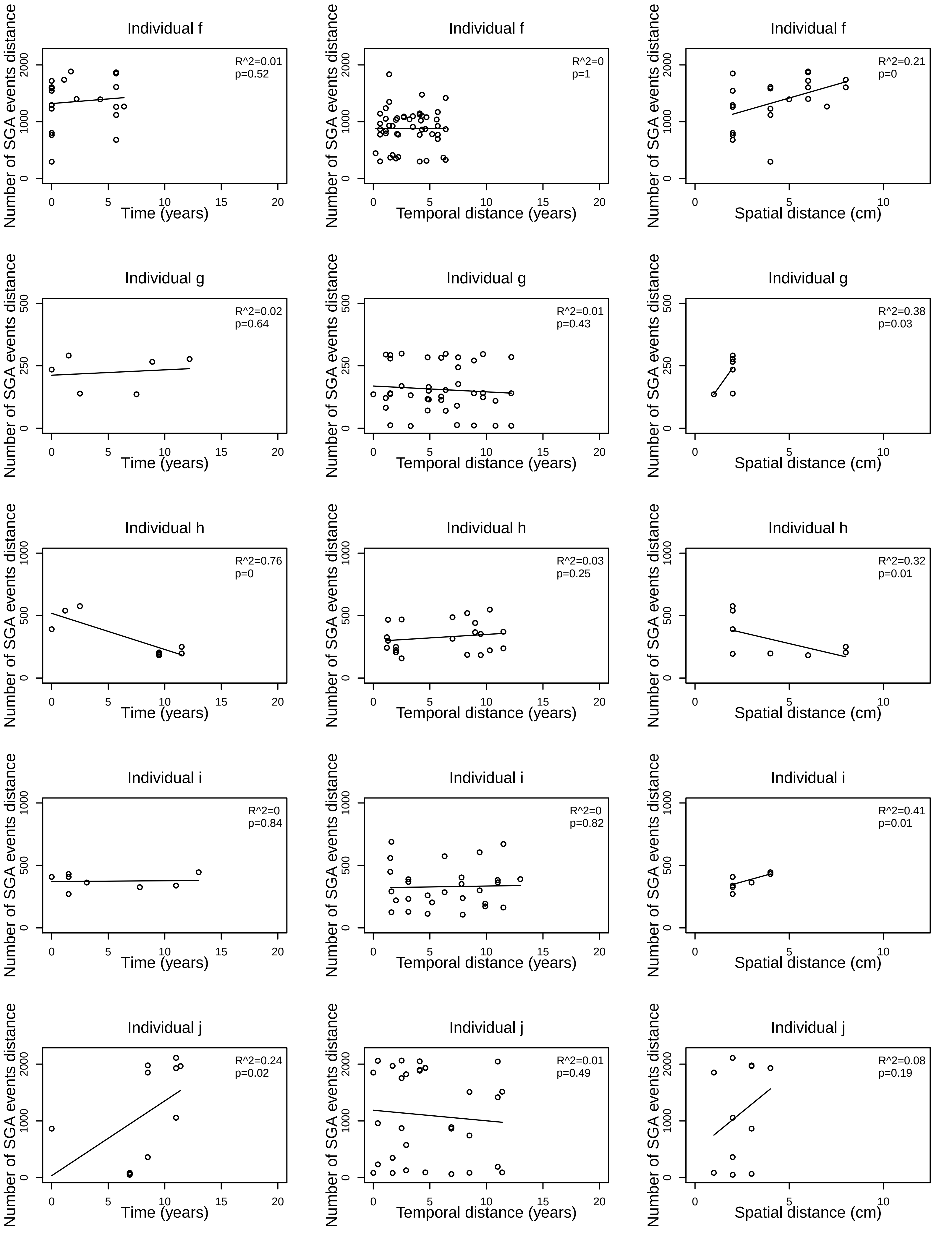

Individual f
Number of SGA events distance
Time (years)
R^2=0.01
p=0.52
Individual f
Temporal distance (years)
R^2=0
p=1
Individual f
Spatial distance (cm)
R^2=0.21
p=0
Individual g
Time (years)
R^2=0.02
p=0.64
Individual g
Temporal distance (years)
R^2=0.01
p=0.43
Individual g
Spatial distance (cm)
R^2=0.38
p=0.03
Individual h
Time (years)
R^2=0.76
p=0
Individual h
Temporal distance (years)
R^2=0.03
p=0.25
Individual h
Spatial distance (cm)
R^2=0.32
p=0.01
Individual i
Time (years)
R^2=0
p=0.84
Individual i
Temporal distance (years)
R^2=0
p=0.82
Individual i
Spatial distance (cm)
R^2=0.41
p=0.01
Individual j
Time (years)
R^2=0.24
p=0.02
Individual j
Temporal distance (years)
R^2=0.01
p=0.49
Individual j
Spatial distance (cm)
R^2=0.08
p=0.19

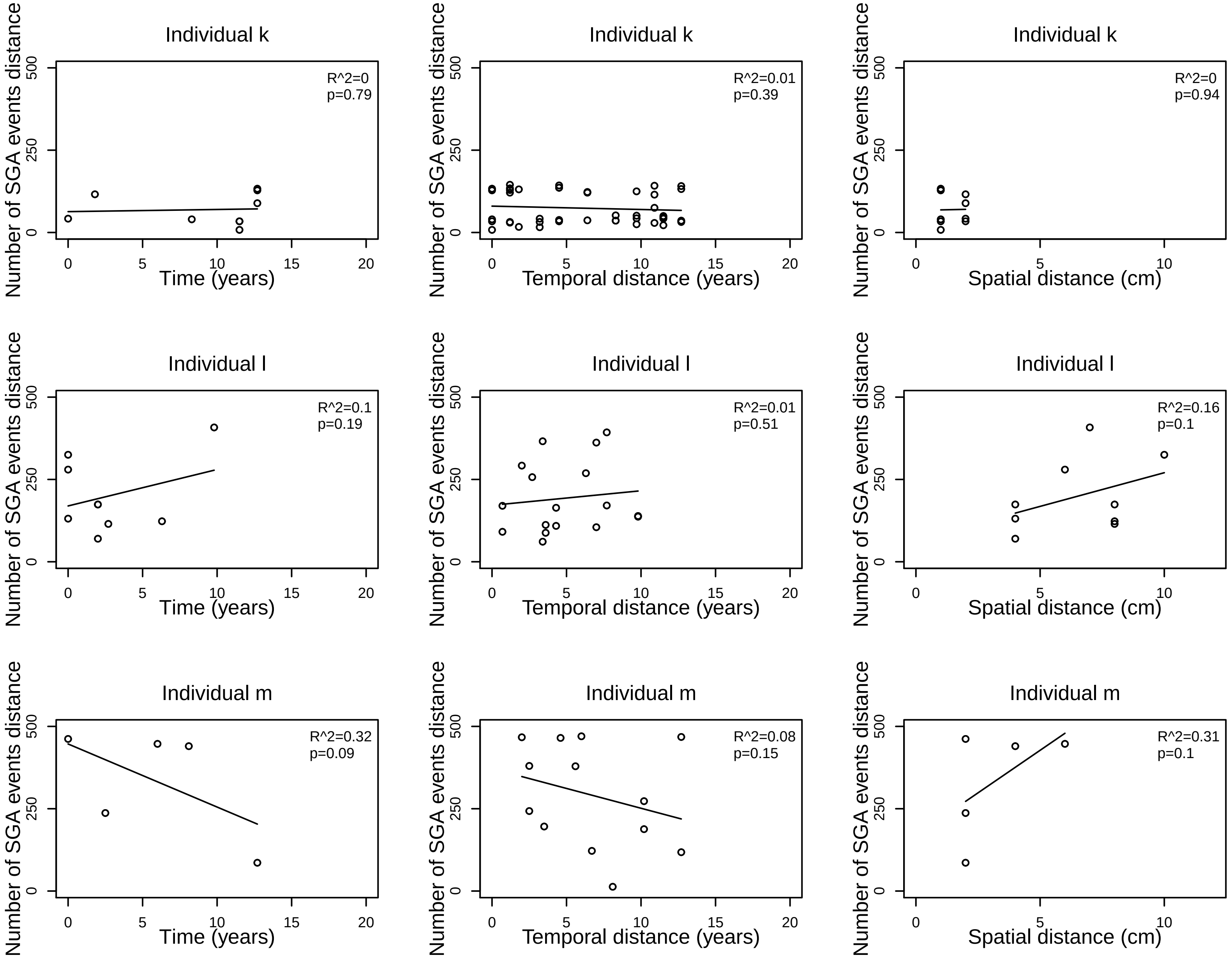
Individual k
R^2=0
p=0.79
Time (years)
Number of SGA events distance
Individual k
R^2=0.01
p=0.39
Temporal distance (years)
Number of SGA events distance
Individual k
R^2=0
p=0.94
Spatial distance (cm)
Number of SGA events distance
Individual l
R^2=0.1
p=0.19
Time (years)
Number of SGA events distance
Individual l
R^2=0.01
p=0.51
Temporal distance (years)
Number of SGA events distance
Individual l
R^2=0.16
p=0.1
Spatial distance (cm)
Number of SGA events distance
Individual m
R^2=0.32
p=0.09
Time (years)
Number of SGA events distance
Individual m
R^2=0.08
p=0.15
Temporal distance (years)
Number of SGA events distance
Individual m
R^2=0.31
p=0.1
Spatial distance (cm)
Number of SGA events distance
