# Supplementary material for: NSAIDs Modulate Clonal Evolution in Barrett's Esophagus
Source: PLoS Genet. 2013 Jun 13;9(6):e1003553. doi: 10.1371/journal.pgen.1003553 (PMC3681672; doi:10.1371/journal.pgen.1003553)
Supplement: Figure S7 — Genetic divergence, estimated as average pairwise proportion of differentially altered genome, between biopsies over time and space (y-axis). The three columns of plots are identical to those in Figure S6; only the y-axis has changed. (PDF) [file pgen.1003553.s008.pdf]

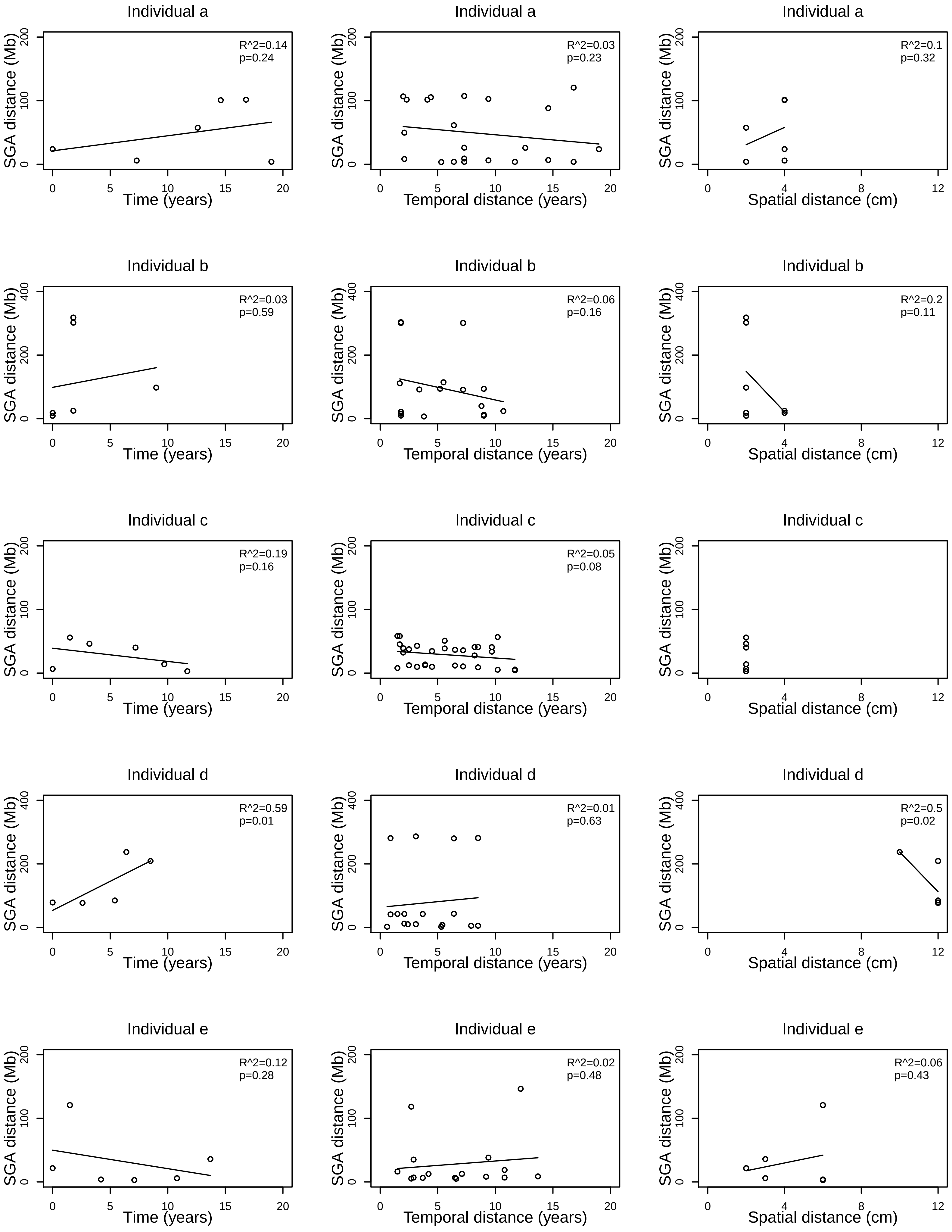

Individual a
R^2=0.14
p=0.24
SGA distance (Mb)
Time (years)
Individual a
R^2=0.03
p=0.23
SGA distance (Mb)
Temporal distance (years)
Individual a
R^2=0.1
p=0.32
SGA distance (Mb)
Spatial distance (cm)
Individual b
R^2=0.03
p=0.59
SGA distance (Mb)
Time (years)
Individual b
R^2=0.06
p=0.16
SGA distance (Mb)
Temporal distance (years)
Individual b
R^2=0.2
p=0.11
SGA distance (Mb)
Spatial distance (cm)
Individual c
R^2=0.19
p=0.16
SGA distance (Mb)
Time (years)
Individual c
R^2=0.05
p=0.08
SGA distance (Mb)
Temporal distance (years)
Individual c
SGA distance (Mb)
Spatial distance (cm)
Individual d
R^2=0.59
p=0.01
SGA distance (Mb)
Time (years)
Individual d
R^2=0.01
p=0.63
SGA distance (Mb)
Temporal distance (years)
Individual d
R^2=0.5
p=0.02
SGA distance (Mb)
Spatial distance (cm)
Individual e
R^2=0.12
p=0.28
SGA distance (Mb)
Time (years)
Individual e
R^2=0.02
p=0.48
SGA distance (Mb)
Temporal distance (years)
Individual e
R^2=0.06
p=0.43
SGA distance (Mb)
Spatial distance (cm)

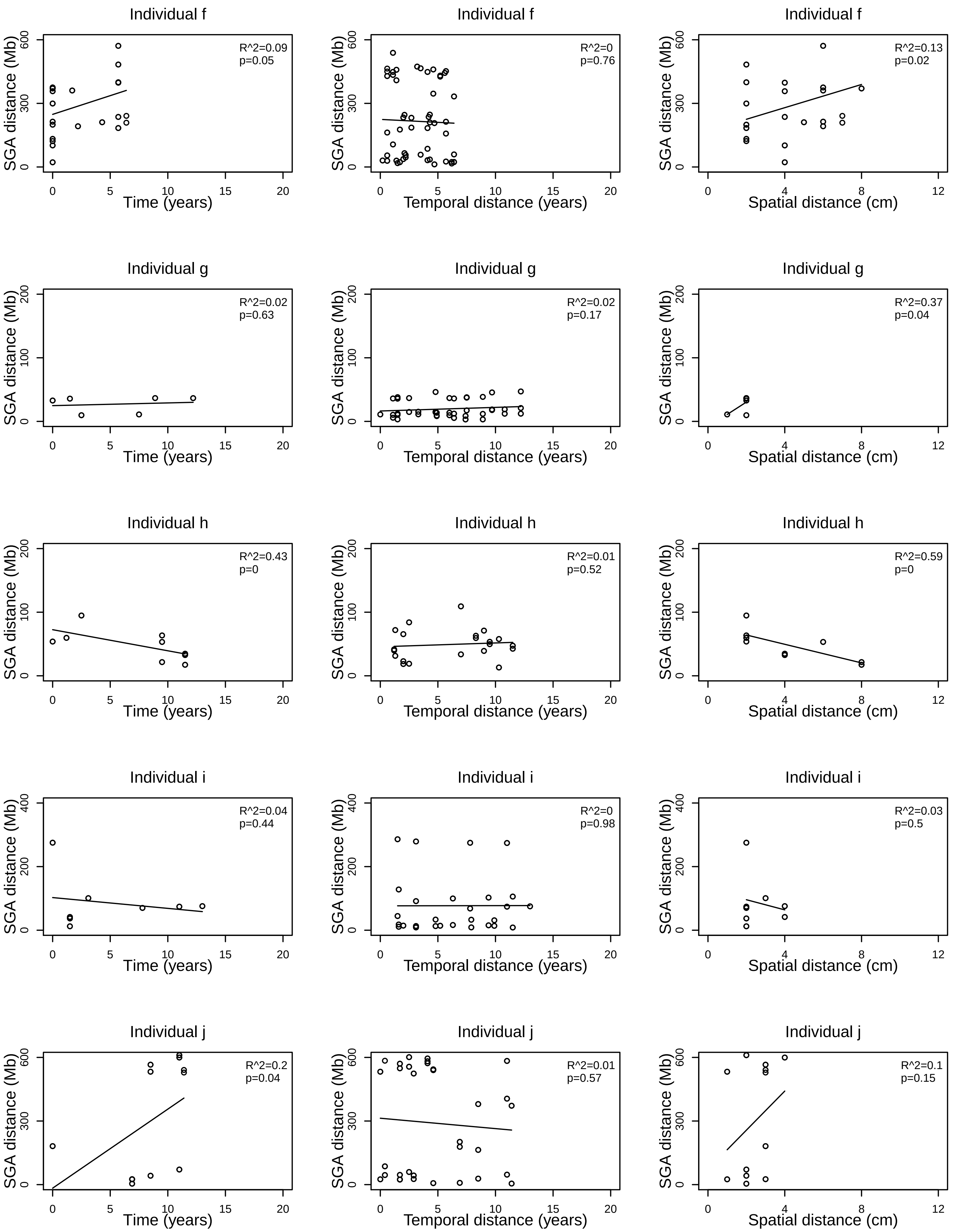
Individual f
R^2=0.09
p=0.05
SGA distance (Mb)
Time (years)
Individual f
R^2=0
p=0.76
SGA distance (Mb)
Temporal distance (years)
Individual f
R^2=0.13
p=0.02
SGA distance (Mb)
Spatial distance (cm)
Individual g
R^2=0.02
p=0.63
SGA distance (Mb)
Time (years)
Individual g
R^2=0.02
p=0.17
SGA distance (Mb)
Temporal distance (years)
Individual g
R^2=0.37
p=0.04
SGA distance (Mb)
Spatial distance (cm)
Individual h
R^2=0.43
p=0
SGA distance (Mb)
Time (years)
Individual h
R^2=0.01
p=0.52
SGA distance (Mb)
Temporal distance (years)
Individual h
R^2=0.59
p=0
SGA distance (Mb)
Spatial distance (cm)
Individual i
R^2=0.04
p=0.44
SGA distance (Mb)
Time (years)
Individual i
R^2=0
p=0.98
SGA distance (Mb)
Temporal distance (years)
Individual i
R^2=0.03
p=0.5
SGA distance (Mb)
Spatial distance (cm)
Individual j
R^2=0.2
p=0.04
SGA distance (Mb)
Time (years)
Individual j
R^2=0.01
p=0.57
SGA distance (Mb)
Temporal distance (years)
Individual j
R^2=0.1
p=0.15
SGA distance (Mb)
Spatial distance (cm)

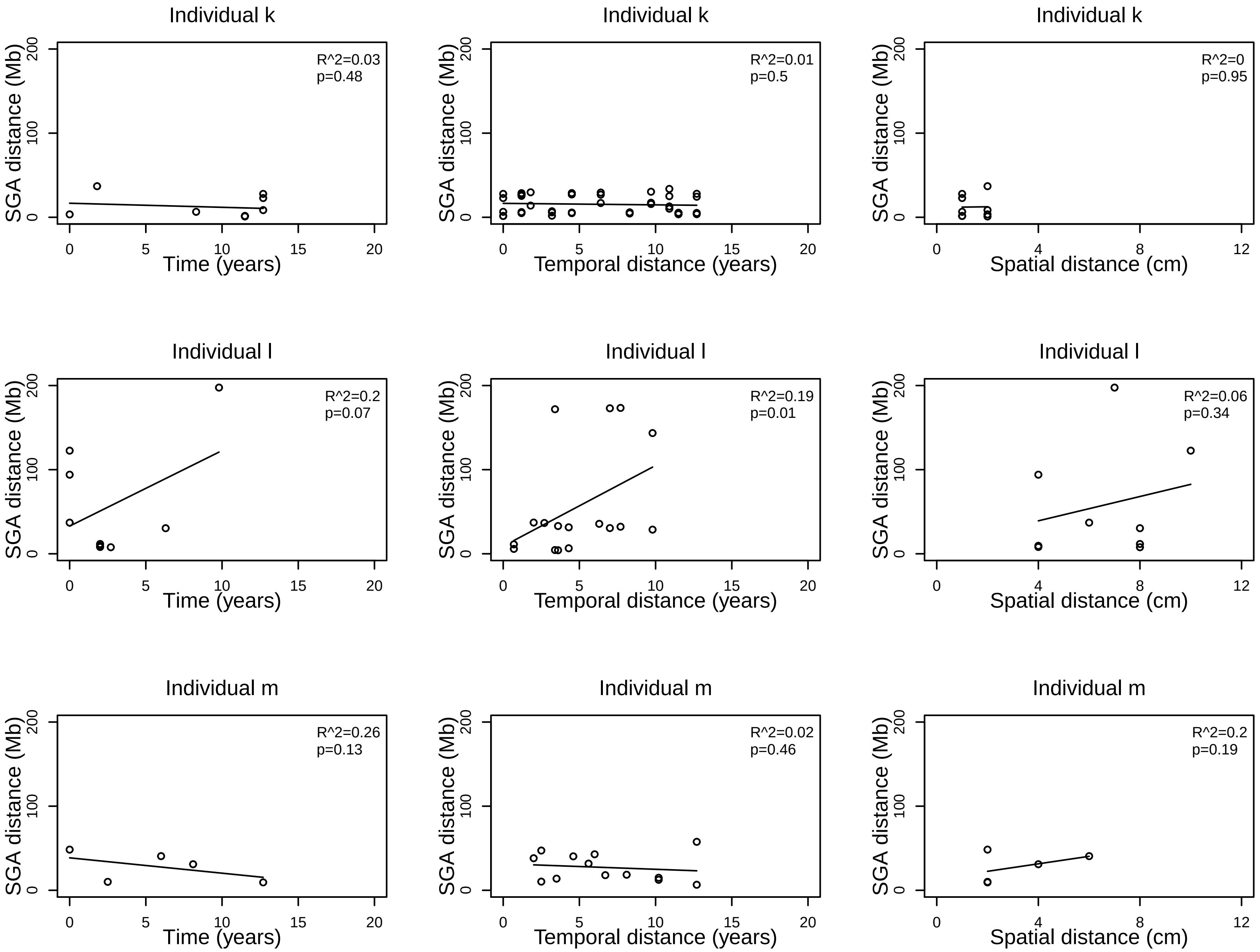
Individual k
R^2=0.03
p=0.48
SGA distance (Mb)
Time (years)
Individual k
R^2=0.01
p=0.5
SGA distance (Mb)
Temporal distance (years)
Individual k
R^2=0
p=0.95
SGA distance (Mb)
Spatial distance (cm)
Individual l
R^2=0.2
p=0.07
SGA distance (Mb)
Time (years)
Individual l
R^2=0.19
p=0.01
SGA distance (Mb)
Temporal distance (years)
Individual l
R^2=0.06
p=0.34
SGA distance (Mb)
Spatial distance (cm)
Individual m
R^2=0.26
p=0.13
SGA distance (Mb)
Time (years)
Individual m
R^2=0.02
p=0.46
SGA distance (Mb)
Temporal distance (years)
Individual m
R^2=0.2
p=0.19
SGA distance (Mb)
Spatial distance (cm)
