# Supplementary material for: NSAIDs Modulate Clonal Evolution in Barrett's Esophagus
Source: PLoS Genet. 2013 Jun 13;9(6):e1003553. doi: 10.1371/journal.pgen.1003553 (PMC3681672; doi:10.1371/journal.pgen.1003553)
Supplement: Text S1 — Additional methods for modeling somatic evolution in the crypt structured Barrett's segment that supplement BEAST phylogenetic analysis. This includes equations for estimating crypt doubling time and the initiation phase duration, as well as the substitution matrix (transition rate matrix) and continuous time solutions for the probability of transitions for the evolution of SGAs. (DOC) [file pgen.1003553.s023.doc]

**Supplementary Text S1. Cell organization into crypts in Barrett’s Esophagus**

To understand how evolution at the genome level unfolds, we need to first consider how cells are spatially organized in the BE tissue. The BE epithelium is a single layer of specialized intestinal metaplasia that typically covers 38 cm^2^ and contains between 10,000-400,000 crypts (Table S4). Crypts hold a reservoir of stem cells at their base that are self-renewing cells capable of generating and propagating genomic alterations over long timespans. Cell proliferation and differentiation occurs at the base of the crypts producing a flux of differentiated cells that move up the crypt and slough off into the lumen. Because of this constant shedding of cells, cell fitness in a crypt-structured epithelium is a complex combination of stem cell self-renewal, survival, and lateral spread, taking over neighboring crypts. Acquired SGAs that boost any of these three cell phenotypes will persist and increase in frequency in the BE cell population. The organization of cells into crypts in itself is an evolved mechanism to protect against cancer since acquired SGAs in differentiated cells can be lost by cells sloughing off and only acquired SGAs in stem cells can persist and increase in frequency over time; and also, in a strictly single-layer columnar epithelium, lateral invasion of mutant cells into neighboring crypts is physically difficult since invading from the lumen side requires going against the flux of cells [1]. A subset of SGAs were detected in all biopsies of an individual over decades (Figure S5) which is evidence that these SGAs must be acquired in long-living self-renewing (stem) cells that have not differentiated, been sloughed into the lumen, and have survived toxic microenvironmental exposures. Other SGAs had various lifespans in the segment (Figures 2, 4B, 4C, 5B, 5C, S3) but only a fraction of them were turning over, either appearing or regressing (Figure S13). In summary, the spatial organization of cells into a single-layer crypt-structured BE epithelium constrains the evolutionary dynamics and spatial dispersal of acquired SGAs over time.

We assumed that the BE segment is initiated by a change in a single cell. According to this model, BE is initiated from a single progenitor stem cell that undergoes initial clonal expansion that forms a single progenitor crypt, and further population growth of the stem cell population occurs by crypt fission (branching). Initiation of the single progenitor stem cell could be a result of transdifferentiation of a stem cell originally supporting squamous epithelium differentiation through activation of multiple molecular pathways, including expression of homeobox CDX genes [2–4], or could be a part of a residual population left over from early embryonic development [5,6] that exists at the gastroesophageal junction. Even if the initiation of BE does not start from a single cell, the fact that in almost all cases, all the biopsies within a patient share some identical lesions the first time they undergo endoscopy suggests that even if BE is not initially clonal, it quickly becomes so. We assumed a crypt cycle model [7] of crypt population growth and assuming a logistic growth (rather than exponential), the time it takes for a single originating crypt to grow to a population of 36,132 crypts would be approximately 4.81 years prior to detection at baseline endoscopy (Table S4).

**Supplementary Equation S1:** $T_{r}=\frac{T_{b}\log2}{\log(1+I_{b})}$

**Supplementary Equation S1.** Estimating the crypt doubling time where the duration of branching (T­_b_) is set to 20 days (see Appendix I from [8]). *I_b_* is the fraction of crypts that appear to be branching in standard pathology slides of BE biopsies.

**Supplementary Equation S2:** $T_{init}=\frac{T_{r}}{\log2}\log\frac{N_{t}(K-N_{0})}{(K-N_{t})N_{0}}$

**Supplementary Equation S2.** Estimating the initiation phase duration, where *N_0_*=1 is the starting population size that is fixed to 1 crypt. *K* is the estimated maximum number of crypts (“carrying capacity”) in a BE segment, *N_t_* is the estimated total number of crypts in a BE segment, and *T_r_* is the crypt doubling time defined in Supplementary Equation S1.

|  | To SGA absence (0) | To SGA presence (1) |
| --- | --- | --- |
| From SGA absence (0) | 1-α | Α |
| From SGA presence(1) | 0 | 1 |

**Supplementary Equation S3:**

**Supplementary Equation S3.** Substitution matrix (transition rate matrix) describing the transition probabilities (rates) between SGA absence/presence (0/1) states that was incorporated into BEAST. The probability α is the probability of acquiring SGA in a segment in one division (at the biopsy level), which is estimated by the BEAST software.

**Supplementary Equation S4:**

**Supplementary Equation S4.** Continuous-time solutions for the probability of all four possible state transitions of a character on a branch of a phylogeny that has branch length of *t* years.

**References:**

1. Cairns J (1975) Mutation selection and the natural history of cancer. Nature 255: 197–200.

2. Chang C-L, Lao-Sirieix P, Save V, De La Cueva Mendez G, Laskey R, et al. (2007) Retinoic acid-induced glandular differentiation of the oesophagus. Gut 56: 906–917.

3. Souza RF, Krishnan K, Spechler SJ (2008) Acid, bile, and CDX: the ABCs of making Barrett’s metaplasia. American journal of physiology Gastrointestinal and liver physiology 295: G211–8.

4. Badreddine RJ, Wang KK (2010) Barrett esophagus: an update. Nature reviews Gastroenterology & hepatology 7: 369–378.

5. Johns B a E (1952) Developmental changes in the oesophageal epithelium in man. Journal of anatomy 86: 431–442.

6. Wang X, Ouyang H, Yamamoto Y, Kumar PA, Wei TS, et al. (2011) Residual Embryonic Cells as Precursors of a Barrett’s-like Metaplasia. Cell 145: 1023–1035.

7. Totafurno J, Bjerknes M, Cheng H (1987) The crypt cycle. Crypt and villus production in the adult intestinal epithelium. Biophysical Journal 52: 279–294.

8. Cheng H, Matthew Bjerknes, Jack Amar, Geoffrey Gardiner (1986) Crypt production in normal and diseased human colonic epithelium. The Anatomical Record 216: 44–48.
