# Supplementary material for: NSAIDs Modulate Clonal Evolution in Barrett's Esophagus
Source: PLoS Genet. 2013 Jun 13;9(6):e1003553. doi: 10.1371/journal.pgen.1003553 (PMC3681672; doi:10.1371/journal.pgen.1003553)
Supplement: Text S2 — Additional methods for calculating genetic divergence between biopsies separated by space and time. (DOC) [file pgen.1003553.s024.doc]

**Supplementary Text S2. Calculating genetic divergence between biopsies separated by time and space**

We use two distance measures: average pairwise SGA event-based Hamming distance and average pairwise proportion of differentially altered genome; to quantify the genetic distance between two biopsies. The term genetic divergence is commonly defined as the average number of differences per locus (or per site, where loci/sites are assumed to be homologous: derive from a common ancestor) between two individual genomes sampled from a population of the same species.

One way to measure genetic divergence is to count the average difference between molecular states (copy number loss/gain, CNLOH) assigned at each of 1 million SNPs between two biopsies. This would overestimate genetic divergence since typically the size of SGA lesions is large (the average total amount of SGA per biopsy was 96.5 Mb, see Table S1). Therefore, we measured genetic divergence by counting the average difference between molecular states assigned at each detected SGA segment (see *SGA detection* in Methods) between two biopsies, and we define this genetic divergence measure as “average pairwise SGA event-based Hamming distance” that is the y-axis in Figure S6.

Additionally, two biopsies may differ only by a few events, which would lead to small average pairwise SGA-based Hamming distance, but the distinct events may be large in size. Difference in few, but large, SGA may be biologically significant therefore as secondary measure we calculate genetic divergence by adding up the total amount of SGA difference at each detected SGA segment and dividing the total by the number of SGA segments (see *SGA detection* in Methods) between two biopsies; and we define this measure as “average pairwise proportion of differentially altered genome” that is the y-axis in Figure S7.

In both Figures S6 and S7 we fit a linear model through the measured genetic distances between biopsies (open circles) using the “lm” and “summary.lm” functions in R [8], where the p-value represents a slope of the fitted line that is significantly different from 0 and the R^2^ value represents the fraction of variance explained by the model.
