# Supplementary material for: NSAIDs Modulate Clonal Evolution in Barrett's Esophagus
Source: PLoS Genet. 2013 Jun 13;9(6):e1003553. doi: 10.1371/journal.pgen.1003553 (PMC3681672; doi:10.1371/journal.pgen.1003553)
Supplement: Text S3 — Alternative non-phylogeny-based methods for analysis of SGA occurrences and regressions during on- and off- NSAID periods. (DOC) [file pgen.1003553.s025.doc]

**Supplementary Text S3. Analysis of appearance of new SGAs and regression of pre-existing SGAs on- and off-NSAIDs**

Here we provide alternative analyses of SGA data, which do not rely on estimating phylogenies (maximum parsimony using PAUP or maximum likelihood using BEAST). While phylogenies are the best tool to account for common ancestry among biopsies within an individual, several interesting facts can be learned from alternative analysis methods that provide summary statistics of the SGA data.

First, we considered an independent lineages model of evolutionary dynamics of SGA within an individual. For example, the SGA phylogenetic matrix shown in Figure S12 consists of four biopsies (numbered 1-4) in which we detected four SGA events (labeled a-d). We modeled of the dynamics of SGA in the main text according to a clonal evolution model of SGA (Figure S12 C) by using PAUP and BEAST. Both phylogeny methods would work on these data by estimating a phylogeny that minimizes the number of homoplasies (or independent, parallel acquisitions) in the data.

In Figure S12 C, SGA events a and b are seen in biopsies 1 and 2, and SGA event c is seen in biopsies 2 and 3, which creates a decision of whether to pair biopsies 1 and 2 leading to 1 homoplasy of event c, or whether to pair biopsies 2 and 3 leading to 2 homoplasies of events a and b. Since phylogenies minimize the number of homoplasies biopsies 1 and 2 end up paired.

In Figure S12 B, we consider that a deterministic chronological order of SGA events occur in an individual. In this case, SGA event occurred at time 0 and hit two independent lineages leading to biopsies 1 and 2. After this, SGA event b occurred at time 1 and hit the same two lineages leading to biopsies 1 and 2. After this, SGA event c occurred at time 3 and hit two independent lineages leading to biopsies 2 and 3. And lastly, at time 4, SGA event d occurred also hitting two lineages leading to biopsies 3 and 4. According to this model, we posit four independent lineages representing four independent self-renewing stem cell populations. And the fact that biopsy 2 has been observed at endoscopy time point 1 forces the temporal placement of the SGA event c, on the lineage leading to biopsy 3, before endoscopy time point 1. According to this scenario, if time period initiation-1 is a period off-NSAIDs and time period 1-2 is a period on-NSAIDs, we would count for on-NSAIDs SGAs (a,b) for biopsy 1, and (a,b,c) for biopsy 2, but only (d) for biopsy 3 and only (d) for biopsy 4. The SGA event (c) that occured on lineage 3, occurred off-NSAIDs according to the chronological order assumption, so it does not contribute to the count of SGA events in time period 1-2.

A caveat to this analysis is the assumption of a chronological ordering of SGA events that is, in this case that the events (ab), (c) and (d) occur one after the other in chronological time. As alternative, all SGA events can be counted as observed, that is, biopsy 1 having (ab), biopsy 2 having (abc), biopsy 3 having (cd) and biopsy 4 having (d), and the results of that is shown visually in Figures S1 and S2, where individual differences between SGA on- and off- NSAIDs can be discerned.

To test for more subtle effects of NSAIDs on the accumulation of SGAs, we evaluated newly-appearing SGAs during periods of NSAID use and NSAID non-use by excluding all SGAs that were detected at baseline. A new SGA was defined as a lesion in a biopsy that had not been seen in any previous time point. Baseline SGAs were not counted as new since they had occurred and increased in frequency for an unknown amount of time prior to detection at baseline and since we have self-reported NSAID use information reaching back only 6 months prior to baseline. We also evaluated whether NSAID use is associated with regression of pre-existing SGAs (dropping out of detection), by evaluating only SGAs that are detected at baseline, or during NSAID use or non-use periods, but not detected in the last endoscopy. With these assumptions, we counted newly-appearing SGA events and SGA events dropping out of detection (regressions of pre-existing SGAs) off-NSAIDs and on-NSAIDs for all individuals (Figure S13).

Across all individuals, we detected a median of 3.86 (95\% range: 0.54-81) newly appearing SGAs per biopsy during on-NSAID periods (n=73 biopsies) compared to a median of 7.33 (95\% range: 1.19-209) newly appearing SGAs per biopsy during off-NSAID periods (n=57 biopsies), which was a significant difference (Wilcoxon test, p=0.027) (Figure S13 A). A significantly greater number of existing SGA events dropped out of detection on-NSAIDs, as compared to off-NSAIDs (median = 9.29, 95\% range: 0.46-58 [n=79] vs. median = 3.19, 95\% range: 1.49-788 [n=55], Wilcoxon test, p<0.001) (Figure S13 B).

In summary, NSAID use was associated with a reduced number of newly appearing SGAs of any size (Figure S13 A) and with higher number of pre-existing SGAs of any size dropping out of detection (Figure S13 B).

Interestingly, Figure S13 B suggests that clones are regressing during NSAID use. Many of the lesions assayed, including LOH and homozygous deletions, are irreversible. The apparent disappearance of SGAs is due to two dynamics: (1) Failure to sample a clone that is still present at the later time point and (2) regression of clones carrying those lesions, to the extent that they either go extinct or are just more difficult to sample. We expect that there are many clones present in the Barrett's segments that were not sampled in our study, with only 2-3 biopsies per time point. However, the phenomenon of lesions seeming to appear and disappear is unlikely to be explained purely by sampling errors. Sampling errors apply to both on NSAID and off NSAID periods equally, since the same number of biopsies were taken under both exposures (and we have normalized the frequency of appearance and disappearance of lesion on a per biopsy basis). So any systematic difference between on and off NSAID periods must be due to a difference in the clonal dynamics. Our results suggest that clones with few new SGA lesions are outcompeting pre-existing clones during NSAID use, making the lesions of those regressing clones seem to disappear. We do not yet know if NSAIDs are selecting for or against particular SGAs.

This analysis of SGAs on- and off- NSAIDs is limited as both biopsies and the lineages leading to them are related by common ancestry, which can best be accounted for with phylogenetic analyses.
