# Supplementary material for: NSAIDs Modulate Clonal Evolution in Barrett's Esophagus
Source: PLoS Genet. 2013 Jun 13;9(6):e1003553. doi: 10.1371/journal.pgen.1003553 (PMC3681672; doi:10.1371/journal.pgen.1003553)
Supplement: Text S4 — Alternative method and analysis of SGA events dropping out of detection (or regressing) during on- and off- NSAID periods. (DOC) [file pgen.1003553.s026.doc]

**Supplementary Text S4. Alternative analysis of SGA events dropping out of detection**

Here we provide a supplementary analysis of regressions of pre-existing SGA events (SGA events dropping out of detection). Each individual had a period off NSAIDs and a period on NSAIDs. Each such period had several endoscopies, including a terminal (last) endoscopy for that period. For each period we counted the total number of distinct lesions in biopsies from non-terminal endoscopies, then asked what proportion of them were not observed in the terminal endoscopy. To correct for varying number of biopsies at the terminal endoscopy, we estimated the probability P of detection of a lesion in a single biopsy, so that failure to detect a lesion in B biopsies would have a probability of (1-P)^B. The probability P was estimated for each individual and period to see if P varied between on-NSAIDs and off-NSAIDs periods (Figure S14).

For each period (on or off NSAIDs) we observed some number of lesions in non-terminal endoscopies, and then for each lesion, we did or did not observe it at the terminal endoscopy (of that period). However the number of biopsies at the terminal endoscopy varies, so how to do a test? Obviously the more biopsies, the more lesions we expect to observe.

Let's assume that biopsies are independent and that there is a probability P of observing a lesion in a single terminal biopsy. This P is specific to each patient and period. We want to test if the P for on and the P for off differ across our set of patients.

The chance that a lesion will not be seen at the terminal endoscopy is (1-P)^B where B is the number of terminal biopsies. The chance that a lesion would be seen in one biopsy is P by definition. The chance of not seeing it in one biopsy is therefore 1-P. If biopsies are independent, the chance of not seeing it in B biopsies is (1-P)^B.

For example, if in one patient/period combination we saw 500 lesions prior to terminal endoscopy. We saw only 400 of them in terminal endoscopy which had 3 biopsies, then:
